# Sequencing of 53,831 diverse genomes from the NHLBI TOPMed Program

**DOI:** 10.1038/s41586-021-03205-y

**Published:** 2021-02-10

**Authors:** Daniel Taliun, Daniel N. Harris, Michael D. Kessler, Jedidiah Carlson, Zachary A. Szpiech, Raul Torres, Sarah A. Gagliano Taliun, André Corvelo, Stephanie M. Gogarten, Hyun Min Kang, Achilleas N. Pitsillides, Jonathon LeFaive, Seung-been Lee, Xiaowen Tian, Brian L. Browning, Sayantan Das, Anne-Katrin Emde, Wayne E. Clarke, Douglas P. Loesch, Amol C. Shetty, Thomas W. Blackwell, Albert V. Smith, Quenna Wong, Xiaoming Liu, Matthew P. Conomos, Dean M. Bobo, François Aguet, Christine Albert, Alvaro Alonso, Kristin G. Ardlie, Dan E. Arking, Stella Aslibekyan, Paul L. Auer, John Barnard, R. Graham Barr, Lucas Barwick, Lewis C. Becker, Rebecca L. Beer, Emelia J. Benjamin, Lawrence F. Bielak, John Blangero, Michael Boehnke, Donald W. Bowden, Jennifer A. Brody, Esteban G. Burchard, Brian E. Cade, James F. Casella, Brandon Chalazan, Daniel I. Chasman, Yii-Der Ida Chen, Michael H. Cho, Seung Hoan Choi, Mina K. Chung, Clary B. Clish, Adolfo Correa, Joanne E. Curran, Brian Custer, Dawood Darbar, Michelle Daya, Mariza de Andrade, Dawn L. DeMeo, Susan K. Dutcher, Patrick T. Ellinor, Leslie S. Emery, Celeste Eng, Diane Fatkin, Tasha Fingerlin, Lukas Forer, Myriam Fornage, Nora Franceschini, Christian Fuchsberger, Stephanie M. Fullerton, Soren Germer, Mark T. Gladwin, Daniel J. Gottlieb, Xiuqing Guo, Michael E. Hall, Jiang He, Nancy L. Heard-Costa, Susan R. Heckbert, Marguerite R. Irvin, Jill M. Johnsen, Andrew D. Johnson, Robert Kaplan, Sharon L. R. Kardia, Tanika Kelly, Shannon Kelly, Eimear E. Kenny, Douglas P. Kiel, Robert Klemmer, Barbara A. Konkle, Charles Kooperberg, Anna Köttgen, Leslie A. Lange, Jessica Lasky-Su, Daniel Levy, Xihong Lin, Keng-Han Lin, Chunyu Liu, Ruth J. F. Loos, Lori Garman, Robert Gerszten, Steven A. Lubitz, Kathryn L. Lunetta, Angel C. Y. Mak, Ani Manichaikul, Alisa K. Manning, Rasika A. Mathias, David D. McManus, Stephen T. McGarvey, James B. Meigs, Deborah A. Meyers, Julie L. Mikulla, Mollie A. Minear, Braxton D. Mitchell, Sanghamitra Mohanty, May E. Montasser, Courtney Montgomery, Alanna C. Morrison, Joanne M. Murabito, Andrea Natale, Pradeep Natarajan, Sarah C. Nelson, Kari E. North, Jeffrey R. O’Connell, Nicholette D. Palmer, Nathan Pankratz, Gina M. Peloso, Patricia A. Peyser, Jacob Pleiness, Wendy S. Post, Bruce M. Psaty, D. C. Rao, Susan Redline, Alexander P. Reiner, Dan Roden, Jerome I. Rotter, Ingo Ruczinski, Chloé Sarnowski, Sebastian Schoenherr, David A. Schwartz, Jeong-Sun Seo, Sudha Seshadri, Vivien A. Sheehan, Wayne H. Sheu, M. Benjamin Shoemaker, Nicholas L. Smith, Jennifer A. Smith, Nona Sotoodehnia, Adrienne M. Stilp, Weihong Tang, Kent D. Taylor, Marilyn Telen, Timothy A. Thornton, Russell P. Tracy, David J. Van Den Berg, Ramachandran S. Vasan, Karine A. Viaud-Martinez, Scott Vrieze, Daniel E. Weeks, Bruce S. Weir, Scott T. Weiss, Lu-Chen Weng, Cristen J. Willer, Yingze Zhang, Xutong Zhao, Donna K. Arnett, Allison E. Ashley-Koch, Kathleen C. Barnes, Eric Boerwinkle, Stacey Gabriel, Richard Gibbs, Kenneth M. Rice, Stephen S. Rich, Edwin K. Silverman, Pankaj Qasba, Weiniu Gan, NHLBI Trans-Omics for Precision Medicine (TOPMed) Consortium, Namiko Abe, Laura Almasy, Seth Ament, Peter Anderson, Pramod Anugu, Deborah Applebaum-Bowden, Tim Assimes, Dimitrios Avramopoulos, Emily Barron-Casella, Terri Beaty, Gerald Beck, Diane Becker, Amber Beitelshees, Takis Benos, Marcos Bezerra, Joshua Bis, Russell Bowler, Ulrich Broeckel, Jai Broome, Karen Bunting, Carlos Bustamante, Erin Buth, Jonathan Cardwell, Vincent Carey, Cara Carty, Richard Casaburi, Peter Castaldi, Mark Chaffin, Christy Chang, Yi-Cheng Chang, Sameer Chavan, Bo-Juen Chen, Wei-Min Chen, Lee-Ming Chuang, Ren-Hua Chung, Suzy Comhair, Elaine Cornell, Carolyn Crandall, James Crapo, Jeffrey Curtis, Coleen Damcott, Sean David, Colleen Davis, Lisa de las Fuentes, Michael DeBaun, Ranjan Deka, Scott Devine, Qing Duan, Ravi Duggirala, Jon Peter Durda, Charles Eaton, Lynette Ekunwe, Adel El Boueiz, Serpil Erzurum, Charles Farber, Matthew Flickinger, Myriam Fornage, Chris Frazar, Mao Fu, Lucinda Fulton, Shanshan Gao, Yan Gao, Margery Gass, Bruce Gelb, Xiaoqi Priscilla Geng, Mark Geraci, Auyon Ghosh, Chris Gignoux, David Glahn, Da-Wei Gong, Harald Goring, Sharon Graw, Daniel Grine, C. Charles Gu, Yue Guan, Namrata Gupta, Jeff Haessler, Nicola L. Hawley, Ben Heavner, David Herrington, Craig Hersh, Bertha Hidalgo, James Hixson, Brian Hobbs, John Hokanson, Elliott Hong, Karin Hoth, Chao Agnes Hsiung, Yi-Jen Hung, Haley Huston, Chii Min Hwu, Rebecca Jackson, Deepti Jain, Min A. Jhun, Craig Johnson, Rich Johnston, Kimberly Jones, Sekar Kathiresan, Alyna Khan, Wonji Kim, Greg Kinney, Holly Kramer, Christoph Lange, Ethan Lange, Leslie Lange, Cecelia Laurie, Meryl LeBoff, Jiwon Lee, Seunggeun Shawn Lee, Wen-Jane Lee, David Levine, Joshua Lewis, Xiaohui Li, Yun Li, Henry Lin, Honghuang Lin, Keng Han Lin, Simin Liu, Yongmei Liu, Yu Liu, James Luo, Michael Mahaney, Barry Make, JoAnn Manson, Lauren Margolin, Lisa Martin, Susan Mathai, Susanne May, Patrick McArdle, Merry-Lynn McDonald, Sean McFarland, Daniel McGoldrick, Caitlin McHugh, Hao Mei, Luisa Mestroni, Nancy Min, Ryan L. Minster, Matt Moll, Arden Moscati, Solomon Musani, Stanford Mwasongwe, Josyf C. Mychaleckyj, Girish Nadkarni, Rakhi Naik, Take Naseri, Sergei Nekhai, Bonnie Neltner, Heather Ochs-Balcom, David Paik, James Pankow, Afshin Parsa, Juan Manuel Peralta, Marco Perez, James Perry, Ulrike Peters, Lawrence S. Phillips, Toni Pollin, Julia Powers Becker, Meher Preethi Boorgula, Michael Preuss, Dandi Qiao, Zhaohui Qin, Nicholas Rafaels, Laura Raffield, Laura Rasmussen-Torvik, Aakrosh Ratan, Robert Reed, Elizabeth Regan, Muagututi‘a Sefuiva Reupena, Carolina Roselli, Pamela Russell, Sarah Ruuska, Kathleen Ryan, Ester Cerdeira Sabino, Danish Saleheen, Shabnam Salimi, Steven Salzberg, Kevin Sandow, Vijay G. Sankaran, Christopher Scheller, Ellen Schmidt, Karen Schwander, Frank Sciurba, Christine Seidman, Jonathan Seidman, Stephanie L. Sherman, Aniket Shetty, Wayne Hui-Heng Sheu, Brian Silver, Josh Smith, Tanja Smith, Sylvia Smoller, Beverly Snively, Michael Snyder, Tamar Sofer, Garrett Storm, Elizabeth Streeten, Yun Ju Sung, Jody Sylvia, Adam Szpiro, Carole Sztalryd, Hua Tang, Margaret Taub, Matthew Taylor, Simeon Taylor, Machiko Threlkeld, Lesley Tinker, David Tirschwell, Sarah Tishkoff, Hemant Tiwari, Catherine Tong, Michael Tsai, Dhananjay Vaidya, Peter VandeHaar, Tarik Walker, Robert Wallace, Avram Walts, Fei Fei Wang, Heming Wang, Karol Watson, Jennifer Wessel, Kayleen Williams, L. Keoki Williams, Carla Wilson, Joseph Wu, Huichun Xu, Lisa Yanek, Ivana Yang, Rongze Yang, Norann Zaghloul, Maryam Zekavat, Snow Xueyan Zhao, Wei Zhao, Degui Zhi, Xiang Zhou, Xiaofeng Zhu, George J. Papanicolaou, Deborah A. Nickerson, Sharon R. Browning, Michael C. Zody, Sebastian Zöllner, James G. Wilson, L. Adrienne Cupples, Cathy C. Laurie, Cashell E. Jaquish, Ryan D. Hernandez, Timothy D. O’Connor, Gonçalo R. Abecasis

**Affiliations:** 1 Department of Biostatistics, University of Michigan School of Public Health, Ann Arbor, MI USA; 2 Center for Statistical Genetics, University of Michigan School of Public Health, Ann Arbor, MI USA; 3 Institute for Genome Sciences, University of Maryland School of Medicine, Baltimore, MD USA; 4 Program in Personalized and Genomic Medicine, University of Maryland School of Medicine, Baltimore, MD USA; 5 Department of Medicine, University of Maryland School of Medicine, Baltimore, MD USA; 6 Department of Computational Medicine and Bioinformatics, University of Michigan, Ann Arbor, MI USA; 7 Department of Genome Sciences, University of Washington, Seattle, WA USA; 8 Department of Biology, Pennsylvania State University, University Park, PA USA; 9 Institute for Computational and Data Sciences, Pennsylvania State University, University Park, PA USA; 10 Biomedical Sciences Graduate Program, University of California, San Francisco, San Francisco, CA USA; 11 New York Genome Center, New York, NY USA; 12 Department of Biostatistics, University of Washington, Seattle, WA USA; 13 Department of Biostatistics, Boston University School of Public Health, Boston, MA USA; 14 Department of Medicine, Division of Medical Genetics, University of Washington, Seattle, WA USA; 15 USF Genomics, College of Public Health, University of South Florida, Tampa, FL USA; 16 Icahn School of Medicine at Mount Sinai, New York, NY USA; 17 The Broad Institute of MIT and Harvard, Cambridge, MA USA; 18 Massachusetts General Hospital, Boston, MA USA; 19 Department of Epidemiology, Rollins School of Public Health, Emory University, Atlanta, GA USA; 20 McKusick-Nathans Institute, Department of Genetic Medicine, Johns Hopkins University School of Medicine, Baltimore, MD USA; 21 University of Alabama, Birmingham, AL USA; 22 Zilber School of Public Health, University of Wisconsin Milwaukee, Milwaukee, WI USA; 23 Cleveland Clinic, Cleveland, OH USA; 24 Department of Medicine, Columbia University Medical Center, New York, NY USA; 25 Department of Epidemiology, Columbia University Medical Center, New York, NY USA; 26 The Emmes Corporation, Rockville, MD USA; 27 Johns Hopkins University, Baltimore, MD USA; 28 National Heart, Lung, and Blood Institute, National Institutes of Health, Bethesda, MD USA; 29 Department of Medicine, Boston University School of Medicine, Boston, MA USA; 30 Department of Epidemiology, Boston University School of Public Health, Boston, MA USA; 31 Framingham Heart Study, Framingham, MA USA; 32 Department of Epidemiology, University of Michigan School of Public Health, Ann Arbor, MI USA; 33 Department of Human Genetics, University of Texas Rio Grande Valley School of Medicine, Brownsville, TX USA; 34 South Texas Diabetes and Obesity Institute, University of Texas Rio Grande Valley School of Medicine, Brownsville, TX USA; 35 Department of Biochemistry, Wake Forest School of Medicine, Winston-Salem, NC USA; 36 Department of Medicine, University of Washington, Seattle, WA USA; 37 Cardiovascular Health Research Unit, University of Washington, Seattle, WA USA; 38 Department of Bioengineering and Therapeutic Sciences, University of California, San Francisco, San Francisco, CA USA; 39 Department of Medicine, University of California, San Francisco, San Francisco, CA USA; 40 Department of Medicine, Harvard Medical School, Boston, MA USA; 41 Department of Medicine, Brigham and Women’s Hospital, Boston, MA USA; 42 Department of Pediatrics, Johns Hopkins University, Baltimore, MD USA; 43 Division of Pediatric Hematology, Johns Hopkins University, Baltimore, MD USA; 44 Department of Medical Genetics, University of British Columbia, Vancouver, British Columbia Canada; 45 Division of Preventive Medicine, Brigham and Women’s Hospital, Boston, MA USA; 46 Harvard Medical School, Boston, MA USA; 47 The Institute for Translational Genomics and Population Sciences, Department of Pediatrics, The Lundquist Institute for Biomedical Innovation, Harbor-UCLA Medical Center, Torrance, CA USA; 48 Channing Division of Network Medicine, Department of Medicine, Brigham and Women’s Hospital, Boston, MA USA; 49 Department of Cardiovascular Medicine, Heart & Vascular Institute, Cleveland Clinic, Cleveland, OH USA; 50 Department of Cardiovascular and Metabolic Sciences, Lerner Research Institute, Cleveland Clinic, Cleveland, OH USA; 51 Department of Molecular Medicine, Cleveland Clinic Lerner College of Medicine, Case Western Reserve University, Cleveland, OH USA; 52 Metabolomics Platform, The Broad Institute of MIT and Harvard, Cambridge, MA USA; 53 Department of Medicine, University of Mississippi Medical Center, Jackson, MS USA; 54 Department of Pediatrics, University of Mississippi Medical Center, Jackson, MS USA; 55 Department of Population Health Science, University of Mississippi Medical Center, Jackson, MS USA; 56 Vitalant Research Institute, San Francisco, CA USA; 57 Department of Laboratory Medicine, University of California, San Francisco, San Francisco, CA USA; 58 Department of Medicine, University of Illinois at Chicago, Chicago, IL USA; 59 Division of Biomedical Informatics and Personalized Medicine, Department of Medicine, University of Colorado Anschutz Medical Campus, Aurora, CO USA; 60 Mayo Clinic, Rochester, MN USA; 61 McDonnell Genome Institute, Washington University, St Louis, MO USA; 62 Department of Genetics, Washington University, St Louis, MO USA; 63 Program in Medical and Population Genetics, The Broad Institute of MIT and Harvard, Cambridge, MA USA; 64 Molecular Cardiology Division, Victor Chang Cardiac Research Institute, Darlinghurst, New South Wales Australia; 65 Faculty of Medicine, University of New South Wales, Kensington, New South Wales Australia; 66 Cardiology Department, St Vincent’s Hospital, Darlinghurst, New South Wales Australia; 67 National Jewish Health, Center for Genes, Environment and Health, Denver, CO USA; 68 Institute of Genetic Epidemiology, Department of Genetics and Pharmacology, Medical University of Innsbruck, Innsbruck, Austria; 69 Institute of Molecular Medicine, University of Texas Health Science Center at Houston, Houston, TX USA; 70 Department of Epidemiology, University of North Carolina, Chapel Hill, NC USA; 71 Institute for Biomedicine, Eurac Research, Bolzano, Italy; 72 Department of Bioethics & Humanities, University of Washington School of Medicine, Seattle, WA USA; 73 Pittsburgh Heart, Lung, Blood and Vascular Medicine Institute, University of Pittsburgh, Pittsburgh, PA USA; 74 Pulmonary, Allergy and Critical Care Medicine, University of Pittsburgh, Pittsburgh, PA USA; 75 Department of Medicine, University of Pittsburgh, Pittsburgh, PA USA; 76 VA Boston Healthcare System, Boston, MA USA; 77 Division of Sleep and Circadian Disorders, Brigham and Women’s Hospital, Boston, MA USA; 78 Department of Epidemiology, Tulane University, New Orleans, LA USA; 79 Tulane University Translational Science Institute, Tulane University, New Orleans, LA USA; 80 Department of Neurology, Boston University School of Medicine, Boston, MA USA; 81 Department of Epidemiology, University of Washington, Seattle, WA USA; 82 Department of Epidemiology, University of Alabama at Birmingham, Birmingham, AL USA; 83 Bloodworks Northwest Research Institute, Seattle, WA USA; 84 Population Sciences Branch, National Heart, Lung, and Blood Institute, National Institutes of Health, Framingham, MA USA; 85 Albert Einstein College of Medicine, New York, NY USA; 86 Department of Epidemiology, Vitalant Research Institute, San Francisco, CA USA; 87 Department of Pediatrics, UCSF Benioff Children’s Hospital, Oakland, CA USA; 88 Division of Pediatric Hematology, UCSF Benioff Children’s Hospital, Oakland, CA USA; 89 Hinda and Arthur Marcus Institute for Aging Research, Hebrew SeniorLife, Boston, MA USA; 90 Department of Medicine, Beth Israel Deaconess Medical Center, Boston, MA USA; 91 Division of Public Health Sciences, Fred Hutchinson Cancer Research Center, Seattle, WA USA; 92 Department of Epidemiology, Johns Hopkins University, Baltimore, MD USA; 93 Institute of Genetic Epidemiology, Faculty of Medicine and Medical Center, University of Freiburg, Freiburg, Germany; 94 Department of Medicine, University of Colorado at Denver, Aurora, CO USA; 95 Brigham and Women’s Hospital, Boston, MA USA; 96 Biostatistics and Statistics, Harvard University, Boston, MA USA; 97 The Charles Bronfman Institute for Personalized Medicine, Icahn School of Medicine at Mount Sinai, New York, NY USA; 98 The Mindich Child Health and Development Institute, Icahn School of Medicine at Mount Sinai, New York, NY USA; 99 Department of Genes and Human Disease, Oklahoma Medical Research Foundation, Oklahoma City, OK USA; 100 Beth Israel Deaconess Medical Center, Boston, MA USA; 101 Center for Public Health Genomics, University of Virginia, Charlottesville, VA USA; 102 Department of Public Health Sciences, University of Virginia, Charlottesville, VA USA; 103 Clinical and Translational Epidemiology Unit, Mongan Institute, Massachusetts General Hospital, Boston, MA USA; 104 Metabolism Program, The Broad Institute of MIT and Harvard, Cambridge, MA USA; 105 Department of Medicine, Johns Hopkins University, Baltimore, MD USA; 106 Cardiovascular Medicine, University of Massachusetts Medical School, Worcester, MA USA; 107 International Health Institute, Brown University, Providence, RI USA; 108 Department of Epidemiology, Brown University, Providence, RI USA; 109 Department of Anthropology, Brown University, Providence, RI USA; 110 Division of General Internal Medicine, Massachusetts General Hospital, Harvard Medical School, The Broad Institute of MIT and Harvard, Boston, MA USA; 111 University of Arizona, Tucson, AZ USA; 112 Geriatrics Research and Education Clinical Center, Baltimore Veterans Administration Medical Center, Baltimore, MD USA; 113 Texas Cardiac Arrhythmia Institute, St David’s Medical Center, Austin, TX USA; 114 Department of Internal Medicine, Dell Medical School, Austin, TX USA; 115 Human Genetics Center, Department of Epidemiology, Human Genetics, and Environmental Sciences, School of Public Health, University of Texas Health Science Center at Houston, Houston, TX USA; 116 Cardiovascular Research Center, Massachusetts General Hospital, Boston, MA USA; 117 Center for Genomic Medicine, Massachusetts General Hospital, Boston, MA USA; 118 Department of Laboratory Medicine and Pathology, University of Minnesota, Minneapolis, MN USA; 119 Division of Cardiology, Department of Medicine, Johns Hopkins University, Baltimore, MD USA; 120 Department of Health Services, University of Washington, Seattle, WA USA; 121 Kaiser Permanente Washington Health Research Institute, Seattle, WA USA; 122 Division of Biostatistics, Washington University in St Louis, St Louis, MO USA; 123 Vanderbilt University Medical Center, Nashville, TN USA; 124 Department of Biostatistics, Johns Hopkins Bloomberg School of Public Health, Baltimore, MD USA; 125 University of Colorado at Denver, Denver, CO USA; 126 Precision Medicine Center, Seoul National University Bundang Hospital, Seongnam, Republic of Korea; 127 Macrogen Inc, Seoul, Republic of Korea; 128 Gong Wu Genomic Medicine Institute, Seoul National University Bundang Hospital, Seongnam, Republic of Korea; 129 Glenn Biggs Institute for Alzheimer’s and Neurodegenerative Diseases, University of Texas Health Sciences Center at San Antonio, San Antonio, TX USA; 130 Department of Pediatrics, Emory University School of Medicine, Atlanta, GA USA; 131 Aflac Cancer and Blood Disorders Center, Children’s Healthcare of Atlanta, Atlanta, GA USA; 132 Taichung Veterans General Hospital Taiwan, Taichung City, Taiwan; 133 Seattle Epidemiologic Research and Information Center, Department of Veterans Affairs Office of Research and Development, Seattle, WA USA; 134 Survey Research Center, Institute for Social Research, University of Michigan, Ann Arbor, MI USA; 135 Division of Epidemiology and Community Health, School of Public Health, University of Minnesota, Minneapolis, MN USA; 136 Duke University, Durham, NC USA; 137 Department of Pathology & Laboratory Medicine, University of Vermont Larner College of Medicine, Burlington, VT USA; 138 Center for Genetic Epidemiology, Department of Preventive Medicine, University of Southern California, Los Angeles, CA USA; 139 Illumina Laboratory Services, Illumina Inc, San Diego, CA USA; 140 Department of Psychology, University of Minnesota, Minneapolis, MN USA; 141 Department of Human Genetics, Graduate School of Public Health, University of Pittsburgh, Pittsburgh, PA USA; 142 Department of Biostatistics, Graduate School of Public Health, University of Pittsburgh, Pittsburgh, PA USA; 143 Department of Internal Medicine-Cardiology, University of Michigan, Ann Arbor, MI USA; 144 Department of Human Genetics, University of Michigan, Ann Arbor, MI USA; 145 Department of Epidemiology, University of Kentucky, Lexington, KY USA; 146 Duke Molecular Physiology Institute, Duke University Medical Center, Durham, NC USA; 147 University of Texas Health Science Center at Houston, Houston, TX USA; 148 Baylor College of Medicine Human Genome Sequencing Center, Houston, TX USA; 149 Northwest Genomics Center, Seattle, WA USA; 150 Brotman Baty Institute, Seattle, WA USA; 151 Department of Psychiatry, University of Michigan, Ann Arbor, MI USA; 152 Department of Physiology and Biophysics, University of Mississippi Medical Center, Jackson, MS USA; 153 Department of Human Genetics, McGill University, Montreal, Quebec Canada; 154 Quantitative Biosciences Institute, University of California, San Francisco, San Francisco, CA USA; 155 Institute for Human Genetics, University of California, San Francisco, San Francisco, CA USA; 156 Bakar Computational Health Sciences Institute, University of California, San Francisco, San Francisco, CA USA; 157 Children’s Hospital of Philadelphia, Philadelphia, PA USA; 158 University of Maryland, Baltimore, MD USA; 159 University of Washington, Seattle, WA USA; 160 University of Mississippi, Jackson, MS USA; 161 National Institutes of Health, Bethesda, MD USA; 162 Stanford University, Stanford, CA USA; 163 University of Pittsburgh, Pittsburgh, PA USA; 164 Fundação de Hematologia e Hemoterapia de Pernambuco–Hemope, Recife, Brazil; 165 National Jewish Health, Denver, CO USA; 166 Medical College of Wisconsin, Milwaukee, WI USA; 167 Washington State University, Seattle, WA USA; 168 University of California, Los Angeles, Los Angeles, CA USA; 169 Broad Institute, Cambridge, MA USA; 170 National Taiwan University, Taipei, Taiwan; 171 University of Virginia, Charlottesville, VA USA; 172 National Health Research Institute Taiwan, Zhunan Township, Taiwan; 173 University of Vermont, Burlington, VT USA; 174 University of Michigan, Ann Arbor, MI USA; 175 University of Chicago, Chicago, IL USA; 176 Washington University in St Louis, St Louis, MO USA; 177 Vanderbilt University, Nashville, TN USA; 178 University of Cincinnati, Cincinnati, OH USA; 179 University of North Carolina, Chapel Hill, NC USA; 180 University of Texas Rio Grande Valley School of Medicine, Edinburg, TX USA; 181 Brown University, Providence, RI USA; 182 Harvard University, Boston, MA USA; 183 University of Texas Health at Houston, Houston, TX USA; 184 Fred Hutchinson Cancer Research Center, Seattle, WA USA; 185 Indiana University, Indianapolis, IN USA; 186 Yale University, New Haven, CT USA; 187 University of Texas Rio Grande Valley School of Medicine, San Antonio, TX USA; 188 University of Colorado Anschutz Medical Campus, Aurora, CO USA; 189 Wake Forest Baptist Health, Winston-Salem, NC USA; 190 University of Iowa, Iowa City, IA USA; 191 Tri-Service General Hospital National Defense Medical Center, Taipei, Taiwan; 192 Blood Works Northwest, Seattle, WA USA; 193 Ohio State University Wexner Medical Center, Columbus, OH USA; 194 Emory University, Atlanta, GA USA; 195 Loyola University, Maywood, IL USA; 196 Harvard School of Public Health, Boston, MA USA; 197 Lundquist Institute, Torrance, CA USA; 198 Boston University, Boston, MA USA; 199 Stanford University, Palo Alto, CA USA; 200 University of Texas Rio Grande Valley School of Medicine, Brownsville, TX USA; 201 George Washington University, Washington, DC USA; 202 Harvard University, Cambridge, MA USA; 203 Ministry of Health, Government of Samoa, Apia, Samoa; 204 Howard University, Washington, DC USA; 205 University at Buffalo, Buffalo, NY USA; 206 University of Minnesota, Minneapolis, MN USA; 207 Northwestern University, Chicago, IL USA; 208 Lutia I Puava Ae Mapu I Fagalele, Apia, Samoa; 209 Universidade de Sao Paulo, Sao Paulo, Brazil; 210 Columbia University, New York, NY USA; 211 Broad Institute, Harvard University, Boston, MA USA; 212 UMass Memorial Medical Center, Worcester, MA USA; 213 University of Pennsylvania, Philadelphia, PA USA; 214 Henry Ford Health System, Detroit, MI USA; 215 Case Western Reserve University, Cleveland, OH USA

**Keywords:** Rare variants, Rare variants, Next-generation sequencing, Genetics research

## Abstract

The Trans-Omics for Precision Medicine (TOPMed) programme seeks to elucidate the genetic architecture and biology of heart, lung, blood and sleep disorders, with the ultimate goal of improving diagnosis, treatment and prevention of these diseases. The initial phases of the programme focused on whole-genome sequencing of individuals with rich phenotypic data and diverse backgrounds. Here we describe the TOPMed goals and design as well as the available resources and early insights obtained from the sequence data. The resources include a variant browser, a genotype imputation server, and genomic and phenotypic data that are available through dbGaP (Database of Genotypes and Phenotypes)^1^. In the first 53,831 TOPMed samples, we detected more than 400 million single-nucleotide and insertion or deletion variants after alignment with the reference genome. Additional previously undescribed variants were detected through assembly of unmapped reads and customized analysis in highly variable loci. Among the more than 400 million detected variants, 97% have frequencies of less than 1% and 46% are singletons that are present in only one individual (53% among unrelated individuals). These rare variants provide insights into mutational processes and recent human evolutionary history. The extensive catalogue of genetic variation in TOPMed studies provides unique opportunities for exploring the contributions of rare and noncoding sequence variants to phenotypic variation. Furthermore, combining TOPMed haplotypes with modern imputation methods improves the power and reach of genome-wide association studies to include variants down to a frequency of approximately 0.01%.

## Abstract (web summary)

The goals, resources and design of the NHLBI Trans-Omics for Precision Medicine (TOPMed) programme are described, and analyses of rare variants detected in the first 53,831 samples provide insights into mutational processes and recent human evolutionary history.

## Main

Advancing DNA-sequencing technologies and decreasing costs are enabling researchers to explore human genetic variation at an unprecedented scale^2,3^. For these advances to improve our understanding of human health, they must be deployed in well-phenotyped human samples and used to build resources such as variation catalogues^3,4^, control collections^5,6^ and imputation reference panels^7–9^. Here we describe high-coverage whole-genome sequencing (WGS) analyses of the first 53,831 TOPMed samples (Box 1 and Extended Data Tables 1, 2); additional data are being made available as quality control, variant calling and dbGaP curation are completed (altogether more than 130,000 TOPMed samples are now available in dbGaP).

**Extended Data Fig. 1:**
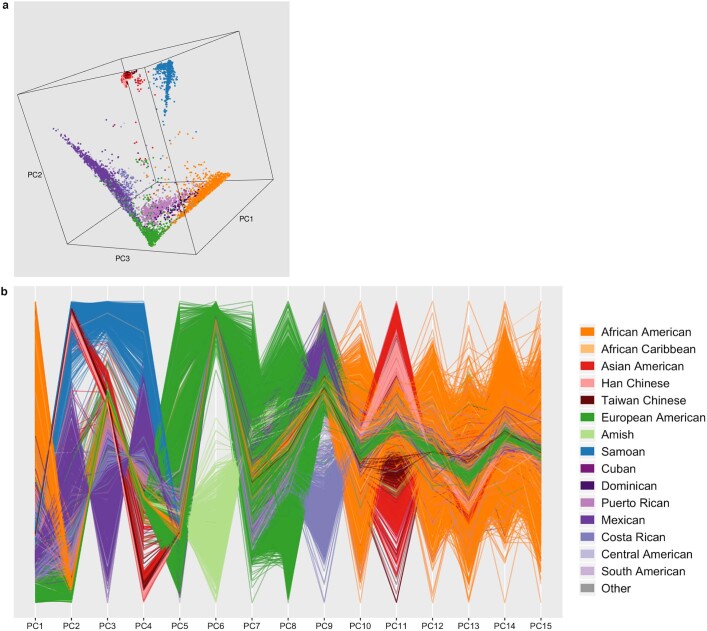
Principal components of the genotypic data from freeze 5 pooled across studies. **a**, Three-dimensional plot of principal components (PC) 1, 2 and 3. **b**, Parallel coordinate plot colour-coded by categories defined according to race, ancestry and/or ethnic information provided by the study participants and/or by study investigators according to study inclusion criteria. Individuals with missing values for ancestry or ethnicity are excluded.

A key goal of the TOPMed programme is to understand risk factors for heart, lung, blood and sleep disorders by adding WGS and other ‘omics’ data to existing studies with deep phenotyping (Supplementary Information 1.1 and Supplementary Fig. 1). The programme currently consists of more than 80 participating studies, around 1,000 investigators and more than 30 working groups (https://www.nhlbiwgs.org/working-groups-public). TOPMed participants are ethnically and ancestrally diverse (Extended Data Fig. 1, Supplementary Information 1.1.4 and Supplementary Fig. 2). Through a combination of race and ethnicity information (from participant questionnaires and/or study inclusion criteria), we classified study participants into ‘population groups’, which varied in composition according to the goals of each analysis. In some analyses, these groups were further refined using genetic ancestry (see Methods and Supplementary Information for details).

Our study extends previous efforts by identifying and characterizing the rare variants that comprise the majority of human genomic variation^7,10–12^. Rare variants represent recent and potentially deleterious changes that can affect protein function, gene expression or other biologically important elements^11,13,14^.

Box 1 TOPMed participant consents and data access The TOPMed programme comprises more than 80 participating studies, of which 32 are represented in the 53,831 whole genomes described here. TOPMed has leveraged existing studies with deep phenotyping and longitudinal follow-up data and with varied informed consent procedures and options. Consent groups range from broad ‘general research use’ and ‘health, medical and biomedical’ categories to disease-specific categories for heart, lung, blood and/or sleep disorders. Many studies have further consent modifiers, such as limiting use to not-for-profit organizations or requiring documentation of local IRB approval. Participant consents guide the appropriate use of data by TOPMed investigators as well; therefore, the set of study-consent groups used varies across different analyses reported in this paper (Extended Data Table 3).

TOPMed data have been deposited in dbGaP and access is adjudicated by a staff committee of the National Institutes of Health. The committee verifies that applications are consistent with data use limitations and consent groups for each sample. Study investigators have no role in the decision, except in a small subset of studies that require a letter of collaboration. A summary of currently available data and any use restrictions is available at https://www.ncbi.nlm.nih.gov/gap/advanced_search/?TERM=topmed.

Although TOPMed studies have separate dbGaP accessions, formats are standardized to facilitate combining data, with all variants from the joint genotype call set included in the variant call format (VCF) files, unique sample identifiers across all of TOPMed and sample attributes with TOPMed-specific variables. Notably, cross-study analyses require the identification of a set of compatible study-consent groups. In addition to genotype calls, CRAM files with aligned sequence reads are also available, hosted in commercial clouds and with access managed by dbGaP. The dbGaP accession numbers for all TOPMed studies referenced in this paper are listed in Extended Data Tables 2, 3.

The TOPMed imputation reference panel is available to users for imputation into their own samples via an imputation server. The server performs imputation into these samples, while the reference panel data themselves are not exposed to the user because they derive from multiple studies with variable consent types and other data use limitations (Extended Data Table 3).

## TOPMed WGS quality assessment

WGS of the TOPMed samples was performed over multiple studies, years and sequencing centres. To minimize batch effects, we standardized laboratory methods, mapped and processed sequence data centrally using a single pipeline, and performed variant calling and genotyping jointly across all samples (see Methods). We annotated each variant site with multiple sequence quality metrics and trained machine learning filters to identify and exclude inconsistencies that are revealed when the same individual was sequenced repeatedly. Available WGS data were processed periodically to produce genotype data ‘freezes’. The 53,831 samples described here are drawn from TOPMed freeze 5.

Stringent variant and sample quality filters were applied and the resulting genotype call sets were evaluated in several ways (Supplementary Information 1.2.2, 1.3, 1.4). First, we compared genotypes for samples sequenced in duplicate (the mean alternative allele concordance was 0.9995 for single-nucleotide variants (SNVs) and 0.9930 for insertions or deletions (indels)). Second, we compared genotypes to those from previous whole-exome sequencing datasets (protein-coding regions from GENCODE^15^; 80% of variants were found with both approaches and overlapping variant calls had a concordance of 0.9993 for SNVs and 0.9974 for indels) (Supplementary Tables 1–3). Third, we compared genotypes to those obtained using alternative informatics tools (compared to GATK v.4.1.3, TOPMed has lower Mendelian inconsistency rates and minimizes batch effects) (Supplementary Table 4). These reproducibility estimates indicate the high quality of the genotype calls and effectiveness of machine-learning-based quality filters.

Batch effects were evaluated by (1) comparing distributions of genetic principal components among sequencing centres, which are very similar between European American and African American individuals (Supplementary Figs. 3–5); (2) comparing alternative allele concordance between duplicates among centres, which is high (the largest difference being 4 × 10^−4^), and the patterns of between- versus within-centre differences, which indicate random errors rather than systematic centre differences (Supplementary Figs. 6–8); and (3) performing tests of association between variants and batches, which show a very small fraction of variants with genome-wide significance (0.004%, Supplementary Figs. 9, 10) (Supplementary Information 1.2). We conclude that batch effects appear to be minor, thus enabling multi-study association testing.

## 410 million genetic variants in 53,831 samples

A total of 7.0 × 10^15^ bases of DNA-sequencing data were generated, consisting of an average of 129.6 × 10^9^ bases of sequence distributed across 864.2 million paired reads (each 100–151 base pairs (bp) long) per individual. For a typical individual, 99.65% of the bases in the reference genome were covered, to a mean read depth of 38.2×.

Sequence analysis identified 410,323,831 genetic variants (381,343,078 SNVs and 28,980,753 indels), corresponding to an average of one variant per 7 bp (Extended Data Table 4). Overall, 78.7% of these variants had not been described in dbSNP build 149; TOPMed variants now account for the majority of variants in dbSNP. Among all variant alleles, 46.0% were singletons, observed once across all 53,831 participants. Among 40,722 unrelated participants (see Methods), the proportion of singleton variants was higher at 53.1% (Table 1). Downsampling analyses show that the proportion of singletons increases until around 15,000 unrelated individuals are sequenced and then decreases very gradually (Supplementary Fig. 11). The fraction of singletons in each region or class of sites closely tracks functional constraints. For example, among all 4,651,453 protein-coding variants in unrelated individuals, the proportion of singletons was the highest for the 104,704 frameshift variants (68.4%), high among the 97,217 putative splice and truncation variants (62.1%), intermediate among the 2,965,093 nonsynonymous variants (55.6%) and lowest among the 1,435,058 synonymous variants (49.8%). Beyond protein-coding sequences, we found increased proportions of singletons in promoters (55.0%), 5′ untranslated regions (54.7%), regions of open chromatin (53.4%) and 3′ untranslated regions (53.3%); we found lower proportions of singletons in intergenic regions (53.0%) (Supplementary Table 5). Although putative transcription factor binding sites initially appeared to show fewer singletons (52.7%) than the remainder of the genome (53.1%), this pattern did not hold when we analysed highly mutable CpG sites separately. In fact, transcription factor binding sites were enriched for singletons in both CpG sites and non-CpG sites, an example of Simpson’s paradox^16^.

**Table 1:** Number of variants in 40,722 unrelated individuals in TOPMed

	All unrelated individuals (*n* = 40,722)		Per individual			
	Total	Singletons (%)	Average	5th percentile	Median	95th percentile
**Total variants**	**384,127,954**	**203,994,740 (53)**	**3,748,599**	**3,516,166**	**3,563,978**	**4,359,661**
**SNVs**	357,043,141	189,429,596 (53)	3,553,423	3,335,442	3,380,462	4,125,740
**Indels**	27,084,813	14,565,144 (54)	195,176	180,616	183,503	233,928
**Novel variants**	**298,373,330**	**191,557,469 (64)**	**29,202**	**20,312**	**24,106**	**44,336**
**SNVs**	275,141,134	177,410,620 (64)	25,027	17,520	20,975	36,861
**Indels**	23,232,196	14,146,849 (61)	4,175	2,747	3,145	7,359
**Coding variation**	**4,651,453**	**2,523,257 (54)**	**23,909**	**22,158**	**22,557**	**27,716**
**Synonymous**	1,435,058	715,254 (50)	11,651	10,841	11,056	13,678
**Nonsynonymous**	2,965,093	1,648,672 (56)	11,384	10,632	10,856	13,221
**Stop/essential splice**	97,217	60,347 (62)	474	425	454	566
**Frameshift**	104,704	71,577 (68)	132	112	127	165
**In-frame**	51,997	29,110 (56)	102	85	99	128

Novel variants are taken as variants that were not present in dbSNP build 149, the most recent dbSNP version without TOPMed submissions.

We identified an average of 3.78 million variants in each genome. Among these, an average of 30,207 (0.8%) were novel and 3,510 (0.1%) were singletons. Among all variants, we observed 3.17 million nonsynonymous and 1.53 million synonymous variants (a 2.1:1 ratio), but individual genomes contained similar numbers of nonsynonymous and synonymous variants (11,743 nonsynonymous and 11,768 synonymous, on average) (Extended Data Table 4). The difference can be explained if more than half of the nonsynonymous variants are removed from the population by natural selection before they become common.

## Putative loss-of-function variants

A notable class of variants is the 228,966 putative loss-of-function (pLOF) variants that we observed in 18,493 (95.0%) GENCODE^15^ genes (Extended Data Table 5 and Supplementary Fig. 12). This class includes the highest proportion of singletons among all of the variant classes that we examined. An average individual carried 2.5 unique pLOF variants. We identified more pLOF variants per individual than in previous surveys based on exome sequencing—an increase that was mainly driven by the identification of additional frameshift variants (Supplementary Table 6) and by a more uniform and complete coverage of protein-coding regions (Supplementary Figs. 13, 14).

We searched for gene sets with fewer rare pLOF variants than expected based on gene size. The gene sets with strong functional constraint included genes that encode DNA- and RNA-binding proteins, spliceosomal complexes, translation initiation machinery and RNA splicing and processing proteins (Supplementary Table 7). Genes associated with human disease in COSMIC^17^ (31% depletion), the GWAS catalogue^18^ (around 8% depletion), OMIM^19^ (4% depletion) and ClinVar^20^ (4% depletion) all contained fewer rare pLOF variants than expected (each comparison *P* < 10^−4^).

## The distribution of genetic variation

**Fig. 1:**
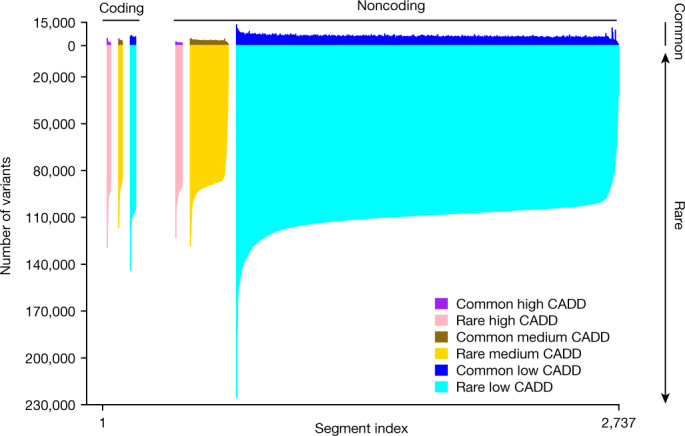
Distribution of genetic variants across the genome. Common (allele frequency ≥ 0.5%) and rare (allele frequency < 0.5%) variant counts are shown above and below the *x* axis, respectively, within 1-Mb concatenated segments (see Methods). Segments are stratified by CADD functionality score, and sorted based on their number of rare variants according to the functionality category. There were 22 high CADD, 22 medium CADD and 34 low CADD coding segments, and 40 high CADD, 238 medium CADD and 2,381 low CADD noncoding segments. Noncoding regions of the genome with low CADD scores (<10, reflecting lower predicted function) have the largest levels of common and rare variation (noncoding plot region, dark and light blue, respectively), followed by low CADD coding regions (coding plot region, dark and light blue, respectively). Overall, the vast majority of human genomic variation comprises rare variation.

**Extended Data Fig. 2:**
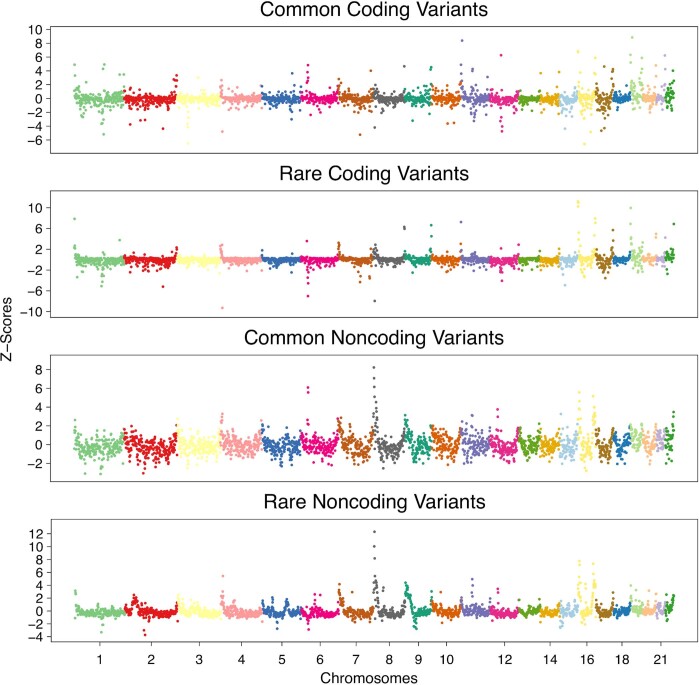
Distribution of genetic variants across the genome. After filtering to focus on regions of the genome that are accessible through short-read sequencing, most contiguous 1-Mb segments show similar levels of common (5,141 ± 1,298 variants with MAF ≥ 0.5%) and rare variation (120,414 ± 19,862 variants with MAF < 0.5%). From top to bottom, panel 1 shows the levels of variation across the genome for common coding variants, panel 2 for rare coding variants, panel 3 for common noncoding variants and panel 4 for rare noncoding variants. Variation levels are represented by the *Z*-score (*X*-mean/s.d.) of the adjusted variant counts per 1-Mb contiguous segment for each variant category.

We examined the distribution of variant sites across the genome by counting variants across ordered 1-megabase (Mb) concatenations of contiguous sequence with a similar conservation level (indicated by combined annotation-dependent depletion (CADD score^21^), and in segments categorized by coding versus noncoding status (Fig. 1 and Extended Data Fig. 2). As expected, the vast majority of human genomic variation is rare (minor allele frequency (MAF) < 0.5%)^10,11^ and located in putatively neutral, noncoding regions of the genome (Fig. 1). Although coding regions have lower average levels of both common (MAF ≥ 0.5%) and rare variation, we identified some ultra-conserved noncoding regions with even lower levels of genetic variation^22^ (Fig. 1 and Supplementary Fig. 15).

Segments with notably high or low levels of variation do exist. For example, one region on chromosome 8p (GRC 38 positions 1,000,001–7,000,000 bp) has the highest overall levels of variation (Extended Data Fig. 2). This is consistent with previous findings, as this region has been shown to have one of the highest mutation rates across the human genome^23^.

Although levels of common and rare variation within segments are significantly correlated (*R*^2^ = 0.462, *P* ≤ 2 × 10^−16^) (Supplementary Fig. 16), there are outliers. For example, segments overlapping the major histocompatibility complex (MHC) have the highest levels of common variation but no notable increase in levels of rare variation, consistent with balancing selection^24–26^. A detailed examination of the MHC shows peaks of increased variation and nucleotide diversity consistent with assembly-based analyses of the region^27^ (Supplementary Fig. 17). Segments with a high proportion of coding bases feature a strong depletion in the number of common variants but only a modest depletion in rare variants (Supplementary Fig. 18).

## Insights into mutation processes

A hallmark of human genetic variation is that SNVs tend to cluster together throughout the genome^3,28^. Such patterns of clustering contain important information about demographic history^29^, signals of natural selection^30^ and processes that generate mutations^31^. To dissect the spatial clustering of SNVs, we analysed a collection of 50,264,223 singleton SNVs ascertained in a subset of 3,000 unrelated individuals selected to have low levels of genetically estimated admixture—1,000 each of African, East Asian and European ancestry^32^ (see Methods).

**Fig. 2:**
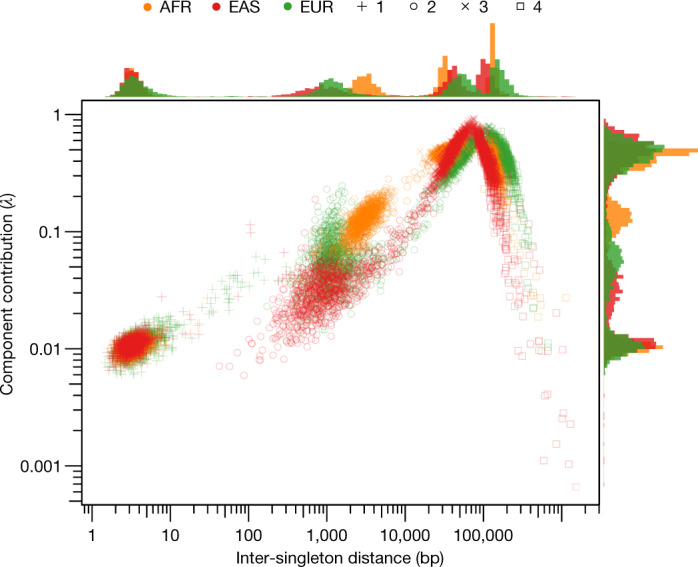
Characteristics of singleton clustering patterns. Parameter estimates for exponential mixture models of singleton density. Each point represents one of the four components in one of the 3,000 individuals in the sample, coloured according to the genetically inferred population of that individual. The rate parameters of each component are shown across the *x* axis, and the lambda parameters (that is, the proportion that the component contributes to the mixture) are shown on the *y* axis (on a log–log scale). Histograms show the distribution of the lambda and rate parameters for each component. AFR, African ancestry; EAS, East Asian ancestry; EUR, European ancestry.

In these data, we observed that 1.9% of singletons in a given individual occur at distances of less than 100 bp apart^33,34^ (Supplementary Figs. 19, 20). In coalescent simulations (see Methods), only 0.16% of the simulated singletons within an individual were less than 100 bp apart (Supplementary Figs. 19, 20). Although demographic history contributes to singleton clustering (Supplementary Information 1.6), population genetic processes alone do not fully account for the observed clustering patterns, particularly for the most closely spaced singletons. To better understand the latent factors that contribute to the observed clustering, we modelled the inter-singleton distance distribution as a mixture of exponential processes (see Methods). The best-fitting version of this model consisted of four mixture components (Fig. 2).

**Extended Data Fig. 3:**
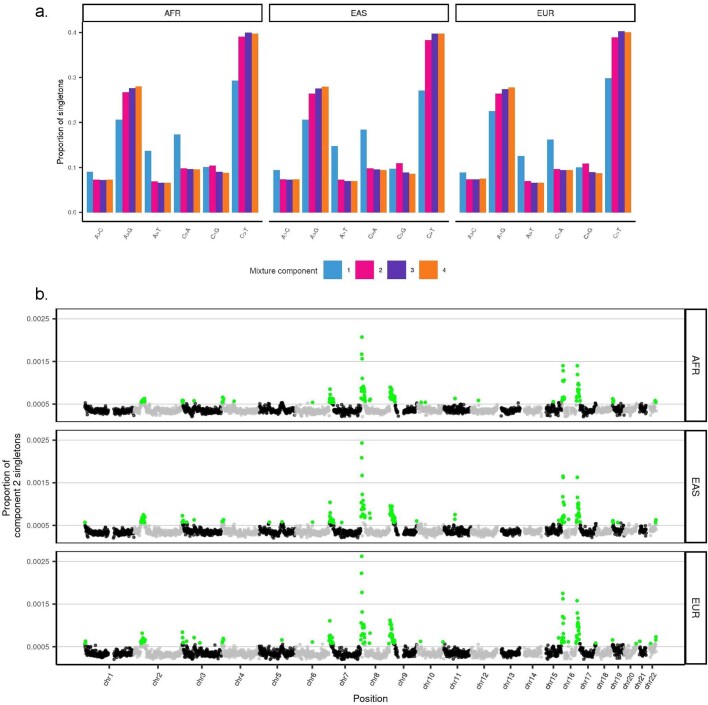
Characteristics of singleton clustering patterns. **a**, Mutational spectra of singletons assigned to each of the four mixture components, separated by population. **b**, Density of mixture component 2 singletons in 1-Mb windows across the genome. Windows with mixture component 2 singleton counts above the 95th percentile (calculated genome-wide per population subsample) are classified as hotspots and are highlighted in green.

Component 1 represents singletons that occurred an average of around 2–8 bp apart and accounted for approximately 1.5% of singletons in each sample. These singletons are substantially enriched for A>T and C>A transversions (Extended Data Fig. 3a), consistent with the signatures of trans-lesion synthesis that causes multiple non-independent point mutations within very short spans^35^. The density of component 1 singletons is also associated with CpG island density (Supplementary Fig. 21). Component 2 represents singletons occurring 500–5,000 bp apart, accounting for around 12–24% of singletons. These singletons are enriched for C>G transversions and show prominent subtelomeric concentrations on chromosomes 8p, 9p, 16p and 16q^36,37^ (Extended Data Fig. 3 and Supplementary Fig. 22), consistent with the recently described maternally derived C>G mutation clusters^36,37^. The exact mechanism that underlies this distinctive clustering pattern is unknown, but may involve either hypermutability of single-stranded DNA intermediates during the repair of double-stranded breaks^36,37^ or transcription-associated mutagenesis, with increased damage on the non-transcribed strand^38^. Our results are compatible with both these mechanisms: component 2 singletons are enriched near regions of H3K4 trimethylation, a mark associated with double-stranded break response^39^, and depleted in exon-dense regions (Supplementary Fig. 21). Component 3 singletons (occurring approximately 30–50 kilobases (kb) apart) accounted for around 43–49% of all singletons, and component 4 singletons (occurring approximately 125–170 kb apart) accounted for around 31–37% of all singletons. These latter components have nearly identical mutational spectra (Extended Data Fig. 3a) and are distributed about uniformly in the genome.

## Beyond SNVs and indels

To evaluate the potential of our data to generate even more comprehensive variation datasets, we developed and applied a method based on de novo assembly of unmapped and mismapped read pairs, enabling us to assemble sequences that are present in a sample but absent, or improperly represented, in the reference. As the majority of non-reference human sequence is present in the assembled genomes of other primates^40,41^, we leveraged available hominid references (see Methods) to specifically discover retained ancestral sequences that have been deleted in some human lineages, including on the reference haplotype.

**Fig. 3:**
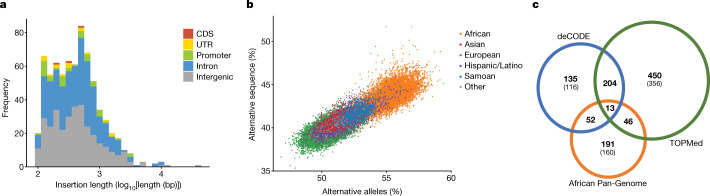
Retained non-reference ancestral sequences discovered from unmapped reads. **a**, Length distribution of fully resolved ancestral sequences, coloured by overlap with GENCODE v.29 genic features. **b**, Percentage of non-reference (alternative) alleles compared with the percentage of non-reference sequence identified per individual, coloured by population group. **c**, Venn diagram showing the positional concordance with insertions identified using short-read data from two previous studies^40,41^. The number of sequences specific to each study and that have not been partially resolved in the other studies is given between brackets.

In total, we placed 1,017 ancestral sequences, of which we were able to fully resolve 713, ranging in length from 100 bp to 39 kb (N50 = 1,183), and accounting for a total of 528,233 bp (Fig. 3a). We partially resolved 304 events, for which we assembled part of the ancestral sequence but could place only one breakpoint on the reference sequence (see Supplementary Information 1.7). Out of all 1,017 events, 551 (54.18%) occur within GENCODE v.29^15^ genes (a proportion that is not significantly different from 54.80% of the current reference genome GRCh38 that is within genes). The assembled sequences contain repetitive motifs at a significantly higher rate than the genome as a whole (58.2% versus 50.1%) (Supplementary Tables 8–10). There is a strong overrepresentation of simple and low complexity sequences both in the reference breakpoints and within the bodies of the non-reference sequences, which could be indicative of the instability of these motifs and/or errors in the reference.

Considering only fully resolved events with genotyping rates above 95% (*n* = 541), we identified between 232 kb and 418 kb of retained ancestral sequence per diploid individual. Allele frequencies of assembled retained sequences are greater than those observed for SNVs and indels, with 76.7% of the assembled sequences present at allele frequency of more than 5% and only 12% of assembled sequences with allele frequency of less than 0.5% (Supplementary Fig. 23). This could reflect difficulty in assembling rare haplotypes. Consistent with observations for SNVs and indels, individuals of African ancestry had, on average, more non-reference alleles (Fig. 3b, Supplementary Fig. 24 and Supplementary Table 11). The overwhelming majority of assembled events are shared by multiple continental groups. We found 58 genic (5 of which are exonic) and 48 intergenic sequences present in a homozygous state in all individuals in the cohort, suggesting that the reference sequence may be incomplete at particular loci, directly affecting the annotation of common forms of genes, such as *UBE2QL1*, *FOXO6* and *FURIN* (Supplementary Fig. 25).

Comparing our findings to two previous short-read studies on different smaller datasets^40,41^, 356 sequences (251 kb) are unique to our call set. Additionally, we resolved the length and both breakpoints for 94 events (104 kb) for which only one breakpoint had been reported (Fig. 3c). Further investigation of the overlap with insertions called using long reads on 15 genomes^42^, showed that—with a single exception—all previously described events with an allele frequency of more than 12% could be confirmed (Supplementary Fig. 26).

## Variation in *CYP2D6*

A complementary approach to de novo genome assembly is to develop approaches that combine multiple types of information—including previously observed haplotype variation, SNVs, indels, copy number and homology information—to identify and classify haplotypes in interesting regions of the genome. One such region is around the *CYP2D6* gene, which encodes an enzyme that metabolizes approximately 25% of prescription drugs and the activity of which varies substantially among individuals^43–45^. More than 150 *CYP2D6* haplotypes have been described, some involving a gene conversion with its nearby non-functional but highly similar paralogue *CYP2D7*.

We performed *CYP2D6* haplotype analysis for all 53,831 TOPMed individuals^43,46^. We called a total of 99 alleles (66 known and 33 novel) representing increased function, decreased function and loss of function (Supplementary Table 12). Nineteen of the known alleles and all of the novel alleles are defined by structural variants, including complex *CYP2D6*-*CYP2D7* hybrids and extensive copy number variation, which ranged from zero to eight gene copies (Supplementary Figs. 27, 28).

## Heterozygosity and rare variant sharing

**Fig. 4:**
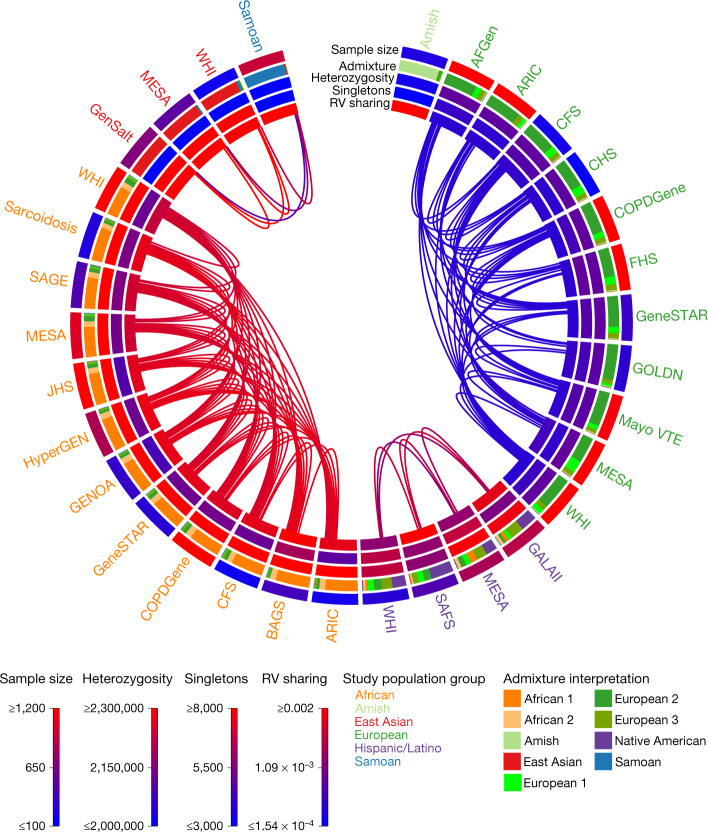
Ancestry, genetic diversity and rare-variant genetic relatedness across the TOPMed studies. Each study label is shaded based on their population group. From the outside moving in each track represents: the unrelated sample size of each study used in these calculations, average admixture values, average number of heterozygous sites in each individual’s genome, average number of singleton variants in each individual’s genome and the average within-study rare-variant (RV) sharing comparisons. The links depict the 75th percentile of between-study rare-variant sharing comparisons. All between-study rare-variant sharing comparisons can be found in Supplementary Fig. 29. The sample size, average heterozygosity, number of singletons, within-cohort rare-variant sharing and admixture values by TOPMed study and population group can be found in Supplementary Table 13. Study name abbreviations are defined in Extended Data Tables 1, 2 and Supplementary Table 20.

The TOPMed variation data also present an opportunity to examine expectations about rare variation, and to specifically investigate which studies show distinct patterns of variation that might be expected to provide unique insights. To do this, we grouped TOPMed participants by study and by population group, and calculated genetically determined ancestry components, heterozygosity, number of singletons and rare variant sharing (Fig. 4, Supplementary Table 13 and Supplementary Data 1).

As expected, African American and Caribbean population groups have the greatest heterozygosity^7,47^, followed by Hispanic/Latino, European American, Amish, East Asian and Samoan groups. This is consistent with a gradual loss of heterozygosity tracking the recent African origin of modern humans and subsequent migration from Africa to the rest of the globe^47,48^. The Asian population groups have among the lowest heterozygosity in our sample (even lower than the Amish, a European ancestry founder population with notably low heterozygosity^49,50^), but also the greatest singleton counts (in contrast to the Amish, who have the lowest; see Supplementary Information 1.8).

Using rare variation, we are also able to distinguish fine-scale patterns of population structure (Fig. 4, Supplementary Fig. 29 and Supplementary Information 1.9). Broadly, we observe sharing between population groups with shared continental ancestry (whether African, European, Asian or American). Nevertheless, additional patterns emerge. The Amish are unique among the included studies: they exhibit little rare variant sharing with outside groups and also the greatest rare variant sharing within the study—consistent with a marked founder effect. Furthermore, we observe an approximately 4× greater rare variant sharing between African American and Caribbean population groups than between European American population groups, even after correcting for sample size differences (Supplementary Fig. 30).

## Haplotype sharing

**Extended Data Fig. 4:**
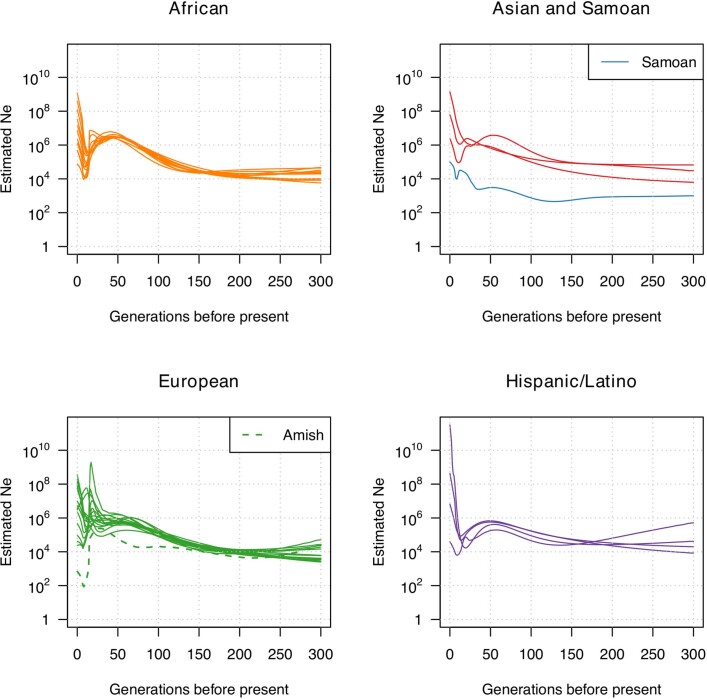
Estimates of recent effective population size by population group. Each line represents the estimate from a single study, considering only individuals with an annotated population group. The included studies are the same as those in Supplementary Fig. 31. The Amish and Samoan results are individually identified due to their distinct recent population size trajectories. *N*_e_, effective population size. The overlay view is shown in Supplementary Fig. 33.

A corollary to rare variant sharing is rare haplotype sharing through segments inherited from a recent common ancestor (Supplementary Figs. 31, 32). The distribution of identical-by-descent segments enables estimates of effective population sizes over the past 300 generations (Extended Data Fig. 4 and Supplementary Fig. 33). The Amish study shows the greatest average levels of within-study identical-by-descent sharing, consistent with a founder event 14 generations ago^50,51^. The demographic histories are broadly similar between population groups, with the exception of the Amish, who experienced a more extreme bottleneck when moving from Europe to America, and Samoan individuals, who have had a smaller effective population size than the East Asian populations from which they separated around 5,000 years ago^52–54^. Both non-Amish European ancestry and African ancestry populations appear to have experienced a bottleneck around 5–10 generations ago, consistent with moving to America, whether through colonization or forced migration (82% of TOPMed participants are US residents).

## Large samples alleviate the effects of linkage

**Fig. 5:**
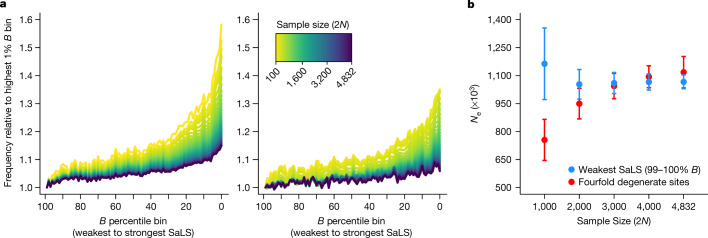
Relative increase in singletons and doubletons of the site frequency spectrum across McVicker’s *B* and the population size inferred from demographic inference using various sample sizes. **a**, The relative increase in the singleton (left) and doubleton (right) bins of the site frequency spectrum for decreasing percentile bins of McVicker’s *B* compared with the highest percentile bin of *B*. The higher percentiles of *B* indicate weaker effects of selection at linked sites (SaLS). These relative increases are plotted for different sample sizes. **b**, Each point corresponds to the population size inferred in the last generation of an exponential growth model for Europeans. Demographic inference was conducted with different sample sizes for fourfold degenerate sites (*n* = 4,718,653 sites) and the highest 1% *B* sites (*n* = 10,977,437 sites). Error bars show 95% confidence intervals (see Supplementary Table 14 for parameter values). *N*_e_, effective population size.

The relative numbers of singletons, doubletons and other very rare variants can be used to infer human demographic history^11,55,56^. Although much of demographic inference in past studies focused on fourfold degenerate synonymous sites in protein sequences, these sites evolve under the influence of strong selection at nearby protein-coding sites^57,58^, which can affect the inferred timing and magnitude of population size changes^59^. WGS enables us to access intergenic regions of the genome that are minimally affected by selection. We measured how the site frequency spectrum and demographic inference changed as a function of sample size and an index of selection at linked sites (McVicker’s *B* statistic^60^) using TOPMed individuals whose genomes suggested mostly European ancestry and low admixture. Estimates of effective population size of European individuals based on the 1% of the genome with the weakest effect of selection at linked sites consistently yielded around 1.1 million individuals (Fig. 5, Supplementary Figs. 34, 35 and Supplementary Table 14).

## Human adaptations

When adaptive mutations arise, they can quickly spread. This process generates distinct genomic patterns surrounding the locus, including extended regions of low-diversity haplotypes and few singletons. We scanned for evidence of very recent ongoing positive selection by taking advantage of our WGS data and large samples. We used the singleton density score^61^ to search for regions where positive selection has occurred or is ongoing in three ancestry groups: European (*n* = 21,196), African (*n* = 2,117) and East Asian (*n* = 1,355). Broadly, each of these populations showed evidence for adaptation in immune system genes, albeit with a variety of different gene targets, which probably reflects historical differences in pathogen exposure.

The European population shows selection signals (Supplementary Fig. 36a) in the vicinity of *LCT* and the MHC locus, reflecting well-known signals for adaptation to lactose metabolism and immune system function^61^. We further identify a strong selection signal implicating *HERC2*, a gene that is associated with iris pigmentation^62^. The African population shows a selection signal (Supplementary Fig. 36b) at a locus situated among a cluster of antimicrobial alpha- and beta-defensin genes^63^, which has an important role in innate immunity, suggesting a possible adaptive response to environmental pathogens. Other regions implicated include a locus 23 kb upstream of *NRG3*, a previously identified putative target of selection expressed in neural tissue^64,65^ and the calcium sensor *STIM1*. Mutations in *STIM1* are known to cause immunodeficiency^66^. The East Asian population shows a selection signal (Supplementary Fig. 36c) at *GJA5*, a gap junction protein that forms intercellular channels to allow transport between cells, and at *PRAG1*, a pseudokinase that interacts with cytoplasmic tyrosine kinase (*CSK*), which ultimately affects antibacterial immune response^67^. Combined with a strong signal at the MHC locus, this once again suggests adaptation in immune system function. We also find evidence of positive selection at two alcohol metabolism genes at mutations known to confer protection against alcoholism: the R48H polymorphism (rs1229984) in *ADH1B*^68,69^ and the E504K polymorphism (rs671) in *ALDH2*^70,71^.

## The TOPMed imputation resource

In addition to enabling detailed analysis of TOPMed sequenced samples, TOPMed can enhance the analysis of any genotyped samples^72^. To this end, we constructed a TOPMed-based imputation reference panel that now includes 97,256 individuals (Extended Data Table 3), including 308,107,085 SNVs and indels (Supplementary Table 15). This is, to our knowledge, the first imputation reference panel that is based exclusively on deep WGS data in diverse samples and greatly exceeds previously published alternatives^7,8^. For example, the average imputation quality *r*^2^ for variants with a frequency of 0.001 in genomes of individuals with an African ancestry increased from around 0.17 in previous panels to 0.96 (Supplementary Fig. 37). Similar improvements were observable in all ancestries that we considered except in South Asian individuals. The minimum allele frequency at which variants could be well-imputed (*r*^2^ > 0.3) decreased to around 0.002–0.003% (European or African ancestry in TOPMed). This means that 89% of the approximately 80,000 rare variants with MAF < 0.5% in an average genome of African ancestry can be recovered through genotype imputation using the TOPMed panel.

To illustrate the possibilities, we imputed TOPMed variants in array-genotyped participants of the UK Biobank^2^ and compared the results to exome-sequencing data of overlapping individuals. Of the 463,182 exome-sequencing variants with MAF > 0.05% in 49,819 participants of the UK Biobank, the majority (84.86%) were also present in the TOPMed-imputed data with imputation quality >0.3. This proportion was lower (52.97%) for 3,587,193 non-singleton exome-sequencing variants with MAF ≤ 0.05%. The TOPMed-imputed genotypes were highly correlated with the exome-sequencing genotypes—the average correlation ranged from 0.73 (MAF ≤ 0.05%) to 0.98 (MAF > 25%) (Supplementary Fig. 38).

An initial association analysis of 94,081 imputed rare autosomal (allele frequency ≤ 0.5%) pLOF variants identified, among other findings, several known rare variant associations with breast cancer: a frameshift variant in *CHEK2* and a stop gain variant in *PALB2* (see Methods and Supplementary Table 16). We also found that the burden of rare pLOF variants in *BRCA2* (comprising 35 rare pLOF variants; *P* = 1.6 × 10^−8^; cumulative allele frequency in cases versus controls, 0.13% versus 0.05%) was increased among cases. The individually associated pLOF variants would not have been detected using previous reference panels (Supplementary Table 16). Other examples of rare variant association signals included associations with the burden of rare pLOF variants in *USH2A* and retinal dystrophies (47 rare pLOF variants; allele frequency in cases versus controls, 3% versus 0.2%), *IFT140* and kidney cyst (18 rare pLOF variants; allele frequency in cases versus controls, 0.5% versus 0.1%), and *MYOC* and glaucoma (14 rare pLOF variants; allele frequency in cases versus controls, 0.5% versus 0.1%).

## Conclusion and future prospects

We show that TOPMed WGS data provide a rich resource for developing and testing methods for surveying human variation, for inference of human demography and for exploring functional constraints on the genome^73,74^. In addition to these uses, we expect that TOPMed data will improve nearly all ongoing studies of common and rare disorders by providing both a deep catalogue of variation in healthy individuals and an imputation resource that enables array-based studies to achieve a completeness that was previously attainable only through direct sequencing.

Members of the broader scientific community are using TOPMed resources through the WGS and phenotype data available on dbGaP, the BRAVO variant server and the imputation reference panel on the TOPMed imputation server. Full utilization of the programme’s resources by the scientific community will require new approaches for dealing with the large size of the omics data, the diversity of the phenotypic data types and structures, and the need to share data in a manner that supports the privacy and consent preferences of participants. These issues are currently being addressed in partnership with the NHLBI BioData Catalyst^75^ cloud-computing programme.

## Methods

### DNA samples

WGS for the 53,831 samples reported here was performed on samples that had previously been collected from and consented to by research participants from 33 NHLBI-funded research projects. All studies were approved by the corresponding institutional review boards (Supplementary Information 4). All sequencing was done from DNA extracted from whole blood, with the exception of 17 Framingham samples (lymphoblastoid cell lines) and HapMap samples NA12878 and NA19238 (lymphoblastoid cell lines) used periodically as sequencing controls. Cell lines were tested for mycoplasma contamination by aligning sequence data to the human genome, and authenticated by comparison with previous genetic analysis.

### WGS

WGS targeting a mean depth of at least 30× (paired-end, 150-bp reads) using Illumina HiSeq X Ten instruments was carried out over several years at six sequencing centres (Supplementary Table 17). All sequencing used PCR-free library preparation kits purchased from KAPA Biosystems, equivalent to the protocol in the Illumina TruSeq PCR-Free Sample Preparation Guide (Illumina, FC-121-2001). Centre-specific details are available from the TOPMed website (https://www.nhlbiwgs.org/topmed-whole-genome-sequencing-project-freeze-5b-phases-1-and-2). In addition, 30× coverage WGS for 1,606 samples from four contributing studies were sequenced before the start of the TOPMed sequencing project and are included in this dataset. These were sequenced at Illumina using HiSeq 2000 or 2500 instruments, have 2 × 100-bp or 2 × 125-bp paired-end reads and sometimes used PCR amplification.

### Sequence data processing and variant calling

Sequence data processing was performed periodically to produce genotype data ‘freezes’ that included all samples available at the time. All sequences were remapped using BWA-MEM^76^ to the hs38DH 1000 Genomes build 38 human genome reference including decoy sequences, following the protocol published previously^77^. Variant discovery and genotype calling was performed jointly, across TOPMed studies, for all samples in a given freeze using the GotCloud^78,79^ pipeline. This procedure results in a single, multi-study genotype call set. A support vector machine quality filter for variant sites was trained using a large set of site-specific quality metrics and known variants from arrays and the 1000 Genomes Project as positive controls and variants with Mendelian inconsistencies in multiple families as negative controls (see online documentation^80^ for more details). After removing all sites with a minor allele count less than 2, the genotypes with a minimal depth of more than 10× were phased using Eagle 2.4^81^. Sample-level quality control included checks for pedigree errors, discrepancies between self-reported and genetic sex, and concordance with previous genotyping array data. Any errors detected were addressed before dbGaP submission. Details regarding WGS data acquisition, processing and quality control vary among the TOPMed data freezes. Freeze-specific methods are described on the TOPMed website (https://www.nhlbiwgs.org/data-sets) and in documents included in each TOPMed accession released on dbGaP (for example, see document phd008024.1 in phs000956.v4.p1).

### Access to sequence data

Copies of individual-level sequence data for each study participant are stored on both Google and Amazon clouds. Access involves an approved dbGaP data access request and is mediated by the NCBI Sequence Data Delivery Pilot mechanism. This mechanism uses fusera software^82^ running on the user’s cloud instance to handle authentication and authorization with dbGaP. It provides read access to sequence data for one or more TOPMed (or other) samples as .cram files (with associated .crai index files) within a fuse virtual file system mounted on the cloud computing instance. Samples are identified by ‘SRR’ run accession numbers assigned in the NCBI Sequence Read Archive (SRA) database and shown under each study’s phs number in the SRA Run Selector (https://trace.ncbi.nlm.nih.gov/Traces/sra/sra.cgi). The phs numbers for all TOPMed studies are readily found by searching dbGaP for the string ‘TOPMed’. The fusera software is limited to running on Google or Amazon cloud instances to avoid incurring data egress charges. Fusera, samtools and other tools are also packaged in a Docker container for ease of use and are available for download from Docker Hub^83^.

### Sample sets

Several sample sets derived from three different WGS data freezes were used in the analyses presented here: freeze 3 (GRCh37 alignment, around 18,000 samples jointly called in 2016), freeze 5 (GRCh38 alignment, approximately 65,000 samples jointly called in 2017), and freeze 8 (GRCh38 alignment, about 140,000 samples jointly called in 2019). Extended Data Table 3 indicates which TOPMed study-consent groups were used in each of several different types of analyses described in this paper. Most analyses were performed on a set of 53,831 samples derived from freeze 5 (‘General variant analyses’ in Extended Data Table 3) or on a subset thereof approved for population genetic studies (‘Population genetics’ in Extended Data Table 3). The set of 53,831 was selected from freeze 5 using samples eligible for dbGaP sharing at the time of analysis, excluding (1) duplicate samples from the same participant; (2) one member of each monozygotic twin pair; (3) samples with questionable identity or low read depth (<98% of variant sites at depth ≥ 10×); and (4) samples with consent types inconsistent with analyses presented here. The ‘unrelated’ sample set consisting of 40,722 samples refers to a subset of the 53,831 samples of individuals who are unrelated with a threshold of third degree (less closely related than first cousins), identified using the PC-AiR method^84^. Exact numbers of samples used in each analysis are listed in Supplementary Table 18.

### High-coverage whole-exome sequencing in BioMe study

From around 10,000 BioMe study samples present in TOPMed freeze 8, we randomly selected 1,000 samples for which whole-exome sequencing (WES) data were available. These samples were whole-exome sequenced using Illumina v4 HiSeq 2500 at an average 36.4× depth. Genetic variants were jointly called using the GATK v.3.5.0 pipeline across all 31,250 BioMe samples with WES data. A series of quality control filters, known as the Goldilocks filter, were applied before data delivery to the Charles Bronfman Institute for Personalized Medicine (IPM). First, a series of filters was applied to particular cells comprising combinations of sites and samples—that is, genotypic information for one individual at one locus. Quality scores were normalized by depth of coverage and used with depth of coverage itself to filter sites, using different thresholds for SNVs and short indels. For SNVs, cells with depth-normalized quality scores less than 3, or depth of coverage less than 7 are set to missing. For indels, cells with depth-normalized quality scores less than 5, or depth of coverage less than 10 are set to missing. Then, variant sites were filtered, such that all samples carrying variation have heterozygous (0/1) genotype calls and all samples carrying heterozygous variation fail the allele balance cut-off; these sites were removed from the dataset at this stage. The allele balance cut-off, as with the depth and quality scores used for cell filtering above, differed depending on whether the site was a SNV or an indel: SNVs require at least one sample to carry an alternative allele balance ≥ 15%, and indels require at least one sample to carry an alternative allele balance ≥ 20%. These filters resulted in the removal of 441,406 sites, leaving 8,761,478 variants in the dataset. After subsetting to 1,000 randomly selected individuals, we had 1,076,707 autosomal variants that passed quality control. We further removed variants with call rate <99% (that is, missing in more than 10 individuals), reducing the number of analysed autosomal variants to 1,044,517. The comparison results of TOPMed WGS and BioMe WES data are described in Supplementary Information 1.3.1.

### Low-coverage WGS and high-coverage WES in the Framingham Heart Study

Investigators of the Framingham Heart Study (FHS) evaluated WGS data from TOPMed in comparison with sequencing data from CHARGE Consortium WGS and WES datasets. Supplementary Table 19 provides the counts and depth of each sequencing effort. The overlap of these three groups is 430 FHS study participants, on whom we report here. We use a subset of 263 unrelated study participants to calculate the numbers of singletons and doubletons, MAF, heterozygosity and all rates, to avoid bias from the family structure. Supplementary Information 1.3.2 provides further detail on the sequencing efforts and a detailed description of the comparison results.

### Identifying pLOF variants

pLOF variants were identified using Loss Of Function Transcript Effect Estimator (LOFTEE) v.0.3-beta^85^ and Variant Effect Predictor (VEP) v.94^86^. The genomic coordinates of coding elements were based on GENCODE v.29^15^. Only stop-gained, frameshift and splice-site-disturbing variants annotated as high-confidence pLOF variants were used in the analysis. The pLOF variants with allele frequency > 0.5% or within regions masked due to poor accessibility were excluded from analysis (see Supplementary Information 1.5 for details).

We evaluated the enrichment and depletion of pLOF variants (allele frequency < 0.5%) in gene sets (that is, terms) from Gene Ontology (GO)^87,88^. For each gene annotated with a particular GO term, we computed the number of pLOF variants per protein-coding base pair, *L*, and proportion of singletons, *S*. We then tested for lower or higher mean *L* and *S* in a GO term using bootstrapping (1,000,000 samples) with adjustment for the gene length of the protein-coding sequence (CDS): (1) sort all genes by their CDS length in ascending order and divide them into equal-size bins (1,000 genes each); (2) count how many genes from a GO term are in each bin; (3) from each bin, sample with replacement the same number of genes and compute the average *L* and *S*; (4) count how many times sampled *L* and *S* were lower or higher than observed values. The *P* values were computed as the proportion of bootstrap samples that exceeded the observed values. The fold change of average *L* and *S* was computed as a ratio of observed values to the average of sampled values. We tested all 12,563 GO terms that included more than one gene. The *P*-value significance threshold was thus ~2 × 10^−6^. The enrichment and depletion of pLOF variants in public gene databases was tested in a similar way.

### Sequencing depth at protein-coding regions

We compared sequencing depth at protein-coding regions in TOPMed WGS and ExAC WES data. The ExAC WES depth at each sequenced base pair on human genome build GRCh37 was downloaded from the ExAC browser website (http://exac.broadinstitute.org). When sequencing depth summary statistics for a base pair were missing, we assumed depth <10× for this base pair. Only protein-coding genes from consensus coding sequence were analysed and the protein-coding regions (CDS) were extracted from GENCODE v.29. When analysing ExAC sequencing depth, we used GENCODE v.29 lifted to human genome build GRCh37. When comparing sequencing depth for each gene individually in TOPMed and ExAC, we used only genes present in both GRCh38 and GRCh37 versions of GENCODE v.29.

### Novel genetic variants in unmapped reads

Analysis of unmapped reads was performed using 53,831 samples from TOPMed data freeze 5. From each sample, we extracted and filtered all read pairs with at least one unmapped mate and used them to discover human sequences that were absent from the reference. The pipeline included four steps: (1) per-sample de novo assembly of unmapped reads; (2) contig alignment to the *Pan paniscus*, *Pan troglodyte*s, *Gorilla gorilla* and *Pongo abelii* genome references and subsequent hominid-reference-based merging and scaffolding of sequences pooled together from all samples; (3) reference placement and breakpoint calling; and (4) variant genotyping. The detailed description of each step is provided in Supplementary Information 1.7.

### Identification of *CYP2D6* alleles using Stargazer’s genotyping pipeline

Details of the Stargazer genotyping pipeline have been described previously^43^. In brief, SNVs and indels in *CYP2D6* were assessed from a VCF file generated using GATK-HaplotypeCaller^89^. The VCF file was phased using the program Beagle^90^ and the 1000 Genomes Project haplotype reference panel. Phased SNVs and indels were then matched to star alleles. In parallel, read depth was calculated from BAM files using GATK-DepthOfCoverage^89^. Read depth was converted to copy number by performing intra-sample normalization^43^. After normalization, structural variants were assessed by testing all possible pairwise combinations of pre-defined copy number profiles against the observed copy number profile of the sample. For new SVs, breakpoints were statistically inferred using changepoint^91^. Information regarding new SVs was stored and used to identify subsequent SVs in copy number profiles. Output data included individual diplotypes, copy number plots and a VCF of SNVs and indels that were not used to define star alleles.

### Genome-wide distribution of genetic variation

#### Contiguous segment analysis

We excluded indels and multi-allelic variants, and categorized the remaining variants as common (allele frequency ≥ 0.005) or rare (allele frequency < 0.005), and as coding or noncoding based on protein-coding exons from Ensembl 94^92^. Variant counts were analysed across 2,739 non-empty (that is, with at least one variant) contiguous 1-Mb chromosomal segments, and counts in segments at the end of chromosomes with length *L* < 10^6^ bp were scaled up proportionally by the factor 10^6^ × *L*^−1^. For each segment, the coding proportion, *C*, was calculated as the proportion of bases overlapping protein-coding exons. The distribution of *C* is fairly narrow, with 80% of segments having *C* ≤ 0.0195, 99% of segments have *C* ≤ 0.067 and only 3 segments having *C* ≥ 0.10. Owing to the significant negative correlation between *C* and the number of variants in a segment, and potential mapping effects, we use linear regression to adjust the variant counts per segment according to the model count = *β* × *C* + *A* + count_adj, where *A* is the proportion of segment bases overlapping the accessibility mask (Supplementary Information 1.5). Unless otherwise noted, we present analyses and results that use these adjusted count values.

#### Concatenated segment analysis

Distinct base classifications were defined by both coding and noncoding annotations (based on Ensembl 94^92^) and CADD in silico prediction scores^21^ (downloaded from the CADD server for all possible SNVs). For each base, we used the maximum possible CADD score (when using the minimum CADD score, results were qualitatively the same). Bases beyond the final base with a CADD score per chromosome were excluded. This resulted in six distinct types of concatenated segments: high (CADD ≥ 20), medium (10 ≤ CADD < 20) and low (CADD < 10) CADD scores for coding and similarly for noncoding variants. Common (allele frequency ≥ 0.005) and rare (allele frequency < 0.005) variant counts were then calculated across these concatenated segments. Multi-allelic variants and those in regions masked due to accessibility were excluded. Counts in segments at the end of chromosomes were scaled up as in the contiguous analysis.

### Singleton clustering analysis

#### Data

From the TOPMed freeze 5 dataset, we selected a subset of 1,000 unrelated individuals of African ancestry, 1,000 unrelated individuals of East Asian ancestry and 1,000 unrelated individuals of European ancestry, with the ancestry of each individual inferred across 7 global reference populations using RFMix^93^. In each of these subsamples, we recalculated the allele counts of each SNV and extracted SNVs that were singletons within that sample, then calculated the distance to the nearest singleton (either upstream or downstream from the focal singleton) occurring within the same individual. Note that a singleton defined here is not necessarily a singleton in the entire TOPMed freeze 5 dataset. We chose to limit the size of each population subsample to *n* = 1,000 for three reasons: first, to ensure the different population subsamples carried roughly a similar number of singletons; second, to ensure homogeneous ancestry within each subsample so that our analysis of singleton clustering patterns was not an artefact of admixed haplotypes; third, to limit the incidence of recurrent mutations at hypermutable sites, which can alter the underlying mutational spectrum of singleton SNVs in large samples^94^. Although the TOPMed Consortium sequenced individuals from several other diverse population groups (for example, Samoan, Hispanic/Latino individuals), the majority of these individuals were of admixed ancestry and the singletons from these smaller samples reflected mutations that have accumulated over a longer period of time, so the mutation spectra and genome-wide distributions of these samples would be more susceptible to distortion by other evolutionary processes such as selection and biased gene conversion^31^.

#### Simulations

To quantify the effects of external branch length heterogeneity on singleton clustering patterns, we used the stdpopsim library^95^ to simulate variants across chromosome 1 for 2,000 European and 2,000 African haploid samples, using a previously reported demographic model^10^. Simulations were performed using a per-site, per-generation mutation rate^96^ of 1.29 × 10^−8^, and using recombination rates derived from the HapMap genetic map^97^. Because our aim was to compare these simulated singletons to unphased singletons observed in the TOPMed data, we randomly assigned each of the 2,000 haploid samples from each population into one of 1,000 diploid pairs, and calculated the inter-singleton distances per diploid sample, ignoring the haplotype on which each simulated singleton originated.

#### Mixture model parameter estimation

The distribution of singletons suggest an underlying nonhomogeneous Poisson process, where the rate of incidence varies across the genome. In other areas of research, it has been shown that the waiting times between events arising from other nonhomogeneous Poisson processes, such as volcano eruptions or extreme weather events, can be accurately modelled as a mixture of exponential distributions^98,99^. Taking a similar approach, we model the distribution of inter-singleton distances across all *S*_*i*_ singletons in individual *i* as a mixture of *K* exponential component distributions (*f*_*k*_(*d*_*i*_;*θ*_*i*,*k*_)), given by:

$$f({d}_{i};{\lambda }_{i},\,{\theta }_{i})=\mathop{\sum }\limits_{k=1}^{K}{\lambda }_{i,k}\,{f}_{k}({d}_{i};{\theta }_{i,k})$$

where *θ*_*i*,1_ < *θ*_*i*,2_ < … < *θ*_*i*,*K*_ and *λ*_*i*,*k*_ = *S*_*i*,*k*_/*S*_*i*_ is the proportion of singletons arising from component $k$, such that ${\sum }_{k=1}^{K}{\lambda }_{i,k}=1$.

We estimate the parameters of this mixture (*λ*_*i*,1_, …, *λ*_*i*,*K*_, *θ*_*i*,1_, …, *θ*_*i*,*K*_) using the expectation–maximization algorithm as implemented in the mixtools R package^100^. Code for this analysis is available for download from the GitHub repository^101^. To identify an optimal number of mixture components, we iteratively fit mixture models for increasing values of *K* and calculated the log-likelihood of the observed data *D* given the parameter estimates $({\hat{\lambda }}_{i,1},\mathrm{...},{\hat{\lambda }}_{i,K},{\hat{\theta }}_{i,1},\mathrm{...},{\hat{\theta }}_{i,K})$, stopping at *K* components if the *P* value of the likelihood ratio test between *K* − 1 and *K* components was >0.01 (χ^2^ test with two degrees of freedom). The goodness-of-fit plateaued at four components for the majority of individuals, so we used the four-component parameter estimates from each individual in all subsequent analyses.

Now let *k*_*i*,*j*_ indicate which of the four processes generated singleton *j* in individual *i*. We calculated the probability of being generated by process *k* as:

$$p({k}_{i,j}=k|{d}_{i,j};\,k\in \{1,\mathrm{...},4\})=\frac{p({d}_{i},k)}{p({d}_{i})}=\frac{{\lambda }_{i,k}\,{f}_{k}({d}_{i};{\theta }_{i,k})}{{\sum }_{k=1}^{4}{\lambda }_{i,k}\,{f}_{k}({d}_{i};{\theta }_{i,k})}.$$

We then classified the process-of-origin for each singleton according to the following optimal decision rule:

$${\hat{k}}_{i,j}={\rm{\arg }}\,{{\rm{\max }}}_{k\in \{1,\mathrm{..}.,4\}}p(k|{d}_{i,j}).$$

#### Identification of mixture component hotspots

After assigning singletons to the most likely mixture component, we pooled singletons across individuals of a given ancestry group and counted the number of occurrences in each component in non-overlapping 1-Mb windows throughout the genome. We defined hotspots as the top 5% of 1-Mb bins containing the most singletons in a component in each ancestry group.

#### Modelling the relationship between clustering patterns and genomic features

In each 1-Mb window, we calculated the average signal for 12 genomic features (H3K27ac, H3K27me3, H3K36me3, H3K4me1, H3K4me3, H3K9ac, H3K9me3, exon density, DNase hypersensitivity, CpG island density, lamin-associated domain density and recombination rate), using the previously described source datasets^31^. For each mixture component, we then applied the following negative binomial regression model to estimate the effects of each feature on the density of that component in 1-Mb windows:

$$\log ({Y}_{a,k,w})={\beta }_{0}+{\beta }_{1}{X}_{1,w}+\mathrm{...}+{\beta }_{12}{X}_{12,w}$$

Where *Y*_*a*,*k*,*w*_ is the number of singletons in ancestry subsample *a* of mixture component *k* in window *w* and *X*_1,*w*_, …, *X*_12,*w*_ are the signals of each of the 12 genomic features in corresponding window *w*.

### Evolutionary genetics of individuals with diverse ancestry

#### Rare variant sharing

In these analyses, we used 39,722 unrelated individuals that had provided consent for population genetics research. Each individual was grouped into their TOPMed study, except for individuals from the AFGen project, which were treated as one study (Extended Data Tables 1, 2). Individuals from the FHS and ARIC projects individuals, which overlapped with the AFGen project, remained in their respective studies and were not grouped into the AFGen project. Individuals for whom the population group was either missing or ‘other’ were removed from the analysis. We then removed all indels, multi-allelic variants and singletons from the remaining 39,168 individuals. Each study was then split by population group. We excluded studies that had fewer than 19 samples from the analysis; however all 39,168 samples were used to define singleton filtering. We used the Jaccard index^102^, *J*, to determine the intersection of rare variants (2 ≤ sample count ≤ 100) between two individuals divided by the union of the rare variants of that pair, where the sample count indicates the number of individuals with either a heterozygote or homozygote variant. We then determined the average *J* value between and within each study.

To confirm that *J* is not biased by sample size, we randomly sampled 500 individuals from each of two studies with European (AFGen and FHS) and African (COPDGene and JHS) population groups in TOPMed freeze 3, without replacement. We then recalculated *J* between and within these randomly sampled studies, considering alternative allele counts between 2 and 100 within these 2,000 individuals.

#### Haplotype sharing

We used the RefinedIBD program^103^ to call segments of identical-by-descent (IBD) sharing of length ≥ 2 cM on the autosomes using passing SNVs with MAF > 5%. All 53,831 samples were included in this analysis, and we used genotype data phased with Eagle2^81^. As IBD logarithm of odds (LOD) scores are often deflated in populations with strong founding bottlenecks, such as the Amish, we used a LOD score threshold of 1.0 instead of the default 3.0. To account for possible phasing and genotyping errors, we filled gaps between IBD segments for the same pair of individuals if the gap had a length of at most 0.5 cM and at most one discordant genotype. As a result of the lower LOD threshold, regions with a low variant density can have an excess of apparent IBD segments. We therefore identified regions with highly elevated levels of detected IBD using a previously described procedure^104^ and removed any IBD segments that fell wholly within these regions.

We divided the data by study and by population group within each study. In the analyses of IBD sharing levels and recent effective size, we did not include studies without appropriate consent or population groups with fewer than 80 individuals within a study. We calculated the total length of IBD segments for each pair of individuals, and we averaged these totals within each population group in a study and between each pair of population-by-study groups. We also estimated recent effective population sizes for each group using IBDNe^104^.

#### Demographic estimation under selection at linked sites

We selected 2,416 samples from the TOPMed data freeze 3 that (1) had a high percentage of European ancestry; (2) were unrelated; and (3) gave consent for population genetics research. More detailed information about ancestry estimation and filters is provided in Supplementary Information 1.10.

We performed several steps to filter the genome for high-quality neutral sites, which were based on a previously described ascertainment scheme^30^ (Supplementary Information 1.10). After filtering, positions in the genome were annotated for how strongly affected they were by selection at linked sites using the background selection coefficient, McVicker’s *B* statistic^60^. We used all sites annotated with a *B* value for performing general analyses. However, when performing demographic inferences, we limited our analyses to regions of the genome within the top 1% of the genome-wide distribution of *B* (*B* ≥ 0.994). These sites correspond to regions of the genome inferred to be under the weakest amount of background selection (that is, under the weakest effects of selection at linked sites). Sites in the genome were also polarized to ancestral and derived states using ancestral annotations called with high-confidence from the GRCh37 e71 ancestral sequence. After keeping only polymorphic bi-allelic sites, we had 20,324,704 sites, of which 191,631 had *B* ≥ 0.994. We also identified 91,177 fourfold degenerate synonymous sites (irrespective of *B*) that were polymorphic (bi-allelic) and had high-confidence ancestral and derived states.

We performed demographic inference with the moments^105^ program by fitting a model of exponential growth with three parameters (*N*_Eur0_, *N*_Eur_, *T*_Eur_) to the site-frequency spectrum. This included two free parameters: the starting time of exponential growth (*T*_Eur_) and the ending population size after growth (*N*_Eur_). The ancestral size parameter (that is, the population size when growth begins), *N*_Eur0_, was kept constant in our model such that the relative starting size of the population was always 1. We applied the inference procedure to either fourfold degenerate sites or sites with *B* ≥ 0.994. The site frequency spectrum used for inference was unfolded and based on the polarization step described above. The inference procedure was fit using sample sizes (2*N*) of 1,000, 2,000, 3,000, 4,000 and 4,832 chromosomes. To convert the scaled genetic parameters output by the inference procedure to physical units, we used the resulting theta (also inferred by moments) and a mutation rate^106^ of 1.66 × 10^−8^ to generate corresponding effective population sizes (*N*_e_). To convert generations to years, we assumed a generation time of 25 years. The 95% confidence intervals were generated by resampling the site frequency spectrum 1,000 times and using the Godambe information matrix to generate parameter uncertainties^107^. A more detailed description is available in Supplementary Information 1.10.

#### Selection

We started with 39,649 unrelated individuals selected from the TOPMed data freeze 5 for which we had consent for population genetic analyses (Extended Data Table 3). As the singleton density score (SDS) requires thousands of samples and a baseline demographic history, we subset our data by population group and limited our population analysis to those population groups for which we had well-studied demographic histories: broadly European, broadly African and broadly East Asian. To avoid potential problems introduced by admixture, we required that our samples had more than 90% inferred European, African or East Asian ancestry as inferred by a seven-way ancestry inference pipeline (Supplementary Information 1.11). This left *n* = 21,196 European samples, *n* = 2,117 African samples and *n* = 1,355 East Asian samples. We specifically excluded Amish samples from the European group as they are a unique founder population. We analysed each population separately. Only bi-allelic sites with an unambiguous ancestral state, inferred using the WGSA pipeline^108^, were used. Sites near chromosome boundaries, near centromeres and in regions with poor accessibility were excluded. We used the previously published R scripts^61^ to perform all demographic history simulations and SDS computations in each population. We then normalized raw SDS scores within 1% frequency bins and treated the normalized scores as *Z*-scores to convert them to *P* values as described previously^61^. Raw and normalized SDS scores are included in Supplementary Data 2.

### TOPMed imputation panel

#### Construction

We divided each autosomal chromosome and the X chromosome into overlapping chunks (with chunk size of 1 Mb each and with 0.1 Mb overlap between consecutive chunks), and then phased each of the chunks using Eagle v.2.4^81^. We removed all singleton sites and compressed the haplotype chunks into m3vcf format^109^. Afterwards, we ligated the compressed haplotype chunks for each chromosome to generate the final reference panel.

#### Evaluation of imputation accuracy

For all TOPMed individuals, genetic ancestries were estimated using the top four principal components projected onto the principal component space of 938 Human Genome Diversity Project (HGDP) individuals using verifyBamID2^110^. For each TOPMed individual, we identified the 10 closest individuals from 2,504 individuals from the 1000 Genomes Project phase 3 based on Euclidean distances in the principal component space estimated by verifyBamID2. If all of the 10 closest individuals from the 1000 Genomes Project phase 3 belonged to the same super-population—among African, admixed American, East Asian, European and South Asian populations—we estimated that the TOPMed individual also belonged to that super-population. Among the 97,256 reference panel individuals, 90,339 (93%) were assigned to a super-population, with the following breakdown: African, 24,267 individuals; admixed American, 17,085 individuals; European, 47,159 individuals; East Asian, 1,184 individuals; South Asian, 644 individuals. We randomly selected 100 individuals from each super-population in the BioMe TOPMed study, and selected markers on chromosome 20 present on the Illumina HumanOmniExpress (8v1-2_A) array. The selected genotypes were phased with Eagle 2.4.1^81^, using the 1000 Genomes Project phase 3 (*n* = 2,504), Haplotype Reference Consortium (HRC, *n* = 32,470) and TOPMed (*n* = 96,756) reference panels, excluding the 500 individuals from the TOPMed reference panel. The phased genotypes were imputed using Minimac4^111^ from each reference panel, and the imputation accuracy was estimated as the squared correlation coefficient (*r*^2^) between the imputed dosages and the genotypes calls from the sequence data. The allele frequencies were estimated among all TOPMed individuals estimated to belong to the same super-population, and the *r*^2^ values were averaged across variants in each MAF category. Variants present in 100 sequenced individuals but absent from the reference panels were assumed to have *r*^2^ = 0 for the purposes of computing the average *r*^2^. The minimum MAF to achieve *r*^2^ > 0.3 was calculated from the average *r*^2^ in each MAF category by finding the MAF that crosses *r*^2^ = 0.3 using linear interpolation. The average number of rare variants (MAF < 0.5%) and the fraction of imputable rare variants (*r*^2^ > 0.3) were calculated based on the number of non-reference alleles in imputed samples above and below the minimum MAF, assuming Hardy–-Weinberg equilibrium.

#### Imputation of the UK Biobank to the TOPMed panel and association analyses

After phasing the UK Biobank genetic data (carried out on 81 chromosomal chunks using Eagle v.2.4), the phased data were converted from GRCh37 to GRCh38 using LiftOver^112^. Imputation was performed using Minimac4^111^.

We compared the correlation of genotypes between the exome-sequencing data released by the UK Biobank (following their SPB pipeline^113^) and the TOPMed-imputed genotypes. The comparison assessed 49,819 individuals and 3,052,260 autosomal variants that were found in both the exome-sequencing and TOPMed-imputed datasets (matched by chromosome, position and alleles, and with an imputation quality of at least 0.3 in the TOPMed-imputed data). We split the variants into MAF bins for which the MAF from the exome data was used to define the bins, and computed Pearson correlations averaged within each bin.

We tested single pLOF, nonsense, frameshift and essential splice-site variants^85,86^ for association with 1,419 PheCodes constructed from composites of ICD-10 (International Classification of Diseases 10th revision) codes to define cases and controls. Construction of the PheCodes has been previously described^114^. We performed the association analysis in the ‘white British’ individuals, which resulted in 408,008 individuals after the following quality control metrics were applied: (1) samples did not withdraw consent from the UK Biobank study as of the end of 2019; (2) ‘submitted gender’ matches ‘inferred sex’; (3) phased autosomal data available; (4) outliers for the number of missing genotypes or heterozygosity removed; (5) no putative sex chromosome aneuploidy; (6) no excess of relatives; (7) not excluded from kinship inference; and (8) in the UK Biobank defined the ‘white British’ ancestry subset. To perform the association analyses, we used a logistic mixed model test implemented in SAIGE^114^ with birth year and the top four principal components (computed from the white British subset) as covariates. For the pLOF burden tests, for each autosomal gene with at least two rare pLOF variants (*n* = 12,052 genes), a burden variable was created in which dosages of rare pLOF variants were summed for each individual. This sum of dosages was tested for association with the 1,419 traits using SAIGE. The same covariates used in the single-variant tests were included. For both the single-variant and the burden tests, we used 5 × 10^−8^ as the genome-wide significance threshold.

### Reporting summary

Further information on research design is available in the Nature Research Reporting Summary linked to this paper.

## Online content

Any methods, additional references, Nature Research reporting summaries, source data, extended data, supplementary information, acknowledgements, peer review information; details of author contributions and competing interests; and statements of data and code availability are available at 10.1038/s41586-021-03205-y.

### Supplementary information

Supplementary Information This file contains details about the TOPMed project and analyses described in the main text, complete list of additional authors from the TOPMed Consortium, grant acknowledgements for each author, acknowledgements and ethics statements for the contributing TOPMed studies.

Reporting Summary

Supplementary Tables and Figures This file contains Supplementary Tables 1-30 and Supplementary Figures 1-51 with their corresponding legends.

Supplementary Data 1 This spreadsheet contains table with between-cohort rare variant sharing values from Figure 4.

Supplementary Data 2 This file contains raw and normalized Singleton Density Scores (SDS) from the section on human adaptations.

## Figures and Tables

**Extended Data Table 1:**
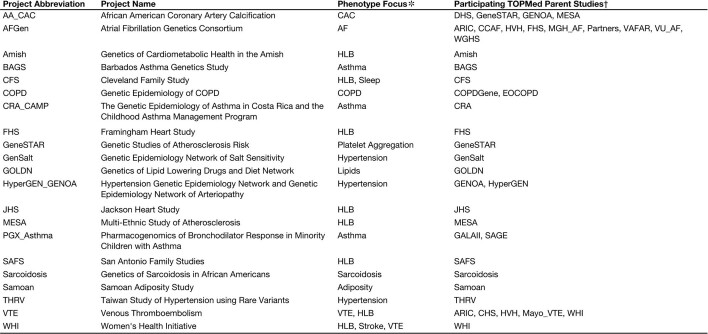
TOPMed projects and participating parent studies included in genotype data freeze 5

See Supplementary Information 1.1.2 for definitions of TOPMed projects and parent studies. AF, atrial fibrillation; CAC, coronary artery calcification; HLB, general heart, lung and blood; VTE, venous thromboembolism. Note, some case-only collections are included. See Extended Data Table 2 for study abbreviations and additional study information.

*Primary phenotype focus for TOPMed samples.

^†^Some TOPMed studies participate in more than one project.

**Extended Data Table 2:**
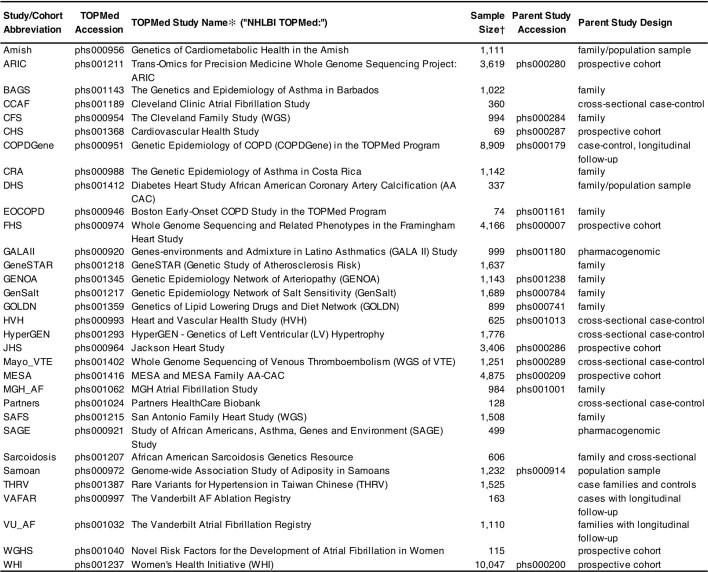
Studies that contributed to the freeze-5 genotype call set

Each study has a dbGaP accession for the TOPMed sequence data and genotypes, although some also have pre-existing parent study accessions. Phenotypic data are mainly in the parent accessions, although some are in the TOPMed accessions. See also Supplementary Figs. 39 and 40 for information about the ancestral and/or ethnic and sex composition of each study. The relationships between these studies and their TOPMed project(s) are summarized in Extended Data Table 1. All of the TOPMed and parent study accessions in this table have been released on dbGaP (see https://www.ncbi.nlm.nih.gov/gap/?term=TOPMed and https://www.nhlbiwgs.org/group/project-studies?field_is_this_a_value=sub).

*Study name as it appears in dbGaP, with ‘NHLBI TOPMed:’ prepended.

^†^Approximate sample size for freeze-4 and freeze-5 releases combined.

**Extended Data Table 3:**
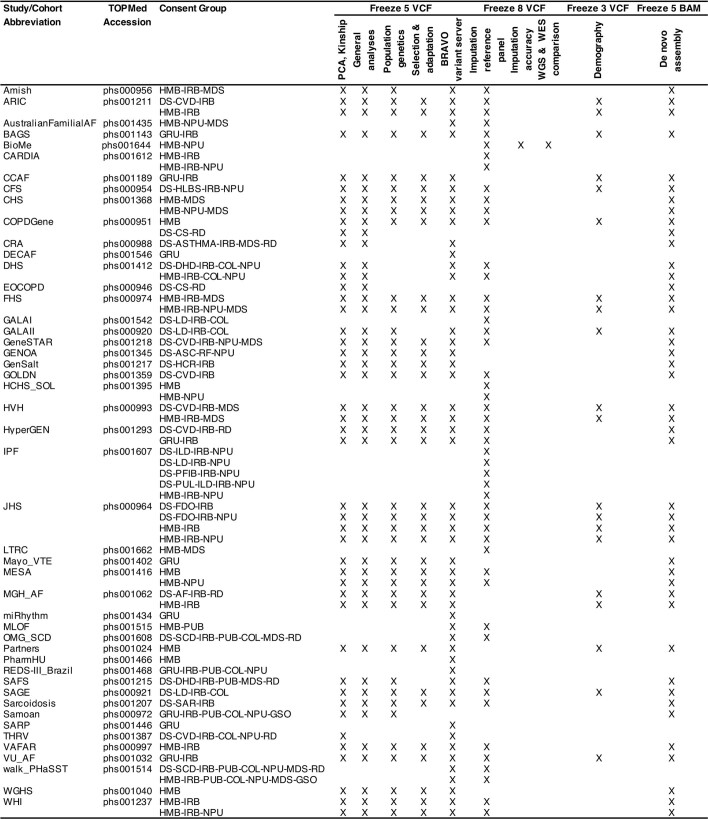
TOPMed study-consent groups used in analyses and tools

Consent group data use limitations are defined as follows: GRU, general research use; HMB, limited to health, medical and/or biomedical purposes; DS, use of the data must be related to specified disease. Consent group data use limitation modifiers include the following: IRB, requestor must provide documentation of local IRB approval; PUB, requestor agrees to make results of studies using the data available to the larger scientific community; COL, requestor must provide a letter of collaboration with the primary study investigator(s); NPU, use of the data are limited to not-for-profit organizations; MDS, use of the data includes methods development research; GSO, use of the data are limited to genetic studies only. AF, atrial fibrillation; ASC-RF, arteriosclerosis and its risk factors; CVD, cardiovascular disease; CS, chronic obstructive pulmonary disease (COPD) and smoking; DHD, diabetes and heart disease; FDO, focus disease only (in JHS, FDO is blood pressure, heart/CVD, obesity, diabetes, kidney disease, or lung disease and risk factors); HCR, high blood pressure and related cardiovascular-renal disease; HLBS, heart, lung, blood and sleep disorders; ILD, interstitial lung disease; LD, lung disease; PFIB, pulmonary fibrosis; PUL, pulmonary, interstitial lung disease; RD, related disorders; SAR, sarcoidosis; SCD, sickle cell disease.

**Extended Data Table 4:**
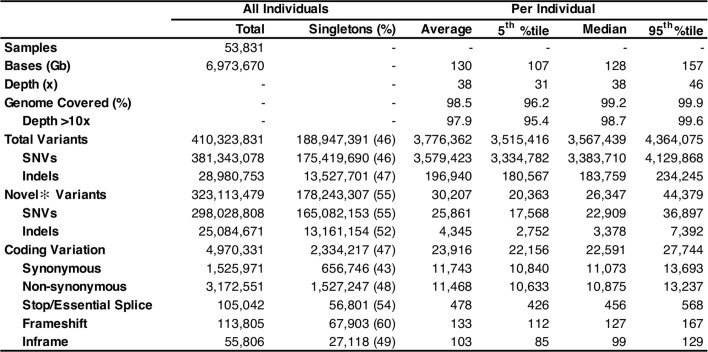
Coverage, sequencing depth and number of variants

*Variant was not present in dbSNP build 149, the most recent dbSNP version without TOPMed submissions.

**Extended Data Table 5:**
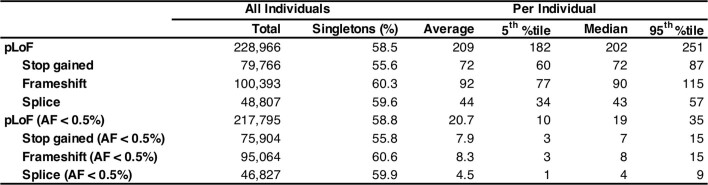
pLOF variants in 53,831 individuals

## Data Availability

A detailed description of the TOPMed participant consents and data access is provided in Box 1. TOPMed data used in this manuscript are available through dbGaP. The dbGaP accession numbers for all TOPMed studies referenced in this paper are listed in Extended Data Tables 2, 3. A complete list of TOPMed genetic variants with summary level information used in this manuscript is available through the BRAVO variant browser (bravo.sph.umich.edu). The TOPMed imputation reference panel described in this manuscript can be used freely for imputation through the NHLBI BioData Catalyst at the TOPMed Imputation Server (https://imputation.biodatacatalyst.nhlbi.nih.gov/). DNA sequence and reference placement of assembled insertions are available in VCF format (without individual genotypes) on dbGaP under the TOPMed GSR accession phs001974.

## Code availability

All code for TOPMed data quality checks and variant calling is available at https://github.com/statgen/topmed_variant_calling. Code for the WGS and WES data comparisons is available at https://github.com/statgen/sequencing_comparison. Code for modelling the singleton distance distribution is available at https://github.com/carjed/topmed_singleton_clusters. Code for identifying novel genetic variants in unmapped reads is available at https://github.com/nygenome/topmed_unmapped. Code for gene-burden association tests using rare pLOF variants is available at https://github.com/sgagliano/GeneBurden. Code for the imputed and genotype UK Biobank WES data comparisons is available at https://github.com/sgagliano/UKB_WES_vs_TOPMed_IMP.
