# Supplementary material for: Sequencing of 53,831 diverse genomes from the NHLBI TOPMed Program
Source: Nature. Author manuscript; Available in PMC 2021 Oct 6. (PMC7875770; doi:10.1038/s41586-021-03205-y)
Supplement: 1675193_SuppInfoGuide [file NIHMS1675193-supplement-1675193_SuppInfoGuide.docx]

Supplementary file: Supplementary_Information.pdf

Title: Supplementary Information

Description:

This file contains details about the TOPMed project and analyses described in the main text, complete list of additional authors from the TOPMed Consortium, grant acknowledgements for each author, acknowledgements and ethics statements for the contributing TOPMed studies.

Supplementary file: Supplementary_Tables_and_Figures.pdf

Title: Supplementary Tables and Figures

Description:

This file contains Supplementary Tables 1-30 and Supplementary Figures 1-51 with their corresponding legends.

Supplementary file: Supplementary_Data_1.xlsx

Title: Between and within cohort rare variant sharing

Description:

This spreadsheet contains table with between-cohort rare variant sharing values from Figure 4.

Supplementary file: Supplementary_Data_2.zip

Title: SDS scores

Description:

This file contains raw and normalized Singleton Density Scores (SDS) from the section on human adaptations.
