# Supplementary material for: Sequencing of 53,831 diverse genomes from the NHLBI TOPMed Program
Source: Nature. Author manuscript; Available in PMC 2021 Oct 6. (PMC7875770; doi:10.1038/s41586-021-03205-y)
Supplement: 1675193_SuppInfo [file NIHMS1675193-supplement-1675193_SuppInfo.pdf]

SUPPLEMENTARY INFORMATION

Sequencing of 53,831 diverse genomes from the NHLBI TOPMed Program

Daniel Taliun[1*], Daniel N. Harris[2,3,4*], Michael D. Kessler[2,3,4*], Jedidiah Carlson[5,6*], Zachary A. Szpiech[7,8*], Raul Torres[9*], Sarah A. Gagliano Taliun[1*], André Corvelo[10*], Stephanie M. Gogarten[11], Hyun Min Kang[1], Achilleas N. Pitsillides[12], Jonathon LeFaive[1], Seung-been Lee[6], Xiaowen Tian[11], Brian L. Browning[13], Sayantan Das[1], Anne-Katrin Emde[10], Wayne E. Clarke[10], Douglas P. Loesch[2,3,4], Amol C. Shetty[2,3,4], Thomas W. Blackwell[1], Albert V. Smith[1], Quenna Wong[11], Xiaoming Liu[14], Matthew P. Conomos[11], Dean M. Bobo[15], François Aguet[16], Christine Albert[17], Alvaro Alonso[18], Kristin G. Ardlie[16], Dan E. Arking[19], Stella Aslibekyan[20], Paul L. Auer[21], John Barnard[22], R. Graham Barr[23,24], Lucas Barwick[25], Lewis C. Becker[26], Rebecca L. Beer[27], Emelia J. Benjamin[28,29,30], Lawrence F. Bielak[31], John Blangero[32,33], Michael Boehnke[1], Donald W. Bowden[34], Jennifer A. Brody[35,36], Esteban G. Burchard[37,38], Brian E. Cade[39,40], James F. Casella[41,42], Brandon Chalazan[43], Daniel I. Chasman[44,45], Yii-Der Ida Chen[46], Michael H. Cho[47], Seung Hoan Choi[16], Mina K. Chung[48,49,50], Clary B. Clish[51], Adolfo Correa[52,53,54], Joanne E. Curran[32,33], Brian Custer[55,56], Dawood Darbar[57], Michelle Daya[58], Mariza de Andrade[59], Dawn L. DeMeo[47], Susan K. Dutcher[60], Patrick T. Ellinor[61], Leslie S. Emery[11], Celeste Eng[38], Diane Fatkin[62,63,64], Tasha Fingerlin[65], Lukas Forer[66], Myriam Fornage[67], Nora Franceschini[68], Christian Fuchsberger[1,66,69], Stephanie M. Fullerton[70], Soren Germer[10], Mark T. Gladwin[71,72,73], Daniel J. Gottlieb[74,75], Xiuqing Guo[46], Michael E. Hall[52], Jiang He[76,77], Nancy L. Heard-Costa[30,78], Susan R. Heckbert[36,79], Marguerite R. Irvin[80], Jill M. Johnsen[35,81], Andrew D. Johnson[30,82], Robert Kaplan[83], Sharon L.R. Kardia[31], Tanika Kelly[76], Shannon Kelly[84,85,86], Eimear E. Kenny[15], Douglas P. Kiel[39,87,88,89], Robert Klemmer[1], Barbara A. Konkle[35,81], Charles Kooperberg[90], Anna Köttgen[91,92], Leslie A. Lange[93], Jessica Lasky-Su[39,40,47,94], Daniel Levy[28,30,82], Xihong Lin[95], Keng-Han Lin[1], Chunyu Liu[12], Ruth J.F. Loos[96,97], Lori Garman[98], Robert Gerszten[99], Steven A. Lubitz[17], Kathryn L. Lunetta[12], Angel C.Y. Mak[38], Ani Manichaikul[100,101], Alisa K. Manning[39,102,103], Rasika A. Mathias[104], David D. McManus[105], Stephen T. McGarvey[106,107,108], James B. Meigs[109], Deborah A. Meyers[110], Julie L. Mikulla[27], Mollie A. Minear[27], Braxton D. Mitchell[3,4,111], Sanghamitra Mohanty[112,113], May E. Montasser[3,4], Courtney Montgomery[98], Alanna C. Morrison[114], Joanne M. Murabito[28], Andrea Natale[112], Pradeep Natarajan[39,61,115,116], Sarah C. Nelson[11], Kari E. North[68], Jeffrey R. O'Connell[3,4], Nicholette D. Palmer[34], Nathan Pankratz[117], Gina M. Peloso[12], Patricia A. Peyser[31], Jacob Pleiness[1], Wendy S. Post[118], Bruce M. Psaty[35,36,79,119,120], D.C. Rao[121], Susan Redline[39,40], Alexander P. Reiner[79,90], Dan Roden[122], Jerome I. Rotter[46], Ingo Ruczinski[123], Chloé Sarnowski[12], Sebastian Schoenherr[66], David A. Schwartz[124], Jeong-Sun Seo[125,126,127], Sudha Seshadri[30,128], Vivien A. Sheehan[129], Wayne H. Sheu[130], M. Benjamin Shoemaker[122], Nicholas L. Smith[79,120,131], Jennifer A. Smith[31,132], Nona Sotoodehnia[36], Adrienne M. Stilp[11], Weihong Tang[133], Kent D. Taylor[46], Marilyn Telen[134], Timothy A. Thornton[11], Russell P. Tracy[135], David J. Van Den

Berg[136], Ramachandran S. Vasan[28,30], Karine A. Viaud-Martinez[137], Scott Vrieze[138], Daniel E. Weeks[139,140], Bruce S. Weir[11], Scott T. Weiss[39,40,47,94], Lu-Chen Weng[17], Cristen J. Willer[5,141,142], Yingze Zhang[71,72,73], Xutong Zhao[1], Donna K. Arnett[143], Allison E. Ashley-Koch[144], Kathleen C. Barnes[58], Eric Boerwinkle[145,146], Stacey Gabriel[16], Richard Gibbs[146], Kenneth M. Rice[11], Stephen S. Rich[100,101], Edwin K. Silverman[47], Pankaj Qasba[27], Weiniu Gan[27], NHLBI Trans-Omics for Precision Medicine (TOPMed) Consortium†, George J. Papanicolaou[27], Deborah A. Nickerson[6,147,148], Sharon R. Browning[11], Michael C. Zody[10], Sebastian Zöllner[1,149], James G. Wilson[150], L Adrienne Cupples[12,30], Cathy C. Laurie[11], Cashell E. Jaquish[27], Ryan D. Hernandez[37,151,152,153,154], Timothy D. O'Connor[2,3,4], Gonçalo R. Abecasis[1]

1 - Department of Biostatistics and Center for Statistical Genetics, University of Michigan School of Public Health, Ann Arbor, MI; 2 - Institute for Genome Sciences, University of Maryland School of Medicine, Baltimore, MD; 3 - Program in Personalized and Genomic Medicine, University of Maryland School of Medicine, Baltimore, MD; 4 - Department of Medicine, University of Maryland School of Medicine, Baltimore, MD; 5 - Department of Computational Medicine and Bioinformatics, University of Michigan, Ann Arbor, MI; 6 - Department of Genome Sciences, University of Washington, Seattle, WA; 7 - Department of Biology, Pennsylvania State University, University Park, PA; 8 - Institute for Computational and Data Sciences, Pennsylvania State University, University Park, PA; 9 - Biomedical Sciences Graduate Program, University of California, San Francisco, CA; 10 - New York Genome Center, New York, NY; 11 - Department of Biostatistics, University of Washington, Seattle, WA; 12 - Department of Biostatistics, Boston University School of Public Health, Boston, MA; 13 - Department of Medicine, Division of Medical Genetics, University of Washington, Seattle, WA; 14 - USF Genomics, College of Public Health, University of South Florida, Tampa, FL; 15 - Icahn School of Medicine at Mount Sinai, New York, NY; 16 - The Broad Institute of MIT and Harvard, Cambridge, MA; 17 - Massachusetts General Hospital, Boston, MA; 18 - Department of Epidemiology, Rollins School of Public Health, Emory University, Atlanta, GA; 19 - McKusick-Nathans Institute, Department of Genetic Medicine, Johns Hopkins University School of Medicine, Baltimore, MD; 20 - University of Alabama, Birmingham, AL; 21 - Zilber School of Public Health, University of Wisconsin Milwaukee, Milwaukee, WI; 22 - Cleveland Clinic, Cleveland, OH; 23 - Department of Medicine, Columbia University Medical Center, New York, NY; 24 - Department of Epidemiology, Columbia University Medical Center, New York, NY; 25 - The Emmes Corporation, Rockville, MD; 26 - Johns Hopkins University, Baltimore, MD; 27 - National Heart, Lung, and Blood Institute, National Institutes of Health, Bethesda, MD; 28 - Department of Medicine, Boston University School of Medicine, Boston, MA; 29 - Department of Epidemiology, Boston University School of Public Health, Boston, MA; 30 - Framingham Heart Study, Framingham, MA; 31 - Department of Epidemiology, University of Michigan School of Public Health, Ann Arbor, MI; 32 - Department of Human Genetics, University of Texas Rio Grande Valley School of Medicine, Brownsville, TX; 33 - South Texas Diabetes and Obesity Institute, University of Texas Rio Grande Valley School of Medicine, Brownsville, TX; 34 - Department of Biochemistry, Wake Forest School of Medicine, Winston-Salem, NC; 35 - Department of Medicine, University of

Washington, Seattle, WA; 36 - Cardiovascular Health Research Unit, University of Washington, Seattle, WA; 37 - Department of Bioengineering and Therapeutic Sciences, University of California, San Francisco, CA; 38 - Department of Medicine, University of California, San Francisco, CA; 39 - Department of Medicine, Harvard Medical School, Boston, MA; 40 - Department of Medicine, Brigham and Women's Hospital, Boston, MA; 41 - Department of Pediatrics, Johns Hopkins University, Baltimore, MD; 42 - Division of Pediatric Hematology, Johns Hopkins University, Baltimore, MD; 43 - Department of Medical Genetics, University of British Columbia, Vancouver, BC; 44 - Division of Preventive Medicine, Brigham and Women's Hospital, Boston, MA; 45 - Harvard Medical School, Boston, MA; 46 - The Institute for Translational Genomics and Population Sciences, Department of Pediatrics, The Lundquist Institute for Biomedical Innovation at Harbor-UCLA Medical Center, Torrance, CA; 47 - Channing Division of Network Medicine, Department of Medicine, Brigham and Women's Hospital, Boston, MA; 48 - Department of Cardiovascular Medicine, Heart & Vascular Institute, Cleveland Clinic, Cleveland, OH; 49 - Department of Cardiovascular and Metabolic Sciences, Lerner Research Institute, Cleveland Clinic, Cleveland, OH; 50 - Cleveland Clinic Lerner College of Medicine of Case Western Reserve University, Cleveland, OH; 51 - Metabolomics Platform, The Broad Institute of MIT and Harvard, Cambridge, MA; 52 - Department of Medicine, University of Mississippi Medical Center, Jackson, MS; 53 - Department of Pediatrics, University of Mississippi Medical Center, Jackson, MS; 54 - Department of Population Health Science, University of Mississippi Medical Center, Jackson, MS; 55 - Vitalant Research Institute, San Francisco, CA; 56 - Department of Laboratory Medicine, UCSF, San Francisco, CA; 57 - Department of Medicine, University of Illinois at Chicago, Chicago, IL; 58 - School of Medicine, University of Colorado, Aurora, CO; 59 - Mayo Clinic, Rochester, MN; 60 - McDonnell Genome Institute and Department of Genetics, Washington University, St Louis, MO; 61 - Program in Medical and Population Genetics, The Broad Institute of MIT and Harvard, Cambridge, MA; 62 - Molecular Cardiology Division, Victor Chang Cardiac Research Institute, Darlinghurst, NSW, Australia; 63 - Faculty of Medicine, University of New South Wales, Kensington, NSW, Australia; 64 - Cardiology Department, St. Vincent’s Hospital, Darlinghurst, NSW, Australia; 65 - National Jewish Health, Center for Genes, Environment and Health, Denver, CO; 66 - Institute of Genetic Epidemiology, Department of Genetics and Pharmacology, Medical University of Innsbruck, Innsbruck, Austria; 67 - Institute of Molecular Medicine, University of Texas Health Science Center at Houston, Houston, TX; 68 - Department of Epidemiology, University of North Carolina, Chapel Hill, NC; 69 - Institute for Biomedicine, Eurac Research, Bolzano, Italy; 70 - Department of Bioethics & Humanities, University of Washington School of Medicine, Seattle, WA; 71 - Pittsburgh Heart, Lung, Blood and Vascular Medicine Institute, University of Pittsburgh, Pittsburgh, PA; 72 - Pulmonary, Allergy and Critical Care Medicine, University of Pittsburgh, Pittsburgh, PA; 73 - Department of Medicine, University of Pittsburgh, Pittsburgh, PA; 74 - VA Boston Healthcare System, Boston, MA; 75 - Division of Sleep and Circadian Disorders, Brigham and Women's Hospital, Boston, MA; 76 - Department of Epidemiology, Tulane University, New Orleans, LA; 77 - Tulane University Translational Science Institute, Tulane University, New Orleans, LA; 78 - Department of Neurology, Boston University School of

Medicine, Boston, MA; 79 - Department of Epidemiology, University of Washington, Seattle, WA; 80 - Department of Epidemiology, University of Alabama at Birmingham, Birmingham, AL; 81 - Bloodworks Northwest Research Institute, Seattle, WA; 82 - Population Sciences Branch, National Heart, Lung, and Blood Institute, National Institutes of Health, Framingham, MA; 83 - Albert Einstein College of Medicine, New York, NY; 84 - Department of Epidemiology, Vitalant Research Institute, San Francisco, CA; 85 - Department of Pediatrics, UCSF Benioff Children's Hospital, Oakland, CA; 86 - Division of Pediatric Hematology, UCSF Benioff Children's Hospital, Oakland, CA; 87 - Marcus Institute for Aging Research/Harvard Medical School, Hebrew SeniorLife, Boston, MA; 88 - Department of Medicine, Beth Israel Deaconess Medical Center, Boston, MA; 89 - Associate Member Broad Institute of Harvard & MIT; 90 - Division of Public Health Sciences, Fred Hutchinson Cancer Research Center, Seattle, WA; 91 - Department of Epidemiology, Johns Hopkins University, Baltimore, MD; 92 - Institute of Genetic Epidemiology, Faculty of Medicine and Medical Center - University of Freiburg, Freiburg, Germany; 93 - Department of Medicine, University of Colorado at Denver, Aurora, CO; 94 - Brigham and Women's Hospital, Boston, MA; 95 - Biostatistics and Statistics, Harvard University, Boston, MA; 96 - The Charles Bronfman Institute for Personalized Medicine, Icahn School of Medicine at Mount Sinai, New York, NY; 97 - The Mindich Child Health and Development Institute, Icahn School of Medicine at Mount Sinai, New York, NY; 98 - Department of Genes and Human Disease, Oklahoma Medical Research Foundation, Oklahoma City, OK; 99 - Beth Israel Deaconess Medical Center, Boston, MA; 100 - Center for Public Health Genomics, University of Virginia, Charlottesville, VA; 101 - Department of Public Health Sciences, University of Virginia, Charlottesville, VA; 102 - Clinical and Translational Epidemiology Unit, Mongan Institute, Massachusetts General Hospital, Boston, MA; 103 - Metabolism Program, The Broad Institute of MIT and Harvard, Cambridge, MA; 104 - Department of Medicine, Johns Hopkins University, Baltimore, MD; 105 - Cardiovascular Medicine, University of Massachusetts Medical School, Worcester, MA; 106 - International Health Institute, Brown University, Providence, RI; 107 - Department of Epidemiology, Brown University, Providence, RI; 108 - Department of Anthropology, Brown University, Providence, RI; 109 - Division Of General Internal Medicine, Massachusetts General Hospital, Harvard Medical School, The Broad Institute of MIT and Harvard, Boston, MA; 110 - University of Arizona, Tucson, AZ; 111 - Geriatrics Research and Education Clinical Center, Baltimore Veterans Administration Medical Center, Baltimore, MD; 112 - Texas Cardiac Arrhythmia Institute, St. David's Medical Center, Austin, TX; 113 - Department of Internal Medicine, Dell Medical School, Austin, TX; 114 - Human Genetics Center, Department of Epidemiology, Human Genetics, and Environmental Sciences, School of Public Health, University of Texas Health Science Center at Houston, Houston, TX; 115 - Cardiovascular Research Center, Massachusetts General Hospital, Boston, MA; 116 - Center for Genomic Medicine, Massachusetts General Hospital, Boston, MA; 117 - Department of Laboratory Medicine and Pathology, University of Minnesota, Minneapolis, MN; 118 - Division of Cardiology, Department of Medicine, Johns Hopkins University, Baltimore, MD; 119 - Department of Health Services, University of Washington, Seattle, WA; 120 - Kaiser Permanente Washington Health Research Institute, Seattle, WA; 121 - Division of Biostatistics,

Washington University in St. Louis, St. Louis, MO; 122 - Vanderbilt University Medical Center, Nashville, TN; 123 - Department of Biostatistics, Johns Hopkins Bloomberg School of Public Health, Baltimore, MD; 124 - University of Colorado at Denver, Denver, CO; 125 - Precision Medicine Center, Seoul National University Bundang Hospital, Seongnam, Republic of Korea; 126 - Macrogen Inc., Seoul, Republic of Korea; 127 - Gong Wu Genomic Medicine Institute, Seoul National University Bundang Hospital, Seongnam, Republic of Korea; 128 - Glenn Biggs Institute for Alzheimer's and Neurodegenerative Diseases, University of Texas Health Sciences Center at San Antonio, San Antonio, TX; 129 - Aflac Cancer and Blood Disorders Center of Children's Healthcare of Atlanta and Emory University Department of Pediatrics, Atlanta, GA USA; 130 - Taichung Veterans General Hospital Taiwan, Taichung City, Taiwan; 131 - Seattle Epidemiologic Research and Information Center, Department of Veterans Affairs Office of Research and Development, Seattle, WA; 132 - Survey Research Center, Institute for Social Research, University of Michigan, Ann Arbor, MI; 133 - Division of Epidemiology and Community Health, School of Public Health, University of Minnesota, Minneapolis, MN; 134 - Duke University, Durham, NC; 135 - University of Vermont, Burlington, VT; 136 - University of Southern California, Center for Genetic Epidemiology, Department of Preventive Medicine, Los Angeles, CA; 137 - Illumina Laboratory Services, Illumina, Inc., San Diego, CA; 138 - Department of Psychology, University of Minnesota, Minneapolis, MN; 139 - Department of Human Genetics, Graduate School of Public Health, University of Pittsburgh, Pittsburgh, PA; 140 - Department of Biostatistics, Graduate School of Public Health, University of Pittsburgh, Pittsburgh, PA; 141 - Department of Internal Medicine - Cardiology, University of Michigan, Ann Arbor, MI; 142 - Department of Human Genetics, University of Michigan, Ann Arbor, MI; 143 - Department of Epidemiology, University of Kentucky, Lexington, KY; 144 - Duke Molecular Physiology Institute, Duke University Medical Center, Durham, NC; 145 - University of Texas Health Science Center at Houston, Houston, TX; 146 - Baylor College of Medicine Human Genome Sequencing Center, Houston, TX; 147 - Northwest Genomics Center, Seattle, WA; 148 - Brotman Baty Institute, Seattle, WA; 149 - Department of Psychiatry, University of Michigan, Ann Arbor, MI; 150 - Department of Physiology and Biophysics, University of Mississippi Medical Center, Jackson, MS; 151 - Department of Human Genetics, McGill University, Montreal, Canada; 152 - Quantitative Biosciences Institute, University of California, San Francisco, CA; 153 - Institute for Human Genetics, University of California, San Francisco, CA; 154 - Bakar Computational Health Sciences Institute, University of California, San Francisco, CA.

* These authors contributed equally to this work
† https://www.nhlbiwgs.org/topmed-banner-authorship; See "Additional Authors from the NHLBI Trans-Omics for Precision Medicine Consortium" for full banner author list (excluding primary authors above)

Additional Authors from the NHLBI Trans-Omics for Precision Medicine Consortium

Namiko Abe[10], Laura Almasy[155], Seth Ament[156], Peter Anderson[157], Pramod Anugu[158], Deborah Applebaum-Bowden[159], Tim Assimes[160], Dimitrios Avramopoulos[26], Emily Barron-Casella[26], Terri Beaty[26], Gerald Beck[22], Diane Becker[26], Amber Beitelshees[156], Takis Benos[161], Marcos Bezerra[162], Joshua Bis[157], Russell Bowler[163], Ulrich Broeckel[164], Jai Broome[157], Karen Bunting[10], Carlos Bustamante[160], Erin Buth[157], Jonathan Cardwell[124], Vincent Carey[94], Cara Carty[165], Richard Casaburi[166], Peter Castaldi[94], Mark Chaffin[167], Christy Chang[156], Yi-Cheng Chang[168], Sameer Chavan[124], Bo-Juen Chen[10], Wei-Min Chen[169], Lee-Ming Chuang[168], Ren-Hua Chung[170], Suzy Comhair[22], Elaine Cornell[171], Carolyn Crandall[166], James Crapo[163], Jeffrey Curtis[172], Coleen Damcott[156], Sean David[173], Colleen Davis[157], Lisa de las Fuentes[174], Michael DeBaun[175], Ranjan Deka[176], Scott Devine[156], Qing Duan[177], Ravi Duggirala[178], Jon Peter Durda[171], Charles Eaton[179], Lynette Ekunwe[158], Adel El Boueiz[180], Serpil Erzurum[22], Charles Farber[169], Matthew Flickinger[172], Myriam Fornage[181], Chris Frazar[157], Mao Fu[156], Lucinda Fulton[174], Shanshan Gao[124], Yan Gao[158], Margery Gass[182], Bruce Gelb[15], Xiaoqi (Priscilla) Geng[172], Mark Geraci[183], Auyon Ghosh[94], Chris Gignoux[160], David Glahn[184], Da-Wei Gong[156], Harald Goring[185], Sharon Graw[186], Daniel Grine[124], C. Charles Gu[174], Yue Guan[156], Namrata Gupta[167], Jeff Haessler[182], Nicola L. Hawley[184], Ben Heavner[157], David Herrington[187], Craig Hersh[94], Bertha Hidalgo[20], James Hixson[181], Brian Hobbs[94], John Hokanson[124], Elliott Hong[156], Karin Hoth[188], Chao (Agnes) Hsiung[170], Yi-Jen Hung[189], Haley Huston[190], Chii Min Hwu[130], Rebecca Jackson[191], Deepti Jain[157], Min A Jhun[172], Craig Johnson[157], Rich Johnston[192], Kimberly Jones[26], Sekar Kathiresan[167], Alyna Khan[157], Wonji Kim[180], Greg Kinney[124], Holly Kramer[193], Christoph Lange[194], Ethan Lange[124], Leslie Lange[124], Cecelia Laurie[157], Meryl LeBoff[94], Jiwon Lee[94], Seunggeun Shawn Lee[172], Wen-Jane Lee[130], David Levine[157], Joshua Lewis[156], Xiaohui Li[195], Yun Li[177], Henry Lin[195], Honghuang Lin[196], Keng Han Lin[172], Simin Liu[179], Yongmei Liu[134], Yu Liu[197], James Luo[27], Michael Mahaney[198], Barry Make[26], JoAnn Manson[94], Lauren Margolin[167], Lisa Martin[199], Susan Mathai[124], Susanne May[157], Patrick McArdle[156], Merry-Lynn McDonald[20], Sean McFarland[200], Daniel McGoldrick[157], Caitlin McHugh[157], Hao Mei[158], Luisa Mestroni[186], Nancy Min[158], Ryan L Minster[161], Matt Moll[94], Arden Moscati[15], Solomon Musani[158], Stanford Mwasongwe[158], Josyf C Mychaleckyj[169], Girish Nadkarni[15], Rakhi Naik[26], Take Naseri[201], Sergei Nekhai[202], Bonnie Neltner[124], Heather Ochs-Balcom[203], David Paik[160], James Pankow[204], Afshin Parsa[156], Juan Manuel Peralta[178], Marco Perez[160], James Perry[156], Ulrike Peters[182], Lawrence S Phillips[192], Toni Pollin[156], Julia Powers Becker[124], Meher Preethi Boorgula[124], Michael Preuss[15], Dandi Qiao[94], Zhaohui Qin[192], Nicholas Rafaels[124], Laura Raffield[177], Laura Rasmussen-Torvik[205], Aakrosh Ratan[169], Robert Reed[156], Elizabeth Regan[163], Muagututi‘a Sefuiva Reupena[206], Carolina Roselli[167], Pamela Russell[124], Sarah Ruuska[190], Kathleen Ryan[156], Ester Cerdeira Sabino[207], Danish Saleheen[208], Shabnam Salimi[156], Steven Salzberg[26], Kevin Sandow[195], Vijay G. Sankaran[209], Christopher Scheller[172], Ellen Schmidt[172], Karen Schwander[174], Frank Sciurba[161], Christine Seidman[45], Jonathan Seidman[45], Stephanie L. Sherman[192], Aniket Shetty[124], Wayne Hui-Heng Sheu[130], Brian Silver[210], Josh Smith[157], Tanja Smith[10], Sylvia Smoller[83], Beverly Snively[187], Michael Snyder[160], Tamar Sofer[94], Garrett Storm[124], Elizabeth Streeten[156], Yun Ju Sung[174], Jody Sylvia[94],

Adam Szpiro[157], Carole Sztalryd[156], Hua Tang[160], Margaret Taub[26], Matthew Taylor[124], Simeon Taylor[156], Machiko Threlkeld[157], Lesley Tinker[182], David Tirschwell[157], Sarah Tishkoff[211], Hemant Tiwari[20], Catherine Tong[157], Michael Tsai[204], Dhananjay Vaidya[26], Peter VandeHaar[172], Tarik Walker[124], Robert Wallace[188], Avram Walts[124], Fei Fei Wang[157], Heming Wang[94], Karol Watson[166], Jennifer Wessel[183], Kayleen Williams[157], L. Keoki Williams[212], Carla Wilson[94], Joseph Wu[160], Huichun Xu[156], Lisa Yanek[26], Ivana Yang[124], Rongze Yang[156], Norann Zaghloul[156], Maryam Zekavat[167], Snow Xueyan Zhao[163], Wei Zhao[172], Degui Zhi[181], Xiang Zhou[172], Xiaofeng Zhu[213]

155 - Children's Hospital of Philadelphia, Philadelphia, PA; 156 - University of Maryland, Baltimore, MD; 157 - University of Washington, Seattle, WA; 158 - University of Mississippi, Jackson, MS; 159 - National Institutes of Health, Bethesda, MD; 160 - Stanford University, Stanford, CA; 161 - University of Pittsburgh, Pittsburgh, PA; 162 - Fundação de Hematologia e Hemoterapia de Pernambuco - Hemope, Recife, Brazil; 163 - National Jewish Health, Denver, CO; 164 - Medical College of Wisconsin, Milwaukee, WI; 165 - Washington State University, Seattle, WA; 166 - University of California, Los Angeles, Los Angeles, CA; 167 - Broad Institute, Cambridge, MA; 168 - National Taiwan University, Taipei, Taiwan; 169 - University of Virginia, Charlottesville, VA; 170 - National Health Research Institute Taiwan, Miaoli County, Taiwan; 171 - University of Vermont, Burlington, VT; 172 - University of Michigan, Ann Arbor, MI; 173 - University of Chicago, Chicago, IL; 174 - Washington University in St Louis, St Louis, MO; 175 - Vanderbilt University, Nashville, TN; 176 - University of Cincinnati, Cincinnati, OH; 177 - University of North Carolina, Chapel Hill, NC; 178 - University of Texas Rio Grande Valley School of Medicine, Edinburg, TX; 179 - Brown University, Providence, RI; 180 - Harvard University, Boston, MA; 181 - University of Texas Health at Houston, Houston, TX; 182 - Fred Hutchinson Cancer Research Center, Seattle, WA; 183 - Indiana University, Indianapolis, IN; 184 - Yale University, New Haven, CT; 185 - University of Texas Rio Grande Valley School of Medicine, San Antonio, TX; 186 - University of Colorado Anschutz Medical Campus, Aurora, CO; 187 - Wake Forest Baptist Health, Winston-Salem, NC; 188 - University of Iowa, Iowa City, IA; 189 - Tri-Service General Hospital National Defense Medical Center, Taipei, Taiwan; 190 - Blood Works Northwest, Seattle, WA; 191 - Ohio State University Wexner Medical Center, Columbus, OH; 192 - Emory University, Atlanta, GA; 193 - Loyola University, Maywood, IL; 194 - Harvard School of Public Health, Boston, MA; 195 - Lundquist Institute, Torrance, CA; 196 - Boston University, Boston, MA; 197 - Stanford University, Palo Alto, CA; 198 - University of Texas Rio Grande Valley School of Medicine, Brownsville, TX; 199 - George Washington University, Washington, DC; 200 - Harvard University, Cambridge, MA; 201 - Ministry of Health, Government of Samoa, Apia, Samoa; 202 - Howard University, Washington, DC; 203 - University at Buffalo, Buffalo, NY; 204 - University of Minnesota, Minneapolis, MN; 205 - Northwestern University, Chicago, IL; 206 - Lutia I Puava Ae Mapu I Fagalele, Apia, Samoa; 207 - Universidade de Sao Paulo, Sao Paulo, Brazil; 208 - Columbia University, New York, NY; 209 - Broad Institute, Harvard University, Boston, MA; 210 - UMass Memorial Medical Center,

Worcester, MA; 211 - University of Pennsylvania, Philadelphia, PA; 212 - Henry Ford Health System, Detroit, MI; 213 - Case Western Reserve University, Cleveland, OH

Table of Contents

1 Supplementary information 9
1.1 TOPMed program description 9
1.1.1 Organizational components 9
1.1.2 Study designs 10
1.1.3 Phenotypic data 11
1.1.4 Participant diversity 13
1.1.5 Multi-omics assays 14
1.1.6 Resources available 15
1.2 Batch effects 16
1.2.1 Principal components analysis 16
1.2.2 Duplicate sample concordance 17
1.2.3 Association tests for batch effects 20
1.3 Comparison to prior WGS and WES data 23
1.3.1 Comparison to high coverage WES in BioMe Study 23
1.3.2 Comparison to low coverage WGS and high coverage WES in Framingham Heart Study 23
1.4 Comparing to GATK variant calling pipeline 27
1.5 Whole genome sequencing accessibility mask 28
1.6 On the distribution of inter-singleton distances in a sample 28
1.7 Novel genetic variants in unmapped reads 30
1.8 Site frequency spectrum 31
1.9 Admixture 32
1.10 Demographic estimation under selection at linked sites 33
1.10.1 Sample selection 33
1.10.2 Site filtering/ascertainment 33
1.10.3 Demographic inference 35
1.11 Selection 36

2 Study acknowledgments 38

3 Other acknowledgments 49

4 Ethics statement 53

5 References 57

1 Supplementary information

1.1 TOPMed program description

Long-term goals of the TOPMed program include (1) characterizing the genetic architecture of phenotypic variation in heart, lung, blood, and sleep (HLBS) disorders and related phenotypes; (2) identifying causal genetic variants and assessing how they may interact with environmental factors; (3) characterizing the spectrum of disease types; (4) understanding differences in these disorders across diverse populations and whether those differences are attributable to genetic or non-genetic factors; and (5) establishing a foundation for personalized interventions for disease prediction, prevention, diagnosis, and treatment. We are pursuing these goals through the generation of WGS and “omics” data for research participants of diverse backgrounds and with deep phenotypic characterization through ongoing studies, and by developing analytical tools to effectively mine the resulting data.

1.1.1 Organizational components

TOPMed has several organizational components: the NHLBI program office, an External Advisory Panel, an Informatics Research Center (IRC), a Data Coordinating Center (DCC), NHLBI’s Centralized Omics REsource (CORE) which funded several Omics Centers (Supplementary Table 17), several internal committees (including Executive; Steering; Analysis; Publications; and Ethical, Legal, and Social Implications (ELSI)), 32 investigator-led Working Groups, and >80 participating studies. The IRC (Gonçalo Abecasis, Principal Investigator) is located in the Biostatistics Department at the University of Michigan; it has primary responsibility for WGS data processing, including read alignments harmonized across sequencing centers, variant discovery, and genotype calling. The DCC (Ken Rice, Principal Investigator) is located in the Biostatistics Department at the University of Washington; it has primary responsibility for coordinating data flow and activities within the TOPMed program, as well as coordination with external entities, such as the NHLBI program office, dbGaP, and other genomics programs. Both the IRC and DCC perform quality control, develop analytical methods, perform analyses, and advise investigators on analytical issues.

The Working Groups are each focused on a particular phenotypic area such as atherosclerosis or asthma, or other general scientific areas such as analysis methods or population genetics. Much of the program’s scientific work is performed in these Working Groups where TOPMed investigators collaborate on designing, performing, and reporting multi-study analyses for publication. TOPMed data are being shared with the general scientific community through periodic releases on dbGaP, after completion of quality control and dbGaP curation (in total, approximately 6 to 12 months after release to study investigators).

A key priority for the TOPMed program is the development of methodology and software for WGS data in genotype-phenotype association and other types of analyses. Therefore, several methods development projects[1] are being supported in areas such as omics data integration and annotation, pedigree based analyses, machine learning, and high performance statistical modeling with a focus on heart, lung, blood, and sleep (HLBS) diseases and risk factors. TOPMed groups have been supported to develop and maintain association analysis pipelines based on the GENESIS[2] and EPACTS[3] statistical software suites. Furthermore, training of young investigators has been supported by periodic analysis workshops[4] focused on TOPMed data.

1.1.2 Study designs

The TOPMed program consists of multiple “Projects”, composed of one or more “Parent” studies, each of which had previously recruited participants and obtained consent, phenotypic data, and biosamples. Projects were considered for inclusion in TOPMed based largely on peer-reviewed responses to an NHLBI Funding Opportunity Announcement[5] for X01 applications. Study selection criteria included ancestral and ethnic diversity; richness of HLBS phenotypes and environmental risk factors; and design features facilitating the detection of rare variant effects. Within each study, participants were selected for WGS according to similar criteria, described more specifically in the TOPMed dbGaP accession for each study. In all cases, participants provided informed consent for genetic studies and data sharing with the scientific community via controlled access.

TOPMed includes multiple study designs with different strengths: (1) prospective cohorts provide broad phenotypic characterization and enable analyses of incident disease, longevity, and mortality; (2) cross-sectional or case-control studies increase precision in comparison of risk factors between cases and controls; (3) families and population isolates facilitate the study of rare variants by enrichment of private variants[6–8]; (4) family-based designs provide robustness to population structure[9]; and (5) case-only studies optimize analyses of disease severity and response to treatment.

Extended Data Tables 1 and 2 provide summaries of Projects and Parent studies contributing samples to the Freeze 5 data set, which includes the 53,831 samples described in the Main Text. All TOPMed Projects and Parent studies are described on the TOPMed website[10]. The following examples illustrate some of the variety in the design of TOPMed Projects and their component studies.

1. “The Jackson Heart Study” (JHS; Adolfo Correa, PI) Project derives from a single Parent study of African Americans, with a prospective cohort design and extensive clinical characterization across many different phenotypic areas.

2. “The Barbados Asthma Genetics Study” (BAGS; Kathleen Barnes, PI) Project derives from a single family-based genetic Parent study focused on asthma. Pediatric probands with asthma were initially recruited through local clinics, followed by recruitment of parents and other family members, and expansion to independent asthma cases and controls.
3. The “Atrial Fibrillation Genetics Consortium” (Patrick Ellinor, PI) Project is a consortium of several Parent studies of largely European ancestry, each contributing early-onset atrial fibrillation cases. Controls were derived from other TOPMed Projects.

1.1.3 Phenotypic data

Parent studies have measured thousands of phenotypic and environmental risk factors. The phenotypic foci of the current studies include heart (37%), lung (33%), blood (11%), or sleep phenotypes (1%), while the remaining 18% are cohort studies with many HLBS phenotypes (Supplementary Figure 1; Extended Data Table 2). There is considerable overlap in phenotypes among studies. For example, many lung-focused studies have heart phenotypes and vice versa. Many of the studies also have a wide range of other biomedical phenotypes including diabetes, kidney disease, and osteoporosis.

The depth of phenotypic characterization is illustrated by data descriptions for seven longitudinal cohort studies participating in TOPMed (Supplementary Table 22). Questionnaires cover sociodemographics; individual and familial medical histories; psychosocial factors; tobacco and alcohol use; diet and physical activity; and exposures such as air pollution and sunlight. Medical testing and monitoring include electrocardiography, spirometry, oral glucose tolerance, vision, hearing, cognitive function, polysomnography, and various measures of exercise. Laboratory tests include blood cell counts and blood chemistry measures (such as inflammatory markers, metabolites, hormones, oxidative stress markers, coagulation factors and many other biomarkers); and urine chemistry includes albumin, creatinine and other metabolites. Imaging studies include computerized tomography scans (aortic, coronary, lung, abdominal); echocardiography; magnetic resonance imaging (brain, cardiac, carotid, coronary wall); carotid ultrasound; bone densitometry; and retinal photography. Events adjudicated through medical records include coronary heart disease, heart failure, stroke, and venous thromboembolism; atrial fibrillation and cause of death are also ascertained. In most cases, time-to-event from study enrollment was also recorded.

Common phenotypic measures across TOPMed Parent studies provide opportunities for cross-study analyses to gain power in detecting genetic effects. However, these studies differ in how their phenotypic data were collected, annotated, and structured. Creating harmonized phenotypic data sets for cross-study analyses is therefore a challenging and largely manual process.

Many TOPMed Working Groups harmonize phenotypes within their focus area. Generally, the writing group for a given paper develops an analysis plan that defines the primary outcome and any necessary covariates. Subsequently, selected Parent studies each contribute source data and modify or transform it to fit these definitions. The individual-level data are then pooled and evaluated for homogeneity across Parent studies.

The TOPMed Data Coordinating Center (DCC) also harmonized phenotypes centrally, in collaboration with phenotype-domain experts in the Working Groups and data managers from the Parent studies. The DCC process used study data from existing dbGaP accessions to enable provenance-tracking and reproducibility by the scientific community, and includes the following steps:

1. Precisely define the harmonized phenotype concept.
2. Identify dbGaP phenotype variables in each participating study that appear to fit the definition (with or without modification).
3. Determine which of these dbGaP variables are sufficiently equivalent to allow a meaningful harmonization[11].
4. Perform quality control of the selected dbGaP variables.
5. Define and implement algorithms to modify or transform the dbGaP variables as needed to produce a harmonized version.
6. Perform quality control on the harmonized variables across multiple studies to evaluate homogeneity, and refine previous steps as needed.
7. Document the process to ensure full reproducibility.
8. Submit harmonized phenotypes with documentation to NHLBI-designated data repositories for distribution to the scientific community.

Depending on the availability of study phenotypes, their heterogeneity, and the results of data QC, this process is often iterative before a harmonized phenotype is finalized. The DCC process is described by Stilp *et al.* (2020) [12].

The DCC-harmonized data for an initial set of frequently-used phenotypes have been submitted to dbGaP and to BioData Catalyst for release to the scientific community; they will be labeled as “TOPMed-DCC harmonized” to distinguish them from the unharmonized source data. Harmonized variables currently include common covariates (subcohort, sex, race, ethnicity, recruitment site, height, weight, BMI, and smoking behavior), blood cell counts, lipids in blood, blood pressure, atherosclerosis measurements, and venous thromboembolism case status. Age at measurement is paired with each variable, except for basic demographics.

In addition, the TOPMed Lung Working Groups are utilizing an extensive lung phenotype harmonization project by the NHLBI Pooled Cohorts Study for lung function[13].

1.1.4 Participant diversity

As of 2009, approximately 96% of participants in genome-wide association studies (GWAS) were of primarily European ancestry; by 2016, this figure was 80% mainly due to an increase in the study of East Asian populations, but the number of GWAS studies involving participants of African ancestry, Amerindigenous ancestry, and Hispanic or Latino ethnicity had not substantially increased[14]. Thus, in TOPMed, a concerted effort is being made to create a resource well-suited for investigating health issues affecting diverse populations. The populations sampled in Parent studies are mainly from the United States, although ~18% are from non-U.S. locations, including Brazil, Samoa, Costa Rica, Barbados, China, Taiwan, Pakistan, and multiple countries in Africa and Europe. TOPMed also includes studies in founder populations, such as Amish and Samoans.

Race, ethnicity, and ancestry information was obtained from participant questionnaires and/or study inclusion criteria. While the participants that have contributed to TOPMed represent a broad continuum of culturally, phenotypically, and genetically diverse populations, some analyses required individuals to be consolidated across studies into ‘population groups’, which varied in composition according to the goals of the analysis. The DCC created guidelines[15] on the use and reporting of race, ethnicity, and ancestry in the TOPMed program that downstream users of TOPMed data are also encouraged to consider. The entire current set of ~155,000 selected participants consists of approximately 41% European ancestry (European, European American), 31% African ancestry (African, African American, African Caribbean), 15% Hispanic/Latino (including Mexican, Mexican American, Central American, South American, Cuban, Dominican, Puerto Rican), 9% Asian ancestry (Chinese, Taiwanese, Asian American, Pakistani) and 4% ‘Other’ (Samoan, Native American, multiple groups, or unknown) (Supplementary Figure 2). For the Freeze 5 genotype call set, sample numbers by study are provided for five broad population groups and for sex in Supplementary Figures 39 and 40, respectively. In Freeze 5, the overall percentage of females is 60%.

Population structure and relatedness were evaluated in order to account for their effects in association studies, as well as to detect pedigree errors. We estimated principal components of genotypic data in the Freeze 5 call set using PC-AiR[16] and PC-Relate[17] (implemented in the GENESIS[2] software package), which distinguish close relatedness from more distant relatedness due to population structure, while accounting for admixture. Extended Data Figure 1a shows the expected separation among the major continental ancestral groups. Extended Data Figure 1b shows differentiation among subgroups, including differences between participants in the Old Order Amish study and other European ancestry groups (PC6), and between participants in the Costa Rican Asthma study and other Central Americans (see also Supplementary Figure 41). Kinship coefficient estimates show that a majority of Parent studies have substantial numbers of first and second degree relatives (Supplementary Figures 42 and

43). A small number of individuals were participants in more than one TOPMed study, resulting in cross-study duplicates and some cross-study relatives (see the Circos[18] plot in Supplementary Figure 43).

1.1.5 Multi-omics assays

The TOPMed program is adding multi-omic assays to samples from participants with sequenced genomes. Initially, a multi-omics pilot study was performed on ~2,000 blood samples from ~1,000 participants in the Multi-Ethnic Study of Atherosclerosis (MESA), an on-going cohort study with extensive longitudinal phenotype data. The pilot measured DNA methylation, RNA-seq, metabolomics, and proteomics, using technologies described below. Subsequently, TOPMed initiated assays of DNA methylation on ~48,500 samples, RNA-seq on ~33,000 samples, metabolomics on ~16,000 samples and proteomics on ~950 samples. These samples derive from 19 different studies and multiple tissue types, including blood, nasal, lung, and cardiovascular tissues, as well as participant-specific, induced pluripotent stem cells. These TOPMed omics data are scheduled for release on dbGaP as early as mid-2020. Several TOPMed Parent studies have omics data sets collected outside of the TOPMed program, some of which are currently available on dbGaP (e.g. the Framingham Heart Study; see “Molecular Data” under Parent study accession numbers in Extended Data Table 2).

TOPMed performed RNA-seq, proteomic, metabolomic, and methylation assays in a pilot project, using blood samples from each of ~1,000 participants in the Multi-Ethnic Study of Atherosclerosis (MESA). One goal of the project was to assess changes in omics values over time, in conjunction with changes in biomedical phenotypes. To this end, the pilot included peripheral blood mononuclear cells (PBMCs), plasma, and DNA samples drawn at each of two time points approximately 10 years apart, Exam 1 (2000-2002) and Exam 5 (2010-2012), from each participant. Each omics assay type was performed on largely the same set of paired samples from participants whose DNA had already been whole genome sequenced. Multiple quality control features were included in each assay to evaluate reproducibility and batch effects. The data from this pilot project are scheduled for release on dbGaP in 2020.

The following assay methods were used:

1. RNA-seq: PolyA+ mRNA libraries were prepared from PBMCs (Illumina TruSeq™) from the ~1,000 pairs of blood samples, and sequenced on Illumina HiSeq 4000 with target coverage of >40M reads at two genomic centers. In addition, RNA-seq was performed on cell-sorted CD19+ monocytes and on CD4+ T cells from ~400 participants obtained at Exam 5. The assays were performed and the resulting data processed at two different genomic centers using a harmonized pipeline[19].
2. Metabolomics: Three methods were used for assaying metabolites in plasma samples, each based on chromatography and mass spectroscopy. These methods included

non-targeted lipids (228 known and 2,662 unknown), non-targeted polar metabolites (253 known and 3,966 unknown) and 84 targeted central metabolites.

3. Proteomics: DNA aptamer technology[20] (SOMAscan®[21]) was used to assay more than 1,300 proteins in plasma samples. In this technology, single-stranded DNAs composed of modified nucleotides are selected from libraries based on affinity to specific proteins. These reagents capture proteins in a complex mixture and their concentrations are estimated by DNA detection technology.
4. Methylation: Microarray technology was used to assay methylation at CpG sites in genomic DNA samples. The assays were performed using the Illumina EPIC array[22], which targets over 850,000 CpG sites in the genome. All DNA samples were treated with bisulfite to assay a combination of 5-methylcytosine (5-mC) and 5-hydroxymethylcytosine (5-hmC), and ~200 of these matched samples were also treated with an oxidizing reagent to distinguish between 5-mC and 5-hmC.

Another pilot study was carried out in 2017 using 950 plasma samples from Generation 3 participants in the Framingham Heart Study. These samples were used for proteomic profiling using the SOMAscan® aptamer technology described above.

Supplementary Table 17 provides information about the omics centers that performed assays for these pilot studies. The same centers have been providing assays for omics assays in 2018-2020, using the same basic technologies described above.

1.1.6 Resources available

Following quality control and dbGaP curation, TOPMed data are available in dbGaP accessions with accompanying study documents that describe the methods of phenotypic and omics data collection. WGS data include read alignments, genotype call sets, and related quality metrics. Methods of sequence data acquisition and processing are provided for each data Freeze. Metadata also include linking of DNA sample and participant identifiers; DNA sample attributes; participant consent group; and, for many studies, pedigree structures. A detailed guide to finding and using TOPMed data in dbGaP accessions is provided on the TOPMed website[23].

The TOPMed variant browser (BRAVO[24]) provides the chromosome location, alleles, TOPMed-wide allelic frequencies, and other characteristics of all single nucleotide or short indel variants called in the current data Freeze. A detail page for each variant shows the distributions of sequencing depth and genotype quality scores for carriers and non-carriers of the non-reference allele, as well as QC metrics used for site-level filtering. A variant site list deposited in NCBI dbSNP is available for download[25].

The TOPMed imputation reference panel is publicly available for imputation of user-supplied samples through the NHLBI BioData Catalyst[26] at TOPMed Imputation Server[27]. Genotypic data

for the reference panel can be obtained through application to dbGaP, as detailed in Box 1. As noted in the Main Text, the TOPMed panel is a significant improvement upon existing panels such as 1000 Genomes[28] and the Haplotype Reference Consortium[29] because of the high quality of TOPMed WGS data, the genetic diversity in its samples, and the large sample size. In particular, these improvements allow more accurate imputation of low frequency variants, which will significantly leverage the value of sample sets with array-based genotypes.

The TOPMed Ethical, Legal, and Social Implications (ELSI) committee moderates discussion of matters regarding access to and use of TOPMed data. The committee has considered consent, participant privacy, and implementation issues regarding: (1) public sharing of variant summary data in the TOPMed variant server; (2) public access to use of a TOPMed reference panel in a genotype imputation server (without sharing individual-level data); and (3) return of results to study participants. Summary reports on the TOPMed website[30] contain points for study investigators to consider in making decisions about each issue.

1.2 Batch effects

TOPMed WGS was performed over multiple studies, years and sequencing centers, introducing the possibility of batch effects in the sequence data. To reduce this possibility, laboratory and data processing methods were standardized as much as possible and genotype calling was performed jointly across all available samples (see Methods). The following three approaches were used to investigate the possibility of batch effects on genotype calls.

1.2.1 Principal components analysis

PCA was performed to explore possible batch effects related to sequencing center. As noted above, in TOPMed, there is a high degree of confounding among studies, ancestries and centers. In most cases, a given study was sequenced at only one center, and studies tend to differ in ancestral composition. Therefore, genetic differences among centers revealed by PCA may be due to this confounding with actual ancestry differences by study. However, lack of center differences within a given population group may indicate that batch effects due to center are minimal.

For PCA, we selected samples with annotated race/ethnicity in three different population groups: African American (17 studies, 9,337 subjects), European American (18 studies, 22,668 subjects), and Mexican American (4 studies, 1,371 subjects). Initially, each of the three groups had small numbers of outlier samples in PC space. These outliers were removed using the Mahalanobis distance[31] computed from the first four PCs within each group; samples with a Mahalanobis distance greater than the $\alpha = 0.01$ critical value (13.28 with 4 d.f.) were excluded from the following analyses. The three population groups were selected because they each

included a substantial number of samples sequenced at more than one center and included multiple studies.

Supplementary Figures 3, 4, and 5 show that centers have very similar PC distributions within European American or African American sample sets, suggesting minor (if any) center effects. More differentiation is observed among centers in the Mexican American group, for which the Illumina and NYGC centers each represent a single study, and the Broad center represents two studies. These same centers do not show notable PC differences in the European and African American groups (Supplementary Figure 5. Therefore, it is likely that the center differences for Mexican American subjects are due to confounding with study, given that studies may have recruited subjects with ancestries from different parts of Mexico.

1.2.2 Duplicate sample concordance

Two types of samples were sequenced in duplicate, providing opportunities to assess the repeatability of sequencing results within centers, between centers and over time:

1) HapMap subjects NA12878 (CEU) and NA19238 (YRI) were sequenced at each of the sequencing centers in alternation, approximately once every 1,000 study samples throughout both Phases 1 and 2. Each center used aliquots from the same lot of DNA extracted from cell culture (Lot K6 for NA12878 and LotE2 for NA19238). The number of sequencing instances for NA12878 was 32 and for NA19238 was 36, resulting in 1,126 pairwise comparisons (292 within-center and 834 between-center).
2) DNA samples from a number of study participants were sequenced more than once (usually just two times). These duplicates were largely unplanned; some were due to duplicate submission from a single study and others were from the same individual enrolled in two different studies. Among a total of 368 pairs of duplicates (each from a different study participant), 234 were sequenced at the same center and 134 at different centers. DNA for study samples was extracted from blood by the contributing studies.

We tested genotype concordance between all duplicate sample pairs across variants for which at least one member of the pair had an alternate allele call, regardless of read depth (i.e. alternate allele concordance). The SeqVarTools[32] R package was used for concordance calculations. Concordance was computed separately for all SNVs, singleton (i.e. variants with a single alternate allele across all unrelated participants) SNVs and all INDELs, and for variants passing and failing the Support Vector Machine (SVM) quality filter. Singleton indels are too rare for meaningful estimation of duplicate concordance; each duplicate pair has a median number of just one singleton indel. For variants of all frequencies, the mean concordance (SE) was 0.99951 (0.00002) for passing SNVs, 0.661 (0.002) for failing SNVs, 0.9930 (0.0002) for passing indels, and 0.756 (0.002) for failing indels. Among singletons, the mean concordance (SE) was 0.9966 (0.0002) for passing SNVs and 0.659 (0.006) for failing SNVs. In the subsequent analyses described below we present concordance calculated only from variants that passed the SVM quality filter.

We first describe concordance variation among centers by analyzing duplicate pairs for 234 participants in which both samples in each pair were sequenced at the same center (i.e., 'within-center' pair). Supplementary Table 23 shows the mean concordance values for each of the four centers having within-center duplicates (labeled here as C1, C3, C5 and C6) and Supplementary Figure 6 shows the center-specific distributions. Concordance for all SNVs is notably higher than for singleton SNVs and indels, while the latter two are similar in magnitude (0.99954 (0.00002) all SNVs, 0.9972 (0.0002) singleton SNVs, 0.9933 (0.0002) all indels). Although concordance values are high for all centers, there appears to be some variation among centers. Sequence data processing was centralized and genotype calling was performed jointly across all centers and studies, so these factors should not contribute to the apparent differences. However, variation associated with centers could be due to variation in laboratory processes, quality of DNA submitted by different studies and/or the ancestry of samples allocated to each center (since allele frequency distributions may vary by ancestry).

There is a high level of confounding among these sources of variation for study samples. For example, 18 of 19 participants from Asian population groups were sequenced at center C5; all of the 29 Mexican American participants were sequenced at center C6; 36 of the 42 European American participants were sequenced at center C3; and 107 of the 133 African American or African Caribbean participants were sequenced at center C1. There is also a high level of confounding of studies with centers. For example, 18 of the 19 participants from two studies were sequenced exclusively at center C5 and 99 of the 112 participants sequenced at center C1 are from one pair of studies (due to individuals enrolling in both studies).

We analyzed the concordance data for the study samples using a linear mixed model including center and the first four principal components of the genotypes (to represent ancestry) as fixed effects and study (or study pair) as a random intercept. In this analysis, an F-test of the center effect has a p-value <0.0001 for all SNVs, singleton SNVs and all INDEL concordances (d.f. 3/199, 3/201, and 3/195, respectively). However, the magnitudes of the center effects are small. Among all pairwise comparisons of the four centers, only one (C3-C1) is significant (p<0.0001) for both SNVs and INDELs, while none are significant for singleton SNVs (multiple comparison adjustment using Tukey method). The 95% confidence interval for the estimated size of this effect on concordance is 0.00043 +/- 0.00018 for all SNVs, 0.00082 +/- 0.00125 for singleton SNVs, and 0.00666 +/- 0.00158 for all INDELs. We consider these to be "center-associated" effects, rather than effects necessarily due to specific center activities because, although the model fit appears to be adequate (e.g. from examination of residual plots, not shown), it may not completely account for the confounding factors (i.e. ancestry and study), due to high collinearity.

Confounding of center with ancestry and DNA quality is not a factor for the HapMap samples. The total number of sequencing instances was 32 for the CEU sample and 36 for the YRI sample. Considering unique pairs of sequencing instances, the sample sizes for within-center comparisons for centers C1-C6 are N=(2,2,7,0,3,0) for CEU and N=(2,3,7,1,3,0) for YRI. These numbers are too small for parametric statistical tests. However, we compared the order of mean concordance values for the three centers having within-center duplicates for study samples (Supplementary Table 23), as well as for the two HapMap controls (Supplementary

Table 24). For all SNVs, the order of concordance is C5 > C3 > C1 for all three sample sets, an observation with a probability of 0.03. For all INDELs, the orders are the same for the study samples and YRI, but CEU has C5 > C1 > C3. In any case, the differences are very small (e.g. 0.00003 for SNVs and 0.00017 for INDELS for C3 - C1).

Whether differences in center concordance, when they occur, can lead to batch effects on genotype calls depends on whether errors tend to be systematic (i.e., leading to consistent errors at consistent sites within a center) or whether the errors are random. Examination of between-center genotype concordance provides some insight into this issue.

Consider a simple model of random sequencing errors for a haploid genome. Assume that each site in the genome has the same probability of error per site per sequencing instance, and that the error rates for centers 1 and 2 are e_1 and e_2, respectively. The probabilities of discordance at any given site are the following for comparisons within center 1, between centers 1 and 2, and within center 2:

$$2e_1(1-e_1) \; > \; e_1(1-e_2) + e_2(1-e_1) \; > \; 2e_2(1-e_2), \text{ when } e_2 < e_1 < ½$$

This relationship indicates that between-center discordance will be intermediate between the two within-center discordances when errors are random (and, of course, also for concordance). When errors are systematic, we might expect to see that the within-center concordances are both greater than the between-center concordance.

Supplementary Figure 7 compares distributions of concordance of all SNVs and all INDELS for the three pairings of the three centers (C1, C3, C5) that each have more than two within- and between-center observations. These comparisons involve sequences from study participants, and all observations are independent - i.e. each participant is represented by just one pair of sequencing instances. The results for all 6 center pairs show that the mean values for the between-center concordance are intermediate between the two within-center values (as are the mean values for singleton SNVs, data not shown). Supplementary Figure 8 compares distributions of concordance for the HapMap samples. In this case, all possible pairs of each sequencing instance are shown, both between- and within- centers, so they are not independent observations. These data also show that between-center concordances are intermediate.

We conclude that evidence for differences in concordance rates due to center-specific factors (rather than confounded factors) is suggestive, but not compelling. Furthermore, comparisons of within- versus between-center concordances are qualitatively consistent with a simple random error model. Under this model, center differences would contribute noise, rather than systematic bias (i.e., batch effects), to association tests.

Supplementary Figure 44 examines the relationship between concordance rate and the time span between the sequencing of the two members of a duplicate sample pair. If laboratory conditions and/or data processing change over time, we might expect to see a decline in concordance as the time difference increases. Although there is some evidence for a slight decline in concordance (especially for indels), the relationship is not strong. We do not provide

statistical tests for these comparisons of concordance rates because of a lack of independence in the data (i.e. the same sample may belong to multiple duplicate pairs).

1.2.3 Association tests for batch effects

To identify variants exhibiting possible batch effects, we performed GWAS using potential batching variables as binary outcomes. We analyzed a Freeze 5 study with two batches of samples sequenced at different sequencing centers and in different Phases of TOPMed. To avoid potential spurious signals due to ancestry effects, we performed this analysis separately within the African American and European American subgroups of that study (after removing outliers defined by Mahalanobis distance, as described in the PCA section above). The African American association analysis included 753 samples sequenced at one sequencing center in Phase 1 and 1942 samples sequenced at another sequencing center in Phase 2. The European American association analysis used 828 samples sequenced at one sequencing center in phase 1 and 4451 samples sequenced at another sequencing center in phase 2.

For each population group analysis, we used GENESIS[33] to fit a logistic regression model under the null hypothesis of no genetic association, with sequencing center (equivalent to TOPMed Phase) as the outcome, and sex as a fixed effect covariate. All variants passing the quality filter with a minor allele count (MAC) $\geq$ 20 in the sample set were tested for single variant association using score tests. All rare variants passing the quality filter with minor allele frequency (MAF) ≤ 1% in the sample set were tested for aggregate variant association using both burden and SKAT tests in 50kb sliding windows shifted by 20kb increments.

Manhattan plots for the association results of each analysis are presented in Supplementary Figures 9 and 10. A very small percentage of variants that pass the variant quality filter show association signals with sequencing center (equivalently, TOPMed phase) in the single variant analyses; 756 of 20,514,262 variants (0.0037%) in the African American analysis, and 485 of 12,322,201 variants (0.0039%) in the European American analysis, were identified as genome-wide significant (i.e. $p < 5\times10^{-8}$). The most significant variants had strong signals with $p < 1\times10^{-100}$. We observe enrichment for indels among these significant variants; 82.8% (Afr) and 74.4% (Eur) of significant variants are indels, as compared to the 6.3% (Afr) and 5.9% (Eur) indels across all variants tested. We also observe enrichment for seemingly common variants; 93.0% (Afr) and 74.0% (Eur) of significant variants have minor allele frequency (MAF) > 5%, as compared to 41.8% (Afr) and 49.8% (Eur) of all variants tested. None of the rare variant aggregation units have significant burden or SKAT associations after Bonferroni correction for the number of sliding windows tested.

We also performed single variant association analyses with each of the population groups using a “null” binary phenotype generated by randomly shuffling the batch labels, keeping the same “case” to “control” ratio as in each batch effect association analysis. Using this null phenotype,

no variants in the African American analysis, and only 4 variants in the European American analysis (minimum $p = 2.2 \times 10^{-9}$), reached genome-wide significance. This result illustrates that the spurious associations observed when using batch labels as the outcome variable are not driven by poor model performance, but rather likely by genotyping artifacts related to batch.

Examination of the batch-associated variants shows relatively little overlap between different TOPMed sample sets. For example, there were 8,523,038 overlapping variants tested in both population group analyses presented here. However, while 722 of the 756 significant variants from the African American analysis were tested in the European American analysis, and 480 of the 485 significant variants from the European American analysis were tested in the African American analysis, only 169 variants were significant in both analyses. We have observed similar results for other studies and sequencing centers in later data Freezes (data not shown). These observations suggest that it may be difficult to identify a comprehensive set of variants that are subject to batch effects in general.

Given that the batch-associated variants are mostly common variants in these TOPMed sample sets, we looked up their allele frequencies in the 1000 Genomes Phase 3 and UK10K datasets using the TOPMed freeze 8 WGSA Google BigQuery annotation database on the Biodata Catalyst powered by Seven Bridges platform[26]. This annotation database was built using variant annotations gathered by Whole Genome Sequence Annotator (WGSA) v0.8[34] and formatted by WGSAParsr v6.3.8[35]. The majority of the batch-associated variants are observed in these external datasets. Of the 485 variants identified in the European American analysis, 76% were observed in 1000 Genomes (alternate allele frequency correlation: AAF r = 0.88) and 73% were observed in UK10K (AAF r = 0.92), for a total of 89% observed in at least one of these cohorts. Of the 756 variants identified in the African American analysis, 70% were observed in 1000 Genomes (AAF r = 0.81) and 64% were observed in UK10K (AAF r = 0.57), for a total of 83% observed in at least one of these cohorts.

It is notable that the significant and apparently common variants tend to lack linkage disequilibrium (LD) partners with even moderate association signals. Only 7 of 703 (Afr) and 13 of 359 (Eur) significant variants with MAF > 5% have LD $r^2 > 0.8$ with some other variant with $p < 0.0001$ within 25kb. When an association signal is observed between a common variant and a phenotype, it is typical to observe a cluster of nearby variants in LD that also have at least moderate association signals. The lack of such LD partners, as observed here, is a classic sign of a spurious signal, lending further support that the observed associations are likely due to genotyping artifacts associated with batch.

Genotyping batch effects, such as those observed for this small percentage of variants, will only be problematic for association testing if the phenotype is also correlated with the relevant batching variable(s). The magnitude of the effect will depend on several factors including: sample size, the genotype frequencies in each batch, and the phenotype distribution/prevalence

in each batch. Given these parameters, as well as the percentage of variants affected by batch effects, we can compute the theoretical quantiles of the distribution of test statistics (the sum of a chi-square distribution for well-behaved variants and a non-central chi-square distribution for affected variants) and calculate the percentage of variants we would expect to identify as significant ($p < 5\times10^{-8}$) in a given analysis.

We present such theoretical results for a binary phenotype under a variety of conditions in Supplementary Table 25. We observe that even modest differences between batches in allele frequencies and case prevalence with modest sample sizes can lead to many spurious association signals. For example, in a sample of 25,000 individuals for which the case prevalence in batches 1 and 2 is 30% and 40%, respectively, and 0.01% of variants are affected by a genotyping batch effect leading to minor allele frequencies (MAF) of 10% and 30% in batches 1 and 2, respectively, we expect to observe 59 spurious associations per every million variants tested; if 30 million variants were tested, that would lead to 1,770 spurious hits. Even for this same sample size and prevalence, but with more subtle batch effects, where only 0.003% of variants are affected such that the MAF in batches 1 and 2 are 20% and 30%, respectively, we expect to observe 0.13 spurious associations per million variants; i.e. about 4 spurious hits when testing 30 million variants. While the fraction of variants identified as spurious associations in this scenario is very small (0.000013%), the identification of those 4 spurious hits can still be problematic for researchers who spend time and money investigating each association signal and designing and performing follow-up replication analyses.

As with all genomic datasets, preventing batch effects is ideal, but difficult in large and complex programs such as TOPMed, which has multiple studies, sequencing centers and time phases. Batch effects on genotype calls in TOPMed appear modest, most likely due to standardization of sequencing methods and joint genotype calling. Nevertheless, analysts should be aware that spurious hits can result from batch effects. Considering factors such as allele frequencies and LD patterns can help to identify such spurious hits. Additionally, the number of spurious hits can potentially be reduced by more stringent quality filtering, which is currently under investigation by the TOPMed IRC, and batching variables can be included in association testing models to lessen their impact. In multi-study association testing, TOPMed investigators typically include study as a covariate in their models. The study variable probably captures a number of potential batching factors because all samples within a study are generally sequenced at the same center within a reasonably short timeframe, and when exceptions to this rule occur, they are generally identifiable (e.g. a study was included in more than one phase, which can also involve splitting between different centers). In any case, investigators are advised to carefully examine the quality of all hits obtained from association testing, as has become standard in the GWAS field.

In conclusion, although TOPMed sequencing has been done over multiple studies, years and centers, batch effects appear to be minor, thus enabling multi-study association testing to maximize sample size.

1.3 Comparison to prior WGS and WES data

1.3.1 Comparison to high coverage WES in BioMe Study

We evaluated potential benefits of WGS relative to exome sequencing. In this comparison, we focus on all GENCODE 26 v31 protein-coding regions for 1,000 individuals from the BioMe TOPMed study (see Methods). Across these 1,000 samples, TOPMed WGS produced 495,580 protein-coding SNVs and 12,418 indels (406,114 and 11,140 with MAF ≤ 1%), ~12% more variants than prior WES, which produced 438,866 SNVs and 9,411 indels (362,308 and 8,596 with MAF ≤ 1%). Per individual, WGS found an average of 23,886 SNVs and 282 indels (1,591 and 32 with MAF ≤ 1%), WES found 20,530 SNVs and 191 indels (1,403 and 26 with MAF ≤ 1%). Overall, per individual, ~80% of variants were found with both approaches and genotypes for overlapping variants were highly concordant (0.9993 concordance for 19,655 overlapping SNVs; 0.9974 for 134 overlapping indels; Supplementary Tables 1 and 2). Among variants missed by WES, the majority were at sites where WES depth was lower (only 6.10% of these sites had >20X WES depth for SNVs, 9.68% for indels), even within WES targeted regions. Among variants that were missed by WGS, we observed a mix of potential explanations – a subset of sites where WES had higher depth, another where WES had very low depth, and another where calls were on the edges of targeted regions.

1.3.2 Comparison to low coverage WGS and high coverage WES in Framingham Heart Study

Investigators in the Framingham Heart Study (FHS) evaluated WGS data from TOPMed in comparison with sequencing data from CHARGE (Cohorts for Heart and Aging Research in Genomic Epidemiology) Consortium WGS and Whole Exome Sequencing (WES). CHARGE is a collaboration of cohort studies of heart, lung, blood, and sleep studies formed for the purpose of genome-wide association studies[36].

FHS WGS for TOPMed was conducted at the Broad Institute. Details of the sequencing procedures are contained in this paper.

The CHARGE WGS contained 5,297 WGS samples in the CHARGE-F3 dataset.They were sequenced using the ILLUMINA HiSeq 2000/2500 with an average depth of coverage ranging between 7× and 10×. The raw data was aligned using the Mercury pipeline[37] . The Mercury pipeline used BWA to align the raw data to the human hg19 reference genome. The samples

consist of three cohorts Cardiovascular Health Study, FHS and Atherosclerosis Risk In Communities Study with 3,396 samples belonging to European American (EuAm) ancestry and 1,901 with African American (AfAm) ancestry. Joint calling used a consensus approach[38] . Repacked tarballs of all sliced BAMs in the same 1 Mbp window were used as input to four variant calling pipelines: GATK-HC[39], GATK-UG[39], GotCloud[40] and SNPTools[41]. Imputation and phasing was profiled using SNPTools imputation engine over 3,176 samples. Further detail can be found in Huang et al. 2016[38]. For sample QC, principal components analysis was used to identify possible population substructure and sample abnormalities. The set of variants for PCA was restricted to variants with MAF>5% and LD (r2<0.30) and was done by chromosome. Two individuals from FHS were identified as outliers in PC1 and were removed from further analyses. For Variant QC, all variants were evaluated for deviation from Hardy-Weinberg equilibrium and exact p-values were calculated and made available. Where SNP array data were available, concordance was calculated as a function of minor allele count. Genotyping accuracy was similar to low pass sequencing data from the 1000 Genomes Project. Based on these evaluations, no variants were recommended for exclusion prior to analysis.

For CHARGE WES Freeze 5 in which FHS was one study, DNA samples were constructed into Illumina paired-end pre-capture libraries according to the manufacturer's protocol. The complete protocol and oligonucleotide sequences are accessible from the Baylor College of Medicine Human Genome Sequencing Center (HGSC) website[42]. Two, four or six pre-capture libraries were pooled together and then hybridized to the HGSC VCRome 2.1 design (42Mb, NimbleGen) and sequenced in paired-end mode in a single lane on the Illumina HiSeq 2000 or the HiSeq 2500 platform. Illumina sequence analysis was performed using the HGSC Mercury analysis pipeline[43]. Pooled samples were de-multiplexed using the Consensus assessment of sequence and variation (CASAVA) software. Reads were mapped to the Genome Reference Consortium Human Build 37 (GRCh37) human reference sequence using Burrows-Wheeler Alignment (BWA) producing Binary Alignment/Map (BAM) files. Aligned reads were then recalibrated using GATK along with BAM sorting, duplicate read marking, and realignment near insertions or deletions (indels). The Atlas2 suite was used to call single nucleotide variants (SNVs) and insertion-deletions (indels) and produce high-quality variant call files (VCF). Each SNV call was filtered based on the following criteria to produce a high-quality variant list: low SNV posterior probability (<0.95), low variant read count (<3), variant read ratio <0.25 or >0.75, strand-bias of more than 99% variant reads in a single strand direction, or total coverage less than 10-fold. All variant calls filtered by these criteria, and reference calls with less than 10-fold coverage, were set to missing. The variant call filters were the same for indels except a total coverage less than 30-fold was used for variant sites. Variant-level quality control steps excluded variants outside the exon capture regions (VCRrome 2.1), monomorphic sites, missing rate >20%, mappability score <0.8, and mean depth of coverage >500-fold. Variants not meeting Hardy-Weinberg equilibrium expectations ($P<5x10^{-6}$) in ancestry-specific groups were also excluded. Sample-level quality control metrics were calculated by cohort and ancestry group. A sample was excluded for missingness >20%, or if compared to the other samples it fell

less than 6 standard deviations (SD) for mean depth, more than 6 SD for singleton count, or outside of 6 SD for heterozygote to homozygote ratio or Ti/Tv ratio. The final sample for CHARGE contained 11263 EA individuals (1751 for CHS, 7810 for ARIC, and 1702 for FHS) and 3180 AA from ARIC. In total, there were 2,556,859 SNVs and 76,133 indels after QC. The mean depth of coverage was 78-fold.

Supplementary Table 19 provides the counts and depth of each sequencing effort. The overlap of these three groups is 430 FHS study participants, on whom we report here. We use a subset of 263 unrelated study participants to calculate the numbers of singletons and doubletons, minor allele frequency (MAF), heterozygosity, and all rates, in order to avoid bias from the family structure. There have been other sequencing efforts in FHS, such as the Exome Sequencing Project, but there was insufficient overlap to include them here.

Supplementary Table 26 shows the number of non-monomorphic variants in each sequencing set. Note that the sets containing all sequenced subjects had more variants than the subgroup of 430 subjects. For the two WGS efforts, the overlap among the 430 subjects of common variants with MAF > 20% is nearly 3 million (Supplementary Figure 45). The CHARGE low coverage WGS has more variants with MAF > 20% than TOPMed; it is likely that different calling and quality control strategies explain this circumstance. Among the 263 study participants, the proportion of variants observed in TOPMed that are also seen in the CHARGE WGS varies from ~67% for rare MAF to ~85% for common variants. Hence, there is a great deal of overlap between the two sequencing efforts, even though CHARGE WGS is low coverage and TOPMed is deep WGS.

Using a set of 263 unrelated individuals, the number of SNV singletons and doubletons found in TOPMed is 9,624,993 and 1,901,162 respectively, while these numbers are lower in the CHARGE WGS at 5,379,585 and 1,344,916. These counts resulted in an average of 36,596.9 singletons and 7,228.7 doubletons per person in TOPMed and 20,454.7 singletons and 5,113.7 doubletons per person in the CHARGE WGS. Dividing the total number for each person by the number of non-monomorphic variants in a dataset (constant for all people in a dataset) yields rates of singletons and doubletons in TOPMed as 1.65×10^{-3} and 3.27×10^{-4} and in CHARGE WGS 1.16×10^{-3} and 2.9×10^{-4}. Hence, the rates of singletons and doubletons are fairly similar between the two WGS efforts.

The rate of bi-allelic SNVs is similar in the two WGS efforts (Supplementary Table 27). The numbers in parentheses are the average number of SNVs seen per person, summing the heterozygous and homozygous calls for the minor allele of each SNV. The rates indicate the average proportion of SNVs that are heterozygous or homozygous for the minor allele. These rates suggest that about 14-15% of variants are heterozygous or homozygous for the minor allele among all SNVs, while the rate in exonic SNVs is lower.

Across all three sequencing efforts (TOPMed, CHARGE WGS and CHARGE WES), rare variants predominated (Supplementary Table 28). Among the 263 unrelated study participants, TOPMed had the greatest number of variants with MAF < 0.005, constituting 47% of the total number. The percentage of variants in this MAF range in the CHARGE WGS was 38% and in the CHARGE WES 60%. Other than the rarest variants, the two WGS efforts have similar numbers in each MAF frequency bin, although the CHARGE WGS has somewhat more, especially in the MAF > 0.05 range.

To focus on the exome, we used Ensembl gene GRCh37.p13 to define exonic regions in the WGS data. The number of exonic SNVs called was greatest in TOPMed with ~17-18% more variants (Supplementary Table 3). The numbers of multi-allelic variants and indels were far fewer relative to SNVs. Fewer multi-allelic variants were seen in the CHARGE WES than in TOPMed (9,226 vs 11,078). The numbers of indels seen in CHARGE WES were about half the ones seen in TOPMed (3,209 vs 5,794). We do not have counts for multi-allelic variants and indels for CHARGE WGS.

We compared the concordance of the calls in the sequencing efforts of the exome with those on the Exome Chip (Illumina Infinium HumanExome BeadChip array v1.0). We found very high concordance with most having concordance > 99%. Only the very rare variants had lower concordance, but still high at > 96.5%. The concordance of the TOPMed exonic variants was the highest across the MAF range with mean concordance rates of 0.999 across the minor allele spectrum; the light-coverage CHARGE WGS had the lowest among the three sequencing sets with concordance rates ranging from 0.990 to 0.998.

In the exome, the average heterozygosity per person for SNVs was 7.97% per variant for TOPMed, 9.66% for CHARGE WGS and 6.85% for CHARGE WES. The average number of heterozygous SNVs per person was 13,208 for TOPMed, 14,601 for CHARGE WGS and 9,640 for CHARGE WES, matching expectations.

Comparing the number of exonic SNVs across the MAF range, TOPMed had the greatest number of rare variants, followed by the CHARGE WES with the low coverage CHARGE WGS having the lowest number (Supplementary Figure 46). In contrast, the light-coverage CHARGE WGS had a greater number of common variants in the 1-50% MAF range. These comparisons match what was observed for the total number of variants (Supplementary Table 28). The CHARGE WES consistently had higher numbers of multi-allelic variants across the minor allele spectrum than TOPMed, nearly double for more common variants of this type. In contrast, TOPMed had similar, but consistently more indels than the CHARGE WES, especially among indels with frequencies greater than 1%.

More than half of exonic variants were non-synonymous in the three sequencing efforts (Supplementary Table 3). In TOPMed 0.96% of variants were LOF while in the light-coverage

CHARGE WGS this percentage was 0.87% and in CHARGE WES it was the same as TOPMed. The numbers of LOF, missense and non-synonymous SNVs by minor allele frequency were comparable across the three sequencing datasets with somewhat fewer among SNVs in the CHARGE WES with MAF > 1%.

In summary, the results suggest more common variants were seen in the light-coverage CHARGE WGS in comparison to TOPMed, while the deep-sequencing in TOPMed revealed many more rare variants compared to TOPMed. Regardless, the vast majority of variants seen in TOPMed were also seen in CHARGE WGS. While CHARGE WGS had somewhat higher rates of common SNVs, this circumstance could possibly be due to different calling and quality control procedures. Other characteristics were similar for the three sequencing datasets. In particular, the heterozygosity rates were similar and the numbers of variants by functional category were similar. Most important, the concordance of each sequencing set with genotypes on the Exome Chip were very high. Hence, we feel confident in using any of the three sequencing sets in data analyses.

1.4 Comparing to GATK variant calling pipeline

We compared our variant call set with one generated using the GATK v4.1.3 standard "best practices" pipeline in 1,207 complete nuclear families (2,414 parents, 1,622 offspring). Nuclear families allowed us to use Mendelian inconsistency rates to assess the quality of each callset (neither variant caller was aware of family structures during analyses). In an average trio, the TOPMed call set included 4,632,934 SNVs (with 0.04% Mendelian inconsistency rate), the GATK call set included 4,924,665 SNVs (with 0.52% inconsistency rate). Of these, 4,590,260 variants overlapped between callsets, with highly concordant genotypes (99.94% concordance, 0.04% Mendelian inconsistency rate) (Supplementary Table 6). Sites unique to either caller had much higher Mendelian inconsistency rates (1.84% for TOPMed, 27.6% for GATK). In an average trio, TOPMed detected 261,355 indels (0.22% inconsistency rate), GATK detected 1,130,696 indels (3.56% inconsistency rate). Of these, 259,865 indel sites overlapped between the call sets (99.37% concordance, 0.20% Mendelian inconsistency rate). Sites unique to either caller again had high Mendelian inconsistency rates (4.73% in TOPMed, 12.51% in GATK). Nearly all (>90%) of the indel variants in the GATK call set are in the initial TOPMed call set but many of them (74.39%) fail site level quality filters. Thus, the TOPMed call set is calibrated to focus on a stringent set of variants that can be genotyped more reliably, resulting in low Mendelian inconsistency rates and minimizing batch effects across centers and studies. Variants that were unique to each caller appear to concentrate in complex regions with a high density of nearby variants.

1.5 Whole genome sequencing accessibility mask

For the population genetic analysis it is important to exclude genomic regions with elevated false positive and false negative variants discovery rates due to pure accessibility by next-generation sequencing methods in these regions. We used per sample coverage information in order to create the accessible genome mask of GRCh38 for our analysis. First, for each of 1,000 randomly selected individuals from TOPMed freeze 5, we computed base-pair coverage using reads with mapping quality greater than 20 and base quality greater than 20. Second, for each base-pair we aggregated coverage across all individuals and computed summary statistics: average coverage, percent of individuals with coverage >1x, >5x, >10x, >50x, and >100x. These coverage summary statistics are available from the BRAVO variant browser at bravo.sph.umich.edu. Base-pairs with N reference allele, low base quality, or where all reads had low map quality were not considered when aggregating. Finally, a base-pair was declared not accessible if: i) reference allele was N; ii) base quality was below threshold or all map qualities were below threshold across all individuals; iii) computed summary statistics did not fall between 1 and 99 percentiles for autosomal chromosomes and pseudoautosomal regions on chromosome X, and between 1 and 99.9 percentiles for non-pseudoautosomal regions on chromosome X.

1.6 On the distribution of inter-singleton distances in a sample

The set of M *de novo* mutations in an individual are often assumed to follow a Poisson distribution, with mutations occurring independently at a constant rate ϕ , represented by the following probability mass function with parameter $\lambda = \phi G$:

$f(m ; \lambda) = e^{-\lambda}(\lambda)^m / m!$ for $m = 0, 1, 2, ...$

where G is the size in base pairs of the mappable genome where *de novo* mutations are ascertained.

Under this simple model, the distances between *de novo* mutations in an individual (measured in base pairs) are expected to follow an exponential distribution with rate $\theta = 1/\phi$:

$f(d) = \theta e^{-\theta d}$

For haploid samples without recombination and assuming a uniform mutation rate, the distances between singleton SNVs within an individual are also expected to follow an exponential distribution, with rate $\theta \times B_i$, where B_i is a constant proportional to the length of the external branch corresponding to sample i in which singletons are ascertained (i.e., a longer external branch will tend to have more singletons than a shorter external branch, because there have been more generations necessary for the longer branch to find a common ancestor in the sample, and thus more time for mutations to accumulate on that branch). However, this naive model of exponential decay does not extend to describing the inter-singleton distance

distribution observed across samples, even under the simple case of haploid samples without recombination. This is due to the intrinsic heterogeneity of external branch lengths across the sample. Consider two independent exponentially distributed random variables, $D_1 \sim exp(B_1 \times \theta)$ and $D_2 \sim exp(B_2 \times \theta)$, where $B_1 \neq B_2$. The probability density of random variable $Z = D_1 + D_2$ can be derived as:

$$f(z) = \frac{B_1 B_2 \theta}{B_2 - B_1}(e^{-(B_1\theta)z} - e^{-(B_2\theta)z})$$

(Note that even if external branch lengths were identical, the probability density of Z is given by $f(z) = (B\theta)^2 z e^{-(B\theta)z}$, which is an Erlang distribution, not an exponential distribution).

This becomes even more complex when we consider a sample of haploid individuals with recombination. Here (again assuming singletons arise via a uniform mutation rate), we can consider the total inter-singleton distance distribution within a single haploid individual as the sum of independent exponential random variables, each corresponding to a haplotype block between recombination breakpoints, with external branch lengths varying across these haplotype blocks within a sample. To further complicate matters, in real short-read sequencing data of diploid individuals we are unable to phase singletons, so the inter-singleton distances are calculated within each diploid individual without consideration of which haplotype the singletons arose, such that the inter-singleton distances are no longer independent with respect to recombination breakpoints of individual haplotype blocks.

Ultimately, this reasoning demonstrates that the demographic history of a sample (specifically the effects of heterogeneous external branch lengths, recombination, and our inability to consider the haplotype-of-origin for singletons) will cause some degree of clustering among singletons in an individual, even in the absence of variable mutation rates and other biological processes which are known to generate clusters of SNVs, such as multinucleotide mutations[44] or temporally non-independent mutation processes[45].

In sensitivity analysis, we compared the results generated with N=1,000 individuals of European ancestry against results generated with N=500 individuals (where individuals in the smaller subsample carry more singletons, and are assumed to have longer external branch lengths). The rate estimates in the N=500 subsample differ from the rate estimates in the N=1,000 subsample primarily in components 3 and 4, indicating that variation in the parameter estimates of these two components can be largely explained by variation in average external branch lengths (i.e., differences in the average number of singletons observed in an individual) (Supplementary Figure 47). Nevertheless, the conclusions and interpretation are broadly the same. Curiously, the parameter estimates of mixture component 2 remain relatively stable regardless of sample size in this analysis, suggesting the between-population differences we observed for this component (Figure 2) are not simply an artifact of average differences in external branch lengths (i.e., the differences in component 2 parameter estimates of the EUR

and AFR subsamples cannot be explained by individuals in the AFR subsample simply having more—and thus more densely-spaced—singletons).

1.7 Novel genetic variants in unmapped reads

From each sample CRAM (mapped against GRCh38), read-pairs with at least one unmapped mate (SAM flags 4 and/or 8) were extracted and converted into FASTQ format. Reads were then sorted by read name and singletons filtered out. Next, read-pairs were screened for adapter sequences and low quality bases (Q<20), which were trimmed using Cutadapt 1.8.1[46]. Read-pairs that, after this process, had either of the two mates shorter than 50bp were removed and the remaining pairs mapped against a phiX reference using GEM mapper[47] for spike-in filtering. Finally, samples with more than 100,000 processed fully unmapped read-pairs at this stage were considered as highly contaminated and were discarded. On a per-sample basis, reads were then assembled into contigs using ABySS v.2.0.2[48] with a *K*-mer size of 77.

Contigs longer than 200bp from all samples were pooled together and mapped against 4 hominid reference genomes (*Pan paniscus* – panPan2; *Pan troglodytes* – panTro6; *Gorilla gorilla* – gorGor5; and *Pongo abelii* – ponAbe3) downloaded from the UCSC Genome Browser[49], using BWA-MEM[50]. Contigs aligning to the same genomic region are likely to represent the same ancestral sequence and were, therefore, merged together based on the alignment pileup and their out-most coordinates transposed into the human reference hg38, using liftOver[51]. Merged contigs that produced a liftOver result in which there was not a significant difference in coordinate pair distance (*interval end - interval start*) between the hominid species and human (<50bp) were discarded. The remaining contigs were then joined into scaffolds based on alignment proximity exploring 3 values of maximum contig distance (2.5kb, 5kb and 7.5 kb), with gap sizes corresponding to the alignment distance in each hominid genome.

For each of the resulting contigs and scaffolds, the out-most alignment coordinates were then transposed into the human reference hg38 using liftOver, defining intervals that may contain the breakpoints for ancestral non-reference sequences. These were aligned to their corresponding contigs/scaffolds using AGE[52]. AGE uses a dynamic programming approach to find the optimal breakpoint position between two partial alignments. If both partial alignments identified by AGE fulfilled the minimal criteria of being 15bp long, with 92% identity and located within 15bp from the contig/scaffold ends, an insertion event was called. Given that four references were used, and three different scaffolding parameters explored, the resulting call set contained a high degree of redundancy. To address this, we clustered overlapping calls (minimum distance of 50bp) and selected a single representative call per cluster, using five criteria: 1) number of non-gap characters; 2) percentage of non-gap characters; 3) alignment distance to the

contig/scaffold edge; 4) number of contigs in scaffold; and 5) length of the terminal contigs. Finally, only insertion calls longer than 100bp were kept. Additionally, to identify breakends, i.e. genomic breakpoint locations of partially assembled insertions, we aligned all contigs/scaffolds to GRCh38 with BWA-MEM and checked whether, within the given liftOver interval, there was a soft-clipped alignment (within 15bp of one contig end) with at least 50 mapped and at least 50 clipped bases. If so, we called a breakend candidate. All breakend candidates (called from each hominid species and from both contigs and scaffolds) were merged and the contig/scaffold with the longest overhanging insertion sequence chosen as the representative. Any breakends overlapping a called insertion position (within 200bp) or a called insertion contig/scaffold ID were discarded.

To assess whether insertions and breakends are enriched in particular regions of the genome, GENCODE v29[53], repeat and segmental duplication annotations for GRCh38 were downloaded through the UCSC Table Browser[54] and overlaps computed using BEDTools v2.25.0[54,55]. Repetitive elements occurring within non-reference sequences were annotated with RepeatMasker v4.0.7[56].

To genotype the full cohort of 53,831 individuals, the final call set of fully resolved insertions was converted from BEDPE into VCF format and used as input for Paragraph v2.2b[57], with the recommended maximum depth parameter (-M) set to 20X the average read depth. To estimate allele frequencies, only sites with at least 95% valid genotypes across unrelated individuals were considered. Comparisons across individuals required that these had valid genotypes across at least 95% of those sites.

1.8 Site frequency spectrum

One way of gaining inference into a population's demographic history is to study the site frequency spectrum (SFS), which shows the proportion of all variants found at each minor allele count.

Using a subsetted sample of 1,370 unrelated individuals each with East Asian, European, and African ancestry we calculated the allele count of each site and generated a log-log histogram of their frequency. 1,370 constitutes all the East Asian samples, and we have subset the other populations randomly to control for the rarity of the variants. A similar procedure was done for the analysis that includes the Amish, except with 225 individuals per cohort, which represents the total number of unrelated Amish individuals in the dataset. Using AC and AN values for each subsampled cohort, we used all variants passing typical variant QC (filter=="PASS") to calculate the frequency of each allele count, as well as its proportion of the total number of variants in the cohort.

Comparing the SFS for 1,370 unrelated individuals with European, African, and East Asian ancestry, we find that all three major groups show an excess of rare variation indicative of recent population expansions[58–60] and purifying selection, with European and East Asian populations exhibiting the greatest excess of rare variants (Supplementary Figure 48). In contrast, the Amish founder population has experienced a very severe and recent bottleneck, and their SFS exhibits a shift toward common variants (Supplementary Figure 49). This then gives a basis for the observation of increased heterozygosity in the Amish compared to East Asians and a higher singleton count in the East Asian groups (Figure 4)

1.9 Admixture

Average ancestry proportions were calculated using a stratified random sample from participating TOPMed studies consented for population genetics research and a standard ADMIXTURE analysis[61,62]. 100 unrelated subjects were randomly sampled from each population group within each TOPMed study. If the population group contained fewer than 100 subjects, the entire unrelated group was included. In total, 4,444 subjects were included in the analysis. Sequencing data was filtered, removing indels, using a minimum MAF of 0.05 and a maximum r^2 value of 0.2. Ancestry proportions were calculated using ADMIXTURE v1.3.0. The value for K was chosen by a cross-validation procedure. The cross-validation errors of Ks 1 through 15 showed two minima, one at a K of 9 and another at a K of 13. K of 9 was chosen as the more conservative value. 20 replications were done; the replication with the highest log-likelihood was used.

ADMIXTURE analysis[61] revealed nine clusters in the dataset, three of which resemble different European ancestries and two of which represent African ancestries (Figure 4). Amish, East Asian, Native American, and Samoan ancestries are all represented by their “own” cluster. The first European cluster contributes similarly to European American and Hispanic/Latino cohorts, the second European cluster is at highest frequency in the European American cohorts, and a third European cluster is at highest frequency in the Hispanic/Latino cohorts. The European ancestry in the African American cohorts is predominantly represented by the second European cluster. We find shared ancestry between the Samoan and the MESA and WHI Asian American subsets, likely due to the recent shared ancestry of Samoan and East Asian populations[63,64].

1.10 Demographic estimation under selection at linked sites

1.10.1 Sample selection

In order to sample individuals with a high percentage of European ancestry and to prevent confounding introduced by population structure, we used two separate ascertainment schemes

for selecting individuals in our study. First, the local ancestry inference program RFMix[65] was run on 18,436 samples from the TOPMed data Freeze 3 using the following parameter settings: PopPhased --num-threads 1 --min-node-size 5. For the reference panel, 938 samples from the Human Genome Diversity Panel[66] (HGDP) were used. The 53 populations of HGDP were condensed into 7 super-populations: 1) Sub-Saharan African (n=104), 2) Central and South Asian (n=200), 3) East Asian (n=229), 4) European (n=154), 5) Native American (n=63), 6) Oceanian (n=28), and 7) Middle Eastern (n=160). After running RFMix, we summed local ancestries assigned for each TOPMed sample to create a vector of global ancestries corresponding to the 7 HGDP super-populations. We then selected individuals that had greater than or equal to 90% global European ancestry (Supplementary Figure 50).

To further limit potential population structure, we also used cluster information from principal components analyses (PCA) on a set of 18,234 individuals from the TOPMed data Freeze 3. We used k-means clustering to cluster individuals on the first 7 PCs. Clustering was initially run on various values of k from 1 to 20, with 25 restarts each. We plotted the total within class sum of squares from this initial clustering round and chose k=9 since large decreases in the sum of squares diminish after this point (Supplementary Figure 51). We then re-ran the clustering for k=9 with 250 restarts to finalize the cluster assignments for the 18,234 samples. This left us with population clusters that roughly aligned with several reported categories, and we assigned population labels based on these (see Supplementary Table 29). We then filtered individuals for those that belonged to the population 1 ('European A') cluster. Finally, we limited our selected individuals to those that were unrelated and gave consent for performing population genetics research. The intersect of the two ascertainment schemes resulted in a total of 2,416 samples for performing further analyses and demographic inference. When measuring the site-frequency spectrum across these samples as a function of sample size, we sampled progressively larger random samples of 50 individuals (100 chromosomes) each, generating 49 discrete sample sizes (2N=100, 200, 300…4800, 4832).

1.10.2 Site filtering/ascertainment

In order to perform inference using a high-quality set of neutral sites that are least influenced by the direct effects of natural selection and putative selective sweeps and to avoid potential sequence/mapping error, we performed several steps to filter the genome. Many of these filtering steps were based off of the ascertainment scheme used by Torres et al. 2018[67]. Specifically, the following filters were applied (all filters are in hg19 and only autosomes were kept for analyses):

1. Coding regions: coding exons annotated in the UCSC known genes track (table: knownGene, track: UCSC Genes) were removed.
2. phyloP: Sites with phyloP[68] scores > 1.2 or < -1.2 were removed to limit the effects of natural selection due to conservation or accelerated evolution. Scores were downloaded from http://hgdownload.cse.ucsc.edu/goldenPath/hg19/phyloP46way/.

3. phastCons: Regions in the UCSC conservation 46-way track (table: phastCons46wayPlacental)[69] were removed to limit the effects of natural selection due to conservation.
4. CpG: CpG islands in the UCSC CpG islands track were removed because of their potential role in gene regulation and/or being conserved.
5. ENCODE blacklist: Regions with high signal artifacts from next-generation sequencing experiments discovered during the ENCODE project[70] were removed.
6. Simple repeats: Regions in the UCSC simple repeats track were removed due to potential misalignments with outgroups and/or being under natural selection.
7. Gaps/centromeres/telomeres: Regions in the UCSC gap track were removed, including centromeres and telomeres.
8. Segmental duplications: Regions in the UCSC segmental dups track[71] were removed to limit potential effects of natural selection.
9. Transposons: Active transposons (HERVK retrotransposons, the AluY subfamily of Alu elements, SVA elements, and L1Ta/L1pre-Ta LINEs) in the human genome were removed.
10. Recent positive selection: Regions inferred to be under hard and soft selective sweeps (using iHS and iHH12 regions from selscan v1.2.0[67,72]); within Thousand Genomes phase 3[28] European and African populations were removed.
11. Non-coding transcripts: Non-coding transcripts from the UCSC genes track were removed to limit potential effects of natural selection.
12. GC-biased gene conversion (gBGC): Regions in UCSC phastBias track[73] from UCSC genome browser were removed to limit regions inferred to be under strong GC-biased gene conversion.
13. Recombination hotspots: All sites within 1.5 kb (i.e., 3 kb windows) of sites with recombination rates >= 10 cM/Mb in the 1000G OMNI genetic maps for non-admixed populations (downloaded from ftp://ftp.1000genomes.ebi.ac.uk/vol1/ftp/technical/working/20130507_omni_recombination_rates/) and the HapMap II genetic map[74] were removed. 1.5 kb flanking regions surrounding the center of hotspots identified by Pratto et al. 2014[75] (downloaded from http://science.sciencemag.org/content/sci/suppl/2014/11/12/346.6211.1256442.DC1/1256442_DatafileS1.txt) were also removed, except for the cases in which the entire hotspot site was greater than 3 kb in length (in which case just the hotspot was removed).

Positions in the genome were then annotated for how strongly they are affected by selection at linked sites by using the background selection (BGS) coefficient, *B*[76] (McVicker's *B* statistic; downloaded from http://www.phrap.org/othersoftware.html). BGS is a process by which neutral variation in the genome is affected by purifying selection via genetic linkage to deleterious sites[77–79]. *B* represents the fraction of neutral genetic variation at a particular site in a population suffering BGS relative to a neutrally evolving population and varies between 0 and 1, with BGS increasing in strength as values of *B* approach 0. Positions for *B* were lifted over from hg18 to

hg19 using the UCSC liftOver tool. Sites that failed to uniquely map from hg18 to hg19 or failed to uniquely map in the reciprocal direction were excluded. Sites lacking a *B* value were also ignored. We used all sites annotated with a *B* value for performing general analyses. However, when performing demographic inference, we only focused our analyses on those regions of the genome within the top 1% of the genome-wide distribution of *B* (*B* >= 0.994). These sites correspond to regions of the genome inferred to be under the weakest amount of BGS (i.e., under the weakest effects of selection at linked sites).

Sites in the genome were also polarized to ancestral and derived states using ancestral annotations called with high-confidence from the GRCh37 e71 ancestral sequence (downloaded from:
ftp://ftp.ensembl.org/pub/release-71/fasta/ancestral_alleles/homo_sapiens_ancestor_GRCh37_e71.tar.bz2) from Ensembl[76,80], which used a multiple species alignment of 6 primates to infer the ancestral state using the Enredo-Pecan-Ortheus (EPO) pipeline[81,82]. All of the filtering steps described, including the annotation for *B* and polarization for ancestral/derived state, left 1,377,691,456 sites within the genome for use in our study, including 10,977,437 sites with *B* >= 0.994. Finally, we filtered polymorphic sites within the filtered genome on being bi-allelic only. This left a total 20,324,704 polymorphic sites across the 2,416 European samples, including 191,631 polymorphic sites that had *B* >= 0.994. To generate a set of fourfold degenerate synonymous sites, all coding sites within the genome (irrespective of *B*) were annotated using the program ANNOVAR[83] using Gencode V19 annotations. This resulted in 5,188,972 total sites. 4,718,653 sites were left after filtering for high-confidence ancestral/derived states, of which 91,177 were polymorphic (bi-allelic) across the 2,416 European samples. Fourfold degenerate synonymous sites were not filtered on *B*.

1.10.3 Demographic inference

We performed demographic inference using the program *moments*[84], which fits a specified demographic model to an observed site-frequency spectrum. For our study, we specified a model of exponential growth with three total parameters (N_{Eur0}, N_{Eur}, T_{Eur}). This included two free parameters: the starting time of exponential growth (T_{Eur}) and the ending population size after growth (N_{Eur}). The ancestral size parameter (i.e, the population size when growth begins), N_{Eur0}, was kept constant in our model such that the relative starting size of the population was always 1. We applied the inference procedure to the 2,416 European samples using either fourfold degenerate sites or sites where *B* >= 0.994 (highest 1% *B* bin). The site-frequency spectrum used for inference was unfolded and based on the polarization step described above. The inference procedure was fit using sample sizes (2N) of 1000, 2000, 3000, 4000, and 4,832 samples (i.e., chromosomes). The inference procedure was run from different initial starting points hundreds of times for each sample size and dataset to ensure convergence on a global optimum. Attempts at using samples sizes smaller than 2N=1000 for inference resulted in convergence issues, likely because of poor model fit.

To convert the scaled genetic parameters output by the inference procedure moments to physical units, we used the resulting theta (also inferred by *moments*) and a mutation rate of 1.66 x 10^{-8} [85] to generate corresponding effective population sizes (N_e). To account for the fact that fourfold degenerate sites and sites from regions within the highest 1% *B* bin are ascertained from different effective sequence lengths, we had to first normalize theta by their corresponding lengths. These lengths were 4,718,653 sites and 10,977,437 sites for fourfold degenerate sites and highest 1% *B* sites, respectively. To convert time to years, we used a generation time of 25 years. 95% confidence intervals were generated by resampling the SFS 1,000 times and using the Godambe Information Matrix to generate parameter uncertainties[86].

1.11 Selection

The same 7-way local ancestry inference procedure described in Supplementary Information 1.10 was repeated on TOPMed data freeze 5. We use these local ancestry calls to determine which samples had > 90% inferred European, African, or East Asian ancestry.

We used only biallelic sites, and the WGSA pipeline to infer ancestral state. Any sites for which ancestral state was ambiguous were filtered. Alleles were then polarized such that 1 represents the derived allele and 0 represents the ancestral allele. We filter all sites with minor allele frequency less than 0.05 (although we retain singletons) as well as sites near chromosome boundaries and centromeres being excluded due to a truncation of the distance to the nearest singleton. The final number of sites for which we calculated SDS scores, per population, is in Supplemental Table 30.

We used the scripts provided by Yair et al. (2016)[87] to perform all SDS computations and demographic history simulations. In order to compute the normalizing alpha values required for SDS computations, we needed to simulate demographic histories. We simulated histories from Gravel et al. (2011)[88], where we use the marginal histories (with no migration) of the jointly inferred three-population African, European, and East Asian history.

We then normalized raw SDS scores within 1% frequency bins, a requirement since raw SDS is correlated with allele frequency. After normalization, SDS scores are approximately normally distributed. We therefore follow Yair et al. (2016)[87] and convert normalized SDS scores to p-values by treating them as Z-scores. Raw and normalized SDS scores are provided in Supplementary Data 2.

2 Study acknowledgments

NHLBI TOPMed: Genetics of Cardiometabolic Health in the Amish

The Amish studies upon which these data are based were supported by NIH grants R01 AG18728, U01 HL072515, R01 HL088119, R01 HL121007, and P30 DK072488. See publication: PMID: 18440328

NHLBI TOPMed: Trans-Omics for Precision Medicine Whole Genome Sequencing Project: ARIC

The Atherosclerosis Risk in Communities study has been funded in whole or in part with Federal funds from the National Heart, Lung, and Blood Institute, National Institutes of Health, Department of Health and Human Services (contract numbers HHSN268201700001I, HHSN268201700002I, HHSN268201700003I, HHSN268201700004I and HHSN268201700005I, and grant number R01HL059367). The authors thank the staff and participants of the ARIC study for their important contributions.

NHLBI TOPMed: The Genetics and Epidemiology of Asthma in Barbados

The Genetics and Epidemiology of Asthma in Barbados is supported by National Institutes of Health (NIH) National Heart, Lung, Blood Institute TOPMed (R01 HL104608-S1) and: R01 AI20059, K23 HL076322, and RC2 HL101651. For the specific cohort descriptions and descriptions regarding the collection of phenotype data can be found at: https://www.nhlbiwgs.org/group/bags-asthma. The authors wish to give special recognition to the individual study participants who provided biological samples and or data, without their support in research none of this would be possible.

NHLBI TOPMed: Cleveland Clinic Atrial Fibrillation Study

The research reported in this article was supported by grants from the National Institutes of Health (NIH) National Heart, Lung, and Blood Institute grants R01 HL090620 and R01 HL111314, the NIH National Center for Research Resources for Case Western Reserve University and the Cleveland Clinic Clinical and Translational Science Award (CTSA) UL1-RR024989, the Department of Cardiovascular Medicine philanthropic research fund, Heart

and Vascular Institute, Cleveland Clinic, the Fondation Leducq grant 07-CVD 03, and The Atrial Fibrillation Innovation Center, State of Ohio.

NHLBI TOPMed: The Cleveland Family Study (WGS)
Support for the Cleveland Family Study was provided by NHLBI grant numbers R01 HL46380, R01 HL113338 and R35 HL135818.

NHLBI TOPMed: Cardiovascular Health Study
This research was supported by contracts HHSN268201200036C, HHSN268200800007C, HHSN268201800001C, N01-HC85079, N01-HC-85080, N01-HC-85081, N01-HC-85082, N01-HC-85083, N01-HC-85084, N01-HC-85085, N01-HC-85086, N01-HC-35129, N01-HC-15103, N01-HC-55222, N01-HC-75150, N01-HC-45133, and N01-HC-85239; grant numbers U01 HL080295, U01 HL130114 and R01 HL059367 from the National Heart, Lung, and Blood Institute, and R01 AG023629 from the National Institute on Aging, with additional contributions from the National Institute of Neurological Disorders and Stroke. A full list of principal CHS investigators and institutions can be found at https://chs-nhlbi.org/pi. Its content is solely the responsibility of the authors and does not necessarily represent the official views of the National Institutes of Health.

NHLBI TOPMed: Genetic Epidemiology of COPD (COPDGene) in the TOPMed Program
This research used data generated by the COPDGene study, which was supported by NIH Award Number U01 HL089897 and Award Number U01 HL089856 from the National Heart, Lung, and Blood Institute. The content is solely the responsibility of the authors and does not necessarily represent the official views of the National Heart, Lung, and Blood Institute or the National Institutes of Health.
The COPDGene project is also supported by the COPD Foundation through contributions made to an Industry Advisory Board comprised of AstraZeneca, Boehringer Ingelheim, GlaxoSmithKline, Novartis, Pfizer, Siemens and Sunovion.

NHLBI TOPMed: The Genetic Epidemiology of Asthma in Costa Rica
This study was supported by NHLBI grants R37 HL066289 and P01 HL132825. We wish to acknowledge the investigators at the Channing Division of Network Medicine at Brigham and Women's Hospital, the investigators at the Hospital Nacional de Niños in San José, Costa Rica

and the study subjects and their extended family members who contributed samples and genotypes to the study, and the NIH/NHLBI for its support in making this project possible.

NHLBI TOPMed: Diabetes Heart Study African American Coronary Artery Calcification (AA CAC)

This work was supported by R01 HL92301, R01 HL67348, R01 NS058700, R01 AR48797, R01 DK071891, the General Clinical Research Center of the Wake Forest University School of Medicine (M01 RR07122, F32 HL085989), the American Diabetes Association, and a pilot grant from the Claude Pepper Older Americans Independence Center of Wake Forest University Health Sciences (P60 AG10484).

NHLBI TOPMed: Boston Early-Onset COPD Study in the TOPMed Program

The Boston Early-Onset COPD Study (dbGaP accession number phs000946) was supported by the following NIH grants: R01 HL075478, U01 HL089856, and R01 HL113264.

NHLBI TOPMed: Whole Genome Sequencing and Related Phenotypes in the Framingham Heart Study

The Framingham Heart Study (FHS) acknowledges the support of contracts NO1-HC-25195 and HHSN268201500001I from the National Heart, Lung, and Blood Institute and grant supplement R01 HL092577-06S1 for this research. We also acknowledge the dedication of the FHS study participants without whom this research would not be possible. Dr. Vasan is supported in part by the Evans Medical Foundation and the Jay and Louis Coffman Endowment from the Department of Medicine, Boston University School of Medicine.

NHLBI TOPMed: Genes-environments and Admixture in Latino Asthmatics (GALA II) Study

The Genes-environments and Admixture in Latino Americans (GALA II) Study was supported by the National Heart, Lung, and Blood Institute of the National Institute of Health (NIH) grants R01HL117004 and X01HL134589; study enrollment supported by the Sandler Family Foundation, the American Asthma Foundation, the RWJF Amos Medical Faculty Development Program, Harry Wm. and Diana V. Hind Distinguished Professor in Pharmaceutical Sciences II and the National Institute of Environmental Health Sciences grant R01ES015794.

The GALA II study collaborators include Shannon Thyne, UCSF; Harold J. Farber, Texas Children's Hospital; Denise Serebrisky, Jacobi Medical Center; Rajesh Kumar, Lurie Children's

Hospital of Chicago; Emerita Brigino-Buenaventura, Kaiser Permanente; Michael A. LeNoir, Bay Area Pediatrics; Kelley Meade, UCSF Benioff Children's Hospital, Oakland; William Rodriguez-Cintron, VA Hospital, Puerto Rico; Pedro C. Avila, Northwestern University; Jose R. Rodriguez-Santana, Centro de Neumologia Pediatrica; Luisa N. Borrell, City University of New York; Adam Davis, UCSF Benioff Children's Hospital, Oakland; Saunak Sen, University of Tennessee and Fred Lurmann, Sonoma Technologies, Inc.

The authors acknowledge the families and patients for their participation and thank the numerous health care providers and community clinics for their support and participation in GALA II. In particular, the authors thank study coordinator Sandra Salazar; the recruiters who obtained the data: Duanny Alva, MD, Gaby Ayala-Rodriguez, Lisa Caine, Elizabeth Castellanos, Jaime Colon, Denise DeJesus, Blanca Lopez, Brenda Lopez, MD, Louis Martos, Vivian Medina, Juana Olivo, Mario Peralta, Esther Pomares, MD, Jihan Quraishi, Johanna Rodriguez, Shahdad Saeedi, Dean Soto, Ana Taveras; and the lab researcher Celeste Eng who processed the biospecimens.

NHLBI TOPMed: Genetic Epidemiology Network of Arteriopathy (GENOA)

Support for GENOA was provided by the National Heart, Lung, and Blood Institute (HL054457, HL054464, HL054481, HL087660, HL085571, and HL119443) of the National Institutes of Health.

NHLBI TOPMed: Genetic Epidemiology Network of Salt Sensitivity (GenSalt)

The Genetic Epidemiology Network of Salt-Sensitivity (GenSalt) was supported by research grants (U01HL072507, R01HL087263, and R01HL090682) from the National Heart, Lung, and Blood Institute, National Institutes of Health, Bethesda, MD.

NHLBI TOPMed: Genetics of Lipid Lowering Drugs and Diet Network (GOLDN)

GOLDN biospecimens, baseline phenotype data, and intervention phenotype data were collected with funding from the National Heart, Lung and Blood Institute (NHLBI) grant U01 HL072524. Whole-genome sequencing in GOLDN was funded by NHLBI grant R01 HL104135 and supplement R01 HL104135-04S1.

NHLBI TOPMed: Heart and Vascular Health Study (HVH)

The research reported in this article was supported by grants HL068986, HL085251, HL095080, and HL073410 from the National Heart, Lung, and Blood Institute.

NHLBI TOPMed: Hypertension Genetic Epidemiology Network (HyperGEN)

The HyperGEN Study is part of the National Heart, Lung, and Blood Institute (NHLBI) Family Blood Pressure Program; collection of the data represented here was supported by grants U01 HL054472 (MN Lab), U01 HL054473 (DCC), U01 HL054495 (AL FC), and U01 HL054509 (NC FC). The HyperGEN: Genetics of Left Ventricular Hypertrophy Study was supported by NHLBI grant R01 HL055673 with whole-genome sequencing made possible by supplement -18S1.

NHLBI TOPMed: The Jackson Heart Study

The Jackson Heart Study (JHS) is supported and conducted in collaboration with Jackson State University (HHSN268201800013I), Tougaloo College (HHSN268201800014I), the Mississippi State Department of Health (HHSN268201800015I/HHSN26800001) and the University of Mississippi Medical Center (HHSN268201800010I, HHSN268201800011I and HHSN268201800012I) contracts from the National Heart, Lung, and Blood Institute (NHLBI) and the National Institute for Minority Health and Health Disparities (NIMHD). The authors also wish to thank the staff and participants of the JHS.

NHLBI TOPMed: Multi-Ethnic Study of Atherosclerosis

MESA and the MESA SHARe projects are conducted and supported by the National Heart, Lung, and Blood Institute (NHLBI) in collaboration with MESA investigators. Support for MESA is provided by contracts 75N92020D00001, HHSN268201500003I, N01-HC-95159, 75N92020D00005, N01-HC-95160, 75N92020D00002, N01-HC-95161, 75N92020D00003, N01-HC-95162, 75N92020D00006, N01-HC-95163, 75N92020D00004, N01-HC-95164, 75N92020D00007, N01-HC-95165, N01-HC-95166, N01-HC-95167, N01-HC-95168, N01-HC-95169, UL1-TR-000040, UL1-TR-001079, UL1-TR-001420. Also supported in part by the National Center for Advancing Translational Sciences, CTSI grant UL1TR001881, and the National Institute of Diabetes and Digestive and Kidney Disease Diabetes Research Center (DRC) grant DK063491 to the Southern California Diabetes Endocrinology Research Center.

NHLBI TOPMed: Whole Genome Sequencing of Venous Thromboembolism (WGS of VTE)

Funded in part by grants from the National Institutes of Health, National Heart, Lung, and Blood Institute (HL66216 and HL83141) and the National Human Genome Research Institute (HG04735).

NHLBI TOPMed: MGH Atrial Fibrillation Study

The research reported in this article was supported by NIH grants K23HL071632, K23HL114724, R21DA027021, R01HL092577, R01HL092577S1, R01HL104156, K24HL105780, U01HL65962, R01HL128914, and American Heart Association, 18SFRN34110082. The research has also been supported by an Established Investigator Award from the American Heart Association (13EIA14220013) and by support from the Fondation Leducq (14CVD01).

NHLBI TOPMed: Partners HealthCare Biobank

We thank the Broad Institute for generating high-quality sequence data supported by the NHLBI grant 3R01HL092577-06S1 to Dr. Patrick Ellinor. The datasets used in this manuscript were obtained from dbGaP at http://www.ncbi.nlm.nih.gov/gap through dbGaP accession number phs001024.

NHLBI TOPMed: San Antonio Family Heart Study (WGS)

Collection of the San Antonio Family Study data was supported in part by National Institutes of Health (NIH) grants R01 HL045522, MH078143, MH078111 and MH083824; and whole genome sequencing of SAFS subjects was supported by U01 DK085524 and R01 HL113323. We are very grateful to the participants of the San Antonio Family Study for their continued involvement in our research programs.

NHLBI TOPMed: Study of African Americans, Asthma, Genes and Environment (SAGE)

The Study of African Americans, Asthma, Genes and Environments (SAGE) was supported by the National Heart, Lung, and Blood Institute of the National Institute of Health (NIH) grants R01HL117004 and X01HL134589; study enrollment supported by the Sandler Family Foundation, the American Asthma Foundation, the RWJF Amos Medical Faculty Development Program, Harry Wm. and Diana V. Hind Distinguished Professor in Pharmaceutical Sciences II.

The SAGE study collaborators include Harold J. Farber, Texas Children's Hospital; Emerita Brigino-Buenaventura, Kaiser Permanente; Michael A. LeNoir, Bay Area Pediatrics; Kelley Meade, UCSF Benioff Children’s Hospital, Oakland; Luisa N. Borrell, City University of New York; Adam Davis, UCSF Benioff Children’s Hospital, Oakland and Fred Lurmann, Sonoma Technologies, Inc.

The authors acknowledge the families and patients for their participation and thank the numerous health care providers and community clinics for their support and participation in

SAGE. In particular, the authors thank study coordinator Sandra Salazar; the recruiters who obtained the data: Lisa Caine, Elizabeth Castellanos, Brenda Lopez, MD, Shahdad Saeedi; and the lab researcher Celeste Eng who processed the biospecimens.

NHLBI TOPMed: Genome-wide Association Study of Adiposity in Samoans
Financial support from the U.S. National Institutes of Health Grant R01-HL093093. We acknowledge the assistance of the Samoa Ministry of Health and the Samoa Bureau of Statistics for their guidance and support in the conduct of this study. We thank the local village officials for their help and the participants for their generosity. The following publication describes the origin of the dataset: Hawley NL, Minster RL, Weeks DE, Viali S, Reupena MS, Sun G, Cheng H, Deka R, McGarvey ST. Prevalence of Adiposity and Associated Cardiometabolic Risk Factors in the Samoan Genome-Wide Association Study. Am J Human Biol 2014. 26: 491-501. DOI: 10.1002/jhb.22553. PMID: 24799123.

NHLBI TOPMed: The Vanderbilt AF Ablation Registry
The research reported in this article was supported by grants from the American Heart Association to Dr. Shoemaker (11CRP742009), Dr. Darbar (EIA 0940116N), and grants from the National Institutes of Health (NIH) to Dr. Darbar (R01 HL092217), and Dr. Roden (U19 HL65962, and UL1 RR024975). The project was also supported by a CTSA award (UL1 TR00045) from the National Center for Advancing Translational Sciences. Its contents are solely the responsibility of the authors and do not necessarily represent the official views of the National Center for Advancing Translational Sciences or the NIH.

NHLBI TOPMed: The Vanderbilt Atrial Fibrillation Registry
The research reported in this article was supported by grants from the American Heart Association to Dr. Darbar (EIA 0940116N), and grants from the National Institutes of Health (NIH) to Dr. Darbar (HL092217), and Dr. Roden (U19 HL65962, and UL1 RR024975). This project was also supported by CTSA award (UL1TR000445) from the National Center for Advancing Translational Sciences. Its contents are solely the responsibility of the authors and do not necessarily represent the official views of the National Center for Advancing Translational Sciences of the NIH.

NHLBI TOPMed: Novel Risk Factors for the Development of Atrial Fibrillation in Women

The Women's Genome Health Study (WGHS) is supported by HL 043851 and HL099355 from the National Heart, Lung, and Blood Institute and CA 047988 from the National Cancer Institute, the Donald W. Reynolds Foundation with collaborative scientific support and funding for genotyping provided by Amgen. AF endpoint confirmation was supported by HL-093613 and a grant from the Harris Family Foundation and Watkin's Foundation.

NHLBI TOPMed: Rare Variants for Hypertension in Taiwan Chinese (THRV)

The Rare Variants for Hypertension in Taiwan Chinese (THRV) is supported by the National Heart, Lung, and Blood Institute (NHLBI) grant (R01HL111249) and its participation in TOPMed is supported by an NHLBI supplement (R01HL111249-04S1). THRV is a collaborative study between Washington University in St. Louis, LA BioMed at Harbor UCLA, University of Texas in Houston, Taichung Veterans General Hospital, Taipei Veterans General Hospital, Tri-Service General Hospital, National Health Research Institutes, National Taiwan University, and Baylor University. THRV is based (substantially) on the parent SAPPHIRe study, along with additional population-based and hospital-based cohorts. SAPPHIRe was supported by NHLBI grants (U01HL54527, U01HL54498) and Taiwan funds, and the other cohorts were supported by Taiwan funds.

NHLBI TOPMed: Women's Health Initiative (WHI)

The WHI program is funded by the National Heart, Lung, and Blood Institute, National Institutes of Health, U.S. Department of Health and Human Services through contracts HHSN268201600018C, HHSN268201600001C, HHSN268201600002C, HHSN268201600003C, and HHSN268201600004C.

NHLBI TOPMed: BioMe Biobank at Mount Sinai

The Mount Sinai BioMe Biobank has been supported by The Andrea and Charles Bronfman Philanthropies and in part by Federal funds from the NHLBI and NHGRI (U01HG00638001; U01HG007417; X01HL134588). We thank all participants in the Mount Sinai Biobank. We also thank all our recruiters who have assisted and continue to assist in data collection and management and are grateful for the computational resources and staff expertise provided by Scientific Computing at the Icahn School of Medicine at Mount Sinai.

NHLBI TOPMed: My Life, Our Future – Genotyping for Progress in Hemophilia

The My Life, Our Future samples and data are made possible through the partnership of Bloodworks Northwest, the American Thrombosis and Hemostasis Network, the National

Hemophilia Foundation, and Bioverativ. We gratefully acknowledge the hemophilia treatment centers and their patients who provided biological samples and phenotypic data.

NHLBI TOPMed: Outcome Modifying Gene in Sickle Cell Disease (OMG-SCD)
The OMG-SCD study was administrated by Marilyn J. Telen, M.D. and Allison E. Ashley-Koch, Ph.D. from Duke University Medical Center and collection of the data set was supported by grants HL068959 and HL079915 from the National Heart, Lung, and Blood Institute (NHLBI) of the National Institute of Health (NIH).

NHLBI TOPMed: Treatment of Pulmonary Hypertension and Sickle Cell Disease With Sildenafil Therapy (Walk-PHaSST)
Special thanks to the volunteers who participated in the Walk-PHaSST study and the investigators of this study. This project was funded with federal funds from the NHLBI, NIH, Department of Health and Human Services, under contract HHSN268200617182C. We also thank the Walk-PHaSST Biorepository at the University of Pittsburgh for their support in this study.

NHLBI TOPMed: GeneSTAR (Genetic Study of Atherosclerosis Risk)
The Johns Hopkins Genetic Study of Atherosclerosis Risk (GeneSTAR) was supported by grants from the National Institutes of Health through the National Heart, Lung, and Blood Institute (U01HL72518, HL087698, HL112064) and by a grant from the National Center for Research Resources (M01-RR000052) to the Johns Hopkins General Clinical Research Center. We would like to thank the participants and families of GeneSTAR and our dedicated staff for all their sacrifices.

NHLBI TOPMed: Molecular Mechanisms of Inherited Cardiomyopathies and Arrhythmias in the Australian Familial AF Study (AustralianFamilialAF)
This study was supported by the Victor Chang Cardiac Research Institute, National Health and Medical Research Council of Australia, Estate of the Late RT Hall, Simon Lee Foundation and the St Vincent's Clinic Foundation

NHLBI TOPMed: Mayo Clinic Venous Thromboembolism Study (Mayo_VTE)
Funded, in part, by grants from the National Institutes of Health, National Heart, Lung and Blood Institute (HL66216 and HL83141). the National Human Genome Research Institute (HG04735, HG06379), and research support provided by Mayo Foundation.

NHLBI TOPMed: Multi-Ethnic Study of Atherosclerosis (MESA)
MESA and the MESA SHARe project are conducted and supported by the National Heart, Lung, and Blood Institute (NHLBI) in collaboration with MESA investigators. Support for MESA is provided by contracts HHSN268201500003I, N01-HC-95159, N01-HC-95160, N01-HC-95161, N01-HC-95162, N01-HC-95163, N01-HC-95164, N01-HC-95165, N01-HC-95166,

N01-HC-95167, N01-HC-95168, N01-HC-95169, UL1-TR-000040, UL1-TR-001079, UL1-TR-001420. MESA Family is conducted and supported by the National Heart, Lung, and Blood Institute (NHLBI) in collaboration with MESA investigators. Support is provided by grants and contracts R01HL071051, R01HL071205, R01HL071250, R01HL071251, R01HL071258, R01HL071259, and by the National Center for Research Resources, Grant UL1RR033176. The provision of genotyping data was supported in part by the National Center for Advancing Translational Sciences, CTSI grant UL1TR001881, and the National Institute of Diabetes and Digestive and Kidney Disease Diabetes Research Center (DRC) grant DK063491 to the Southern California Diabetes Endocrinology Research Center.

NHLBI TOPMed: Genetics of Sarcoidosis in African Americans (Sarcoidosis)
National Institutes of Health (R01HL113326, P30 GM110766-01)

NHLBI TOPMed: Coronary Artery Risk Development in Young Adults (CARDIA)
The Coronary Artery Risk Development in Young Adults Study (CARDIA) is conducted and supported by the National Heart, Lung, and Blood Institute (NHLBI) in collaboration with the University of Alabama at Birmingham (HHSN268201800005I & HHSN268201800007I), Northwestern University (HHSN268201800003I), University of Minnesota (HHSN268201800006I), and Kaiser Foundation Research Institute (HHSN268201800004I). CARDIA was also partially supported by the Intramural Research Program of the National Institute on Aging (NIA) and an intra-agency agreement between NIA and NHLBI (AG0005).

NHLBI TOPMed: ATGC Gene-Environment, Admixture and Latino Asthmatics Study I Asthma (GALAI)
The Genes-environments and Admixture in Latino Americans (GALA I) Study was supported by the National Heart, Lung, and Blood Institute of the National Institute of Health (NIH) grants R01HL117004 and X01HL134589; study enrollment supported by Sandler Center for Basic Research in Asthma and the Sandler Family Foundation, the American Asthma Foundation, the American Lung Association, the NIH grants K23HL04464 and HL07185, the Resource Centers for Minority Aging Research from the National Institute on Aging, RCMAR P30-AG15272, the National Institute of Nursing Research and the National Center on Minority Health and Health Disparities.

NHLBI TOPMed: Hispanic Community Health Study - Study of Latinos (HCHS/SOL)
The Hispanic Community Health Study/Study of Latinos is a collaborative study supported by contracts from the National Heart, Lung, and Blood Institute (NHLBI) to the University of North Carolina (HHSN268201300001I / N01-HC-65233), University of Miami (HHSN268201300004I / N01-HC-65234), Albert Einstein College of Medicine (HHSN268201300002I / N01-HC-65235), University of Illinois at Chicago – HHSN268201300003I / N01-HC-65236 Northwestern Univ), and San Diego State University (HHSN268201300005I / N01-HC-65237). The following Institutes/Centers/Offices have contributed to the HCHS/SOL through a transfer of funds to the

NHLBI: National Institute on Minority Health and Health Disparities, National Institute on Deafness and Other Communication Disorders, National Institute of Dental and Craniofacial Research, National Institute of Diabetes and Digestive and Kidney Diseases, National Institute of Neurological Disorders and Stroke, NIH Institution-Office of Dietary Supplements.

3 Other acknowledgments

Alisa K. Manning was supported in part by grants K01 DK107836 and R03 DK118305.
Diane Fatkin was supported by the National Health and Medical Research Council of Australia (1074386), with additional support from the Victor Chang Cardiac Research Institute, Estate Late RT Hall, and the Simon Lee Foundation.
Gonçalo R. Abecasis was supported in part by grants U01HL117626, R01HG007022, and HHSN268201800002I.
Anna Köttgen was supported in part by grant DFG KO 3598/5-1.
Lori Garman and Courtney Montgomery were supported in part by grant HL113326.
Ani Manichaikul was supported in part by grant R01 HL131565.
Kari E. North was supported in part by grants 75N92019D00010, R01HL142302, and R01HG010297.
Charles Kooperberg was supported by the National Heart, Lung, and BloodInstitute, National Institutes of Health, U.S. Department of Health and Human Services through contracts HHSN268201600018C, HHSN268201600001C, HHSN268201600002C, HHSN268201600003C, and HHSN268201600004C.
Michelle Daya and Kathleen C. Barnes were supported in part by grant R01HL104608.
Cristen J. Willer was supported in part by grants R01-HL127564, R35-HL135824, and R01-HL142023.
Seung Hoan Choi was the recipient of an analysis support program from TOPMed.
May E. Montasser was supported in part by grants U01 HL137181 and AHA 17GRNT33661168.
Eimear E. Kenny was supported in part by grant X01HL134588.
Anne-Katrin Emde and Soren Germer were supported in part by a Centers for Common Disease Genomics grant from the National Human Genome Research Institute (UM1HG008901).
Thomas W. Blackwell was supported in part by grants U01HL117626, HHSN268201800002I, and R01HG007022.
Patrick T. Ellinor was supported in part by grants from the National Institutes of Health (1RO1HL092577, R01HL128914, K24HL105780), American Heart Association (18SFRN34110082), Fondation Leducq (14CVD01), Carol and Roch Hillenbrand and the George L. Nardi, MD, funds at Massachusetts General Hospital.
David J. Van Den Berg was supported in part by grant HHSN268201500017C.
Stella Aslibekyan was supported in part by the grant K01HL136700.
D.C. Rao was supported in part by grants R01HL111249 and R01HL111249-04S1.
Donald W. Bowden and Nicholette D. Palmer were supported in part by grants R01 HL92301, R01 HL67348, R01 NS058700, R01 NS075107, R01 AR48797, R01 DK071891, M01 RR07122, F32 HL085989, and P60 AG10484.
James G. Wilson was supported in part by grant U54GM115428.

James B. Meigs was supported in part by grants U01 DK078616 and K24 DK080140.
Daniel E Weeks was supported in part by grants R01 HL093093 and R01 HL1333040.
David D. McManus was supported in part by grants R01HL126911, R01HL137734, R01HL137794, R01HL135219, R01HL136660, and U54HL143541.
Adolfo Correa was supported in part by grants HHSN268201800010I, HHSN268201800011I, HHSN268201800012I, HHSN268201800013I, HHSN268201800014I, and HHSN268201800015I.
R. Graham Barr was supported in part by grant R01 HL077612.
Hyun Min Kang was supported in part by grant U01HL137182.
John Blangero and Joanne E. Curran were supported in part by grants HL045522, MH078143, MH078111, MH083824, DK085524, and HL113323.
Dawood Darbar was supported in part by grant R01 HL138737.
Ramachandran S. Vasan was supported in part by grants NO1-HC-25195, HHSN268201500001I, R01 HL092577-06S1, Evans Medical Foundation, and the Jay and Louis Coffman Endowment from the Department of Medicine, Boston University School of Medicine.
Scott Vrieze was supported in part by grants R01 DA 037904, R01 HG 008983, R21 DA 040177, and R01 DA 044283.
Esteban G. Burchard was supported in part by Sandler Family Foundation, the American Asthma Foundation, the RWJF Amos Medical Faculty Development Program, the Harry Wm. and Diana V. Hind Distinguished Professor in Pharmaceutical Sciences II, U01HL138626, R01HL117004, R01HL128439, R01HL135156, X01HL134589, R01HL141992, R01HL141845, U01HG009080, R01ES015794, R21ES24844, P60MD006902, R01MD010443, RL5GM118984, R56MD013312, 24RT-0025 and 27IR-0030.
Emelia J. Benjamin was supported in part by grants R01HL128914, 2R01 HL092577, 2U54HL120163, and American Heart Association 18SFRN34110082.
Pradeep Natarajan was supported in part by grants K08HL140203 and R01HL142711.
Vivien A. Sheehan was supported in part by grant 5K08DK110448-03.
Steven A. Lubitz was supported by NIH grant 1R01HL139731 and American Heart Association 18SFRN34250007.
Edwin K. Silverman was supported in part by grants U01 HL089856, U01 HL089897, R01 HL113264, and P01 HL114501.
Sudha Seshadri was supported in part by grants R01 AG054076, R01 AG052409, and AG059421.
Nancy L. Heard-Costa was supported in part by grants NO1-HC-25195, HHSN268201500001I, and R01HL092577-06S1.
Michael H. Cho was supported in part by grants U01 HL089856 and U01 HL089897.
Gina M. Peloso was supported in part by grants K01HL125751, R03HL141439, and R01HL142711.
Mina K. Chung was supported in part by grants R01 HL 090620 and R01 HL 111314, NIH National Center for Research Resources for Case Western Reserve University and Cleveland Clinic Clinical and Translational Science Award UL1-RR024989, Cleveland Clinic Department of

Cardiovascular Medicine philanthropy research funds, and Tomsich Atrial Fibrillation Research Fund.
Xiaowen Tian, Brian L. Browning and Sharon R. Browning were supported in part by grant HG005701.
Sebastian Zöllner was supported in part by grant R01 HG005855.
Mariza de Andrade was supported in part by grants R01 HL66216 and R01 HL83141.
Achilleas N. Pitsillides and L Adrienne Cupples were supported in part by grants NO1-HC-25195, HHSN268201500001I, and R01 HL092577-06S1.
Deborah A. Nickerson was supported in part by grants HHSN268201600032I and S10OD021553.
Lu-Chen Weng was supported in part by American Heart Association grant 17POST33660226 and grant 18SFRN34110082.
Brian E. Cade was supported in part by grant K01HL135405.
Nora Franceschini was supported in part by grants R01-MD012765, R01 DK117445-01A1, and R21-HL140385.
Douglas P. Kiel was supported in part by grants R01 AR072199 and R01 AR041398.
Weihong Tang was supported in part by grant R01HL059367, NHLBI contracts HHSN268201700001I, HHSN268201700002I, HHSN268201700003I, HHSN268201700004I, and HHSN268201700005I.
Zachary A. Szpiech, Raul Torres, and Ryan D. Hernandez were supported in part by grant R01HG007644.
Bruce M. Psaty was supported in part by grants HL120393 and HL130114.
Kathryn L. Lunetta was supported in part by grants R01 HL092577-10 and 18SFRN34110082.
Brian Custer and Shannon Kelly were supported in part by grant HHSN268201100007I.
Stephen T. McGarvey was supported in part by grant HL093093.
Kenneth M. Rice was supported in part by grants OT3HL142478, R01 HL120393, U01 HL 137162, HHSN268201800001I, and HHSN26800001.
Lawrence F. Bielak, Sharon L.R. Kardia, Patricia A. Peyser, and Jennifer A. Smith were supported in part by grants U10 HL054457, U10 HL054464, U10 HL054481, R01 HL087660, R01 HL085571, and R01 HL119443.
Jiang He was supported in part by grants U01HL072507, R01HL087263, and R01HL090682.
Amol C. Shetty, Braxton D. Mitchell, Timothy D. O'Connor were supported in part by grants U01 HL137181-01, R01 HG002898-09A, R01 HL121007-01, OT3 OD025459-01, and R35 HG010692-01.
Yingze Zhang was supported in part by grant HHSN268200617182C.
Lewis C. Becker and Rasika A. Mathias were supported in part by grants U01 HL72518, HL087698, HL112064, HL11006, HL118356 and M01-RR000052.
Alexander P. Reiner was supported in part by grants R01HL132947, R01HL136574, R01HL129132, and RO1HL130733.
Ruth J.F. Loos was supported in part by grants X01HL134588, R01DK110113, and R01DK107786.

Daniel N. Harris, Michael D. Kessler, and Douglas P. Loesch were supported in part by grants U01 HL137181-01, R01 HG002898-09A, R01 HL121007-01, OT3 OD025459-01, R35 HG010692-01, and T32HL007698.
Susan Redline was supported in part by grants R35 HL 135818, HL 046389, and HL 113338.
Jessica Lasky-Su was supported in part by grant 1 P01 HL13285.
André Corvelo, Wayne E. Clarke, and Michael C. Zody were supported in part by a Centers for Common Disease Genomics grant from the National Human Genome Research Institute (UM1HG008901) and by the Alfred P. Sloan Foundation.
Donna K. Arnett was supported in part by grant U01HL72524.
Alvaro Alonso was supported in part by grant K24HL148521.
Dan E. Arking was supported in part by grants 1R01HL141989, 1R01HL14469.
Angel C.Y. Mak was supported in part by grants R01MD010443, R01HL135156, R01HL141845, R01HL117004 and U01HL138626.
Nicholas L. Smith was supported in part by grants HL73410 and HL95080.

4 Ethics statement

Amish

All study protocols were approved by the institutional review board at the University of Maryland Baltimore. Informed consent was obtained from each study participant.

AustralianFamilialAF

The Australian Familial AF cohort is comprised of probands with a positive family history of atrial fibrillation. All subjects have provided informed written consent and have undergone medical history, ECG and echocardiogram, and blood or buccal samples collected for genetics analyses. Study protocols have been reviewed by the St Vincent's Hospital Human Research Ethics Committee.

BAGS

NIH guidelines for conducting human genetic research were followed. The Institutional Review Boards (IRB) of Johns Hopkins University (GRAAD, BASS and BAGS), Howard University (CRAD and HUFS), Wake Forest University (SARP), the University of California, San Francisco (coordinating center for the SAGE II and GALA II studies), the Western Institutional Review Board for the recruitment in Puerto Rico (GALA II Puerto Ricans), Children's Hospital and Research Center Oakland and Kaiser Permanente-Vallejo Medical Center (SAGE II), the University of Chicago (CAG), University of the West Indies, Mona, Jamaica and Cave Hill Campus, Barbados (BAGS), University of Mississippi Medical Center (JHS), Henry Ford Health System (SAPPHIRE), the Universidad Católica de Honduras in San Pedro Sula (HONDAS), Federal University of Bahia (BIAS and ProAR), the University of Cartagena (PGCA), all reviewed and approved this study. All participants provided written informed consent.

BioMe

The BioMe cohort was approved by the Institutional Review Board at the Icahn School of Medicine at Mount Sinai. All BioMe participants provided written, informed consent for genomic data sharing.

CARDIA

All CARDIA participants provided informed consent, and the study was approved by the Institutional Review Boards of the University of Alabama at Birmingham and the University of Texas Health Science Center at Houston.

CFS

Cleveland Family Study was approved by the Institutional Review Board (IRB) of Case Western Reserve University and Mass General Brigham (formerly Partners HealthCare). Written informed consent was obtained from all participants.

CHS

All CHS participants provided informed consent, and the study was approved by the Institutional Review Board [or ethics review committee] of University Washington.

COPDGene

All COPDGene participants provided written informed consent, and the study was approved by the Institutional Review Boards of the participating clinical centers.

CRA

All Genetics of Asthma in Costa Rica parents provided informed consent and all children provided assent for participation in the study and the study was approved by the Institutional Review Board of the Hospital de Ninos in San Juan, Costa Rica and by the Institutional Review Board of Brigham and Women's Hospital, Boston MA.

DHS

Written informed consent was obtained from all participants and all study protocols were approved by the WFSM institutional review board.

EOCOPD

All Boston Early-Onset COPD Study participants provided written informed consent, and the study was approved by the Institutional Review Board at Brigham and Women's Hospital.

FHS

The Framingham Heart Study was approved by the Institutional Review Board of the Boston University Medical Center. All study participants provided written informed consent.

GeneSTAR

All participants provided written informed consent and the study was approved by the Johns Hopkins Medicine Institutional Review Board.

GENOA

Written informed consent was obtained from all subjects and approval was granted by participating institutional review boards (University of Michigan, University of Mississippi Medical Center, and Mayo Clinic).

GenSalt

All subjects provided informed consent and the GenSalt study was approved by the Institutional Review Board (IRB) of all participating institutes in the US and China.

GOLDN

All GOLDN participants provided informed consent, and the study was approved by the Institutional Review Board of the University of Kentucky.

HCHS_SOL
This study was approved by the institutional review boards (IRBs) at each field center, where all participants gave written informed consent, and by the Non-Biomedical IRB at the University of North Carolina at Chapel Hill, to the HCHS/SOL Data Coordinating Center. All IRBs approving the study are: Non-Biomedical IRB at the University of North Carolina at Chapel Hill. Chapel Hill, NC; Einstein IRB at the Albert Einstein College of Medicine of Yeshiva University. Bronx, NY; IRB at Office for the Protection of Research Subjects (OPRS), University of Illinois at Chicago. Chicago, IL; Human Subject Research Office, University of Miami. Miami, FL; Institutional Review Board of San Diego State University. San Diego, CA.

HVH
Study approval was granted by the human subjects committee at Group Health, and written informed consent was provided by all study participants.

HyperGEN
All HyperGEN participants provided informed consent, and the study was approved by the Institutional Review Board of the University of Kentucky.

JHS
The JHS study was approved by Jackson State University, Tougaloo College, and the University of Mississippi Medical Center IRBs, and all participants provided written informed consent.

LTRC
All LTRC participants provided written informed consent, and the study was approved by the Institutional Review Boards of the participating clinical centers.

Mayo_VTE
All Mayo_VTE participants provided informed consent and the study was approved by the Institutional Review Board of Mayo Clinic, Rochester, MN.

MESA
All MESA participants provided written informed consent, and the study was approved by the Institutional Review Boards at The Lundquist Institute (formerly Los Angeles BioMedical Research Institute) at Harbor-UCLA Medical Center, University of Washington, Wake Forest School of Medicine, Northwestern University, University of Minnesota, Columbia University, and Johns Hopkins University.

OMG_SCD

All OMG-SCD participants provided informed consent, and the study was approved by the Institutional Review Board of Duke University.

SAFS

All SAFS participants provided informed consent, and the study was approved by Institutional Review Board at the University of Texas Rio Grande Valley.

Sarcoidosis

All sarcoidosis study participants provided informed consent, and the study was approved by the IRB at the Oklahoma Medical Research Foundation.

Samoan

All Samoan study participants provided written informed consent, and the study was approved by the Institutional Review Board at Brown University, and the Health Research Committee of the Samoa Ministry of Health.

THRV

All THRV participants provided informed consent, and the study was approved by the Institutional Review Board at The Lundquist Institute (formerly Los Angeles BioMedical Research Institute) at Harbor-UCLA Medical Center. All THRV participants provided informed consent, and the study was approved by the Institutional Review Board at Washington University in St. Louis.

walk_PHaSST

All walk-PHaSST participants provided informed consent, and the study was approved by the Institutional Review Board of all clinical sites (Albert Einstein College of Medicine, Children's Hospital of Oakland, Children's Hospital Pittsburgh, Howard University Hospital, Imperial College London and Hammersmith Hospital, Johns Hopkins University, NIH Clinical Center, University of Colorado, University of Illinois at Chicago) and of the University of Pittsburgh for the walk-PHaSST Biorepository.

WHI

All WHI participants provided informed consent and the study was approved by the Institutional Review Board (IRB) of the Fred Hutchinson Cancer Research Center.

5 References

1. NHLBI Trans-omics for Precision Medicine. Analysis Projects. https://www.nhlbiwgs.org/awards.
2. GENESIS. *Bioconductor* https://bioconductor.org/packages/release/bioc/html/GENESIS.html.
3. Kang, H. M. EPACTS Software. http://csg.sph.umich.edu/kang/epacts/download/index.html.
4. NHLBI Trans-Omics for Precision Medicine. 2018 DCC Analysis Workshop in Seattle. https://www.nhlbiwgs.org/2018-seattle-workshop.
5. PAR-16-021: NHLBI TOPMed: Omics Phenotypes of Heart, Lung, and Blood Disorders (X01). https://grants.nih.gov/grants/guide/pa-files/PAr-16-021.html.
6. Wang, S. R. *et al.* Simulation of Finnish population history, guided by empirical genetic data, to assess power of rare-variant tests in Finland. *Am. J. Hum. Genet.* **94**, 710–720 (2014).
7. Jun, G. *et al.* Evaluating the contribution of rare variants to type 2 diabetes and related traits using pedigrees. *Proc. Natl. Acad. Sci. U. S. A.* **115**, 379–384 (2018).
8. Bureau, A. *et al.* Inferring disease risk genes from sequencing data in multiplex pedigrees through sharing of rare variants. *Genet. Epidemiol.* (2018) doi:10.1002/gepi.22155.
9. Spielman, R. S., McGinnis, R. E. & Ewens, W. J. Transmission test for linkage disequilibrium: the insulin gene region and insulin-dependent diabetes mellitus (IDDM). *Am. J. Hum. Genet.* **52**, 506–516 (1993).
10. NHLBI Trans-Omics for Precision Medicine. TOPMed Projects and their Parent Studies. https://www.nhlbiwgs.org/group/project-studies.

11. Fortier, I. *et al.* Maelstrom Research guidelines for rigorous retrospective data harmonization. *Int. J. Epidemiol.* **46**, 103–105 (2017).

12. Stilp, A. M. *et al.* A system for phenotype harmonization in the NHLBI Trans-Omics for Precision Medicine (TOPMed) Program. 2020.06.18.146423 (2020) doi:10.1101/2020.06.18.146423.

13. Oelsner, E. C. *et al.* Harmonization of Respiratory Data From 9 US Population-Based Cohorts: The NHLBI Pooled Cohorts Study. *Am. J. Epidemiol.* **187**, 2265–2278 (2018).

14. Popejoy, A. B. & Fullerton, S. M. Genomics is failing on diversity. *Nature News* **538**, 161 (2016).

15. Guidelines on the use and reporting of race, ethnicity, and ancestry in the NHLBI Trans-Omics for Precision Medicine (TOPMed) program. https://www.nhlbiwgs.org/guidelines-use-and-reporting-race-ethnicity-and-ancestry-topmed.

16. Conomos, M. P., Miller, M. B. & Thornton, T. A. Robust inference of population structure for ancestry prediction and correction of stratification in the presence of relatedness. *Genet. Epidemiol.* **39**, 276–293 (2015).

17. Conomos, M. P., Reiner, A. P., Weir, B. S. & Thornton, T. A. Model-free Estimation of Recent Genetic Relatedness. *Am. J. Hum. Genet.* **98**, 127–148 (2016).

18. Krzywinski, M. *et al.* Circos: an information aesthetic for comparative genomics. *Genome Res.* **19**, 1639–1645 (2009).

19. TOPMed RNA-seq Pipeline Harmonization Summary. https://www.nhlbiwgs.org/sites/default/files/TOPMed_RNAseq_pipeline_COREyr2.pdf.

20. Rohloff, J. C. *et al.* Nucleic Acid Ligands With Protein-like Side Chains: Modified Aptamers and Their Use as Diagnostic and Therapeutic Agents. *Mol. Ther. Nucleic Acids* **3**, e201

(2014).

21. The SOMAscan® Platform. *SomaLogic* https://somalogic.com/technology/our-platform/.
22. Infinium(R) MethylationEPIC BeadChip,. *Illumina* https://www.illumina.com/content/dam/illumina-marketing/documents/products/datasheets/humanmethylationepic-data-sheet-1070-2015-008.pdf.
23. NHLBI Trans-Omics for Precision Medicine. TOPMed Data Access for the Scientific Community. https://www.nhlbiwgs.org/topmed-data-access-scientific-community.
24. Bravo. https://bravo.sph.umich.edu/freeze5/hg38/.
25. Bravo. Variants in dbSNP. https://bravo.sph.umich.edu/freeze5/hg38/download.
26. National Heart *et al.* The NHLBI BioData Catalyst. (2020) doi:10.5281/zenodo.3822858.
27. TOPMed Imputation Server. https://imputation.biodatacatalyst.nhlbi.nih.gov.
28. 1000 Genomes Project Consortium *et al.* A global reference for human genetic variation. *Nature* **526**, 68–74 (2015).
29. McCarthy, S. *et al.* A reference panel of 64,976 haplotypes for genotype imputation. *Nat. Genet.* **48**, 1279–1283 (2016).
30. NHLBI Trans-Omics for Precision Medicine. ELSI. https://www.nhlbiwgs.org/elsi.
31. Mahalanobis, P. C. On the Generalised Distance in Statistics. *Proceedings of the National Institute of Sciences of India* **2**, 49–55 (1936).
32. SeqVarTools. http://bioconductor.org/packages/SeqVarTools/.
33. Gogarten, S. M. *et al.* Genetic association testing using the GENESIS R/Bioconductor package. *Bioinformatics* (2019) doi:10.1093/bioinformatics/btz567.
34. Liu, X. *et al.* WGSA: an annotation pipeline for human genome sequencing studies. *J. Med. Genet.* **53**, 111–112 (2016).

35. UW-GAC. UW-GAC/wgsaparsr. https://github.com/UW-GAC/wgsaparsr.
36. Psaty, B. M. *et al.* Cohorts for Heart and Aging Research in Genomic Epidemiology (CHARGE) Consortium: Design of prospective meta-analyses of genome-wide association studies from 5 cohorts. *Circ. Cardiovasc. Genet.* **2**, 73–80 (2009).
37. Reid, J. G. *et al.* Launching genomics into the cloud: deployment of Mercury, a next generation sequence analysis pipeline. *BMC Bioinformatics* **15**, 30 (2014).
38. Huang, Z. *et al.* A hybrid computational strategy to address WGS variant analysis in >5000 samples. *BMC Bioinformatics* **17**, 361 (2016).
39. McKenna, A. *et al.* The Genome Analysis Toolkit: A MapReduce framework for analyzing next-generation DNA sequencing data. *Genome Research* vol. 20 1297–1303 (2010).
40. Jun, G., Wing, M. K., Abecasis, G. R. & Kang, H. M. An efficient and scalable analysis framework for variant extraction and refinement from population-scale DNA sequence data. *Genome Research* vol. 25 918–925 (2015).
41. Wang, Y., Lu, J., Yu, J., Gibbs, R. A. & Yu, F. An integrative variant analysis pipeline for accurate genotype/haplotype inference in population NGS data. *Genome Res.* **23**, 833–842 (2013).
42. Protocols: Sequencing Library Construction. *BCM-HGSC* https://www.hgsc.bcm.edu/content/protocols-sequencing-library-construction (2016).
43. Mercury. *BCM-HGSC* https://www.hgsc.bcm.edu/software/mercury (2014).
44. Chan, K. & Gordenin, D. A. Clusters of Multiple Mutations: Incidence and Molecular Mechanisms. *Annu. Rev. Genet.* **49**, 243–267 (2015).
45. Tian, D. *et al.* Single-nucleotide mutation rate increases close to insertions/deletions in eukaryotes. *Nature* **455**, 105 (2008).

46. Martin, M. Cutadapt removes adapter sequences from high-throughput sequencing reads. *EMBnet.journal* **17**, 10 (2011).

47. Marco-Sola, S., Sammeth, M., Guigó, R. & Ribeca, P. The GEM mapper: fast, accurate and versatile alignment by filtration. *Nat. Methods* **9**, 1185–1188 (2012).

48. Jackman, S. D. *et al.* ABySS 2.0: resource-efficient assembly of large genomes using a Bloom filter. *Genome Res.* **27**, 768–777 (2017).

49. Kent, W. J. *et al.* The Human Genome Browser at UCSC. *Genome Res.* **12**, 996–1006 (2002).

50. Li, H. Aligning sequence reads, clone sequences and assembly contigs with BWA-MEM. *arXiv preprint* (2013).

51. Kuhn, R. M., Haussler, D. & Kent, W. J. The UCSC genome browser and associated tools. *Brief. Bioinform.* **14**, 144–161 (2013).

52. Abyzov, A. & Gerstein, M. AGE: defining breakpoints of genomic structural variants at single-nucleotide resolution, through optimal alignments with gap excision. *Bioinformatics* **27**, 595–603 (2011).

53. Frankish, A. *et al.* GENCODE reference annotation for the human and mouse genomes. *Nucleic Acids Res.* **47**, D766–D773 (2019).

54. Karolchik, D. *et al.* The UCSC Table Browser data retrieval tool. *Nucleic Acids Res.* **32**, D493–D496 (2004).

55. Quinlan, A. R. & Hall, I. M. BEDTools: a flexible suite of utilities for comparing genomic features. *Bioinformatics* **26**, 841–842 (2010).

56. Smit, A., Hubley, R. & Green, P. *RepeatMasker Open-4.0. 2013–2015*. (2015).

57. Chen, S. *et al.* Paragraph: A graph-based structural variant genotyper for short-read

sequence data. *bioRxiv* 635011 (2019) doi:10.1101/635011.

58. Gutenkunst, R. N., Hernandez, R. D., Williamson, S. H. & Bustamante, C. D. Diffusion Approximations for Demographic Inference: DaDi. (2010) doi:10.1038/npre.2010.4594.1.

59. Keinan, A. & Clark, A. G. Recent Explosive Human Population Growth Has Resulted in an Excess of Rare Genetic Variants. *Science* **336**, 740–743 (2012).

60. Tennessen, J. A. *et al.* Evolution and Functional Impact of Rare Coding Variation from Deep Sequencing of Human Exomes. *Science* **337**, 64–69 (2012).

61. Alexander, D. H., Novembre, J. & Lange, K. Fast model-based estimation of ancestry in unrelated individuals. *Genome Res.* **19**, 1655–1664 (2009).

62. Zhou, H., Alexander, D. & Lange, K. A quasi-Newton acceleration for high-dimensional optimization algorithms. *Stat. Comput.* **21**, 261–273 (2011).

63. Wollstein, A. *et al.* Demographic history of Oceania inferred from genome-wide data. *Curr. Biol.* **20**, 1983–1992 (2010).

64. Lipson, M. *et al.* Population Turnover in Remote Oceania Shortly after Initial Settlement. *Curr. Biol.* **28**, 1157–1165.e7 (2018).

65. Maples, B. K., Gravel, S., Kenny, E. E. & Bustamante, C. D. RFMix: a discriminative modeling approach for rapid and robust local-ancestry inference. *Am. J. Hum. Genet.* **93**, 278–288 (2013).

66. Li, J. Z. *et al.* Worldwide human relationships inferred from genome-wide patterns of variation. *Science* **319**, 1100–1104 (2008).

67. Torres, R., Szpiech, Z. A. & Hernandez, R. D. Human demographic history has amplified the effects of background selection across the genome. *PLoS Genet.* **14**, e1007387 (2018).

68. Pollard, K. S., Hubisz, M. J., Rosenbloom, K. R. & Siepel, A. Detection of nonneutral

substitution rates on mammalian phylogenies. *Genome Res.* **20**, 110–121 (2010).

69. Siepel, A. Evolutionarily conserved elements in vertebrate, insect, worm, and yeast genomes. *Genome Res.* **15**, 1034–1050 (2005).

70. ENCODE Project Consortium. An integrated encyclopedia of DNA elements in the human genome. *Nature* **489**, 57–74 (2012).

71. Bailey, J. A., Yavor, A. M., Massa, H. F., Trask, B. J. & Eichler, E. E. Segmental duplications: organization and impact within the current human genome project assembly. *Genome Res.* **11**, 1005–1017 (2001).

72. Szpiech, Z. A. & Hernandez, R. D. selscan: an efficient multithreaded program to perform EHH-based scans for positive selection. *Mol. Biol. Evol.* **31**, 2824–2827 (2014).

73. Capra, J. A., Hubisz, M. J., Kostka, D., Pollard, K. S. & Siepel, A. A model-based analysis of GC-biased gene conversion in the human and chimpanzee genomes. *PLoS Genet.* **9**, e1003684 (2013).

74. International HapMap Consortium *et al.* A second generation human haplotype map of over 3.1 million SNPs. *Nature* **449**, 851–861 (2007).

75. Pratto, F. *et al.* DNA recombination. Recombination initiation maps of individual human genomes. *Science* **346**, 1256442 (2014).

76. McVicker, G., Gordon, D., Davis, C. & Green, P. Widespread genomic signatures of natural selection in hominid evolution. *PLoS Genet.* **5**, e1000471 (2009).

77. Charlesworth, B., Morgan, M. T. & Charlesworth, D. The effect of deleterious mutations on neutral molecular variation. *Genetics* **134**, 1289–1303 (1993).

78. Nordborg, M., Charlesworth, B. & Charlesworth, D. The effect of recombination on background selection. *Genet. Res.* **67**, 159 (1996).

79. Charlesworth, B. The effects of deleterious mutations on evolution at linked sites. *Genetics* **190**, 5–22 (2012).

80. Flicek, P. *et al.* Ensembl 2013. *Nucleic Acids Res.* **41**, D48–55 (2013).

81. Paten, B. *et al.* Genome-wide nucleotide-level mammalian ancestor reconstruction. *Genome Res.* **18**, 1829–1843 (2008).

82. Paten, B., Herrero, J., Beal, K., Fitzgerald, S. & Birney, E. Enredo and Pecan: genome-wide mammalian consistency-based multiple alignment with paralogs. *Genome Res.* **18**, 1814–1828 (2008).

83. Wang, K., Li, M. & Hakonarson, H. ANNOVAR: functional annotation of genetic variants from high-throughput sequencing data. *Nucleic Acids Res.* **38**, e164 (2010).

84. Jouganous, J., Long, W., Ragsdale, A. P. & Gravel, S. Inferring the Joint Demographic History of Multiple Populations: Beyond the Diffusion Approximation. *Genetics* **206**, 1549–1567 (2017).

85. Palamara, P. F. *et al.* Leveraging Distant Relatedness to Quantify Human Mutation and Gene-Conversion Rates. *Am. J. Hum. Genet.* **97**, 775–789 (2015).

86. Coffman, A. J., Hsieh, P. H., Gravel, S. & Gutenkunst, R. N. Computationally Efficient Composite Likelihood Statistics for Demographic Inference. *Mol. Biol. Evol.* **33**, 591–593 (2016).

87. Field, Y. *et al.* Detection of human adaptation during the past 2000 years. *Science* **354**, 760–764 (2016).

88. Gravel, S. *et al.* Demographic history and rare allele sharing among human populations. *Proc. Natl. Acad. Sci. U. S. A.* **108**, 11983–11988 (2011).
