# Supplementary material for: Sequencing of 53,831 diverse genomes from the NHLBI TOPMed Program
Source: Nature. Author manuscript; Available in PMC 2021 Oct 6. (PMC7875770; doi:10.1038/s41586-021-03205-y)
Supplement: 1675193_SuppTablesandFigures [file NIHMS1675193-supplement-1675193_SuppTablesandFigures.pdf]

SUPPLEMENTARY TABLES AND FIGURES

Sequencing of 53,831 diverse genomes from the NHLBI TOPMed Program

Supplementary Table 1. Autosomal variants identified using whole genome sequencing (WGS) and whole exome sequencing (WES) in all protein coding regions defined by GENCODE.

		SNVs Per Individual				Indels Per Individual					
		N	% genotype concordant	% bp >20X in WGS	% bp >20X in WES	N	% genotype concordant	% bp >20X in WGS	% bp >20X in WES	% Del.	Size >3 bp
						GENCODE coding regions: all MAF					
**WGS**	**All Variants**	23,886	--	97.20%	83.20%	282	--	92.11%	69.52%	72.70%	71
	**Also in WES**	19,655	99.93%	97.49%	89.13%	134	99.74%	93.51%	89.13%	77.61%	26
	**Only in WGS**	1,384	--	94.47%	6.08%	57	--	88.16%	9.68%	68.42%	17
	**Failed in WES**	2,846	99.65%	96.50%	79.54%	90	95.90%	92.51%	77.90%	68.89%	29
**WES**	**All Variants**	20,530	--	97.04%	89.24%	191	--	91.38%	88.43%	65.97%	36
	**Also in WGS**	19,655	99.93%	97.49%	89.13%	134	99.74%	93.51%	89.13%	77.61%	26
	**Only in WES**	83	--	77.05%	80.35%	16	--	77.23%	78.09%	56.25%	2
	**Failed in WGS**	792	96.84%	87.98%	92.85%	41	96.29%	90.10%	90.23%	31.71%	9
						GENCODE coding regions: MAF ≤ 1%					
**WGS**	**All Variants**	1,591	--	97.47%	84.92%	32	--	92.98%	74.41%	65.63%	8
	**Also in WES**	1,353	99.99%	97.61%	89.08%	20	99.96%	93.71%	85.56%	65.00%	3
	**Only in WGS**	73	--	95.52%	7.57%	6	--	89.56%	19.21%	50.00%	2
	**Failed in WES**	166	99.90%	97.26%	84.43%	7	99.81%	94.11%	86.60%	71.43%	2
**WES**	**All Variants**	1,403	--	97.24%	88.37%	26	--	92.34%	82.34%	61.54%	5
	**Also in WGS**	1,351	99.99%	97.60%	89.02%	20	99.96%	93.66%	85.17%	65.00%	4
	**Only in WES**	16	--	88.66%	43.55%	3	--	84.03%	53.95%	66.67%	1
	**Failed in WGS**	36	99.66%	87.35%	85.50%	3	99.53%	93.27%	91.46%	33.33%	1

Supplementary Table 2. Autosomal variants identified using whole genome sequencing (WGS) and whole exome sequencing (WES) in all protein coding regions targeted by WES.

		SNVs Per Individual				Indels Per Individual					
		N	% genotype concordant	% bp >20X in WGS	% bp >20X in WES	N	% genotype concordant	% bp >20X in WGS	% bp >20X in WES	% Del.	Size >3 bp
						**Coding regions targeted in WES: all MAF**					
**WGS**	**All Variants**	22,316	--	97.26%	88.20%	213	--	92.71%	86.36%	75.59%	55
	**Also in WES**	19,432	99.93%	97.49%	89.46%	125	99.73%	93.25%	90.10%	79.20%	25
	**Only in WGS**	160	--	81.44%	37.20%	8	--	85.53%	51.79%	87.50%	5
	**Failed in WES**	2,724	99.67%	96.53%	82.13%	79	95.69%	92.58%	84.01%	69.62%	26
**WES**	**All Variants**	20,275	--	97.04%	89.55%	181	--	91.22%	89.35%	66.30%	35
	**Also in WGS**	19,432	99.93%	97.49%	89.46%	125	99.73%	93.25%	90.10%	79.20%	25
	**Only in WES**	81	--	76.83%	81.23%	15	--	78.39%	80.32%	60.00%	2
	**Failed in WGS**	761	96.84%	87.78%	92.75%	41	96.31%	90.03%	90.54%	29.27%	9
						**Coding regions targeted in WES: MAF ≤ 1%**					
**WGS**	**All Variants**	1,505	--	97.52%	88.85%	26	--	92.82%	86.48%	73.08%	7
	**Also in WES**	1,334	99.99%	97.60%	89.46%	18	99.96%	93.50%	88.90%	72.22%	3
	**Only in WGS**	10	--	90.05%	41.83%	2	--	82.40%	57.29%	50.00%	1
	**Failed in WES**	161	99.91%	97.27%	86.62%	6	99.81%	94.09%	87.80%	83.33%	2
**WES**	**All in WES**	1,383	--	97.24%	88.80%	24	--	92.25%	85.30%	62.50%	5
	**Also in WGS**	1,332	99.99%	97.60%	89.41%	18	99.96%	93.43%	88.53%	72.22%	3
	**Only in WES**	15	--	88.47%	45.47%	3	--	84.59%	56.81%	66.67%	0
	**Failed in WGS**	35	99.66%	87.37%	85.72%	3	99.51%	93.70%	92.15%	33.33%	1

Supplementary Table 3. Number of exonic SNVs discovered by different sequencing experiments in Framingham Heart Study. Categories are not mutually exclusive.

Study	Exonic SNVs	Loss-of-Function	Missense	Non-synonymous
TOPMed	212,603	2,047	125,862	127,834
CHARGE WGS (depth <6X)	182,165	1,581	105,588	107,283
CHARGE WES (depth >30X)	180,520	1,742	108,481	109,849

Supplementary Table 4. Autosomal variants discovered by our pipeline and by GATK standard "best practices" pipeline. [a]Counted alleles which pass or fail quality checks.

		SNVs					Indels				
		Avg. per individual	Avg. % genotype concordant	Avg. per trio	Avg. % w/ Mendelian errors	Avg. alleles[a] within +/-50bp	Avg. per individual	Avg. % genotype concordant	Avg. per trio	Avg. % w/ Mendelian errors	Avg. alleles[a] within +/-50bp
						All MAF					
**vt**	**All Variants**	3,408,493	--	4,632,934	0.04%	1.36	186,795	--	261,355	0.22%	1.24
	**Also in GATK**	3,377,771	99.94%	4,590,260	0.04%	1.35	185,796	99.37%	259,865	0.20%	1.24
	**Only in vt**	5,815	--	9,507	1.84%	2.18	690	--	1,024	4.73%	1.93
	**Failed in GATK**	24,669	99.05%	33,167	0.23%	1.66	309	89.21%	466	2.54%	1.58
**GATK**	**All Variants**	3,594,828	--	4,924,665	0.52%	1.40	775,110	--	1,130,696	3.56%	1.68
	**Also in vt**	3,377,771	99.94%	4,590,260	0.04%	1.33	185,796	99.37%	259,865	0.11%	1.24
	**Only in GATK**	24,639	--	53,186	27.59%	3.59	73,215	--	115,830	12.51%	3.12
	**Failed in vt**	192,418	94.44%	281,218	3.08%	2.46	516,099	84.07%	755,001	3.37%	1.63
						MAF ≤ 1%					
**vt**	**All Variants**	192,770	--	310,347	0.08%	1.37	10,267	--	18,693	0.20%	1.22
	**Also in GATK**	190,561	99.97%	306,610	0.07%	1.37	10,084	99.91%	18,417	0.19%	1.21
	**Only in vt**	284	--	591	3.10%	2.22	149	--	215	1.22%	1.62
	**Failed in GATK**	1,925	98.71%	3,147	0.37%	1.56	33	89.84%	61	1.39%	1.58
**GATK**	**All Variants**	215,196	--	360,092	3.47%	1.39	35,957	--	67,701	9.05%	1.87
	**Also in vt**	189,928	99.99%	305,968	0.05%	1.24	10,072	99.89%	18,393	0.10%	1.20
	**Only in GATK**	12,551	--	30,635	29.62%	3.14	9,015	--	20,950	23.36%	3.11
	**Failed in vt**	12,717	98.05%	23,489	6.48%	2.02	16,870	93.64%	28,357	2.47%	1.61

Supplementary Table 5. Percent of singletons in regions of different functions. Percent of singletons was computed using unrelated individuals (N=40,722). Variant sites were split into non-CpG and CpG groups defined by 3-mer sequence context.

Category	% of variants at CpG sites (CI)	% of singletons (CI)		
		All sites	non-CpG sites	CpG sites
Intergenic	9.6 [9.62, 9.63]	53.03 [53.03,53.03]	55.69 [55.69,55.70]↓	28.02 [28.01,28.03]↓
Genome	10.64 [10.64,10.65]	53.11 [53.10,53.11]	55.94 [55.93,55.94]=	29.34 [29.33,29.34]=
Intronic	11.45 [11.45,11.46]	53.17 [53.16,53.17]	56.17 [56.17,56.17]↑	29.95 [29.94,29.96]↑
Open Chromatin	12.18 [12.17,12.19]	53.38 [53.36,53.40]	56.54 [56.52,56.56]↑	30.62 [30.58,30.66]↑
3' Untranslated Regions (UTR)	13.08 [13.06,13.09]	53.27 [53.24,53.29]	56.54 [56.52,56.57]↑	31.48 [31.43,31.54]↑
CTCF Binding Sites	13.56 [13.55,13.57]	52.29 [52.27,52.31]	56.13 [56.11,56.15]↑	27.81 [27.77,27.86]↓
Enhancers	16.02 [15.99,16.05]	53.07 [53.03,53.11]	56.57 [56.53,56.61]↑	34.73 [34.64,34.82]↑
Transcription Factor (TF) Binding Sites	19.55 [19.53,19.58]	52.73 [52.70,52.76]	56.85 [56.81,56.89]↑	35.78 [35.71,35.85]↑
Promoters	21.89 [21.85,21.93]	54.96 [54.91,55.00]	57.29 [57.23,57.34]↑	46.63 [46.52,46.73]↑
5' Untranslated Regions (UTR)	25.82 [25.79,25.86]	54.70 [54.67,54.74]	56.83 [56.79,56.88]↑	48.58 [48.51,48.66]↑
Coding Sequence (CDS)	28.04 [28.02,28.06]	54.08 [54.06,54.11]	60.05 [60.03,60.08]↑	38.77 [38.72,38.81]↑

Supplementary Table 6. Putative loss of function variants per individual in TOPMed Freeze 5 and ExAC data sets. To compare the number of putative loss of function (pLoF) variants per individual, we used only rare (AF < 0.5%) bi-allelic variants which were not present in dbSNP build 142 (last dbSNP database version without ExAC variants).

	**Exome Aggregation Consortium (ExAC)**				**TOPMed Freeze 5**			
	**% Singletons**	**Per Individual**	**Singletons**	**Total**	**% Singletons**	**Per Individual**	**Singletons**	**Total**
LoF								
all	73.00	5.51	114,621	157,006	63.90	7.83	110,815	173,428
frameshift	70.94	2.81	46,027	64,883	62.27	4.51	53,861	86,498
splice	76.25	1.09	28,596	37,501	66.76	1.39	24,167	36,198
stop_gained	73.23	1.61	39,998	54,622	64.63	1.94	32,787	50,732
Coding								
all	66.71	116.96	2,088,105	3,130,064	59.43	129.63	1,700,286	2,861,087
inframe	59.30	1.49	13,972	23,561	51.41	4.13	23,083	44,899
missense	67.62	72.88	1,390,186	2,056,022	60.24	79.17	1,109,809	1,842,262
synonymous	64.24	38.37	604,628	941,154	57.48	39.30	478,827	833,050

Supplementary Table 7. Enrichment and depletion of putative loss-of-function (pLoF) variants in gene sets. For each gene set we computed the number of rare (AF < 0.5%) pLoF variants per coding sequence base pair (pLoF/bp) and proportion of singletons. We compared observed pLoF/bp and proportion of singletons to 1,000,000 randomly sampled gene sets of same size and coding sequence length. P-value (bootstrap, two-sided) significance threshold is 2×10^{-6} after adjusting for multiple testing. BP - biological process, MF - molecular function, CC - cellular component.

Gene Sets		No. of Genes	pLoF / bp				Proportion of Singletons			
			Observed	Sample Mean	Ratio	P-value	Observed	Sample Mean	Ratio	P-value
Gene Ontology (GO)										
Class	Term									
MF	(GO:0043565) sequence-specific DNA binding	596	0.0033	0.0072	0.46	$<1\text{x}10^{-6}$	0.4956	0.4767	1.04	$<1\text{x}10^{-6}$
BP	(GO:0006413) translational initiation	142	0.0045	0.0083	0.55	$<1\text{x}10^{-6}$	0.5171	0.4803	1.08	$3\text{x}10^{-6}$
BP	(GO:0008380) RNA splicing	283	0.0048	0.0071	0.67	$<1\text{x}10^{-6}$	0.5056	0.4772	1.06	$<1\text{x}10^{-6}$
BP	(GO:0006397) mRNA processing	359	0.0048	0.0071	0.67	$<1\text{x}10^{-6}$	0.5085	0.4772	1.07	$<1\text{x}10^{-6}$
BP	(GO:0006357) regulation of transcription by RNA polymerase II	768	0.0043	0.0069	0.62	$<1\text{x}10^{-6}$	0.4911	0.4766	1.03	$<1\text{x}10^{-6}$
MF	(GO:0003700) DNA binding transcription factor activity	996	0.0048	0.0070	0.68	$<1\text{x}10^{-6}$	0.4900	0.4763	1.03	$<1\text{x}10^{-6}$
CC	(GO:0005654) nucleoplasm	3,135	0.0055	0.0070	0.79	$<1\text{x}10^{-6}$	0.4914	0.4768	1.03	$<1\text{x}10^{-6}$
CC	(GO:0030529) intracellular ribonucleoprotein complex	351	0.0063	0.0082	0.77	$<1\text{x}10^{-6}$	0.5000	0.4783	1.05	$<1\text{x}10^{-6}$
MF	(GO:0003723) RNA binding	1,491	0.0057	0.0071	0.79	$<1\text{x}10^{-6}$	0.4930	0.4771	1.03	$<1\text{x}10^{-6}$
BP	(GO:0006351) transcription, DNA-templated	2,364	0.0052	0.0068	0.75	$<1\text{x}10^{-6}$	0.4875	0.4764	1.02	$<1\text{x}10^{-6}$
BP	(GO:0006355) regulation of transcription, DNA-templated	2,537	0.0052	0.0069	0.76	$<1\text{x}10^{-6}$	0.4874	0.4764	1.02	$<1\text{x}10^{-6}$
CC	(GO:0005634) nucleus	6,262	0.0061	0.0071	0.86	$<1\text{x}10^{-6}$	0.4863	0.4769	1.02	$<1\text{x}10^{-6}$
CC	(GO:0005886) plasma membrane	4,620	0.0065	0.0070	0.94	$<1\text{x}10^{-6}$	0.4713	0.4766	0.99	$<1\text{x}10^{-6}$
BP	(GO:0055114) oxidation-reduction process	704	0.0094	0.0074	1.27	$<1\text{x}10^{-6}$	0.4620	0.4772	0.97	$<1\text{x}10^{-6}$
MF	(GO:0016491) oxidoreductase activity	577	0.0093	0.0072	1.29	$<1\text{x}10^{-6}$	0.4618	0.4767	0.97	$<1\text{x}10^{-6}$
Public Databases										
COSMIC genes		916	0.0048	0.0069	0.69	$<1\text{x}10^{-6}$	0.4846	0.4761	1.018	$9\text{x}10^{-5}$
GWAS Catalog upstream genes		1,917	0.0068	0.0075	0.91	$<1\text{x}10^{-6}$	0.4773	0.4766	1.001	$3\text{x}10^{-1}$

GWAS Catalog downstream genes	1,944	0.0069	0.0075	0.92	$<1x10^{-6}$	0.4764	0.4767	0.999	$4x10^{-1}$
GWAS Catalog genes	5,179	0.0067	0.0070	0.96	$4x10^{-5}$	0.4736	0.4761	0.995	$3x10^{-3}$
ClinVar genes with ≥1 pathogenic variants	3,893	0.0068	0.0071	0.96	$8x10^{-4}$	0.4717	0.4762	0.990	$2x10^{-5}$
ClinVar genes without pathogenic variants	2,103	0.0072	0.0070	1.02	$7x10^{-2}$	0.4703	0.4762	0.988	$3x10^{-5}$
OMIM genes	4,335	0.0069	0.0072	0.96	$5x10^{-5}$	0.4713	0.4763	0.990	$<1x10^{-6}$

Supplementary Table 8. Location of non-reference human sequences relative to various genomic features. When the reference allele overlaps multiple repeat categories, the corresponding event is counted multiple times. That is not the case for genic features as these were prioritized by potential impact over gene expression/product in the order shown in the table. Abbreviations: *ins*, insertions; *brk*, breakends.The reported *P*-values are from a two-sided chi-squared test.

	**Genome occupancy**	**N events *(ins/brk)***	**Fraction of events *(ins/brk)***	**Fold enrichment *(ins/brk)***	**χ2 raw P-value *(ins/brk)***
**Gene**	0.548	551 (387/164)	0.542 (0.543/0.539)	0.989 (0.990/0.984)	0.714 (0.809/0.810)
**Exonic**	0.045	26 (19/7)	0.026 (0.027/0.023)	0.572 (0.596/0.515)	0.004 (0.025/0.091)
**CDS**	0.012	4 (3/1)	0.004 (0.004/0.003)	0.326 (0.349/0.273)	0.026 (0.080/0.255)
**UTR**	0.033	22 (16/6)	0.022 (0.022/0.020)	0.663 (0.688/0.605)	0.059 (0.154/0.269)
**Promoter**	0.057	75 (42/33)	0.074 (0.059/0.109)	1.285 (1.026/1.891)	0.030 (0.926/2.1E-4)
**Intronic**	0.446	450 (326/124)	0.442 (0.457/0.408)	0.992 (1.025/0.915)	0.851 (0.568/0.202)
**Repeats**	0.501	576 (372/204)	0.566 (0.522/0.671)	1.131 (1.042/1.341)	3.1E-5 (0.274/3.9E-9)
**DNA**	0.033	11 (9/2)	0.011 (0.013/0.007)	0.326 (0.381/0.199)	1.0E-4 (0.003/0.015)
**LINE**	0.209	88 (70/18)	0.087 (0.098/0.059)	0.414 (0.470/0.283)	1.2E-21 (5.0E-13/2.2E-10)
**Low complexity**	0.002	10 (4/6)	0.010 (0.006/0.020)	4.952 (2.825/9.940)	1.4E-7 (0.080/2.8E-10)
**LTR**	0.088	48 (40/8)	0.047 (0.056/0.026)	0.538 (0.640/0.300)	6.6E-6 (0.004/2.3E-4)
**Other**	0.027	5 (5/0)	0.005 (0.007/0.000)	0.181 (0.258/0.000)	1.9E-5 (0.001/0.006)
**Simple repeat**	0.012	231 (145/86)	0.227 (0.203/0.283)	18.499 (16.563/23.040)	<2.2E-16 (<2.2E-16/<2.2E-16)
**SINE**	0.130	202 (117/85)	0.199 (0.164/0.280)	1.533 (1.266/2.158)	7.5E-11 (0.007/1.3E-14)
**Segmental duplications**	0.055	49 (32/17)	0.048 (0.045/0.056)	0.884 (0.823/1.026)	0.411 (0.293/1)

Supplementary Table 9. Repeat content of non-reference human sequences. "Single element" refers to the number/fraction of sequences consisting of, or containing, a single repetitive element annotated. Abbreviations: *ins* – insertions; *brk* – breakends.

Repeat content	N events *(ins/brk)*	Fraction of events *(ins/brk)*	Single element N *(ins/brk)*	Single element fraction *(ins/brk)*
**>=99%**	143 (85/58)	0.141 (0.119/0.191)	120 (71/49)	0.118 (0.100/0.161)
**75%-99%**	333 (204/129)	0.327 (0.286/0.424)	166 (107/59)	0.163 (0.150/0.194)
**50%-75%**	157 (111/46)	0.154 (0.156/0.151)	46 (39/7)	0.045 (0.055/0.023)
**25%-50%**	118 (97/21)	0.116 (0.136/0.069)	44 (37/7)	0.043 (0.052/0.023)
**1-25%**	67 (57/10)	0.066 (0.080/0.033)	47 (39/8)	0.046 (0.055/0.026)
**<1%**	199 (159/40)	0.196 (0.223/0.132)	0 (0/0)	0 (0/0)

Supplementary Table 10. Repeat content of non-reference human sequences, categorized by repeat class. Abbreviations: *ins*, insertions; *brk*, breakends.

Repeat class	Genome occupancy	Base-pairs *(ins/brk)*	Fraction *(ins/brk)*
**DNA**	0.033	16,808 (10,748/6,060)	0.024 (0.020/0.033)
**LINE**	0.209	108,566 (79,365/29,201)	0.152 (0.150/0.157)
**Low complexity**	0.002	7,088 (3,821/3,267)	0.010 (0.007/0.018)
**LTR**	0.088	46,218 (35,614/10,604)	0.065 (0.067/0.057)
**Other**	0.027	3,176 (2,314/862)	0.005 (0.005/0.005)
**Simple repeat**	0.012	131,635 (90,026/41,609)	0.184 (0.170/0.223)
**SINE**	0.130	102,462 (62,692/39,770)	0.143 (0.119/0.213)
**Total**	0.501	415,953 (284,580/131,373)	0.582 (0.539/0.705)

Supplementary Table 11. Average number of non-reference alleles per genome. In this analysis, we used only those autosomal SNVs and Indels which were located in parts of genomes accessible to the sequencing methods used (Supplementary Information 1.5). For non-reference allele counts in ancestral sequences, only variable sites (non-reference allele frequency < 1) in autosomes were considered. Number of non-reference alleles in a genome was computed as $N_{het} + 2 \times N_{hom}$, where N_{het} is a number of variants with a single alternate allele and N_{hom} is a number of variants with two alternate alleles. Number of individuals in 1000 Genomes populations: AFR - 661, EAS - 504, EUR - 503, AMR - 347. Number of individuals in TOPMed population groups: African - 15,622, Asian - 2,394, European - 29,979, Hispanic/Latino - 4,669, Samoan - 1,198.

TOPMed population group (1000 Genomes population)	Avg. no. non-reference alleles in ancestral sequences (SE)	Avg. no. non-reference alleles at SNVs (SE)		Avg. no. non-reference alleles at Indels (SE)	
		TOPMed	1000 Genomes	TOPMed	1000 Genomes
African (AFR)	365.3 (0.1)	5,200,461 (776)	3,818,410 (2,185)	280,394 (52)	375,884 (218)
Asian (EAS)	334.1 (0.3)	4,662,036 (316)	3,335,322 (391)	244,752 (22)	328,358 (47)
European (EUR)	324.6 (0.1)	4,532,427 (121)	3,242,547 (528)	235,856 (8)	319,294 (56)
Hispanic/Latino (AMR)	329.4 (0.3)	4,648,127 (1,350)	3,318,118 (2,646)	242,937 (90)	326,693 (263)
Samoan	342.2 (0.3)	4,686,777 (501)	--	245,082 (43)	--

Supplementary Table 12. Frequency (%) of unique *CYP2D6* star alleles (haplotypes) detected by the Stargazer program, both known and novel, in 40,250 unrelated TOPMed individuals (80,500 allele calls). Abbreviations: SV, structural variation; AS, activity score; UNK, unknown; DEL, deletion; DUP, duplication; MLP, multiplication; HYB, *CYP2D6/CYP2D7* hybrid; NOV, novel structural variant.

#	Star Allele	SV	AS	European	African	Hispanic/Latino	Asian	Samoan	Amish
1	**1*	Reference	1	33.986	29.003	47.025	27.445	42.100	45.111
2	**1x2*	DUP	2	0.781	0.389	0.727	0.292	0.260	.
3	**1x3*	MLP, NOV	3	0.027	0.094	0.050	0.109	0.104	.
4	**1x4*	MLP, NOV	4	0.014	0.005	.	.	.	.
5	**1x5*	MLP, NOV	5	0.006	.	.	.	.	.
6	**1x6*	MLP, NOV	6	0.002	.	.	.	.	.
7	**2*	.	1	14.811	16.110	16.479	9.526	17.204	9.333
8	**2x2*	DUP	2	0.681	1.384	1.570	0.255	.	0.444
9	**2x3*	MLP, NOV	3	0.063	0.064	0.066	0.036	.	.
10	**2x4*	MLP, NOV	4	0.006	0.005	.	.	.	.
11	**3*	.	0	1.572	0.350	0.860	.	0.052	.
12	**3x2*	DUP	0	0.004	.	.	.	.	.
13	**3x5*	MLP, NOV	0	0.002	.	.	.	.	.
14	**4*	.	0	13.802	3.631	9.537	0.511	0.156	8.444
15	**4x2*	DUP	0	0.147	2.636	0.264	.	.	.
16	**4x3*	MLP, NOV	0	0.004	0.113	.	.	.	.
17	**4x4*	MLP, NOV	0	.	0.010	.	.	.	.
18	**4x5*	MLP, NOV	0	.	.	0.017	.	.	.
19	**4N+*4*	HYB	0	0.761	0.251	0.149	0.146	.	3.556
20	**5*	DEL	0	3.079	5.671	2.959	6.241	1.299	5.111
21	**6*	.	0	1.060	0.256	0.545	.	.	.
22	**6x2*	DUP	0	0.008	.	.	.	.	.
23	**7*	.	0	0.047	0.005	0.033	0.036	.	.
24	**8*	.	0	0.002	.	.	.	.	.
25	**9*	.	0.5	2.541	0.532	1.455	.	0.104	.
26	**9x2*	DUP	1	0.016	.	0.017	.	.	.
27	**10*	.	0.5	1.584	3.833	1.653	12.883	1.507	4.889
28	**10x2*	DUP	1	0.004	0.074	0.017	0.365	.	.
29	**10x3*	MLP, NOV	1.5	0.002	0.005	.	0.109	.	.

30	**10x4*	MLP, NOV	2	.	.	.	0.073	.	.
31	**10x6*	MLP, NOV	3	0.002	.	.	.	.	.
32	**11*	.	0	0.022	.	.	.	.	.
33	**12*	.	0	.	0.084	0.033	.	.	.
34	**13B*	HYB	0	0.047	0.034	0.017	0.036	.	.
35	**13C*	HYB	0	0.261	0.064	0.182	.	0.052	.
36	**14*	.	0.5	.	.	0.017	1.606	.	.
37	**15*	.	0	0.020	0.020	0.033	.	.	.
38	**17*	.	0.5	0.159	15.711	1.719	0.036	0.312	.
39	**17x2*	DUP	1	.	0.123	0.017	.	.	.
40	**18*	.	0	.	.	.	0.073	.	.
41	**19*	.	0	0.008	0.005	0.017	.	.	.
42	**20*	.	0	0.471	0.128	0.231	0.219	0.364	1.778
43	**20x2*	DUP, NOV	0	0.002	.	.	.	.	.
44	**20x3*	MLP, NOV	0	.	0.005	.	.	.	.
45	**21*	.	0	0.012	.	.	0.146	.	.
46	**22*	.	UNK	0.004	.	0.017	.	.	.
47	**28*	.	UNK	0.328	0.084	0.446	.	.	.
48	**28x2*	DUP, NOV	UNK	0.004	.	.	.	.	.
49	**29*	.	0.5	0.071	8.395	1.240	.	.	.
50	**29x2*	DUP	1	.	0.207	0.033	.	.	.
51	**29x3*	MLP, NOV	1.5	.	0.010	.	.	.	.
52	**30*	.	UNK	.	0.044	.	.	.	.
53	**31*	.	0	0.014	.	0.198	.	.	.
54	**33*	.	1	1.085	0.217	0.281	.	.	0.444
55	**33x2*	DUP, NOV	2	0.002	.	.	.	.	.
56	**34*	.	1	0.304	0.512	0.545	0.146	0.624	.
57	**34x2*	DUP, NOV	2	0.020	0.153	0.066	.	.	.
58	**34x3*	MLP, NOV	3	0.002	0.015	.	.	.	.
59	**35*	.	1	5.353	0.857	1.868	0.073	0.156	0.444
60	**35x2*	DUP	2	0.061	0.005	0.165	.	.	.
61	**35x4*	MLP, NOV	4	0.002	.	.	.	.	.
62	**36+*10*	HYB	0.5	0.031	0.187	0.149	34.197	18.139	.
63	**36x7+*10*	HYB, MLP, NOV	0.5	.	.	.	.	0.052	.

64	**39*	.	1	0.073	0.158	0.033	0.036	0.416	.
65	**39x2*	DUP, NOV	2	0.004	.	.	.	.	.
66	**39x3*	MLP, NOV	3	0.004	.	.	.	.	.
67	**39x4*	MLP, NOV	4	.	.	.	0.036	.	.
68	**40*	.	0	0.008	0.631	0.083	.	.	.
69	**40x2*	DUP, NOV	0	.	0.010	.	.	.	.
70	**41*	.	0.5	9.825	2.404	5.719	4.015	5.405	7.333
71	**41x2*	DUP	1	0.024	0.034	0.033	0.073	.	.
72	**41x3*	MLP, NOV	1.5	0.033	.	.	.	.	.
73	**42*	.	0	.	0.236	0.066	.	.	.
74	**43*	.	UNK	0.020	0.887	0.149	0.036	.	.
75	**43x2*	DUP	UNK	.	0.108	.	.	.	.
76	**43x3*	MLP, NOV	UNK	.	0.005	.	.	.	.
77	**45*	.	1	.	0.064	0.017	.	.	.
78	**45x2*	DUP	2	.	0.005	.	.	.	.
79	**46*	.	1	0.008	0.611	0.099	.	.	.
80	**49*	.	0.5	.	.	.	0.036	.	.
81	**50*	.	0.5	.	0.005	.	.	.	.
82	**56*	.	0	0.004	0.168	0.033	.	.	.
83	**59*	.	0.5	0.392	0.064	0.050	.	0.104	1.778
84	**62*	.	0	0.004	.	.	.	.	.
85	**68+*4*	HYB	0	5.508	0.995	2.165	0.073	.	11.333
86	**68x5+*4*	HYB, MLP, NOV	0	0.063	0.005	.	.	.	.
87	**69*	.	0	0.031	0.005	.	.	.	.
88	**71*	.	UNK	0.004	0.005	0.017	0.109	10.291	.
89	**71x2*	DUP, NOV	UNK	.	.	.	.	0.052	.
90	**81*	.	0	.	0.015	.	.	.	.
91	**82*	.	UNK	.	.	0.017	.	.	.
92	**83+*2*	HYB, NOV	1	0.035	0.222	0.083	.	0.052	.
93	**84*	.	0.5	0.002	0.266	0.033	.	.	.
94	**86*	.	UNK	0.002	0.005	.	.	.	.
95	**96*	.	0	.	.	.	0.036	.	.
96	**106*	.	UNK	0.002	0.892	0.066	.	.	.
97	**107*	.	UNK	.	0.025	.	.	.	.

98	**S1+*1*	HYB, NOV	1	0.208	0.113	0.165	0.036	0.156	.
99	**S2+*1*	DEL, NOV	1	0.029	0.044	0.116	.	0.208	.
100	Undetermined	.	.	0.416	0.739	0.364	0.949	0.832	.

Supplementary Table 13. Sample Size (n), Average (Avg) heterozygosity (Het), number of singletons (Sing), within cohort rare variant (RV) sharing, ADMIXTURE values by TOPMed study and population group. Groups: Afr = African, EAsn = East Asian, Eur = European, His/Lat = Hispanic/Latino, Sam = Samoan. Admixture Clusters: Eur1 = first European cluster, Asn = Asian cluster, Amish = Amish cluster, Sam = Samoan cluster, Eur2 = second European cluster, Afr1 = first African cluster, Eur3 = third European cluster, Afr2 = second African cluster, NatAm = Native American cluster. The between-study RV sharing values are given in Supplementary Data 1.

Study	Pop. Group	n	Avg, Het	Avg, Sing	Avg. RV Sharing	Avg. Eur1	Avg. Asn	Avg. Amish	Avg. Sam	Avg. Eur2	Avg. Afr1	Avg. Eur3	Avg. Afr2	Avg. NatAm
Amish	Eur	225	2022666.44	425.22	3.525×10^{-2}	0.0122	0.0002	0.9094	0.0001	0.0562	1.00×10^{-5}	0.0219	1.00×10^{-5}	4.54×10^{-5}
AFGen	Eur	2572	2115260.32	4105.61	4.868×10^{-4}	0.1249	0.0022	0.0254	0.0018	0.7211	0.0023	0.1172	0.0022	0.0029
ARIC	Eur	3150	2109648.87	3950.55	4.999×10^{-4}	0.0767	0.0023	0.0270	0.0017	0.7640	0.0010	0.1223	0.0010	0.0039
CFS	Eur	182	2114570.49	4648.35	5.515×10^{-4}	0.1655	0.0042	0.0244	0.0021	0.6947	0.0003	0.1033	0.0020	0.0036
CHS	Eur	53	2109758.51	3876.19	5.328×10^{-4}	0.1858	0.0020	0.0262	0.0017	0.6698	0.0005	0.1106	0.0016	0.0019
COPDGene	Eur	5653	2115083.01	4359.24	4.860×10^{-4}	0.1280	0.0022	0.0251	0.0016	0.7163	0.0030	0.1173	0.0009	0.0056
FHS	Eur	1262	2114087.25	4929.14	5.293×10^{-4}	0.1789	0.0015	0.0273	0.0017	0.6566	0.0001	0.1308	0.0009	0.0022
GeneSTAR	Eur	340	2113552.12	4634.87	5.018×10^{-4}	0.1406	0.0020	0.0262	0.0019	0.7029	0.0017	0.1216	0.0012	0.0019
GOLDN	Eur	280	2107030.61	4385.82	5.778×10^{-4}	0.0622	0.0051	0.0248	0.0023	0.8088	1.18×10^{-5}	0.0912	0.0005	0.0050
Mayo_VTE	Eur	1161	2108846.87	4626.79	5.318×10^{-4}	0.1042	0.0030	0.0257	0.0018	0.7811	3.11×10^{-5}	0.0816	0.0005	0.0020
MESA	Eur	1621	2117335.35	3972.58	6.717×10^{-4}	0.2722	0.0040	0.0227	0.0021	0.5842	0.0005	0.1078	0.0017	0.0049
WHI	Eur	8101	2112381.22	4240.63	4.927×10^{-4}	0.1112	0.0029	0.0232	0.0011	0.7453	0.0013	0.1115	0.0014	0.0022
GALAII	His/Lat	891	2279532.13	5426.62	1.828×10^{-3}	0.0870	0.0068	0.0140	0.0038	0.0643	0.0787	0.3033	0.0610	0.3810
MESA	His/Lat	828	2293654.33	6827.96	1.232×10^{-3}	0.1196	0.0116	0.0132	0.0056	0.1164	0.1559	0.2458	0.0663	0.2656
SAFS	His/Lat	443	2167178.99	5863.18	2.959×10^{-3}	0.1438	0.0043	0.0150	0.0049	0.1638	0.0139	0.1171	0.0237	0.5134
WHI	His/Lat	273	2225397.04	6685.21	1.207×10^{-3}	0.1577	0.0125	0.0172	0.0062	0.1692	0.0409	0.2447	0.0356	0.3161
ARIC	Afr	186	2815360	4820.82	1.834×10^{-3}	0.0182	0.0035	0.0053	0.0036	0.0644	0.7367	0.0250	0.1373	0.0060
BAGS	Afr	383	2814073.26	6248.38	2.366×10^{-3}	0.0128	0.0020	0.0040	0.0009	0.0443	0.7736	0.0213	0.1362	0.0049
CFS	Afr	157	2809913.25	5113.15	1.820×10^{-3}	0.0142	0.0036	0.0067	0.0030	0.0757	0.7319	0.0398	0.1191	0.0060
COPDGene	Afr	2646	2814045.84	5320.36	1.752×10^{-3}	0.0164	0.0041	0.0050	0.0027	0.0771	0.7132	0.0364	0.1383	0.0068
GeneSTAR	Afr	270	2808915.3	5145.25	1.806×10^{-3}	0.0207	0.0027	0.0070	0.0034	0.0725	0.7223	0.0323	0.1318	0.0074
GENOA	Afr	331	2816393.91	4420.97	1.846×10^{-3}	0.0147	0.0044	0.0046	0.0031	0.0623	0.7449	0.0227	0.1382	0.0051
HyperGEN	Afr	855	2820505.11	5157.84	1.864×10^{-3}	0.0125	0.0044	0.0053	0.0022	0.0514	0.7536	0.0276	0.1372	0.0058
JHS	Afr	1897	2813758.33	4644.06	1.784×10^{-3}	0.0212	0.0031	0.0065	0.0034	0.0781	0.7240	0.0317	0.1258	0.0062
MESA	Afr	1061	2800932.63	5268.29	1.661×10^{-3}	0.0279	0.0035	0.0072	0.0031	0.1090	0.6898	0.0298	0.1228	0.0069
SAGE	Afr	437	2811047.94	5477.55	1.664×10^{-3}	0.0286	0.0069	0.0065	0.0051	0.0710	0.7087	0.0357	0.1255	0.0120
Sarcoidosis	Afr	236	2811252.67	5413.35	1.818×10^{-3}	0.0134	0.0036	0.0039	0.0031	0.0562	0.7608	0.0362	0.1168	0.0059
WHI	Afr	1286	2792439.3	5075.41	1.597×10^{-3}	0.0212	0.0038	0.0081	0.0020	0.1121	0.6744	0.0432	0.1273	0.0080
GenSalt	EAsn	677	1972061.56	13954.51	9.770×10^{-3}	0.0019	0.9865	0.0012	0.0026	0.0022	1.00×10^{-5}	0.0021	1.00×10^{-5}	0.0036
MESA	EAsn	509	1968481.72	13874.66	8.792×10^{-3}	0.0008	0.9538	0.0006	0.0401	0.0037	1.00×10^{-5}	0.0003	1.00×10^{-5}	0.0007
WHI	EAsn	176	1980241.1	14752.07	8.889×10^{-3}	0.0062	0.9074	0.0011	0.0564	0.0202	0.0002	0.0038	0.0003	0.0044
Samoan	Sam	962	1907790.71	2415.64	2.045×10^{-2}	0.0036	0.0222	0.0015	0.9541	0.0147	0.0007	0.0011	0.0005	0.0015

Supplementary Table 14. Resulting fitted parameters from performing demographic inference with 4-fold degenerate sites and sites under the weakest effects of selection at linked sites (SaLS). Weakest SaLS represent sites from the highest 1% *B* bin (99-100% *B*; McVicker's *B* statistic). Parameters include the starting population size before growth ($NEur_0$), the ending population size after growth (*NEur*), and the time span over which exponential growth occurred (*TEur*). The rate of growth is shown in the last column (rEur). Population size is given in units of N_e and time is given in years assuming a generation time of 25 years.

Sample Size (2N)	Data	$NEur_0$ (95% CI)	*NEur* (95% CI)	*TEur* (95% CI)	rEur * 100 (95% CI)
1,000	weakest SaLS	11,689 (11,586-11,792)	1,163,554 (971,534-1,355,573)	5,741 (5,566-5,916)	2.02 (1.94-2.09)
	4-fold degenerate	10,059 (9,902-10,217)	755,334 (644,947-865,722)	8,505 (8,131-8,880)	1.28 (1.23-1.32)
2,000	weakest SaLS	11,762 (11,658-11,866)	1,053,407 (974,047-1,132,768)	5,813 (5,682-5,943)	1.95 (1.92-1.98)
	4-fold degenerate	10,313 (10,159-10,466)	949,761 (867,911-1,031,612)	8,058 (7,802-8,314)	1.41 (1.38-1.44)
3,000	weakest SaLS	11,835 (11,735-11,936)	1,060,540 (1,003,916-1,117,164)	5,793 (5,687-5,899)	1.96 (1.93-1.98)
	4-fold degenerate	10,487 (10,334-10,639)	1,043,842 (976,228-1,111,456)	7,853 (7,634-8,072)	1.48 (1.45-1.50)
4,000	weakest SaLS	11,895 (11,790-12,000)	1,065,695 (1,022,542-1,108,848)	5,778 (5,676-5,879)	1.96 (1.95-1.98)
	4-fold degenerate	10,619 (10,462-10,775)	1,094,072 (1,035,292-1,152,852)	7,738 (7,524-7,952)	1.51 (1.49-1.53)
4,832	weakest SaLS	11,937 (11,716-12,158)	1,066,503 (1,029,592-1,103,414)	5,770 (5,600-5,940)	1.97 (1.95-1.98)
	4-fold degenerate	10,709 (9,469-11,948)	1,118,240 (1,033,993-1,202,487)	7,677 (6,416-8,938)	1.53 (1.50-1.55)

Supplementary Table 15. Distribution of variants in the TOPMed imputation panel in non-reference allele frequency bins.

Variation type	Non-reference allele frequency bins				
	(0, 0.005]	(0.005, 0.01]	(0.01, 0.05]	(0.05, 1)	Totals
**SNVs**	270,352,495	3,365,284	5,330,340	7,020,861	286,068,980
**Insertions**	5,462,262	74,150	130,506	148,595	5,815,513
**Deletions**	15,406,052	185,606	297,186	333,748	16,222,592
**Totals**	291,220,809	3,625,040	5,758,032	7,503,204	308,107,085

Supplementary Table 16. **Rare pLoF disease-associated variants identified in the UK Biobank not present in the HRC panel**. Reported *P*-values are from a single variant association test (two-sided) using the software SAIGE. <5×10^{-8} was used as the threshold to mark statistical significance. AF - non-reference allele frequency. R^2 - Minimac4 imputation quality metric. OR and corresponding 95% confidence intervals are reported from using the Firth test on the unrelated subset of the UK Biobank white British (no third degree relative pairs or closer) because SAIGE's estimates of effect size are unstable for rare variants for traits with a small number of cases.

Trait	Cases		Controls		CHR:BP:REF:ALT	R^2	P-value	OR	95% CI	Status in ClinVar	Gene
	N	AF (%)	N	AF (%)							
Breast cancer	12,564	0.55%	200,481	0.22%	22:28695868:AG:A	0.93	2.7E-22	2.80	2.28-3.43	Pathogenic for familial breast cancer	*CHEK2*
Breast cancer	12,564	0.15%	200,481	0.04%	16:23621362:C:T (present on the UK BiLEVE array with 1,078 untyped)	0.99	2.0E-14	4.60	3.07-6.99	Pathogenic/ likely pathogenic for familial breast cancer	*PALB2*
Hematuria	16,238	0.34%	378,371	0.05%	2:227052367:G:C (present on the UK BiLEVE array with 966 untyped)	0.99	3.5E-48	7.03	5.57-8.87	Pathogenic for Alport's disease (note: key symptom is hematuria)	*COL4A4*
Hereditary hemolytic anemias	151	0.99%	388,413	0.00182%	11:5227001:CT:C (present on the UK BiLEVE array, but "uncounted")	0.85	5.1E-09	706	201-2480	Pathogenic for Beta thalassemia	*HBB*

Supplementary Table 17. Centers that have provided genomic assays to TOPMed.

Center name	Principal Investigator	Assay type(s)
Baylor Human Genome Sequencing Center	Richard Gibbs	WGS
Broad Institute Genomics Platform	Stacey Gabriel	WGS, RNA-seq
McDonnell Genome Institute	Susan K. Dutcher	WGS
Illumina	Karine A. Viaud-Martinez	WGS
Psomagen	Sal Situ	WGS
New York Genome Center	Soren Germer	WGS
Northwest Genomics Center	Deborah Nickerson	WGS, RNA-seq
Beth Israel Deaconess Medical Center	Robert E. Gerszten	Proteomics, Metabolomics
Broad Institute Metabolomics Platform	Clary Clish	Metabolomics
Keck Molecular Genomics Core Facility	David Van Den Berg	Methylomics

Supplementary Table 18. Number of individuals included in each analysis. In total 53,831 individuals were approved for general analyses, and 52,182 individuals were approved for population genetics analyses.

Section	Analysis Type	Sample Size	Comment
Protein loss of function variants	General	53,831	--
The distribution of genetic variation	General	40,722	Unrelated individuals
Insights into mutation processes	Population genetics	3,000	1,000 unrelated individuals of African ancestry, 1,000 unrelated individuals of East Asian ancestry, and 1,000 unrelated individuals of European ancestry which have low levels of genetically estimated admixture.
Beyond SNVs and Indels	General	53,831	
Variation in CYP2D6	General	40,250	Unrelated individuals with reported ancestry
Heterozygosity and rare variant sharing among ancestrally diverse sampled individuals	Population genetics	39,722	Unrelated individuals with reported ancestry
Haplotype sharing	Population genetics	52,182	--
Large sample sizes alleviate impact of selection at linked sites	Population genetics	2,416	Unrelated individuals from TOPMed Freeze 3 whose genomes suggested mostly European ancestry and low admixture
Human adaptations	Population genetics	39,649	Unrelated individuals
TOPMed imputation resource	General	97,256	Samples from TOPMed Freeze 8 with study approvals for use in imputation

Supplementary Table 19. Description of samples in Framingham Heart Study.

Sample	Sequencing Center	Sample Size	Depth
TOPMed WGS	Broad Institute	4,158	>30X
CHARGE WGS	Baylor HGSC	855	6-7X
CHARGE WES	Baylor HGSC	1,702	>30X

Supplementary Table 20. Funding sources for each study and genomic center in TOPMed. Whole genome sequencing support for TOPMed studies was provided by the National Heart, Lung, and Blood Institute (NHLBI) through the Centralized Omics REsource (CORE) program. NYGC = New York Genome Center; BROAD = Broad Institute of MIT and Harvard; UW NWGC = University of Washington Northwest Genomics Center; ILLUMINA = Illumina Genomic Services; PSOMAGEN = Psomagen, Inc.; BAYLOR = Baylor Human Genome Sequencing Center.

Study Accession	TOPMed Parent Study Name	Sequencing Center	Sequencing Support
phs000956	NHLBI TOPMed: Genetics of Cardiometabolic Health in the Amish	BROAD	3R01HL121007-01S1
phs001211	NHLBI TOPMed: Trans-Omics for Precision Medicine Whole Genome Sequencing Project: ARIC	BAYLOR, BROAD	3R01HL092577-06S1, HHSN268201500015C, 3U54HG003273-12S2
phs001143	NHLBI TOPMed: The Genetics and Epidemiology of Asthma in Barbados	ILLUMINA	3R01HL104608-04S1
phs001189	NHLBI TOPMed: Cleveland Clinic Atrial Fibrillation Study	BROAD	3R01HL092577-06S1
phs000954	NHLBI TOPMed: The Cleveland Family Study (WGS)	UW NWGC	3R01HL098433-05S1
phs001368	NHLBI TOPMed: Cardiovascular Health Study	BAYLOR	HHSN268201500015C
phs000951	NHLBI TOPMed: Genetic Epidemiology of COPD (COPDGene) in the TOPMed Program	BROAD, UW NWGC	HHSN268201500014C
phs000988	NHLBI TOPMed: The Genetic Epidemiology of Asthma in Costa Rica	UW NWGC	3R37HL066289-13S1
phs001412	NHLBI TOPMed: Diabetes Heart Study African American Coronary Artery Calcification (AA CAC)	BROAD	HHSN268201500014C
phs000946	NHLBI TOPMed: Boston Early-Onset COPD Study in the TOPMed Program	UW NWGC	3R01HL089856-08S1
phs000974	NHLBI TOPMed: Whole Genome Sequencing and Related Phenotypes in the Framingham Heart Study	BROAD	3R01HL092577-06S1
phs000920	NHLBI TOPMed: Genes-environments and Admixture in Latino Asthmatics (GALA II) Study	NYGC	3R01HL117004-02S3
phs001218	NHLBI TOPMed: GeneSTAR (Genetic Study of Atherosclerosis Risk)	PSOMAGEN, BROAD, ILLUMINA	HHSN268201500014C, R01HL112064
phs001345	NHLBI TOPMed: Genetic Epidemiology Network of Arteriopathy (GENOA)	BROAD, UW NWGC	HHSN268201500014C, 3R01HL055673-18S1
phs001217	NHLBI TOPMed: Genetic Epidemiology Network of Salt Sensitivity (GenSalt)	BAYLOR	HHSN268201500015C

phs001359	NHLBI TOPMed: Genetics of Lipid Lowering Drugs and Diet Network (GOLDN)	UW NWGC	3R01HL104135-04S1
phs000993	NHLBI TOPMed: Heart and Vascular Health Study (HVH)	BROAD, BAYLOR	3R01HL092577-06S1, 3U54HG003273-12S2
phs001293	NHLBI TOPMed: HyperGEN - Genetics of Left Ventricular (LV) Hypertrophy	UW NWGC	3R01HL055673-18S1
phs000964	NHLBI TOPMed: The Jackson Heart Study	UW NWGC	HHSN268201100037C
phs001402	NHLBI TOPMed: Whole Genome Sequencing of Venous Thromboembolism (WGS of VTE)	BAYLOR	HHSN268201500015C, 3U54HG003273-12S2
phs001416	NHLBI TOPMed: MESA and MESA Family AA-CAC	BROAD	3U54HG003067-13S1, HHSN268201500014C
phs001062	NHLBI TOPMed: MGH Atrial Fibrillation Study	BROAD	3R01HL092577-06S1
phs001024	NHLBI TOPMed: Partners HealthCare Biobank	BROAD	3R01HL092577-06S1
phs001215	NHLBI TOPMed: San Antonio Family Heart Study (WGS)	ILLUMINA	3R01HL113323-03S1
phs000921	NHLBI TOPMed: Study of African Americans, Asthma, Genes and Environment (SAGE) Study	NYGC	3R01HL117004-02S3
phs001207	NHLBI TOPMed: African American Sarcoidosis Genetics Resource	BAYLOR	3R01HL113326-04S1
phs000972	NHLBI TOPMed: Genome-wide Association Study of Adiposity in Samoans	UW NWGC, NYGC	HHSN268201100037C, HHSN268201500016C
phs001387	NHLBI TOPMed: Rare Variants for Hypertension in Taiwan Chinese (THRV)	BAYLOR	3R01HL111249-04S1, HHSN26820150015C
phs000997	NHLBI TOPMed: The Vanderbilt AF Ablation Registry	BROAD	3R01HL092577-06S1
phs001032	NHLBI TOPMed: The Vanderbilt Atrial Fibrillation Registry	BROAD	3R01HL092577-06S1
phs001040	NHLBI TOPMed: Novel Risk Factors for the Development of Atrial Fibrillation in Women	BROAD	3R01HL092577-06S1
phs001237	NHLBI TOPMed: Women's Health Initiative (WHI)	BROAD	HHSN268201500014C
phs001435	NHLBI TOPMed: Molecular Mechanisms of Inherited Cardiomyopathies and Arrhythmias in the Australian Familial AF Study	BROAD	3U54HG003067-12S2, 3U54HG003067-13S1
phs001546	NHLBI TOPMed: Determining the association of chromosomal variants with non-PV triggers and ablation-outcome in AF-DECAF	BROAD	3U54HG003067-12S2, 3U54HG003067-13S1
phs001434	NHLBI TOPMed: Defining time-dependent genetic and transcriptomic responses to cardiac injury among patients with arrhythmias	BROAD	3U54HG003067-12S2, 3U54HG003067-13S1
phs001608	NHLBI TOPMed: Outcome Modifying Gene in SCD (OMG-SCD)	BAYLOR	HHSN268201500015C

phs001466	NHLBI TOPMed: Pharmacogenomics of Hydroxyurea in Sickle Cell Disease (PharmHU)	BAYLOR	HHSN268201500015C
phs001468	NHLBI TOPMed: REDS-III Brazil SCD Cohort	BAYLOR	HHSN268201500015C
phs001446	NHLBI TOPMed: Severe Asthma Research Program (SARP)	NYGC	HHSN268201500016C
phs001644	NHLBI TOPMed: BioMe Biobank at Mount Sinai	BAYLOR, WASHU	HHSN268201600033I, HHSN268201600037I, 3UM1HG008853-01S2
phs001515	NHLBI TOPMed: My Life, Our Future: Genotyping for Progress in Hemophilia (MLOF)	NYGC, BAYLOR	HHSN268201500016C, HHSN268201600033I
phs001514	NHLBI TOPMed: Treatment of Pulmonary Hypertension and Sickle Cell Disease With Sildenafil Therapy (Walk-PHaSST)	BAYLOR	HHSN268201500015C

Supplementary Table 21. Analysts and senior scientists who contributed to the particular manuscript section.

Section Title/Contribution	Responsible Analysts
TOPMed program description (Introduction Section and Supplement 1.1)	Stephanie M. Gogarten, Cashell E. Jaquish, Cathy C. Laurie, Sarah Nelson, Quenna Wong
TOPMed WGS data production and batches	Thomas W. Blackwell, Jonathon LeFaive, Matthew P. Conomos, Stephanie M. Gogarten, Cathy C. Laurie, Hyun Min Kang
TOPMed WGS quality assessment	Stephanie M. Gogarten, Achilleas N. Pitsillides, Daniel Taliun, Gonçalo R. Abecasis, L. Adrienne Cupples
410 million genetic variants in 53,831 samples	Daniel Taliun, Gonçalo R. Abecasis
Protein loss of function variants	Daniel Taliun, Gonçalo R. Abecasis, Hyun Min Kang
The distribution of genetic variation	Michael D. Kessler, Timothy D. O'Connor
Insights into mutation process	Jedidiah E. Carlson, Sebastian Zöllner
Beyond SNVs and Indels	Wayne E. Clarke, André Corvelo, Anne-Katrin Emde, Michael C. Zody
Variation in *CYP2D6*	Seung-been Lee, Deborah A. Nickerson
Heterozygosity and rare variant sharing	Daniel Harris, Michael D. Kessler, Douglas Loesch, Amol Shetty, Timothy D. O'Connor
Haplotype sharing	Xiaowen Tian, Sharon R. Browning
Large samples alleviate the effects of linkage	Raul Torres, Ryan D. Hernandez
Human adaptations	Zachary Szpiech, Ryan D. Hernandez
TOPMed imputation resource	Sarah Gagliano Taliun, Hyun Min Kang
TOPMed imputation server	Jacob Pleiness, Lukas Forer, Jonathon LeFaive, Sebastian Schoenherr, Daniel Taliun, Christian Fuchsberger, Albert V. Smith

Supplementary Table 22. Links to summaries and descriptions of phenotype data collected by seven longitudinal cohort studies participating in TOPMed.

Cohort Study	Link to Phenotypic Data Summary
MESA	https://www.mesa-nhlbi.org/aboutMESAStudyTime.aspx
FHS	https://www.framinghamheartstudy.org/wp-content/uploads/2017/08/fhsphenotypicdata.xls
WHI	https://www.whi.org/researchers/data/Pages/Available%20Data.aspx
CARDIA	https://www.cardia.dopm.uab.edu/images/more/recent/CARDIA_Exam_Components---AllYears2018-12-13.pdf
ARIC	https://sites.cscc.unc.edu/aric/cohort-forms/
JHS	https://www.jacksonheartstudy.org/Research/Study-Design/Timeline-Procedures
CHS	https://chs-nhlbi.org/schedule

Supplementary Table 23. Mean (SE) of alternate allele concordance for study sample duplicates for variants passing the SVM quality filter. Calculations were made after removing 6 SNV all, 9 SNV singleton and 4 INDEL outliers with low concordance. Note that the means in this table are for samples pairs sequenced at the same center, whereas those given in the main text include both within- and between-center pairs.

	SNV All		SNV Singletons		INDEL All	
**Sequencing Center**	**N**	**Mean (SE)**	**N**	**Mean (SE)**	**N**	**Mean(SE)**
C1	112	0.99946 (0.00003)	107	0.9966 (0.0002)	115	0.9925 (0.0003)
C2	0	-	-	-	-	-
C3	51	0.99968 (0.00004)	53	0.9971 (0.0003)	50	0.9939 (0.0004)
C4	0	-	-	-	-	-
C5	19	0.99977 (0.00004)	19	0.9990 (0.0002)	19	0.9962 (0.0001)
C6	46	0.99946 (0.00002)	46	0.9979 (0.0002)	46	0.9933 (0.0001)
All	228	0.99954 (0.00002)	230	0.9972 (0.0002)	230	0.9933 (0.0002)

Supplementary Table 24. Means of alternate allele concordance for HapMap genotyping controls CEU NA12878 and YRI NA19238 sequenced within the same center for variants passing the SVM quality filter. The means were calculated over all possible pairs of the sequencing instances. See distributions in Supplementary Figure 8. Only variants that passed the SVM filter were included. N is the number of sequencing instances.

	N	SNV All		INDEL All	
**Sequencing Center**	**CEU/YRI**	**CEU**	**YRI**	**CEU**	**YRI**
C1	5/5	0.99972	0.99946	0.99652	0.99268
C2	5/7	0.99967	0.99961	0.99447	0.99389
C3	15/15	0.99975	0.99963	0.99497	0.99392
C4	0/2	-	0.99962	-	0.99541
C5	6/6	0.99985	0.99980	0.99732	0.99659
C6	1/1	-	-	-	-
All	32/36	0.99975	0.99960	0.99529	0.99410

Supplementary Table 25. Theoretical impact of genotyping batch effects on the identification of spurious association signals. Each row represents a different parameter setting, specifying [a]the sample size, [d]the phenotype prevalence in each of the two batches, and [b]the percentage of tested variants affected by a genotyping batch effect such that [c]the minor allele frequency of these variants in each of the two batches is as specified (genotype frequencies are assumed to follow Hardy-Weinberg equilibrium). Using the theoretical quantiles of the distribution of test statistics (logistic regression score test, two-sided) under these settings, the [e]expected percentage of variants reaching genome-wide significance (i.e. $p < 5\times10^{-8}$) and [f]how many variants that percentage corresponds to per one million variants tested is calculated. As a comparison, the [g]total number of variants affected by the genotyping batch effect is shown as well; in some settings, nearly all affected variants are expected to be detected as significant. The two settings displayed in bold correspond to the two settings highlighted in the supplementary text.

[a]Sample Size	[b]Percent Affected Variants	[c]Minor Allele Frequency (Batch1/Batch2)	[d]Phenotype Prevalence (Batch1/Batch2)	[e]Expected Percent Significant Variants	[f]Expected Number Significant Variants per Million	[g]Number Affected Variants per Million
10000	0%	0.2/0.3	0.3/0.4	5.00E-06%	0.05	0
10000	0.003%	0.2/0.3	0.3/0.4	5.20E-06%	0.05	30
10000	0.003%	0.1/0.3	0.3/0.4	9.90E-05%	0.99	30
10000	0.003%	0.2/0.3	0.2/0.4	8.70E-05%	0.87	30
10000	0.003%	0.1/0.3	0.2/0.4	0.0029%	29.35	30
**25000**	**0.003%**	**0.2/0.3**	**0.3/0.4**	**1.30E-05%**	**0.13**	**30**
25000	0.003%	0.1/0.3	0.3/0.4	0.0018%	17.54	30
25000	0.003%	0.2/0.3	0.2/0.4	0.0017%	16.59	30
25000	0.003%	0.1/0.3	0.2/0.4	0.003%	29.9	30
10000	0.01%	0.2/0.3	0.3/0.4	5.80E-06%	0.06	100
10000	0.01%	0.1/0.3	0.3/0.4	0.00032%	3.17	100
10000	0.01%	0.2/0.3	0.2/0.4	0.00028%	2.79	100
10000	0.01%	0.1/0.3	0.2/0.4	0.0097%	97.05	100
25000	0.01%	0.2/0.3	0.3/0.4	3.40E-05%	0.34	100
**25000**	**0.01%**	**0.1/0.3**	**0.3/0.4**	**0.0059%**	**59.06**	**100**
25000	0.01%	0.2/0.3	0.2/0.4	0.0055%	54.87	100
25000	0.01%	0.1/0.3	0.2/0.4	0.0099%	98.86	100

Supplementary Table 26. Number of variants in each sequencing set in Framingham Heart Study.

Sequencing Set	Total	SNVs	Indels	Multi-allelic
TOPMed WGS Depth>0 (4,158)	58,740,718	51,390,7555	4,519,101	2,830,862
TOPMed WGS Depth>0 (430)	23,836,812	21,120,031	1,697,530	1,019,251
CHARGE WGS (855)	25,832,397	25,832,397	N/A	N/A
CHARGE WGS (430)	20,546,566	20,546,566	N/A	N/A
CHARGE WES (1,701)	475,758	441,263	11,533	22,962
CHARGE WES (430)	225,455	210,025	4,629	10,801

Supplementary Table 27. Average bi-allelic SNV Rates.

Study	All SNVs	Exonic SNVs
TOPMed	0.138 (2,383,558.6)	0.095 (15,829.1)
CHARGE WGS	0.151 (2,658,391.9)	0.115 (17,374.4)

Supplementary Table 28. Counts and percentages of variants by minor allele frequency.

MAF	TOPMed (%)	CHARGE WGS (%)	CHARGE WES (%)
0.001-0.005	8,900.787 (47)	6,724,501 (38)	105,781 (60)
0.005-0.01	1,527,058 (8)	1,605,014 (9)	15,502 (9)
0.01-0.05	2,482,218 (13)	2,920,460 (17)	21,457 (12)
0.05-0.1	1,178,543 (6)	1,469,328 (8)	8,414 (5)
0.1-0.2	1,542,854 (8)	1,695,692 (10)	9,503 (5)
0.2-0.3	1,139,687 (6)	1,187,933 (7)	6,277 (3)
0.3-0.4	1,017,644 (5)	1,048,952 (6)	5,301 (3)
0.4-0.5	944,966 (5)	970,532 (5)	4,709 (3)

Supplementary Table 29. Population clusters in the selection at linked sites demography analysis. Population clusters for Freeze 3 individuals identified by k-means clustering, with total number of individuals, total number of unrelated individuals, and total number of unrelated and consented individuals per population, along with population label assignments. Unrelated and consented individuals from Population 1 ('European A') were selected for demographic analysis.

Population	Label Assignment	N	N Unrelated	N Unrelated and Consented
1	European A	6,474	4,488	3,288
2	Puerto Rican	487	477	475
3	Amish	1,111	231	0
4	Costa Rican	1,062	567	0
5	European B	301	215	159
6	African Admixed	6,330	4,186	3,323
7	Samoan	384	363	0
8	Mexican	504	490	489
9	European C	1,581	742	643
	**TOTAL**	18,234	11,759	8,377

Supplementary Table 30. Total number of sites with MAF > 0.05 analyzed per population in the SDS analysis.

Population	Sites Analyzed
European	4,385,704
African	6,425,469
East Asian	4,260,373

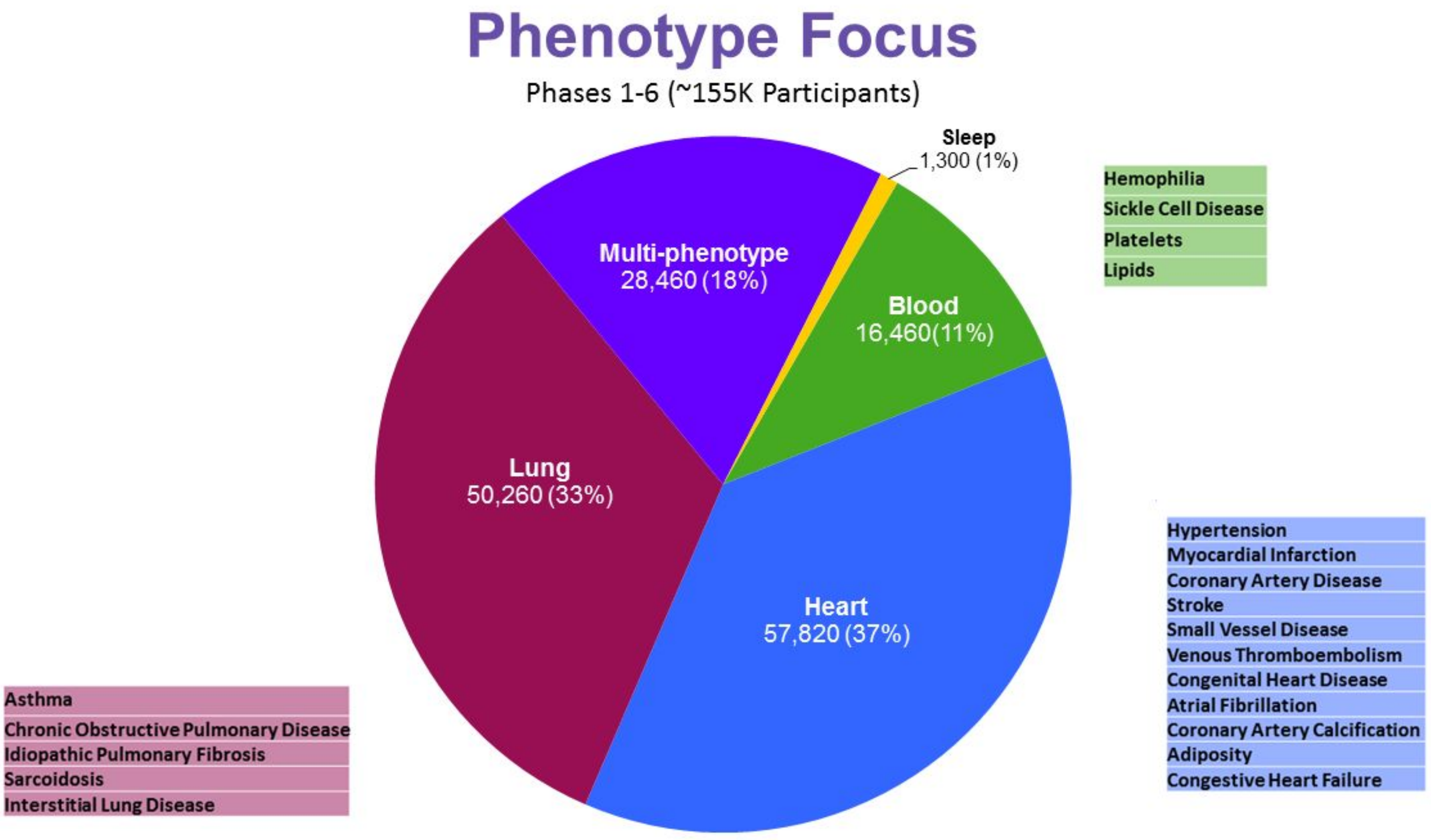

Supplementary Figure 1. Areas of phenotype focus among TOPMed Parent studies. Approximate sample sizes and percentages are given for ~155,000 participants in the first six Phases of the program who have been or are being whole-genome sequenced. "Multi-phenotype" refers to cohort studies with a wide range of phenotypes. In addition, lung studies also have many heart-related phenotypes and vice versa. The participant numbers given do not refer specifically to case counts, but rather to all participants in the study.

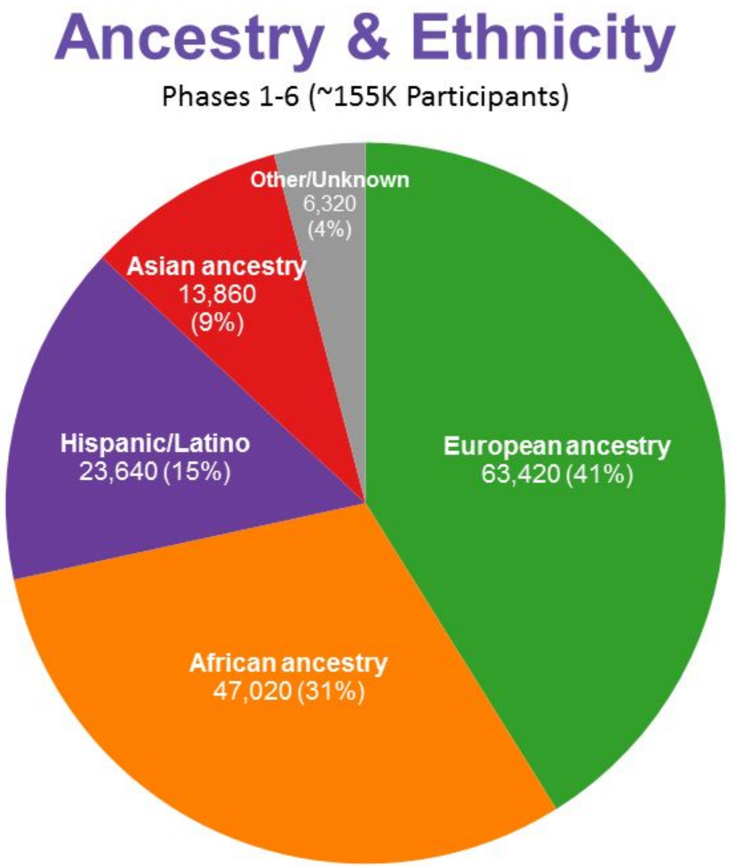

Supplementary Figure 2. Major ancestral and ethnic groups among TOPMed participants. Approximate sample sizes and percentages are given for ~155,000 participants in the first six Phases of the program who have been or are being whole-genome sequenced. These groups consist of European ancestry (European, European American); African ancestry (African, African American, African Caribbean); Hispanic/Latino (including Mexican, Mexican American, Central American, South American, Cuban, Dominican, Puerto Rican); Asian ancestry (Chinese, Taiwanese, Asian American, Pakistani); and 'Other' (Samoan, Native American, multiple groups, or unknown).

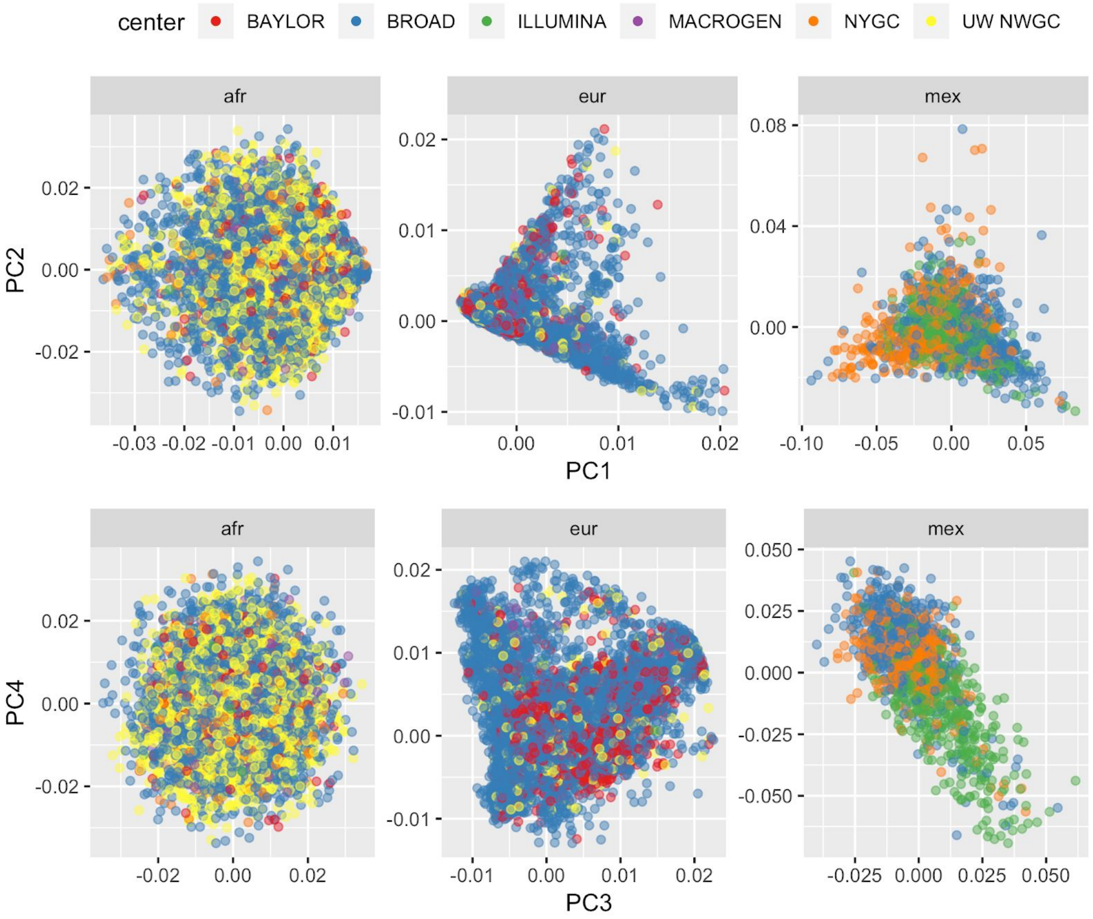

Supplementary Figure 3. Principal component analysis within population groups. Each point represents a single subject. Color-coding is according to the sequencing center at which the subject's genome was sequenced. PCA was done for each population group separately - African Americans (afr), European Americans (eur) and Mexican Americans (mex).

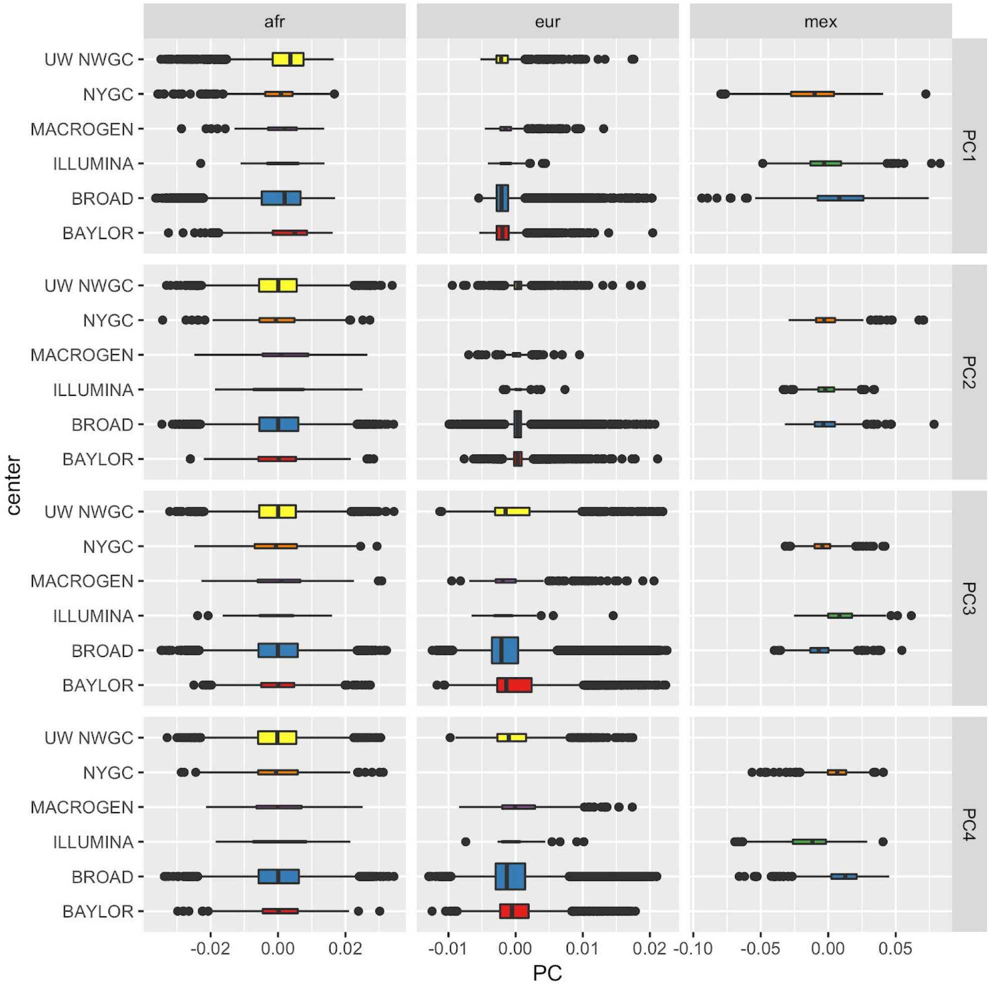

Supplementary Figure 4. Principal component analysis within population groups: boxplots. These boxplots represent the distributions of PC values displayed in Supplementary Figure 3. Centre line denotes the median; box limits denote upper and lower quartiles; whiskers denote 1.5× the interquartile range; points denote outliers. In total 33,376 individuals were examined: 9,337 from African Americans (afr), 22,668 from European Americans (eur), 1,371 from Mexican Americans (mex).

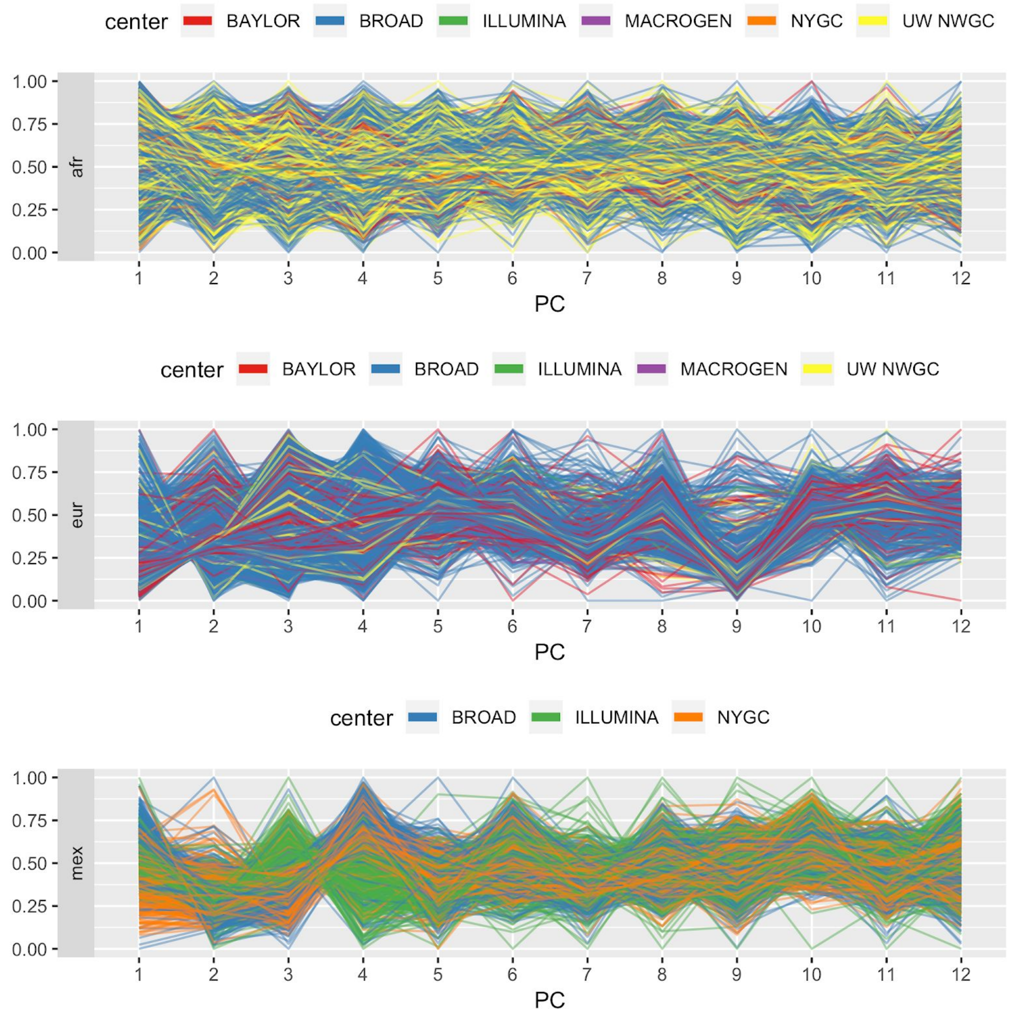

Supplementary Figure 5. Principal component analysis within population groups: parallel coordinate plots. Each line represents a single subject. Color-coding is according to the sequencing center at which the subject's genome was sequenced. PCA was done for each population group separately - African Americans (afr), European Americans (eur) and Mexican Americans (mex).

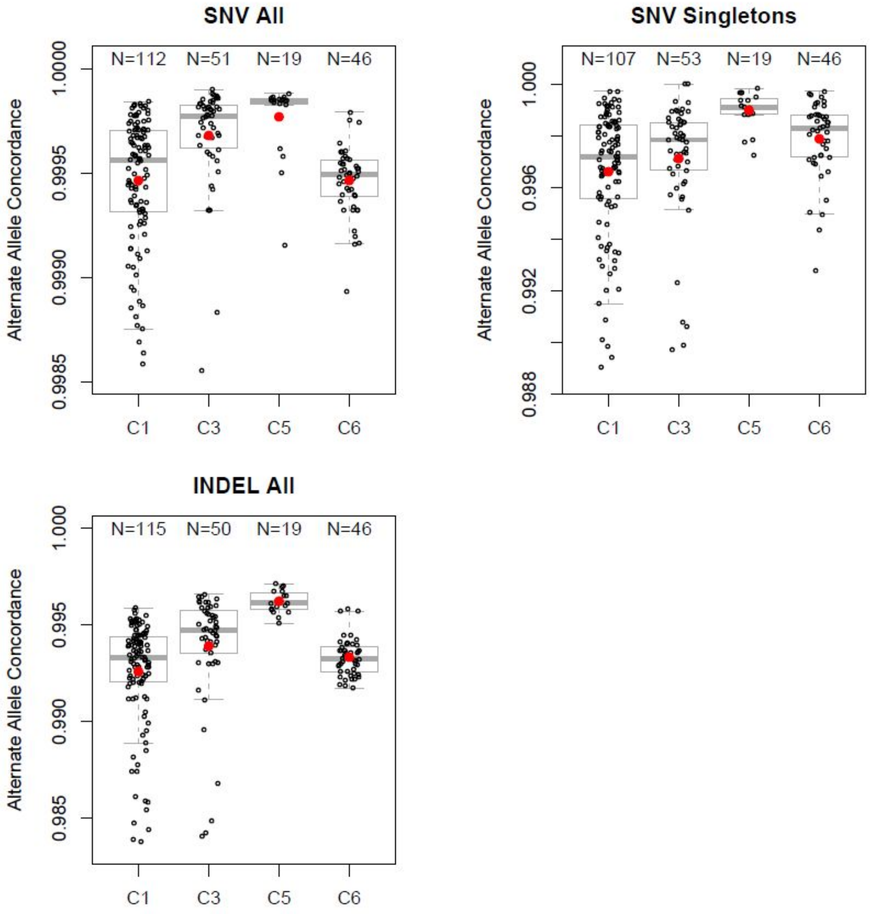

Supplementary Figure 6. Alternate allele concordance for pairs of sequencing instances for the same subject within each of four different centers (C1, C3, C5 and C6), using only variants that pass the SVM quality filter. Each subject is represented by only one pair (and one point in each panel). The box delimits the interquartile range, its center line is the median, and the red dot is the mean. The lower/upper whiskers mark the minimum/maximum of the observations or the first/third quartile minus/plus 1.5 times the interquartile range. All individual observations are overlaid on the box plot, except for a small number of low outliers excluded from the distribution. N is the number of sequencing instance pairs represented by each boxplot. The numbers are slightly different due to excluding outliers (6 for SNV all, 9 for SNV singletons and 4 for INDEL all). Note the differences in scale among the three plots. Centers not shown (C2,C4) had no within-center sequencing duplicates.

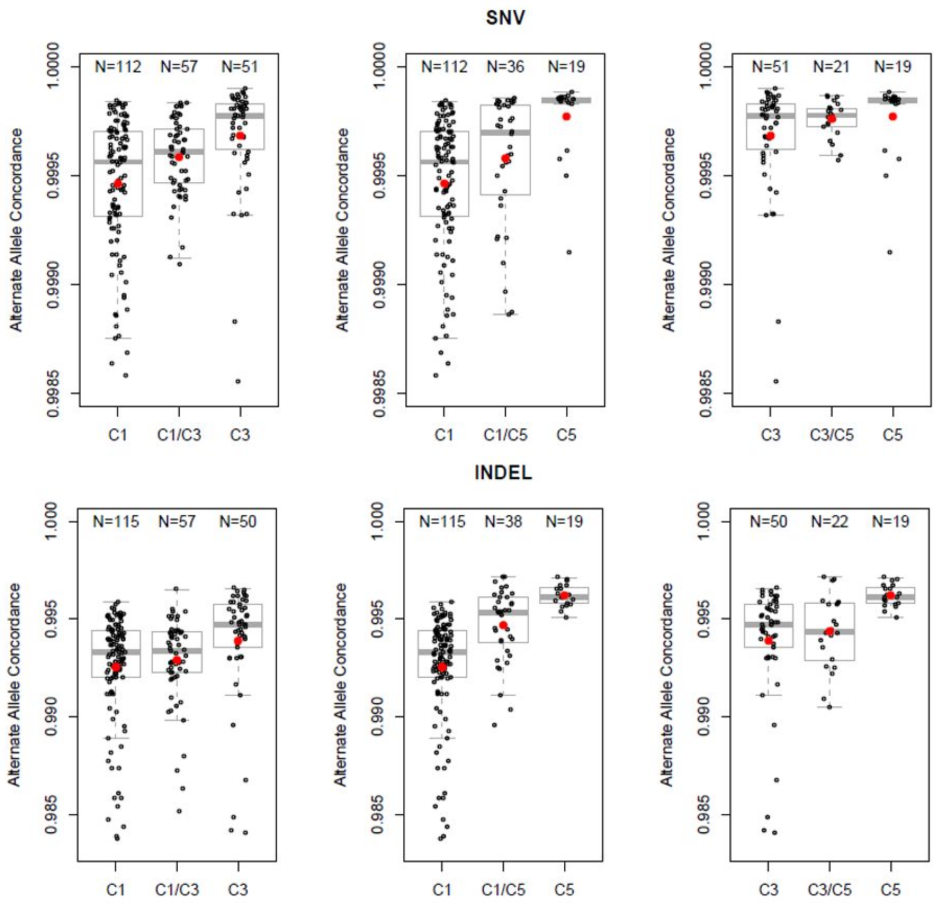

Supplementary Figure 7. Alternate allele concordance for within- and between-center comparisons involving three centers (C1, C3 and C5), using only variants that pass the SVM quality filter. Within each triplet of boxplots, the outer two are within-center and the middle is between center concordance. Each point represents a pair of sequencing instances for the same subject and each subject is represented only once for either SNV or INDEL concordance. The box delimits the interquartile range, its center line is the median, and the red dot is the mean. The lower/upper whiskers mark the minimum/maximum of the observations or the first/third quartile minus/plus 1.5 times the interquartile range. All individual observations are overlaid on the box plot, except for a small number of low outliers excluded from the distribution. N is the number of pairs represented by each boxplot. The numbers are slightly different for SNVs and INDELs due to excluding outliers (9 for SNV and 4 for INDEL). Note the difference in scale between the SNV and INDEL rows. Centers not shown had < 3 pairs of between-center sequencing instances.

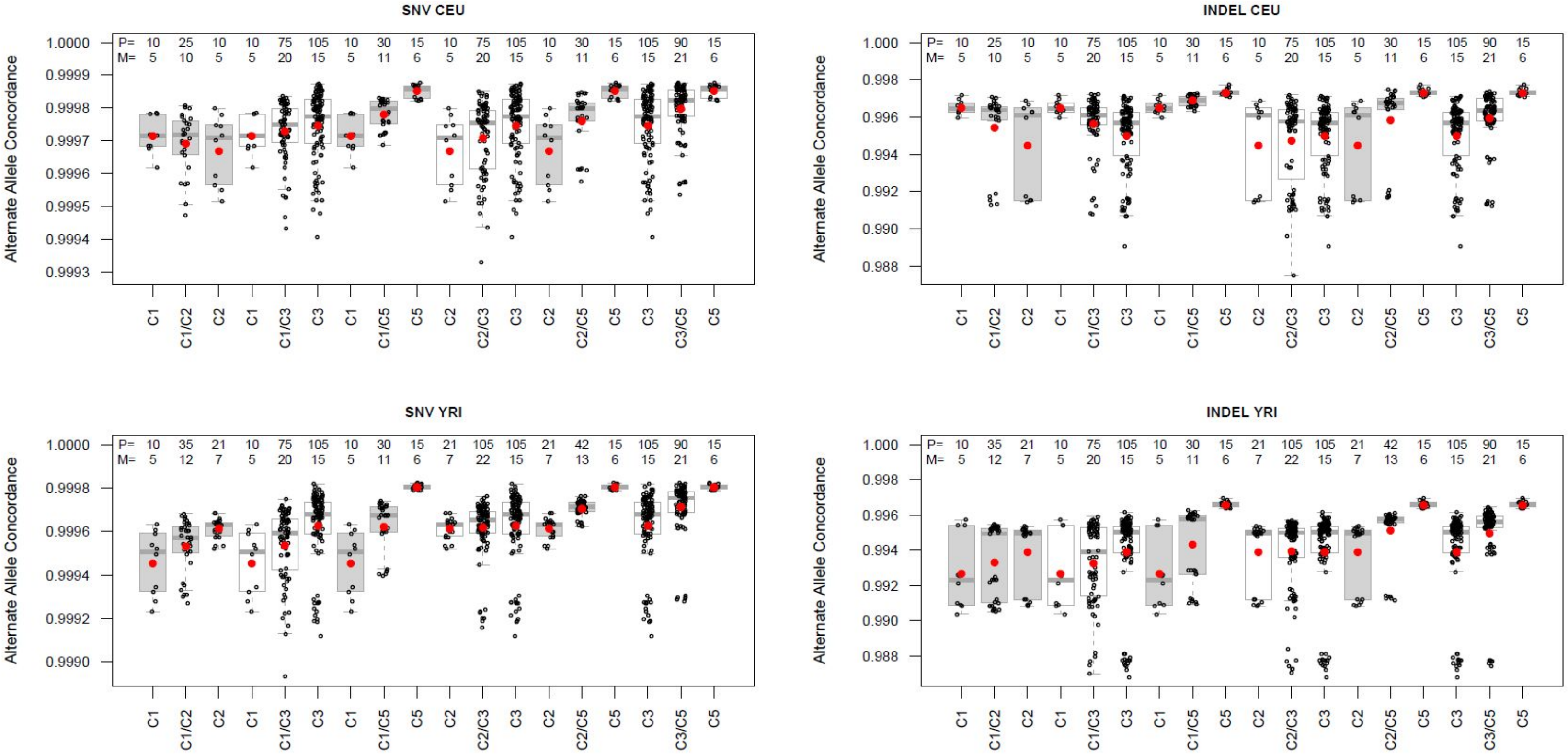

Supplementary Figure 8. Alternate allele concordance distributions for each of two HapMap controls (CEU NA12878 and YRI NA19238), using only variants that pass the SVM quality filter. The plots show within- and between-center comparisons involving four centers (C1, C2, C3 and C5), each having > 2 within-center sequencing instances. Within each triplet of boxplots, the outer two are within-center and the middle is the corresponding between-center concordance. The numbers at the top of each panel represent the number of sequencing instances contributing to the boxplot below it (M), and the number of pairwise comparisons among those M sequencing instances (P). The P individual data points are overlaid on the boxplot. Note that these data points are not independent of one another. The box delimits the interquartile range, its center line is the median, and the red dot is the mean. The lower/upper whiskers mark the minimum/maximum of the observations or the first/third quartile minus/plus 1.5 times the interquartile range.

(A) Manhattan plot of African American single variant analysis (truncated)

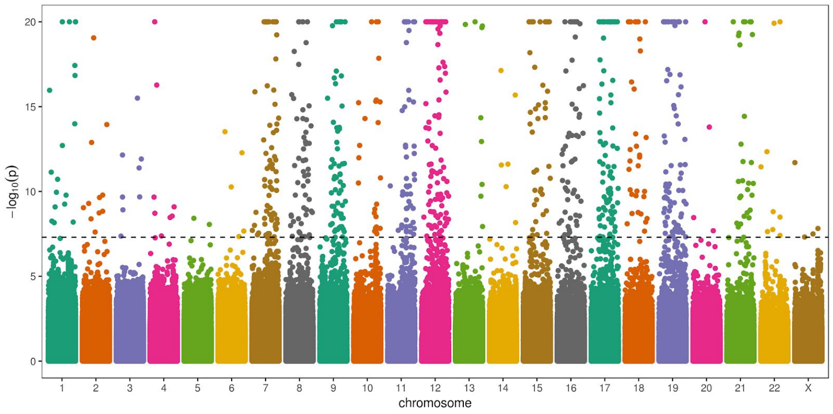

(B) Manhattan plot of European American single variant analysis (truncated)

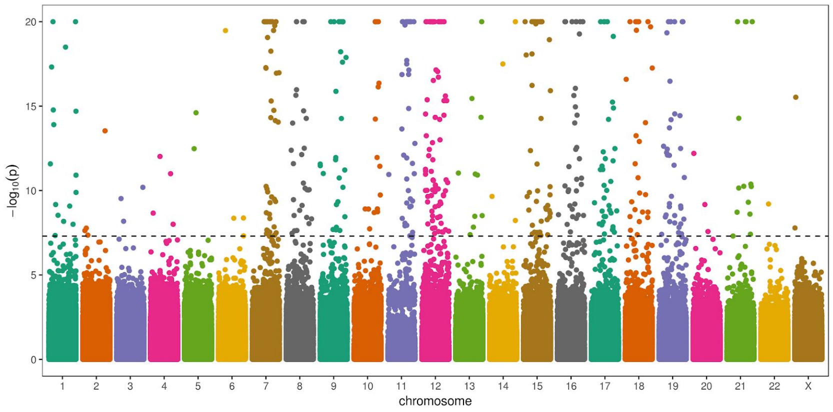

Supplementary Figure 9. Truncated Manhattan plots of association tests for batch effects. Manhattan plots of the -$\log_{10}$(p)-values from logistic regression score tests (two-sided) of association for the (A) African American and (B) European American analyses where the sequencing center (equivalently TOPMed phase) was used as the binary outcome variable. As the most significant variants have -$\log_{10}$(p) > 100, all values of -$\log_{10}$(p) > 20 are truncated and plotted at 20 for readability. The dashed line indicates the genome-wide significance level of $p < 5 \times 10^{-8}$ accounting for multiple testing. All variants with minor allele count (MAC) $\geq$ 20 were tested for association.

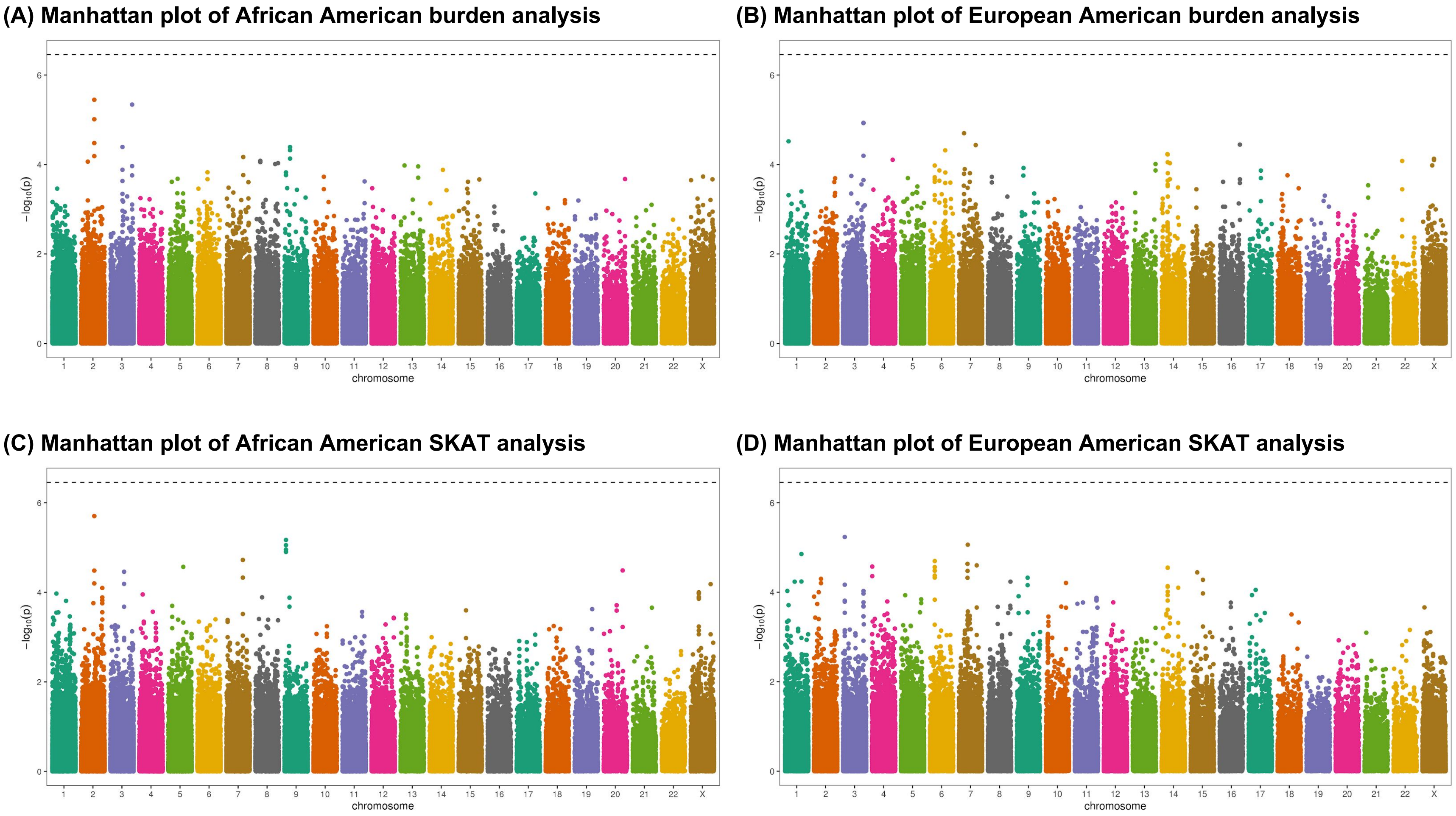

Supplementary Figure 10. Manhattan plots of rare variant association tests for batch effects. Manhattan plots of the -$\log_{10}$(p)-values from logistic regression score tests (two-sided) for association for the (A) African American and (B) European American rare variant burden analyses and the (C) African American and (D) European American rare variant SKAT analyses, where the sequencing center (equivalently TOPMed phase) was used as the binary outcome variable. All variants with minor allele frequency (MAF) $\leq$ 1% in the sample were tested for association using 50kb sliding windows shifted by 20kb increments genome-wide. The dashed line indicates the Bonferroni adjusted significance level adjusted for the number of windows tested; no aggregation units reached significance.

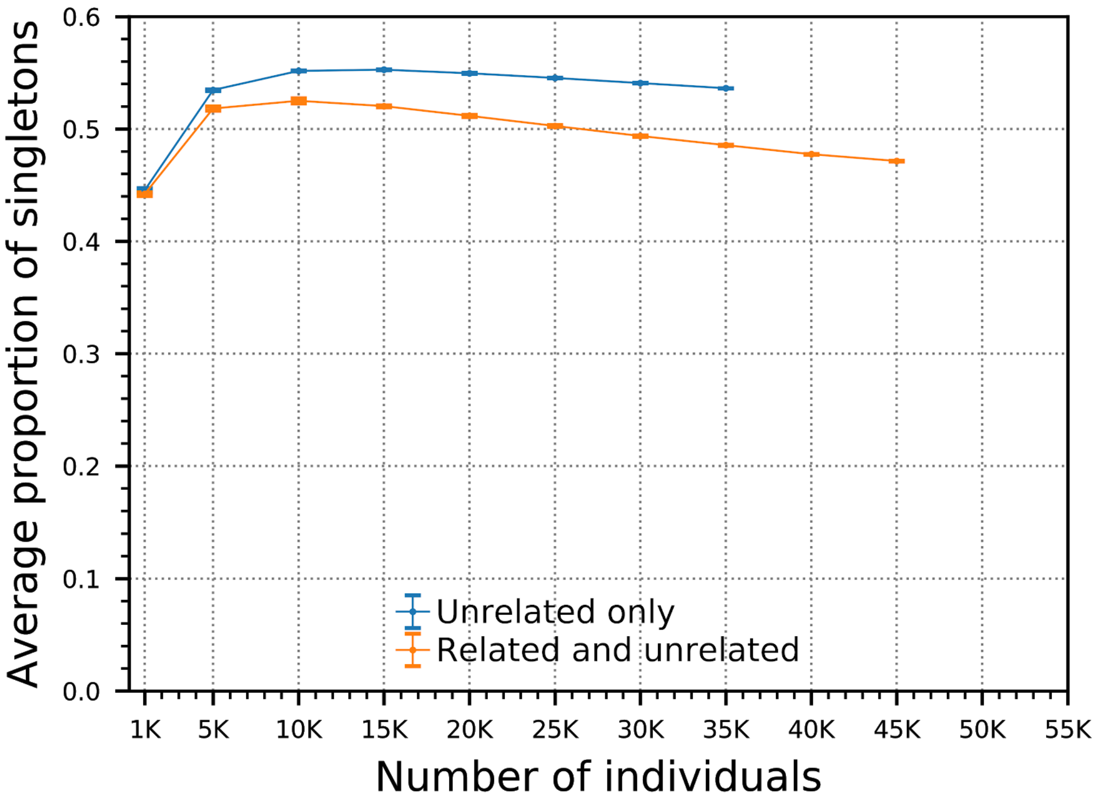

Supplementary Figure 11. Average proportion of singletons by sample size. X-axis represents the number of individuals in a random sample drawn from the original data. For each different number of individuals, N = 20 samples were drawn with replacement and proportion of singletons was computed for each sample. Centre points represent average values. Error bars represent standard errors (SE).

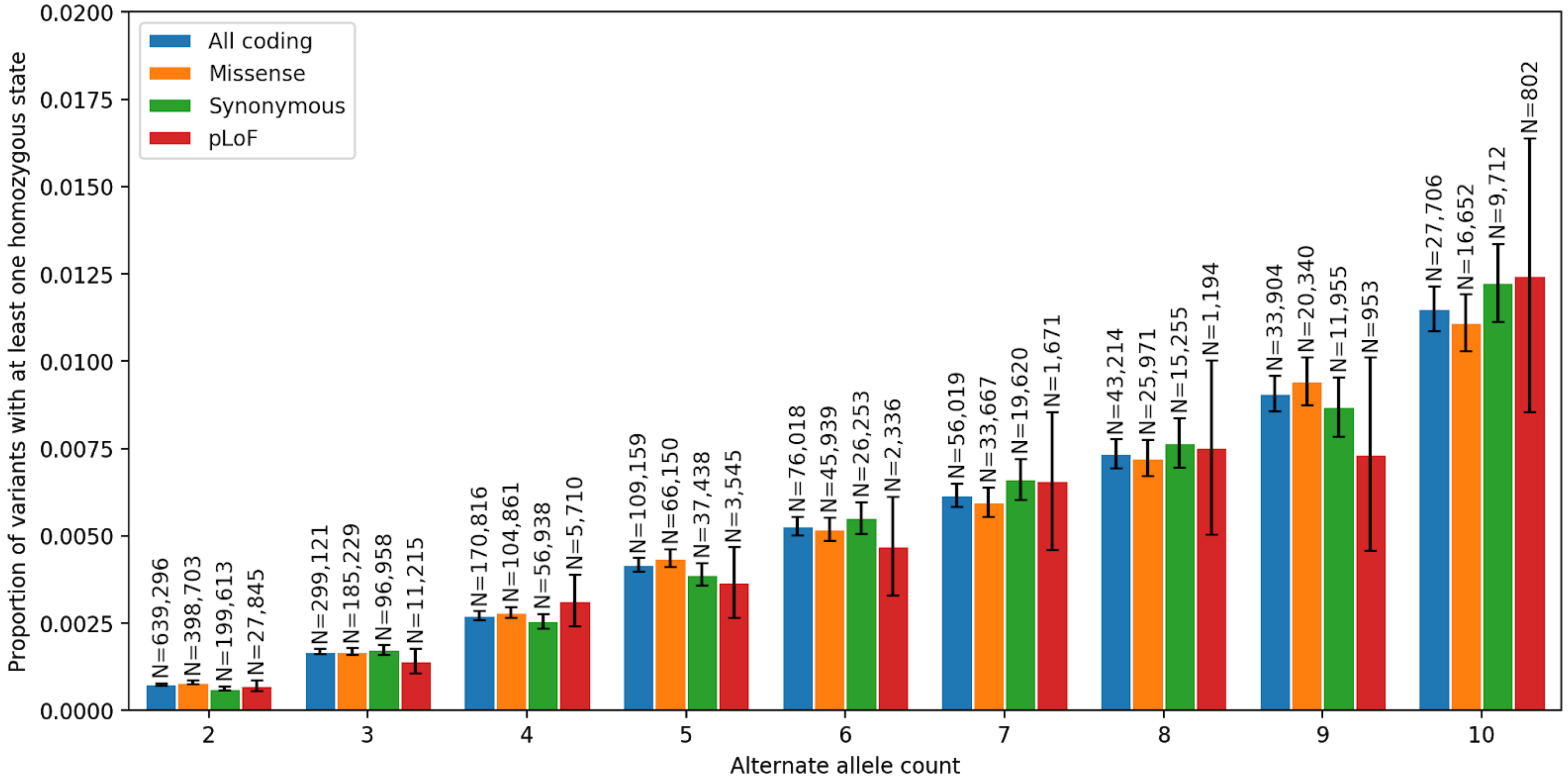

Supplementary Figure 12. Proportion of coding variants with at least one homozygous state in unrelated individuals. Centre of error bars represent the sample proportion (i.e. proportion of variants with at least one homozygous state in a group), error bars indicate standard error (SE) of sample proportion, N indicates total number of variants in each group. There was no evidence that pLoF variants are less likely to be in homozygous state compared to other coding variants.

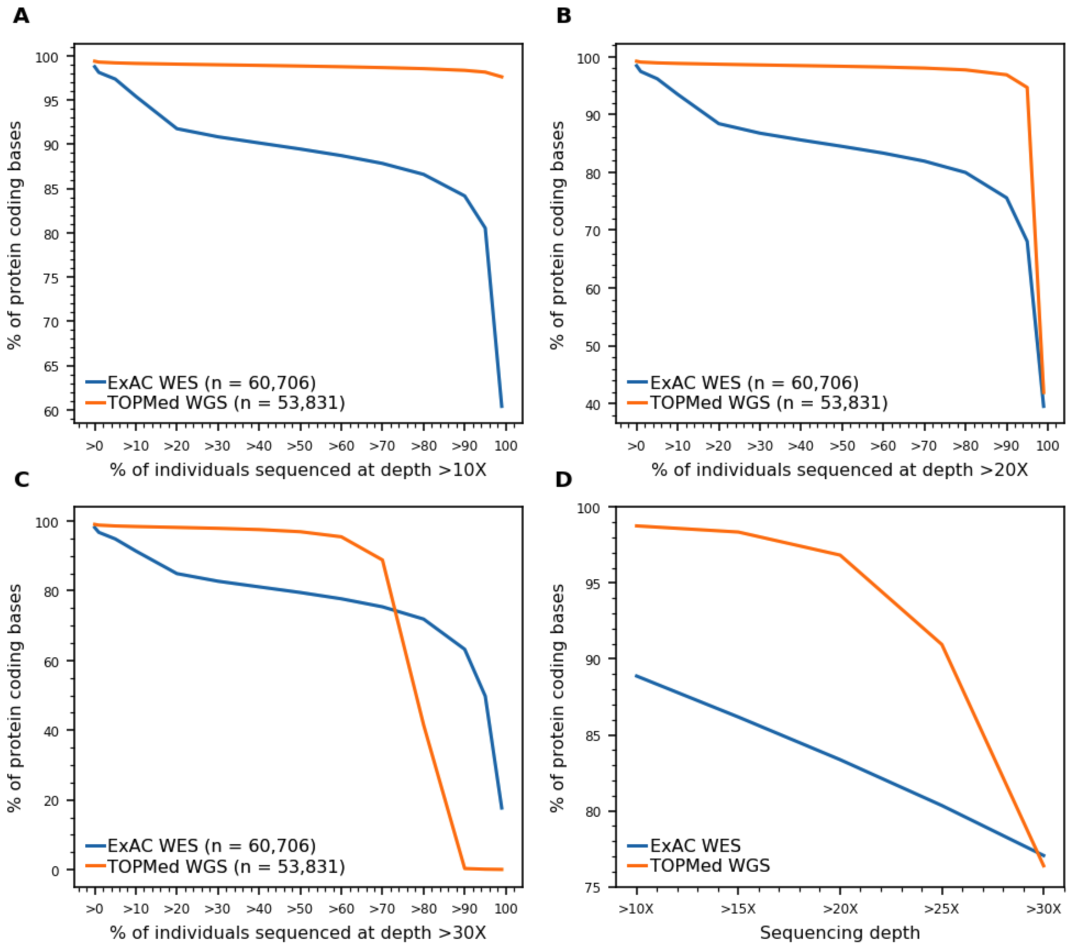

Supplementary Figure 13. Sequencing depth at protein coding bases in TOPMed and ExAC. We computed percent of protein coding bases in CCDS genes sequenced at different depths in TOPMed and ExAC datasets. **A**. Percent of protein coding bases which were sequenced at depth >10X. **B**. Percent of protein coding bases which where sequenced at depth >20X. **C**. Percent of protein coding bases which where sequenced at depth >30X. **D**. Percent of protein coding bases across all individuals sequenced at different depths.

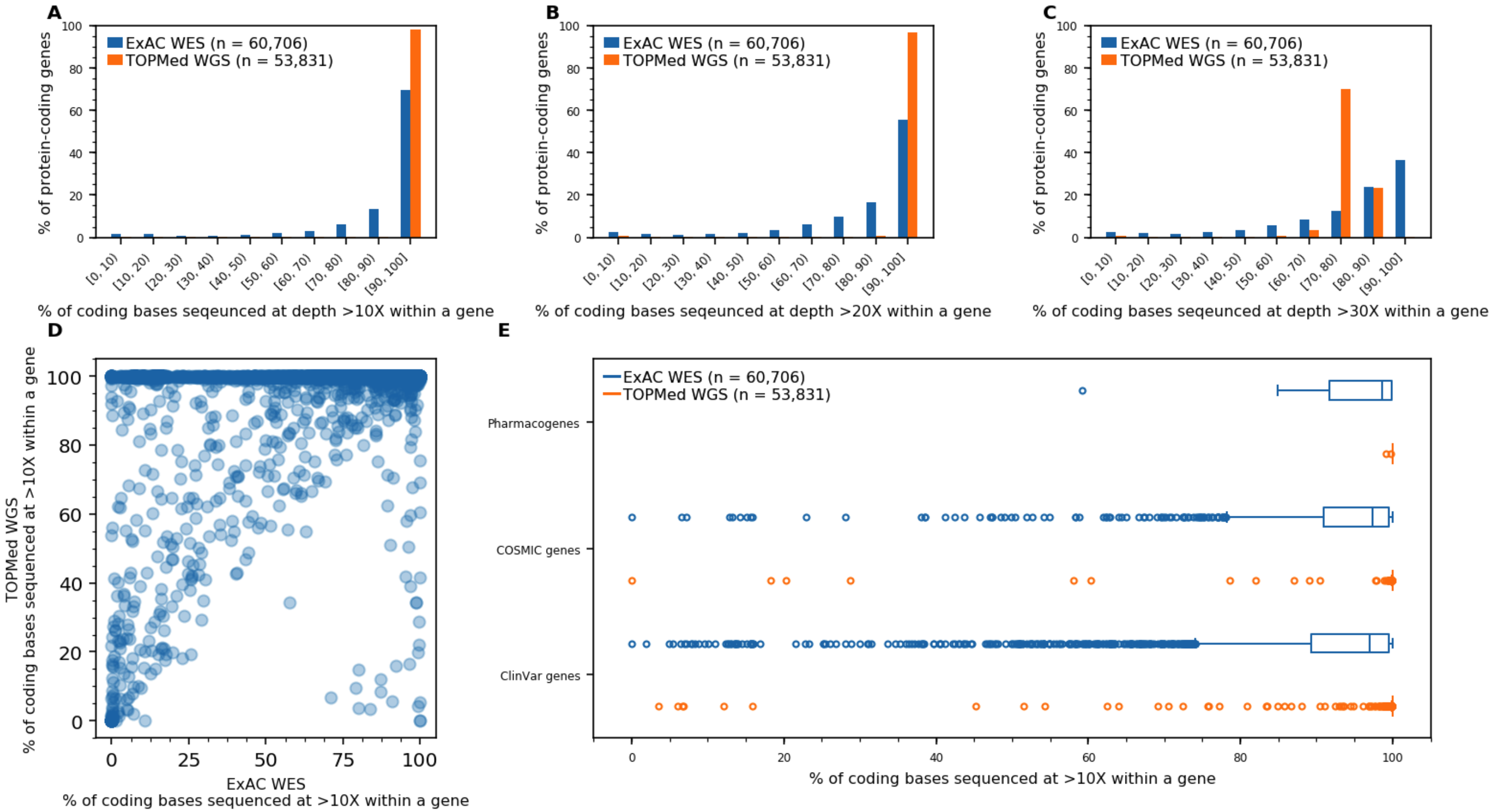

Supplementary Figure 14. Sequencing depth at protein coding genes in TOPMed and ExAC. We compared sequencing depth at protein coding genes, present in Consensus Coding Sequence (CCDS) Project, in ExAC and TOPMed. **A-C**. Percent of protein-coding CCDS genes in ExAC and TOPMed with different percent of coding bases sequenced at >10X, >20X, and >30X, respectively. **D**. For each protein-coding CCDS gene (point) shows the percent of coding bases sequenced at >10X in ExAC (x axis) and TOPMed (y axis). **E.** Percent of coding bases sequenced at >10X in ExAC and TOPMed in genes from ClinVar database (at least one pathogenic variant reported, N = 3,340), genes from COSMIC database (N = 866), and pharmacogenes (N = 15). Centre line denotes the median; box limits denote upper and lower quartiles; whiskers denote 1.5× the interquartile range; points denote outliers.

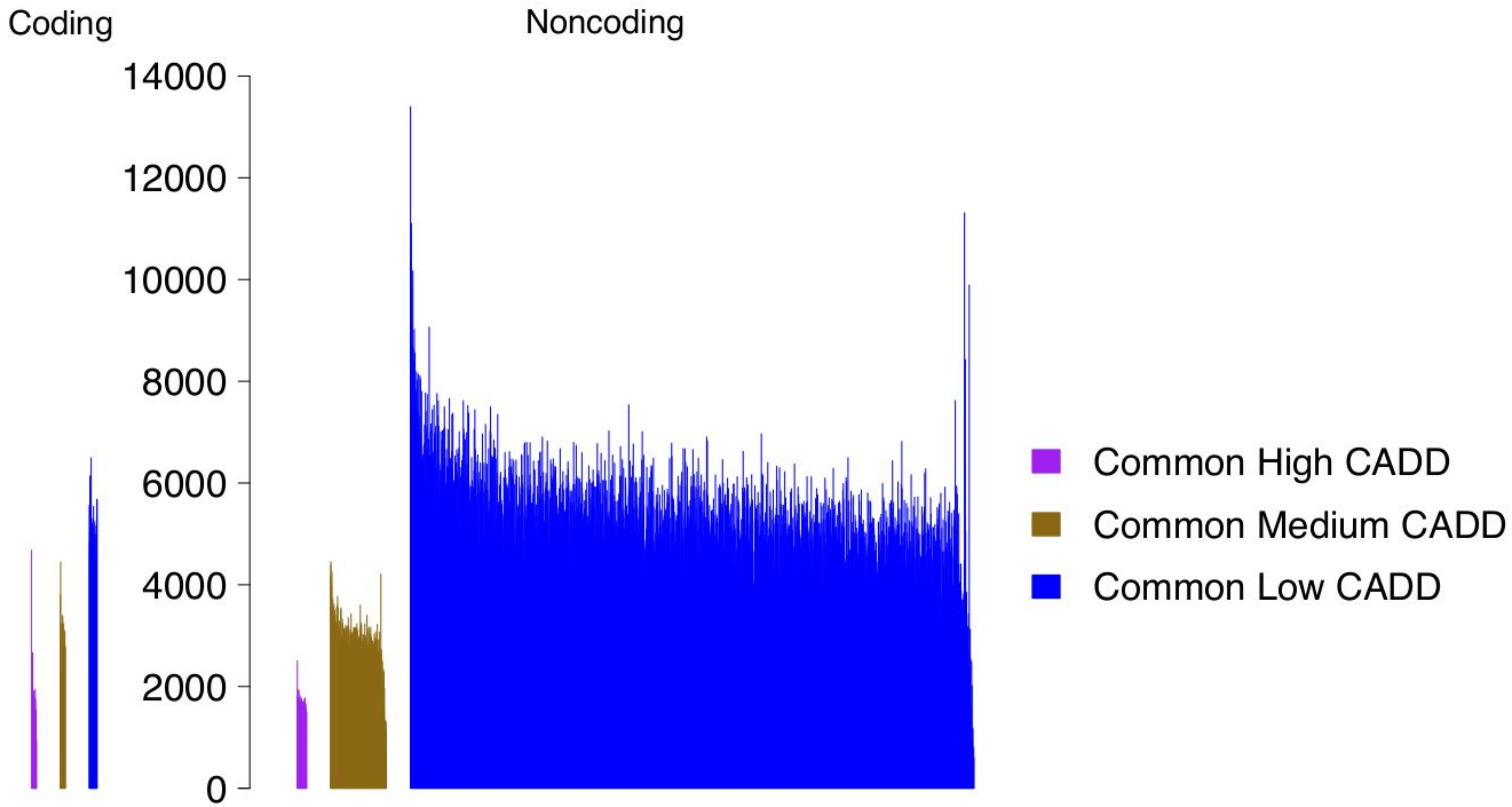

Supplementary Figure 15. Distribution of common genetic variants across concatenated genomic segments. The distributions of common genetic variants are shown across concatenated segments built from bases with high, medium, and low CADD scores. These are the same distributions that are featured in Figure 1, but are enlarged to enhance visualization.

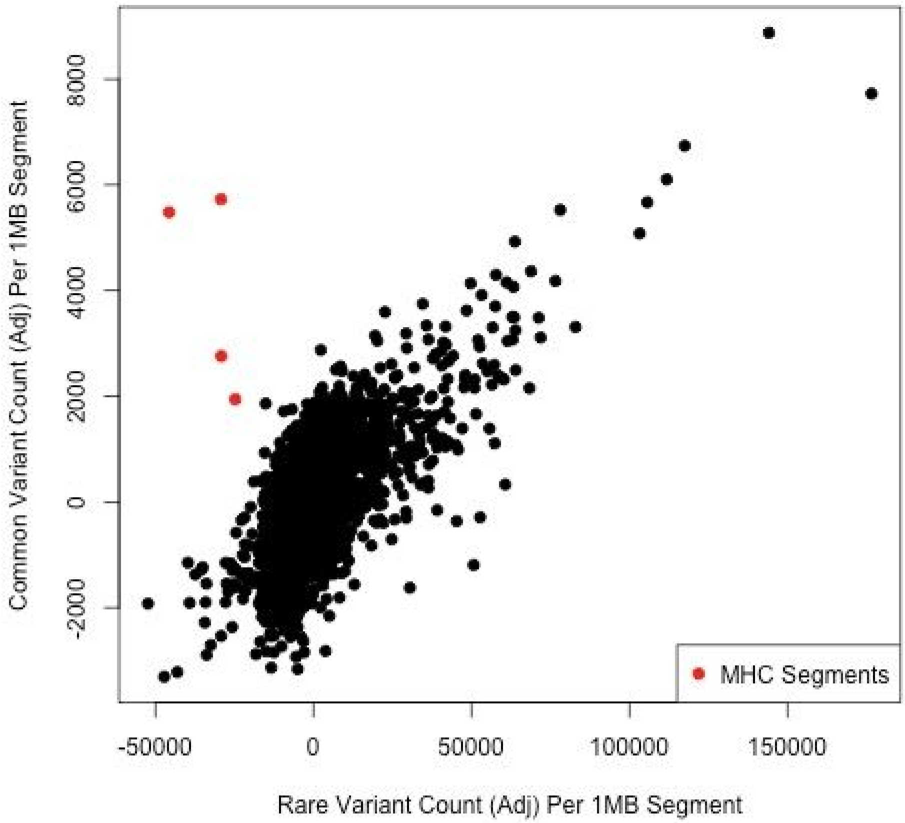

Supplementary Figure 16. Scatter plot of common and rare variant counts per contiguous segment. After using regression to adjust variant counts by the proportion of bases per 1Mb segment that were flagged for mappability concerns, the number of common (MAF ≥ 0.5%) and rare variants (MAF < 0.5%) in contiguous segments are highly and significantly correlated (linear regression, R^2 = 0.462, p-value ≤ $2{\times}10^{-16}$). Outlier segments with higher than expected levels of common variation overlap MHC regions of the genome (red), which is consistent with the effects of balancing selection known to shape these loci.

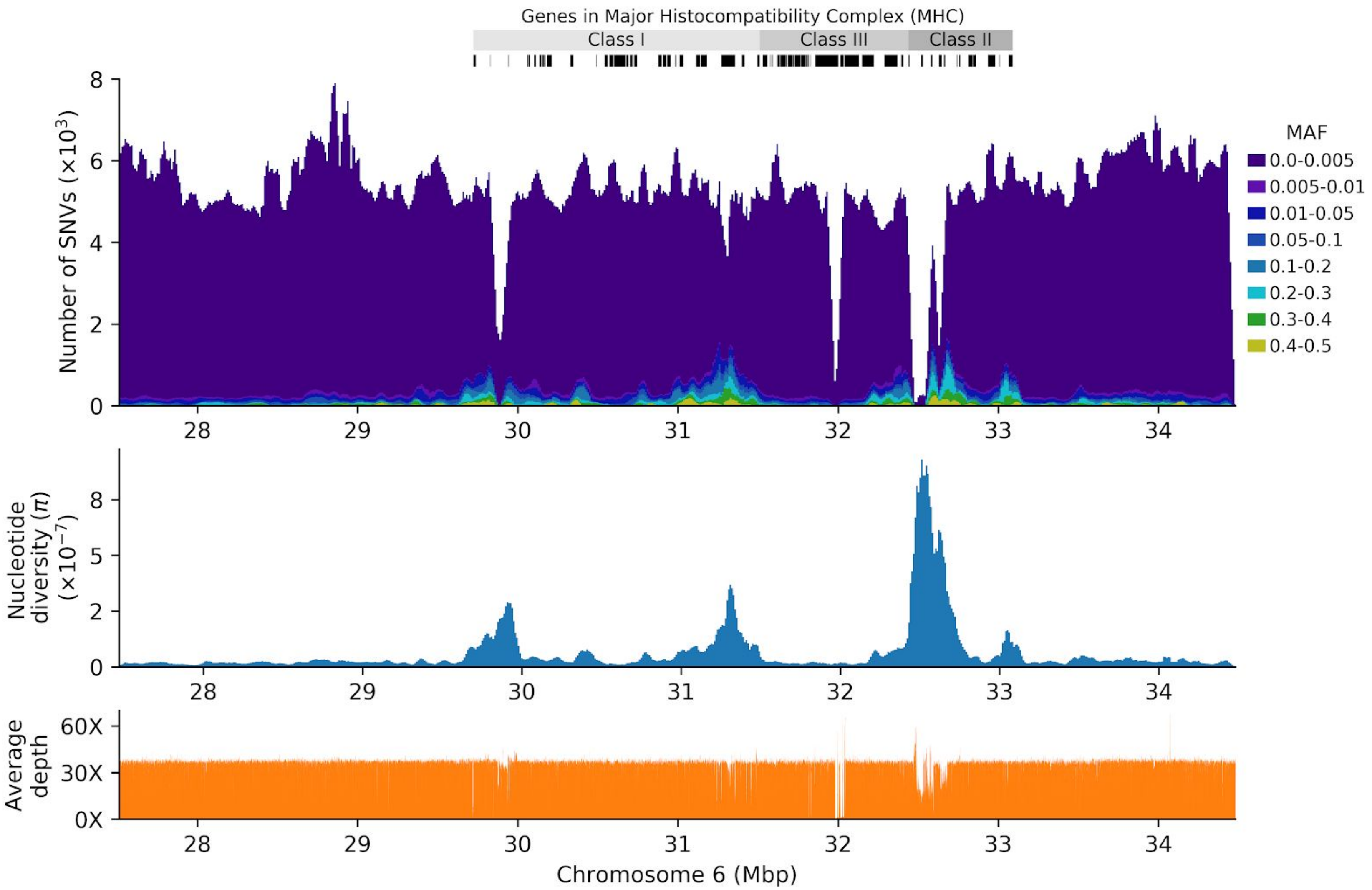

Supplementary Figure 17. Number of SNVs, nucleotide diversity, and average depth in the MHC region in TOPMed. Top panel shows the number of SNVs in 50 Kbp sliding windows with the 10 Kbp step size. Middle panel shows nucleotide diversity (**π**) in 50 Kbp sliding windows with the 10 Kbp step size. Bottom panel shows average sequencing depth at each base pair across randomly selected 1,000 individuals.

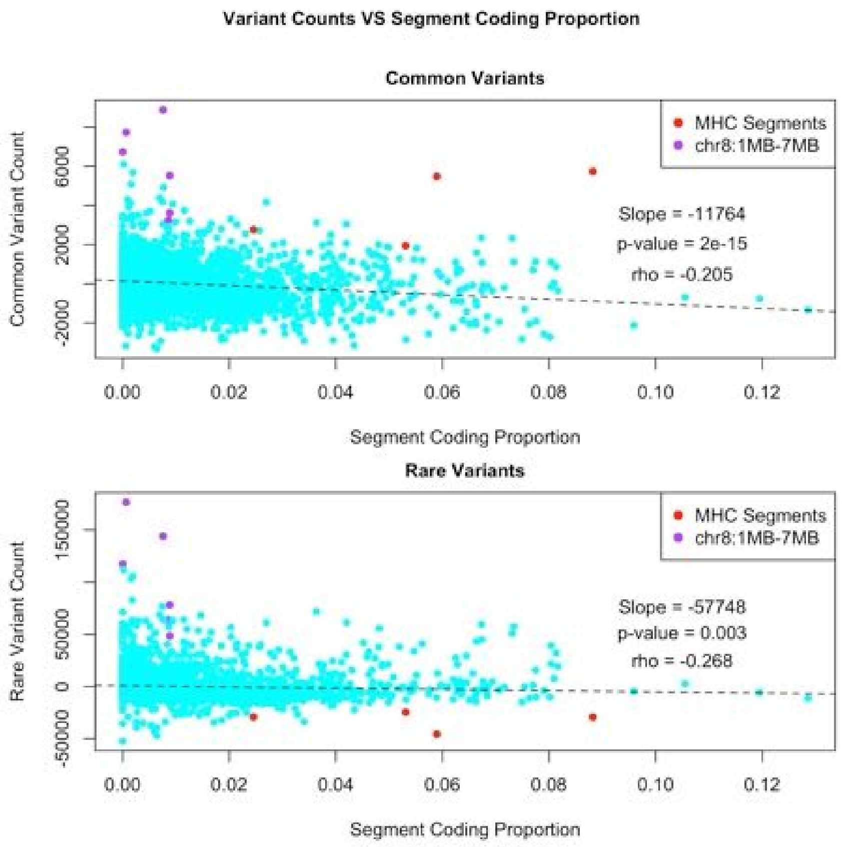

Supplementary Figure 18. Scatter plot of the relationship between variant count and contiguous segment coding proportion. There is a significant negative correlation (linear regression) between segment coding proportion and variant count, which holds when subsetting variants according to allele frequency, i.e. common (Pearson's r=-0.205, p-value $\leq 2\times10^{-15}$) and rare (Pearson's r=-0.268, p-value = 0.003) variants. Outliers, representing regions of potential interest can be seen, including megabases 1 to 7 on chromosome 8 (purple) and segments overlapping Major Histocompatibility Complex (MHC) genes (red). Counts are adjusted for segment coding proportion and mappability (i.e. accessibility mask, see Supplementary Section 1.5).

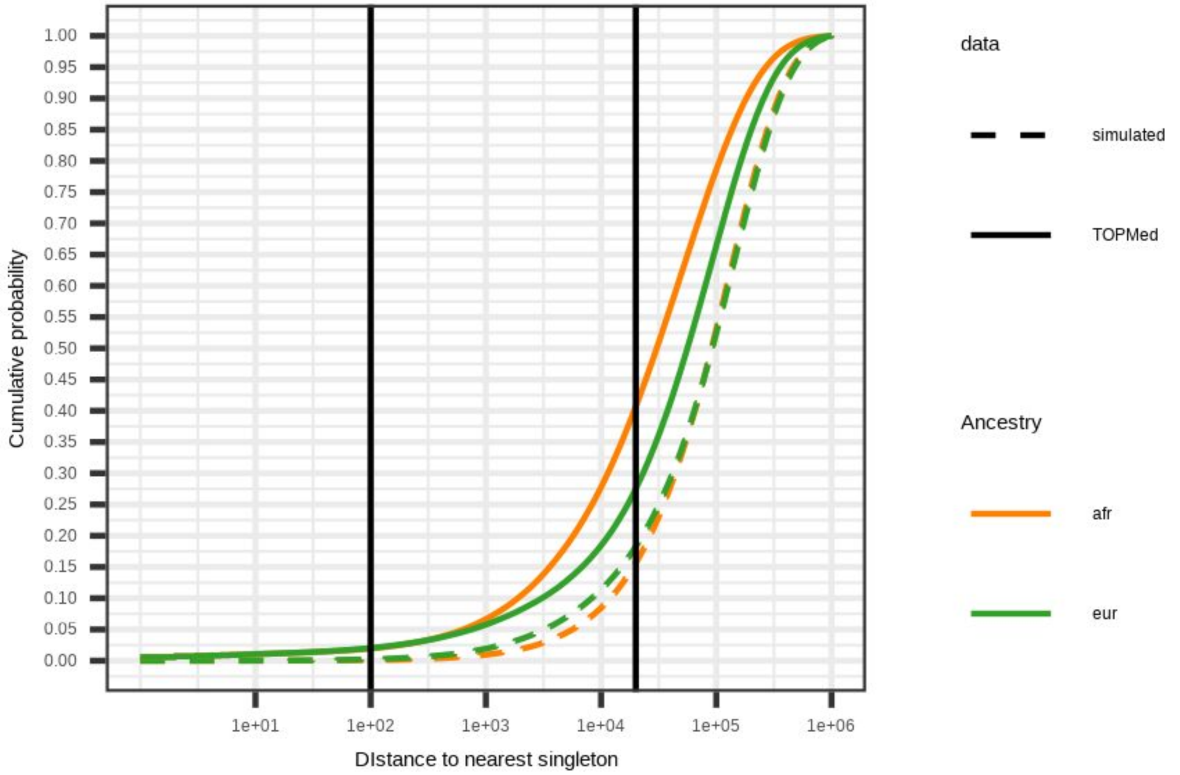

Supplementary Figure 19. Cumulative distribution functions for the inter-singleton distance distributions. CDFs for the observed TOPMed data are shown as solid lines and data simulated under a coalescent model are shown as dashed lines. We observe 1.9% of singletons in a given individual occur at distances of <100bp apart (first vertical line from left), and 34.9% occur at distances <20,000bp apart (second vertical line from left). We performed coalescent simulations based on a realistic model of human demographic history (see Methods) and found that only 0.16% of the simulated singletons were <100bp apart and 16.9% of simulated singletons were <20,000bp apart within the same individual. Note that we do not include the EAS TOPMed samples in this figure because the demographic model used in our simulations only considered African and European populations.

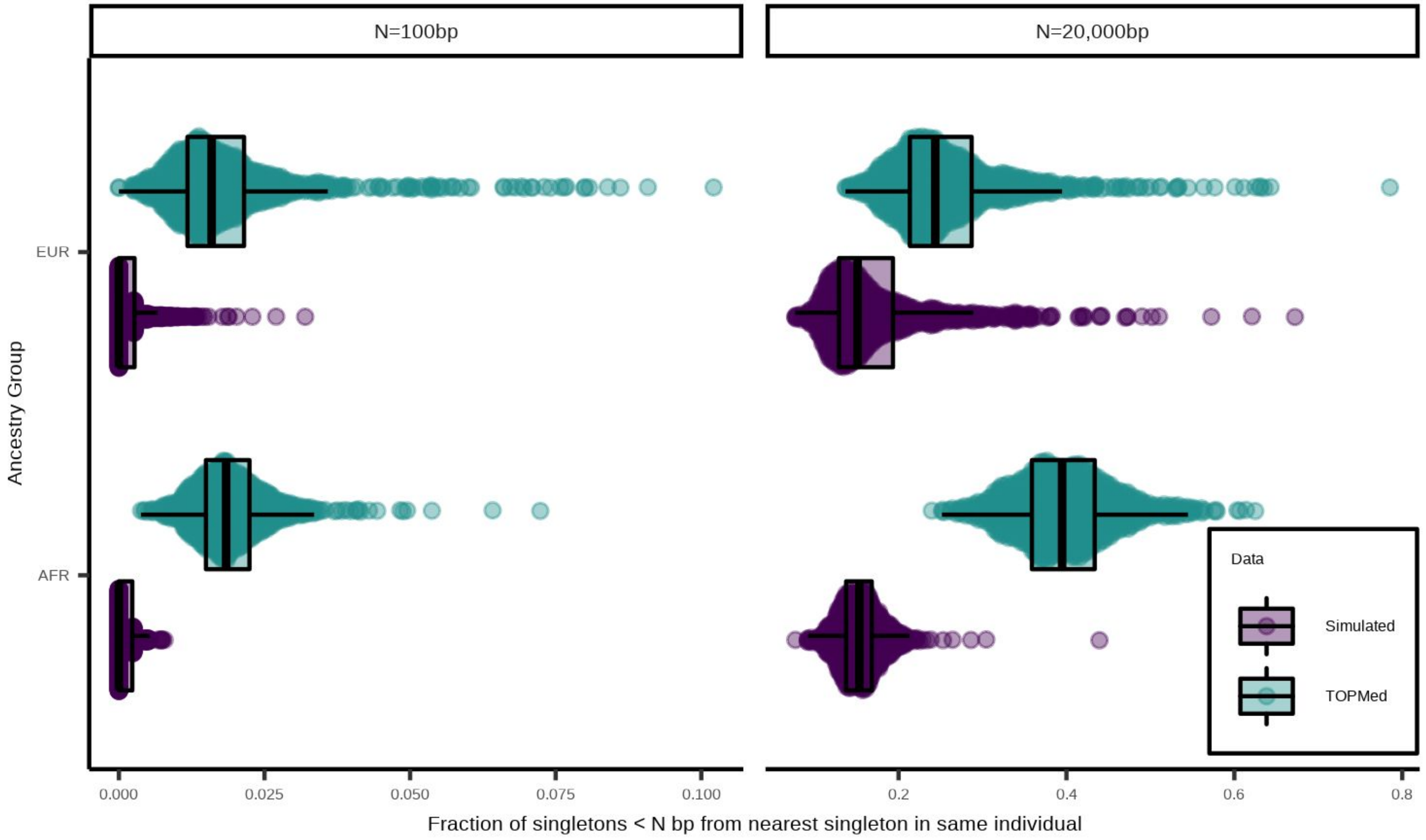

Supplementary Figure 20. Comparison of singleton clustering in TOPMed data and simulated data. In each panel, each point represents the fraction of singletons in an individual that are less than 100bp away (left panel) and 20,000bp away (right panel) from another singleton in the same individual, either in the observed (green) TOPMed data (European N = 1,000, African N = 1,000) or in data simulated (purple) under a coalescent model (European N = 1,000, African N = 1,000). Centre line denotes the median; box limits denote upper and lower quartiles; whiskers denote 1.5× the interquartile range.

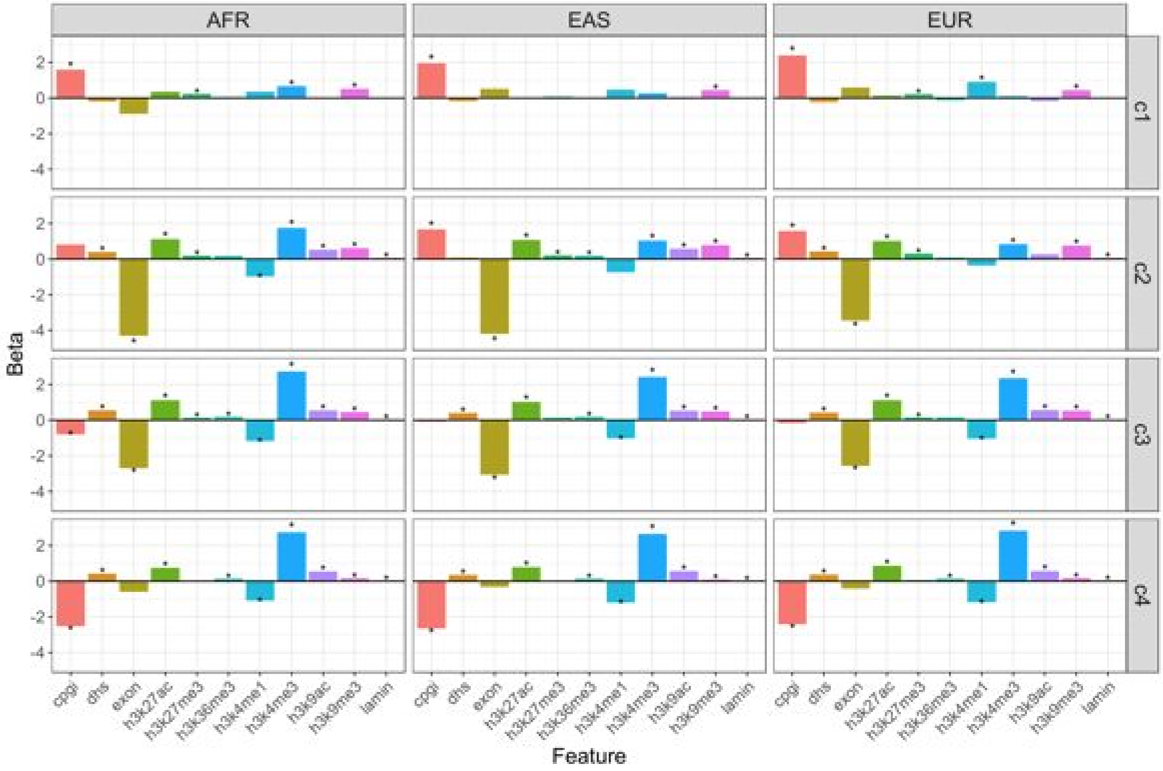

Supplementary Figure 21. Cluster densities by various features of genomic landscape. For each cluster we applied a negative binomial regression model with genomic features as predictors and the frequency of singletons in N=2,897 1Mbp windows as the response variable. The y-axis indicates the magnitude of the beta coefficients from the regression models. Asterisks denote statistically significant associations (two-sided Z test, p-value < 0.05).

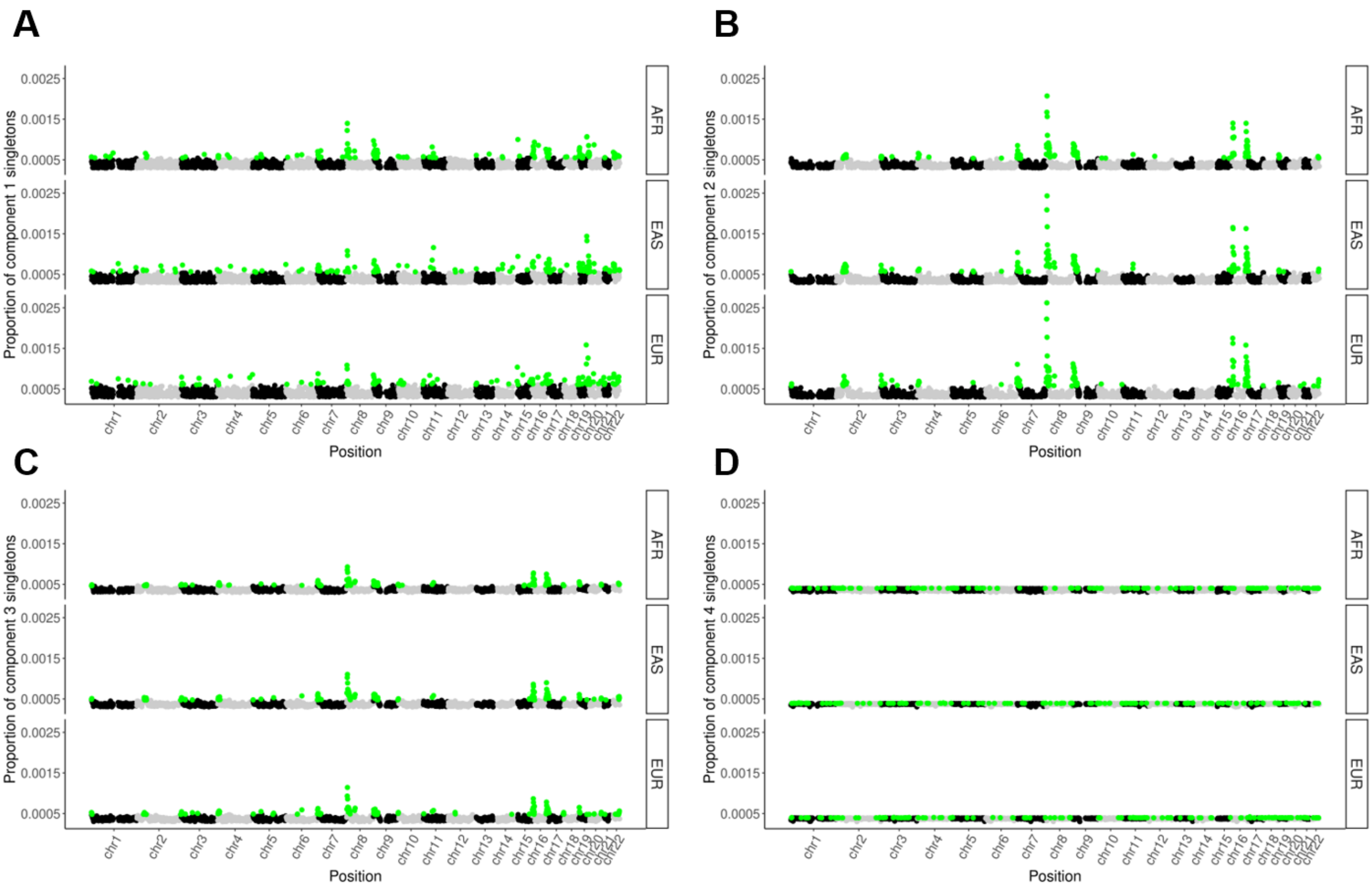

Supplementary Figure 22. Genomic hotspots for different singleton cluster classes. Density of singletons assigned to cluster class 1 (**A**), class 2 (**B**), class 3 (**C**), and class 4 (**D**) in 1 Mbp windows across the genome, with the proportion of singletons of a given class in each window indicated on the y-axis. For each cluster class, windows with singleton counts above the 95th percentile (calculated genome-wide per population subsample) are classified as hotspots and are highlighted in green (as in Extended Data Figure 3b). Note that all panels are shown on the same y-axis scale.

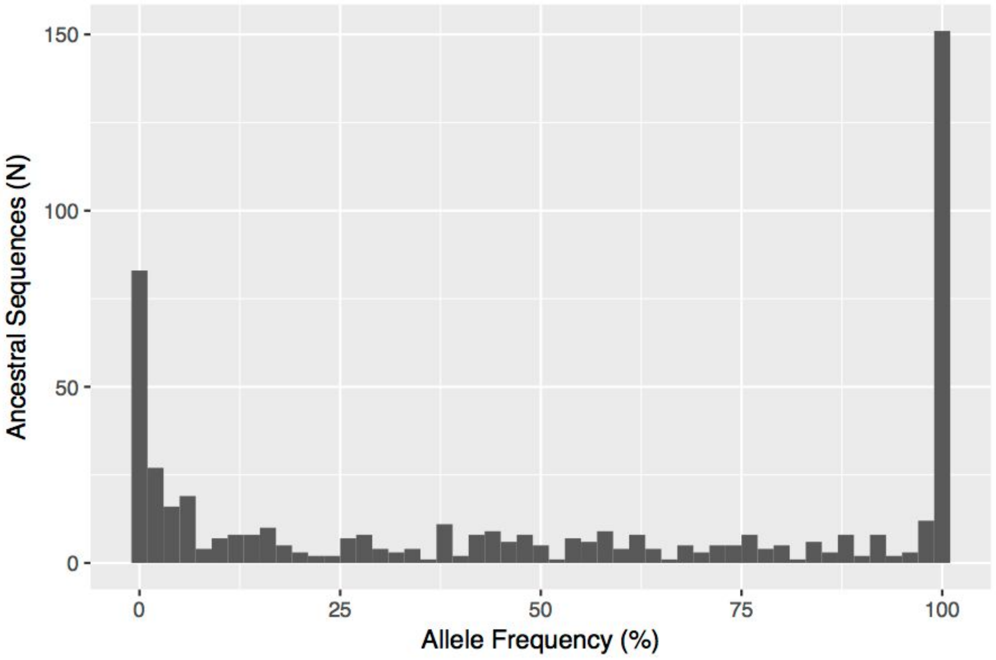

Supplementary Figure 23. Allele frequency distribution across unrelated samples of fully resolved non-reference ancestral sequences.

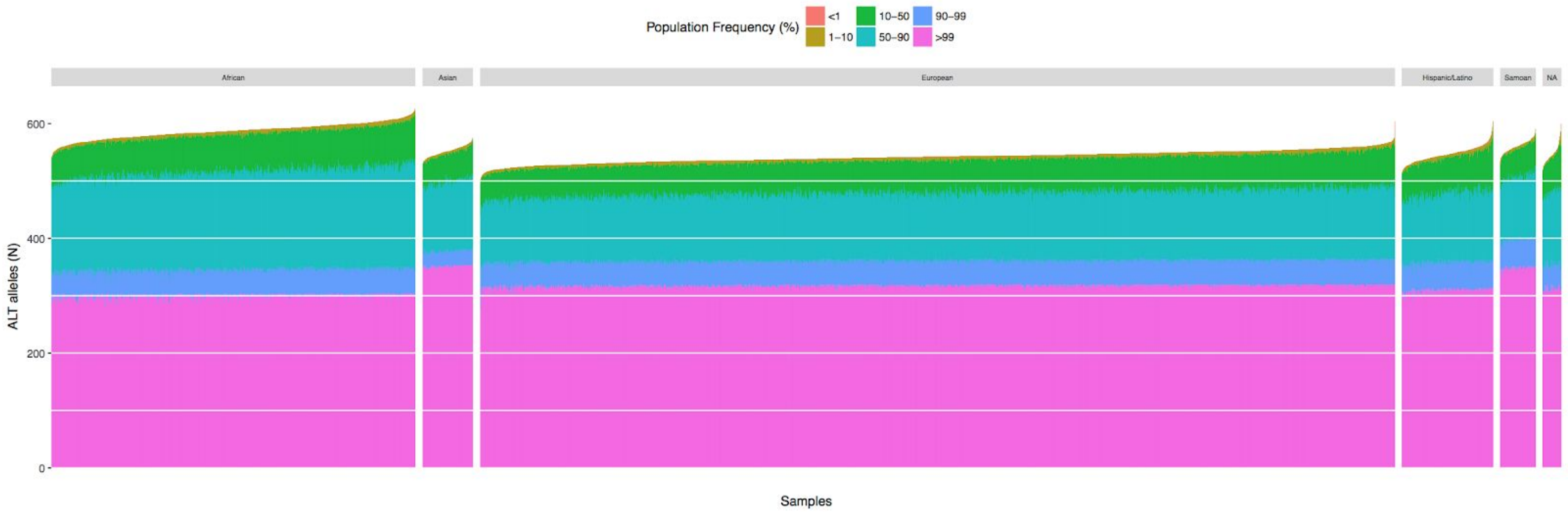

Supplementary Figure 24. Number of non-reference (ALT) alleles per individual, categorized by population-specific allele frequency. Individuals have been separated by population and sorted by the total number of non-reference alleles.

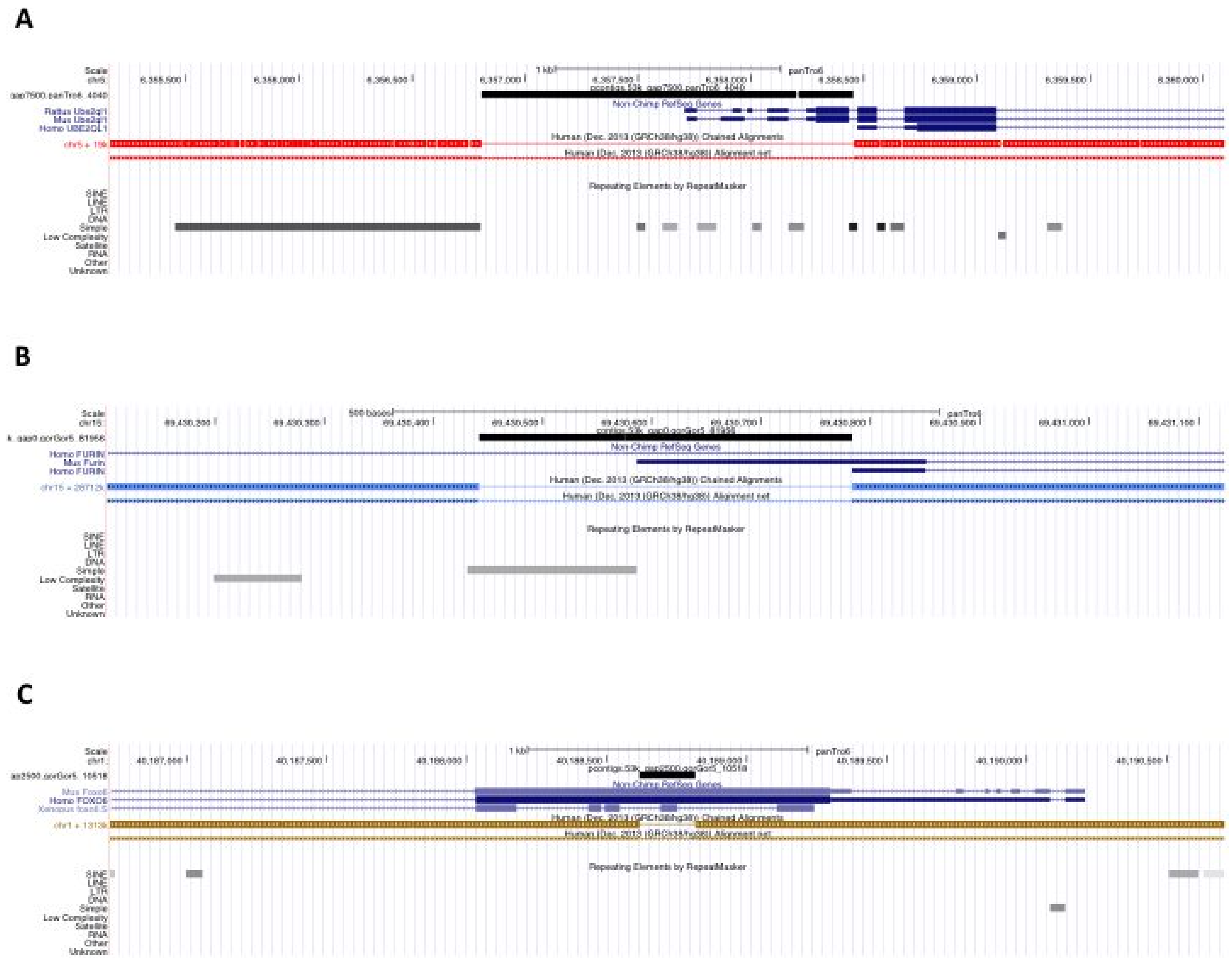

Supplementary Figure 25. UCSC Genome Browser screenshots of non-reference ancestral human sequences aligned to the chimp genome (panTro6). A. Sequence overlapping both the transcription and translation start sites of the *UBE2QL1* gene from mouse and rat that

have been aligned to the same reference. **B.** Sequence overlapping the transcription start site of mouse *FURIN* gene. **C.** Sequence overlapping a coding region of *FOXO6* transcripts from both human and mouse. The alignment gaps visible in the Chain and Net tracks (in red, blue and brown on panels A, B and C, respectively) reflect missing sequence in the human GRCh38/hg38 reference.

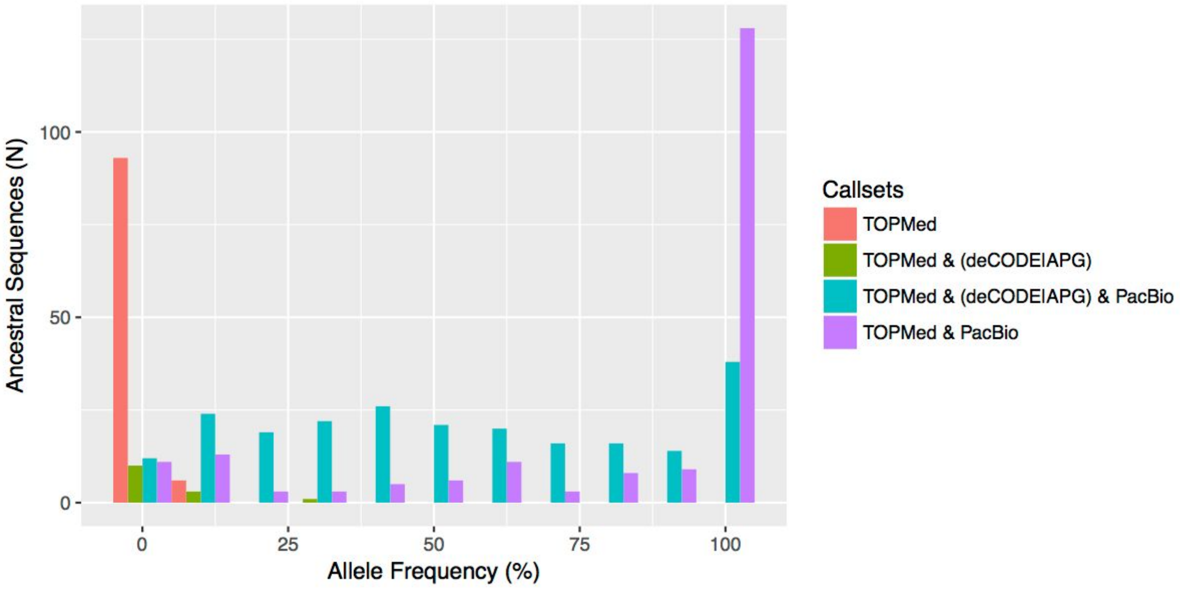

Supplementary Figure 26 Allele frequency distribution of non-reference ancestral sequences, categorized by overlap with insertions from 3 other studies by Sherman et al. (2018)[1], Kehr et al. (2017)[2], and Audano et al. (2019)[3].

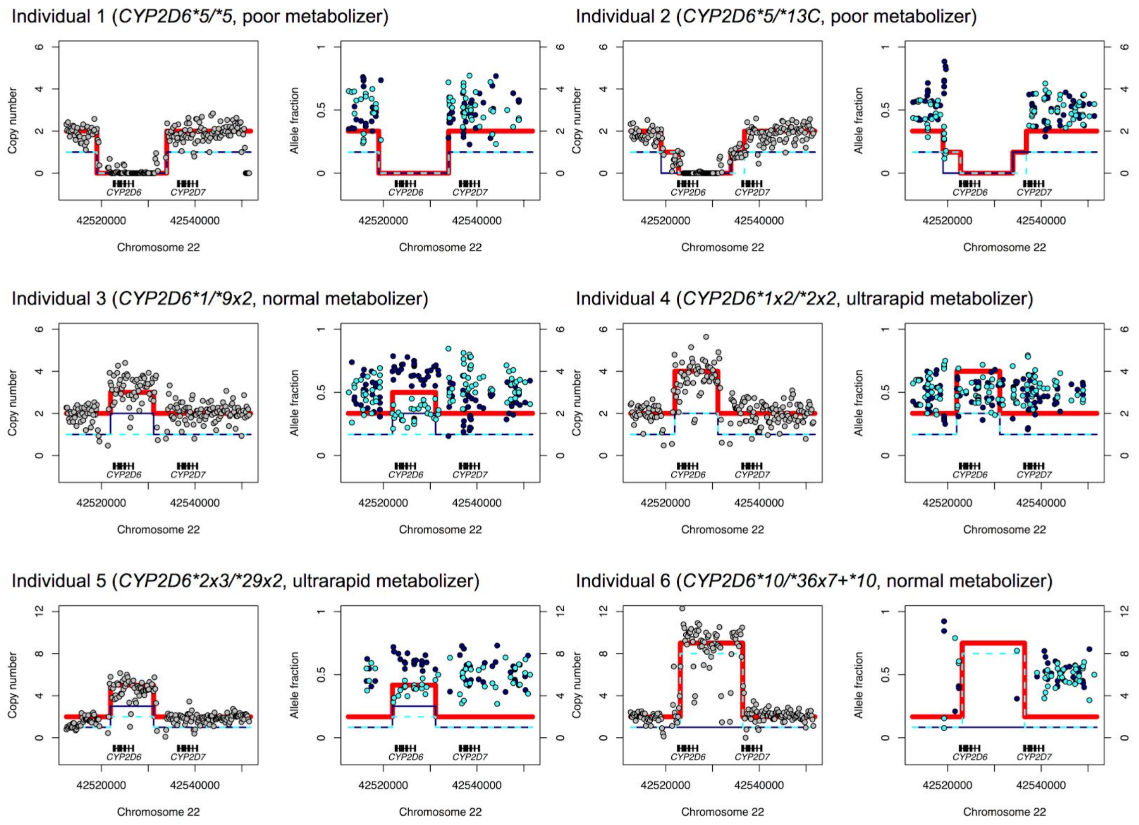

Supplementary Figure 27. Examples of *CYP2D6* star alleles (haplotypes) with structural variation detected by the Stargazer program. Each panel displays Stargazer's copy number profile (left) and allele fraction profile (right) for an individual sample (N=6). Also shown are *CYP2D6* diplotypes and phenotype predictions from Stargazer. Gray dots indicate the sample's per-base copy number estimates computed from read depth. The navy solid line and the cyan dashed line represent copy number profiles for each haplotype. The red line represents the copy number profile for both haplotypes combined. Navy dots and cyan dots indicate allele fraction estimates computed from allelic read depth for each haplotype. More examples can be found in the Database of Pharmacogenomic Structural Variants or DPSV (https://stargazer.gs.washington.edu/stargazerweb/res/dpsv.html).

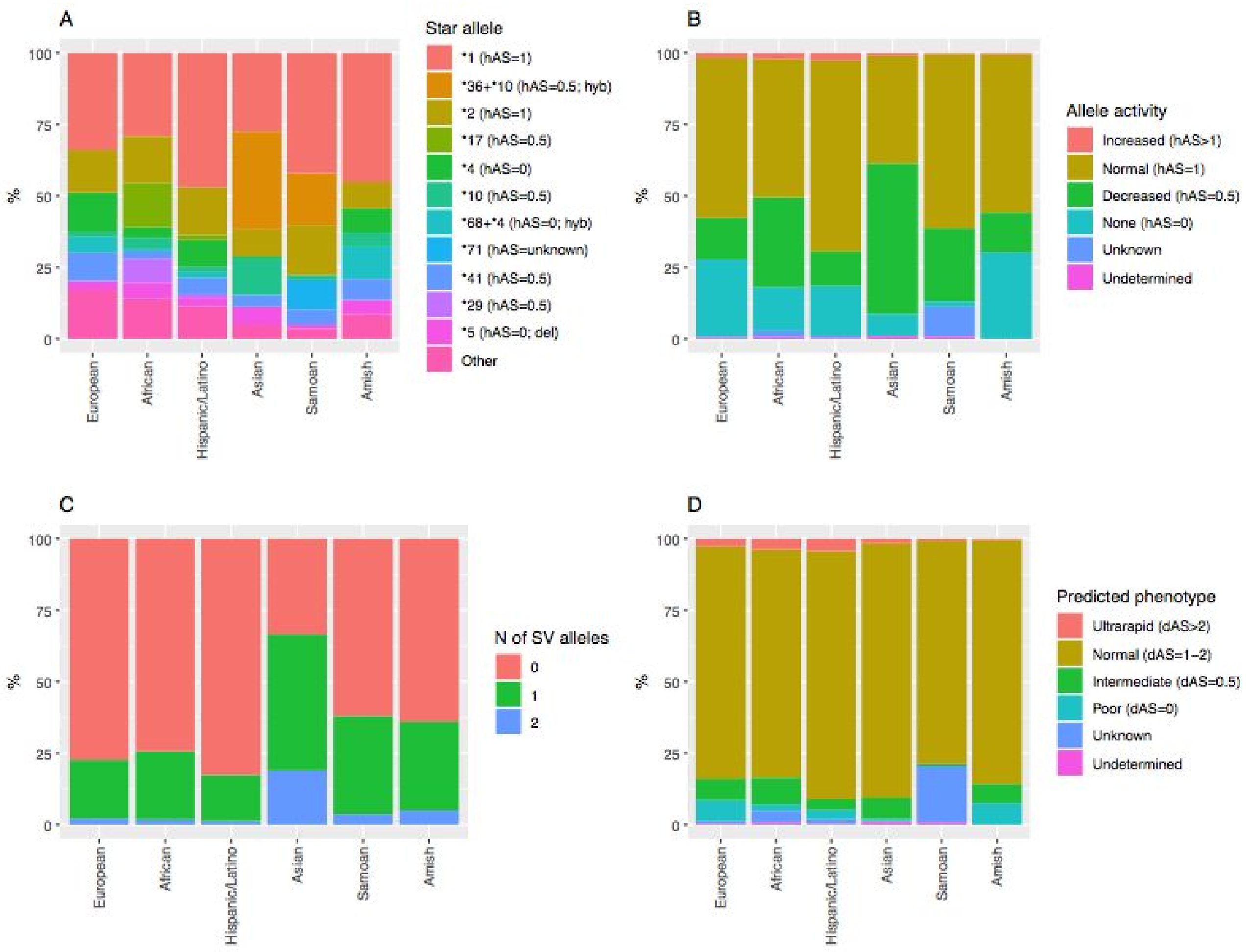

Supplementary Figure 28. Summary of *CYP2D6* haplotype analysis using the Stargazer program. Population-specific frequencies for (A) common *CYP2D6* star alleles, (B) haplotype activity, (C) SV-defined haplotypes, and (D) predicted metabolism phenotypes. Abbreviations: hAS, haplotype activity score; dAS, diplotype activity score; N, number; SV, structural variation; del, whole gene deletion; hyb, *CYP2D6/CYP2D7* hybrid.

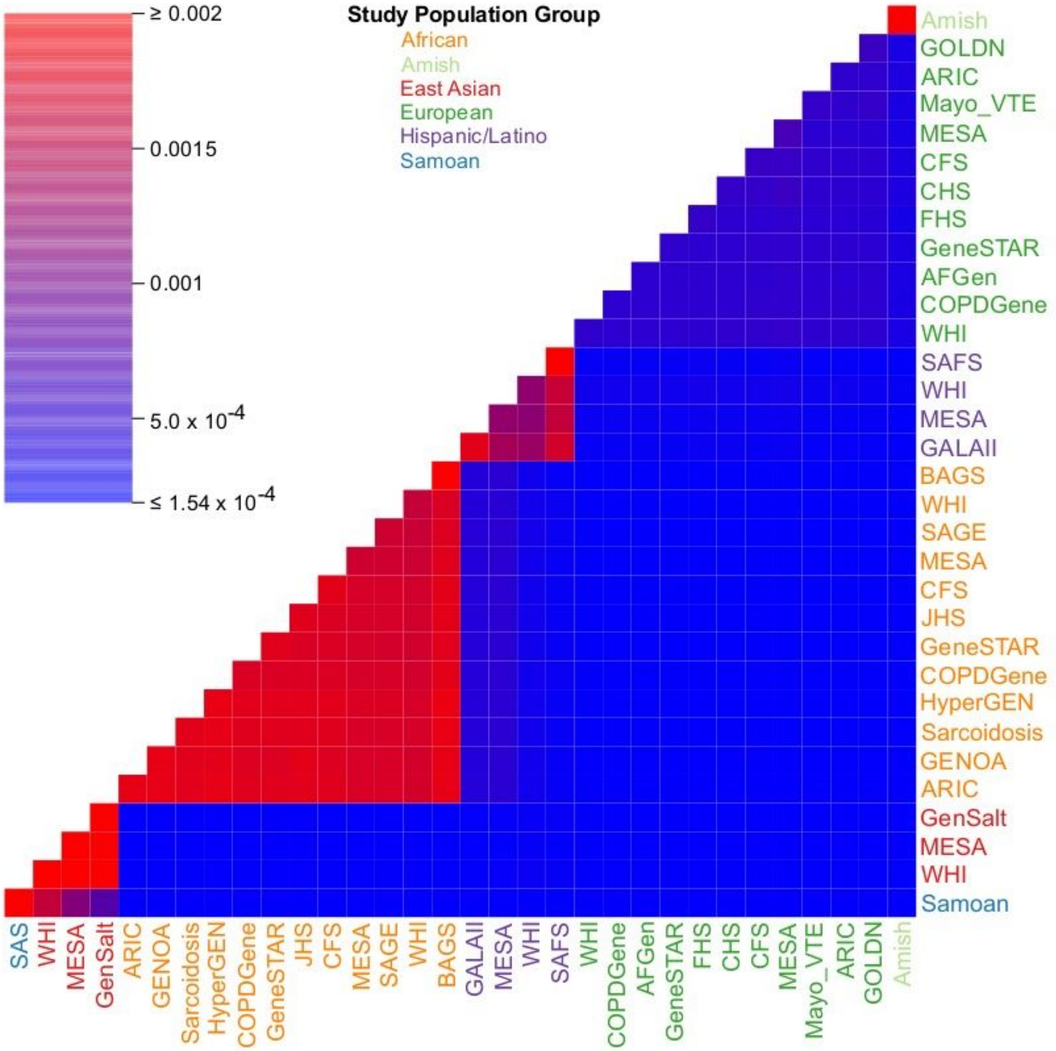

Supplementary Figure 29. Rare variant sharing between and within TOPMed studies. Each study label is colored based on population group. The heatmap scale depicts the 50th percentile of between and within rare variant sharing values.

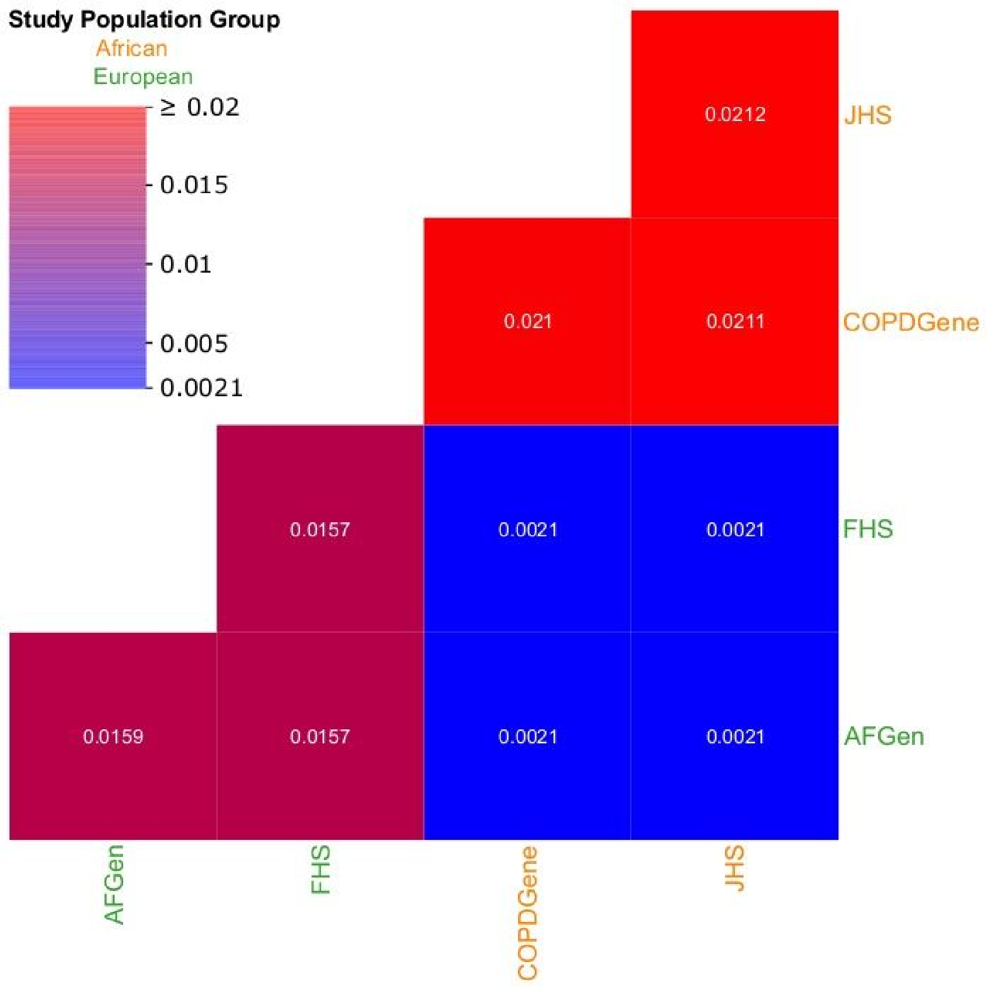

Supplementary Figure 30. Equal Study Sizes Rare Variant Sharing Control. Heatmap representation of sharing within and between 4 TOPMed studies each sampled to 500 individuals.

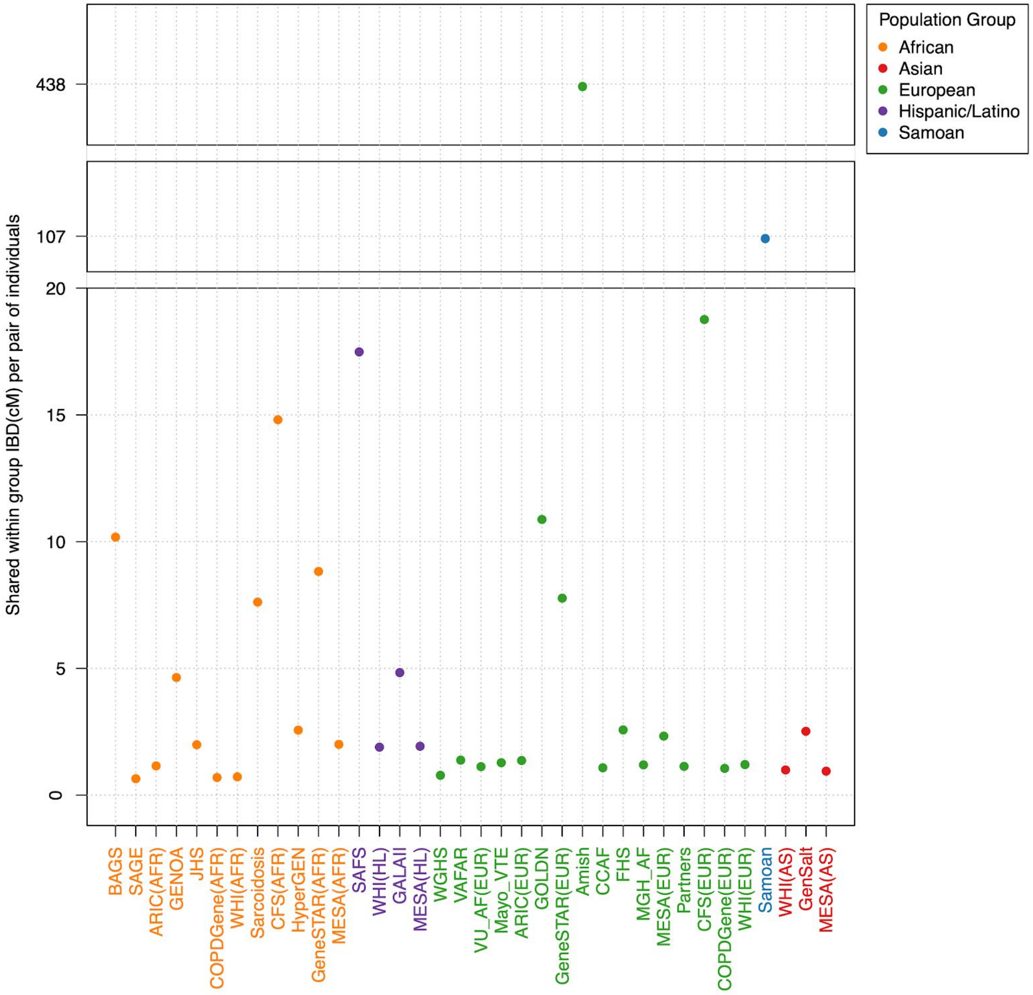

Supplementary Figure 31. Within-group average IBD sharing. We calculated the total autosome-wide length of detected IBD segments per pair of individuals and averaged across pairs within each population group. For studies with multiple population groups, parentheses after the study name identify the group (AFR, African; HL, Hispanic/Latino; EUR, European; AS, Asian). Points and labels are colored by population group.

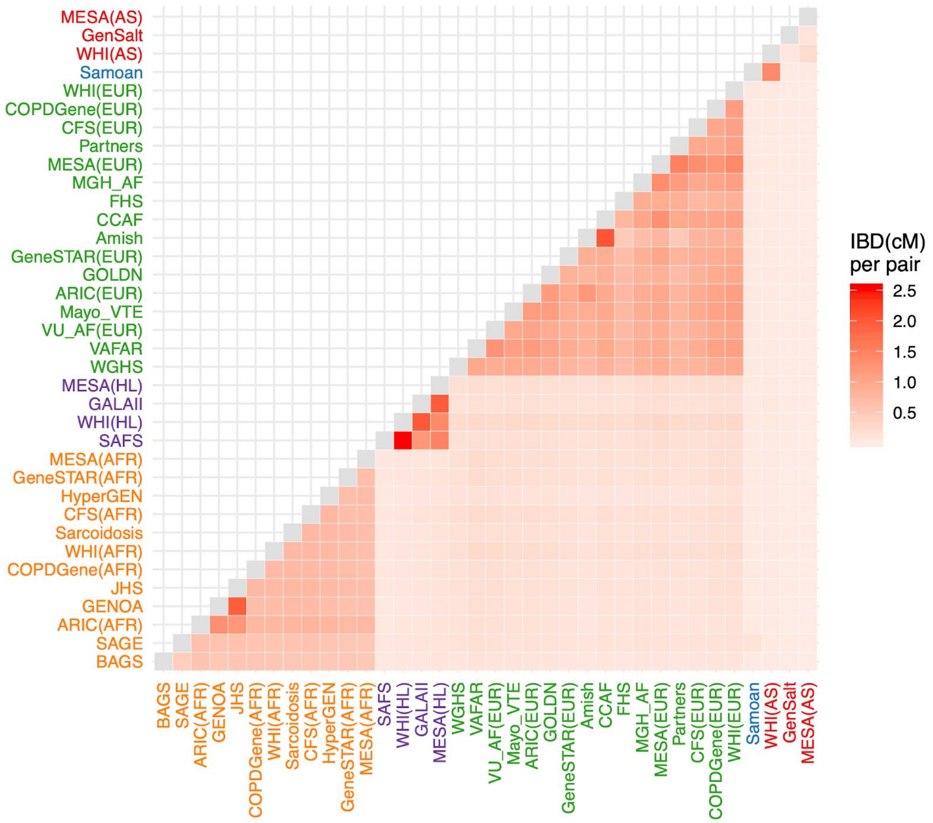

Supplementary Figure 32. Between group average IBD sharing. We calculated the total autosome-wide length of detected IBD segments per pair of individuals and averaged across pairs. For studies with multiple population groups, parentheses after the study name identify the group (AFR, African; HL, Hispanic/Latino; EUR, European; AS, Asian). Labels are colored by population group (Asian, red; Samoan, blue; European, green; Hispanic/Latino, purple; African, orange).

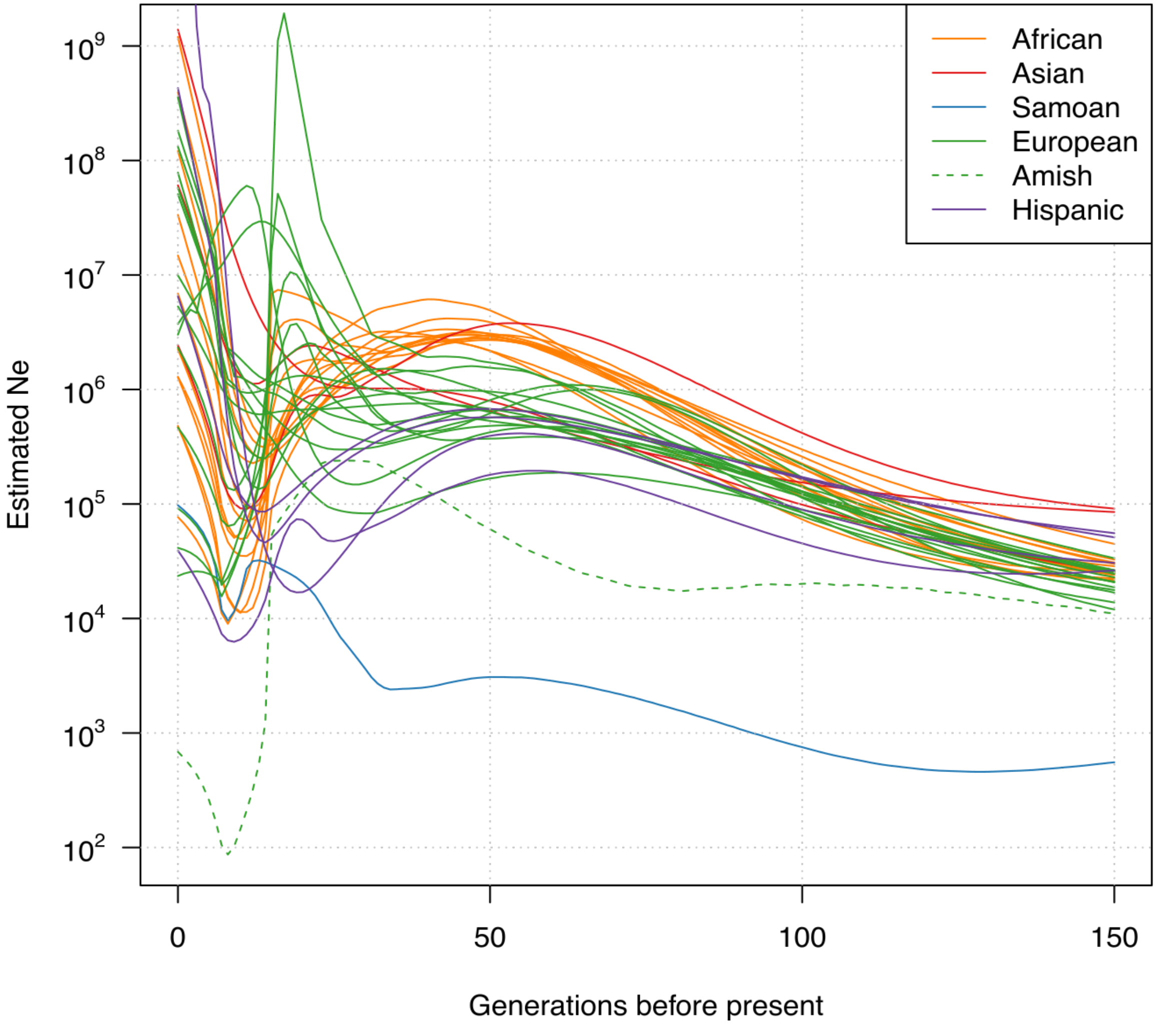

Supplementary Figure 33. Estimates of recent effective population size by population group. Each line represents the estimate from a single study, considering only individuals from that population group. The included studies are the same as those in Supplementary Figure 32. The Amish and Samoan results are individually identified due to their distinct recent population size trajectories. N_e indicates effective population size.

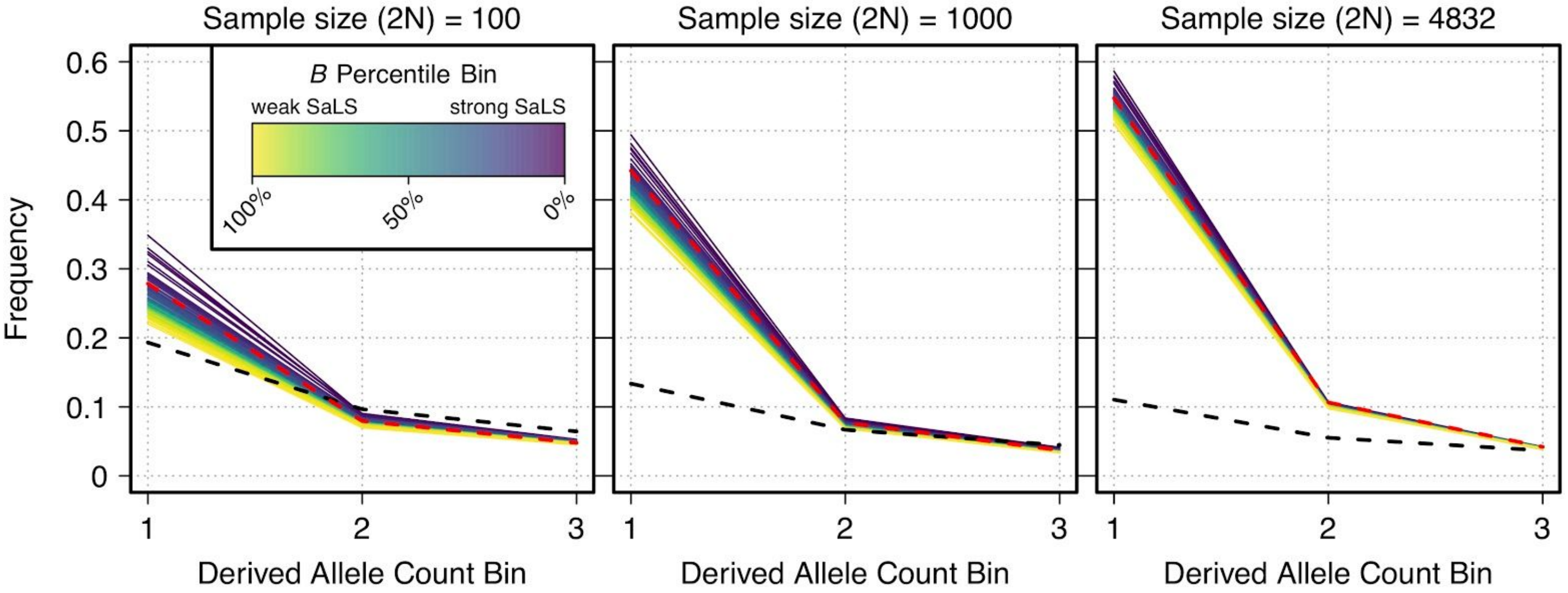

Supplementary Figure 34. Site-frequency spectrum (SFS) for different sample sizes and *B* for the first three derived allele counts. SFS data is shown for each of 100 percentile bins of *B* (McVicker's *B* statistic; higher percentiles of *B* indicate weaker effects of selection at linked sites [SaLS]). Each separate plot shows a different sample size from which the SFS was made. Dashed red lines show the SFS from fourfold degenerate sites. Dashed black lines show the SFS from a standard neutral model for the given sample size.

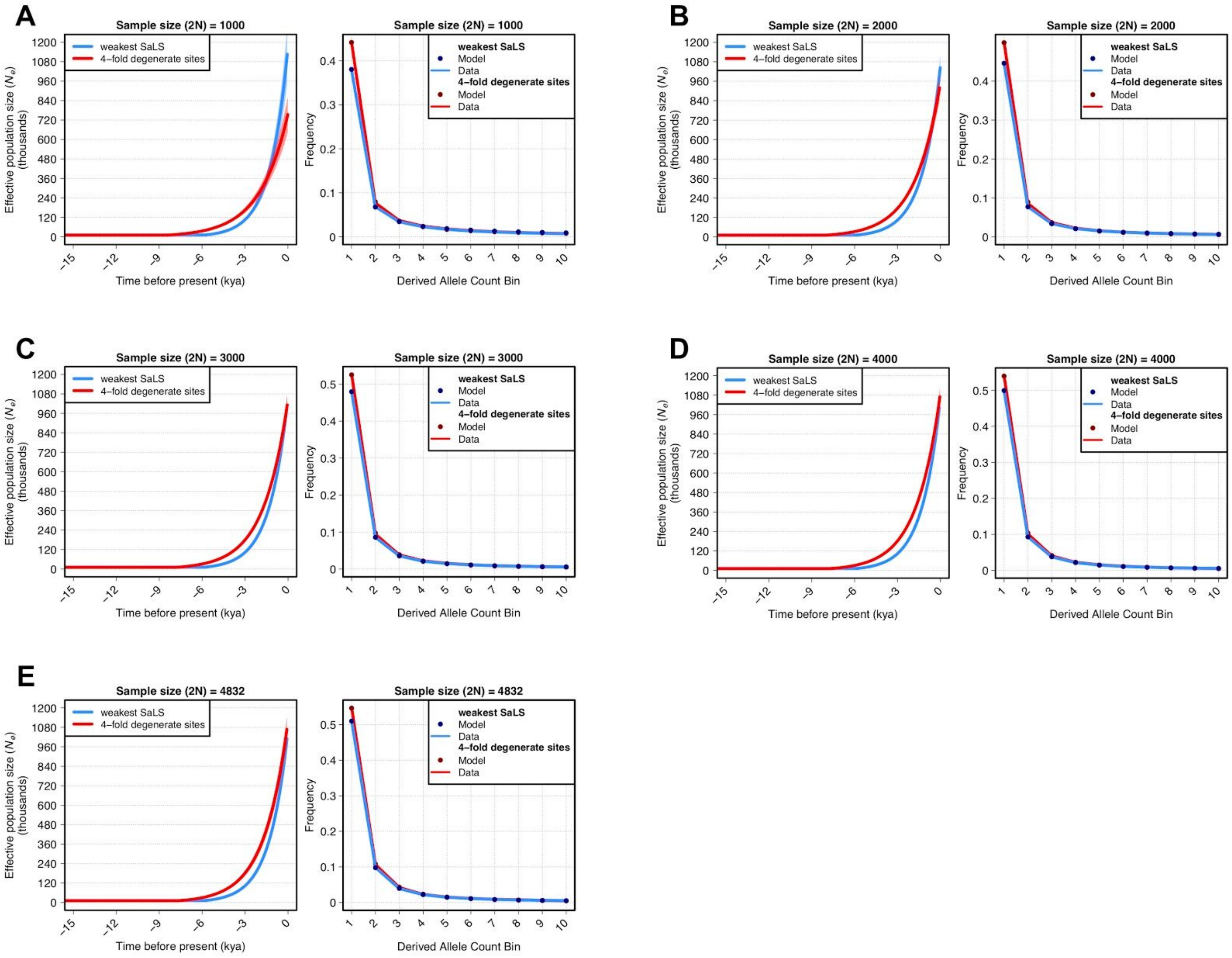
A
Sample size (2N) = 1000
weakest SaLS
4−fold degenerate sites
Effective population size (N_e) (thousands)
Time before present (kya)
Frequency
Model
Data
Derived Allele Count Bin
B
Sample size (2N) = 2000
C
Sample size (2N) = 3000
D
Sample size (2N) = 4000
E
Sample size (2N) = 4832

Supplementary Figure 35. Results from performing demographic inference fitting a model of exponential growth to fourfold degenerate sites and sites under the weakest effects of selection at linked sites (SaLS). Weakest SaLS represent sites from the highest 1% *B* bin (99-100% *B*; McVicker's *B* statistic). The left panels of each figure show the inferred exponential growth using various sample sizes. Shaded envelopes represent 95% confidence intervals (see Supplementary Table 14 for parameter values). The right panels of each figure show the observed site-frequency spectrum represented as solid lines. Number of sites from the site-frequency spectrum used for demographic inference for 4-fold degenerate sites was N=4,718,653 sites and for highest 1% *B* sites was N=10,977,437 sites. The resulting fits to the site-frequency spectrum from the fitted demographic models are shown as points.

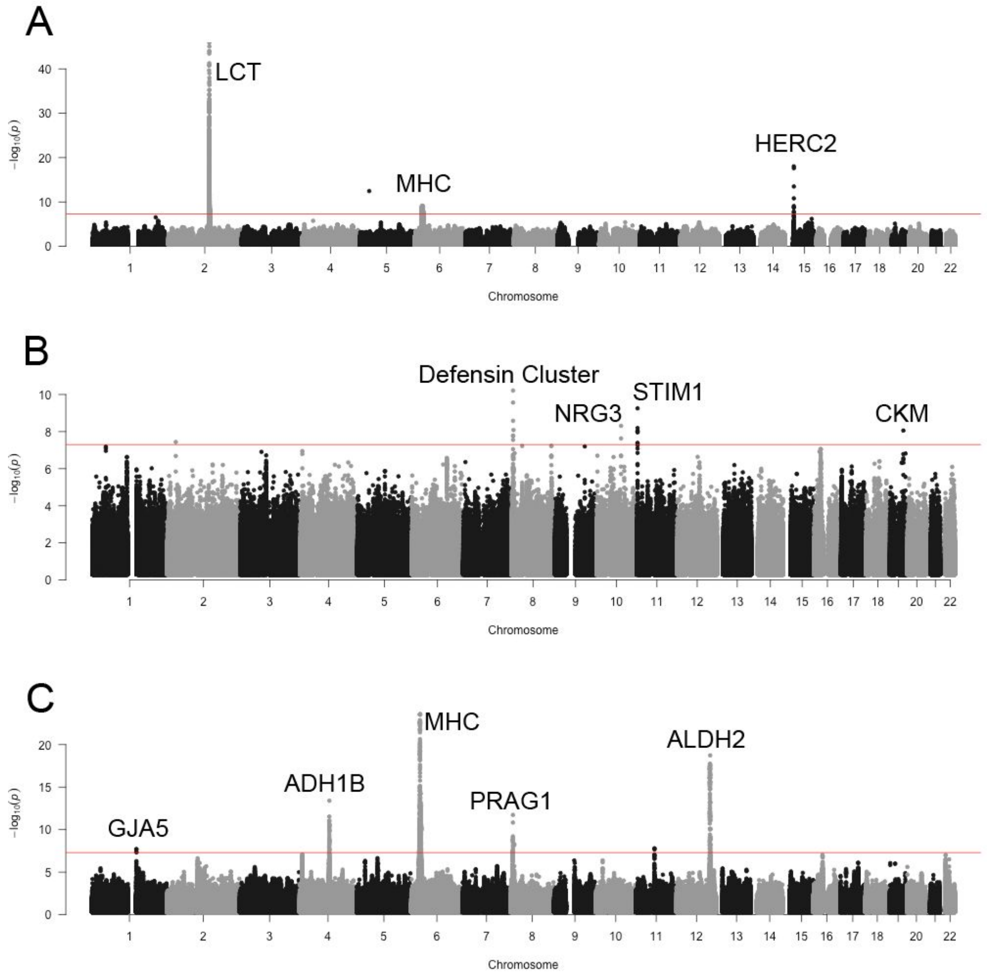

Supplementary Figure 36. Manhattan plot of SDS *P*-values in each population. A. European. **B.** African. **C.** East Asian. P-values are two-sided tail probabilities of standard normal distribution. Horizontal red line indicates genome-wide significance threshold after adjustment for multiple testing, $p = 5\times10^{-8}$.

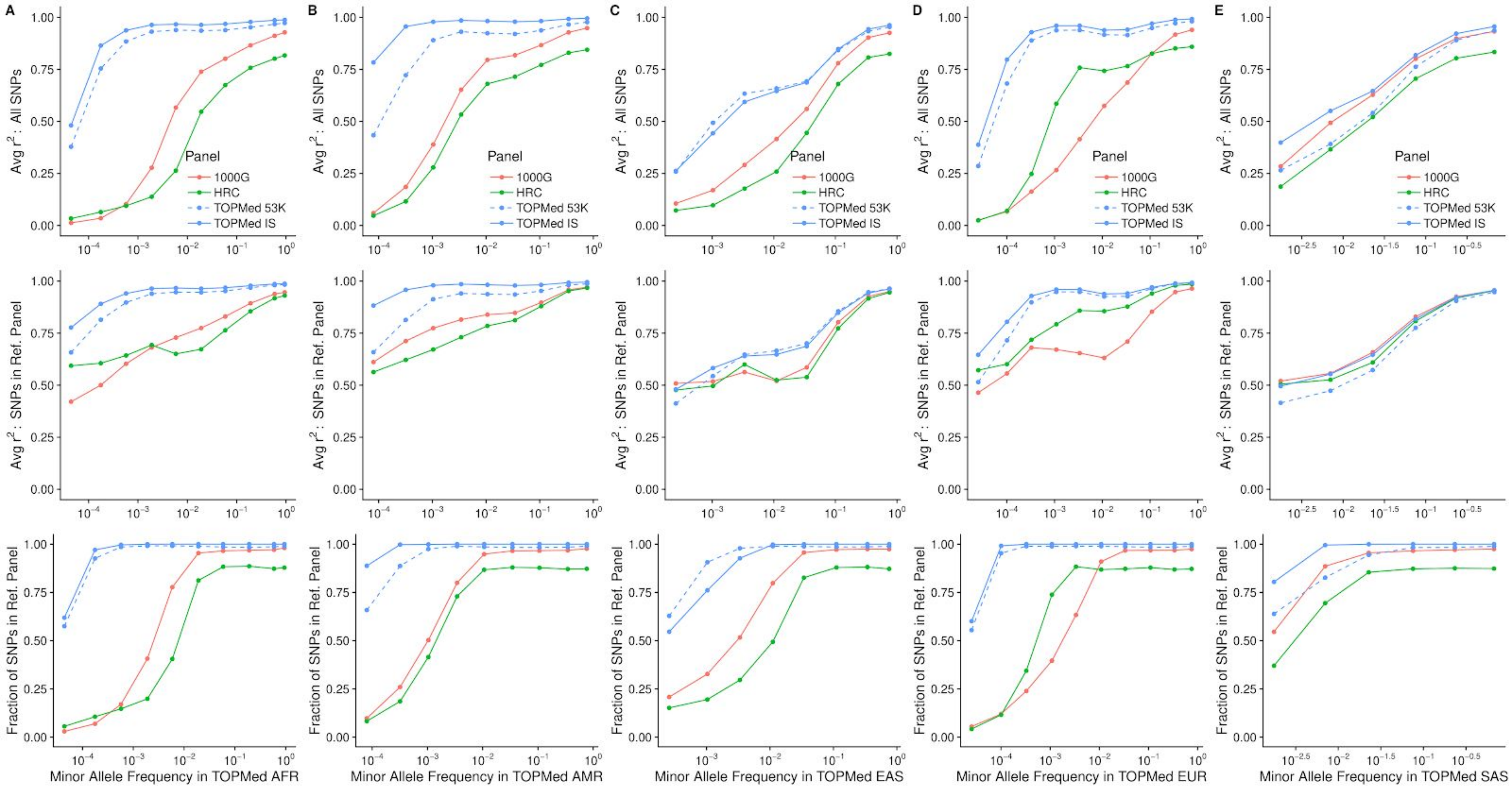
A
B
C
D
E
Avg r²: All SNPs
Avg r²: SNPs in Ref. Panel
Fraction of SNPs in Ref. Panel
Panel
1000G
HRC
TOPMed 53K
TOPMed IS
Minor Allele Frequency in TOPMed AFR
Minor Allele Frequency in TOPMed AMR
Minor Allele Frequency in TOPMed EAS
Minor Allele Frequency in TOPMed EUR
Minor Allele Frequency in TOPMed SAS

Supplementary Figure 37. Evaluation of imputation accuracy. Evaluation of genotype imputation accuracy from various reference panels - 1000 Genomes Phase 3 Panel (1000G)[4], Haplotype Reference Consortium Panel (HRC)[5], panel based on 53,831 TOPMed samples (TOPMed 53K), and TOPMed Imputation Server Panel (TOPMed IS). Each column represents a different continental population matched with five 1000 Genomes continental populations, namely (A) AFR : Africans, (B) AMR : Admixed Americans, (C) EAS : East Asians, (D) EUR : Europeans, (E) SAS : South Asians. 100 samples from the BioMe study that were not included in the imputation panel are selected from each continental population, and population-specific allele frequencies are calculated excluding the selected target samples. Top panels show the average squared correlation (r2) between the sequence-based genotypes and imputed dosages across all variants, assigning r2 = 0 to variants absent from each Reference Panel. The middle panels compute average r2 with only the variants present from each Reference Panel. The proportion of variants present in the reference panels is shown in the bottom panel.

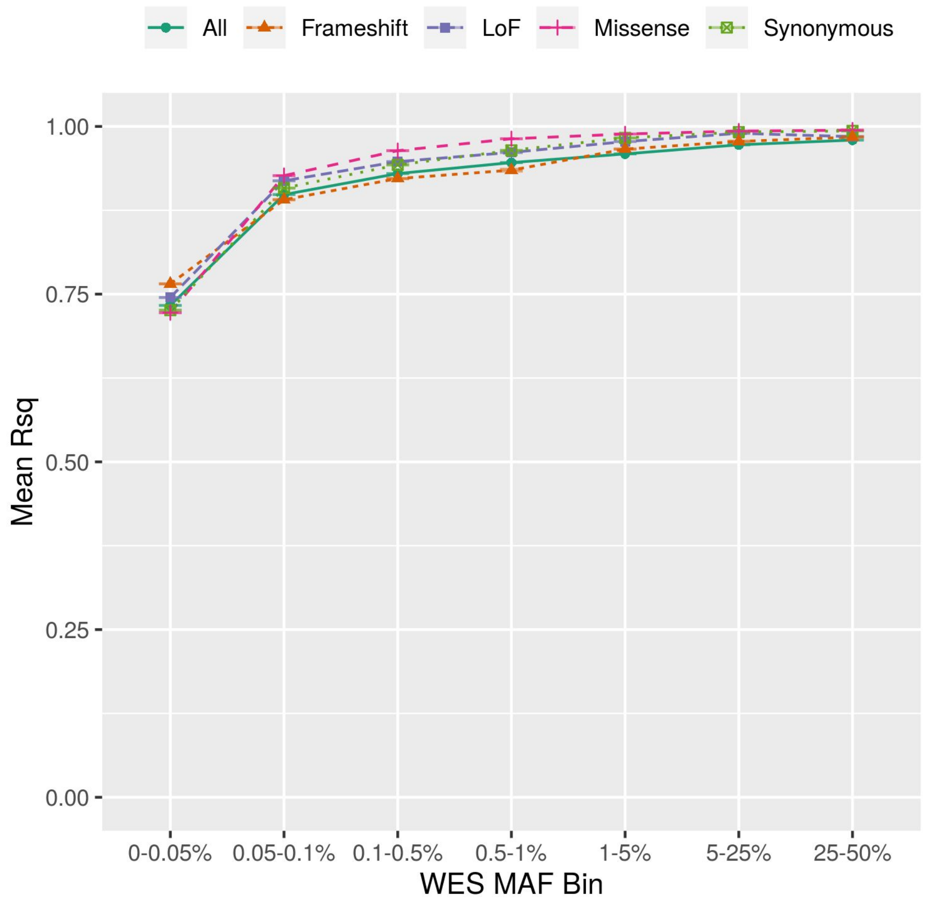

Supplementary Figure 38. Correlation between TOPMed-imputed and whole exome sequenced genotypes in UK Biobank individuals.

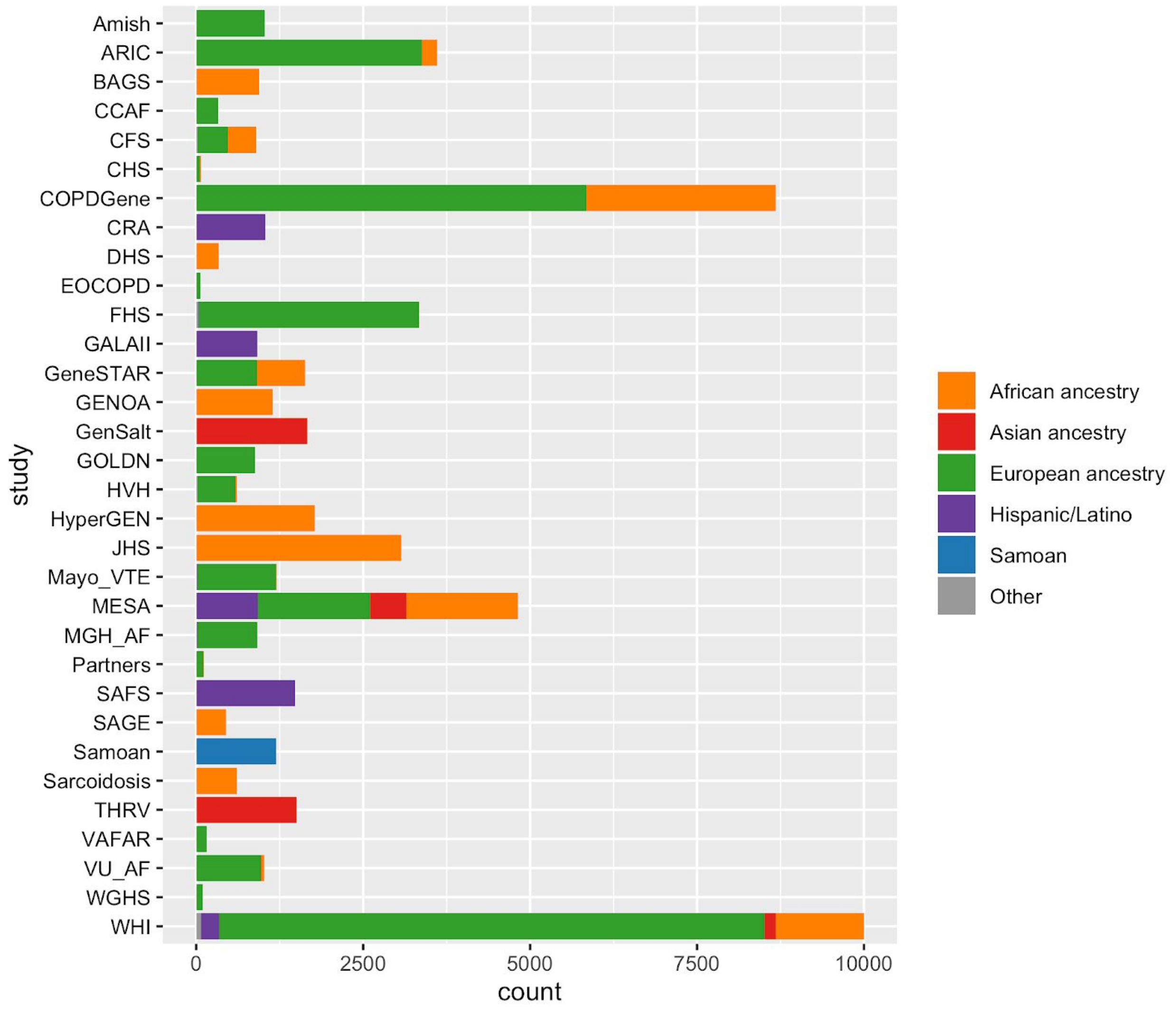

Supplementary Figure 39. Ancestral/ethnic composition of studies included in the TOPMed Freeze 5 genotype call set. These counts are based on participant responses to questions regarding race and ethnicity and/or study recruitment criteria, and were used to define population groups. See Extended Data Table 2 for study abbreviations.

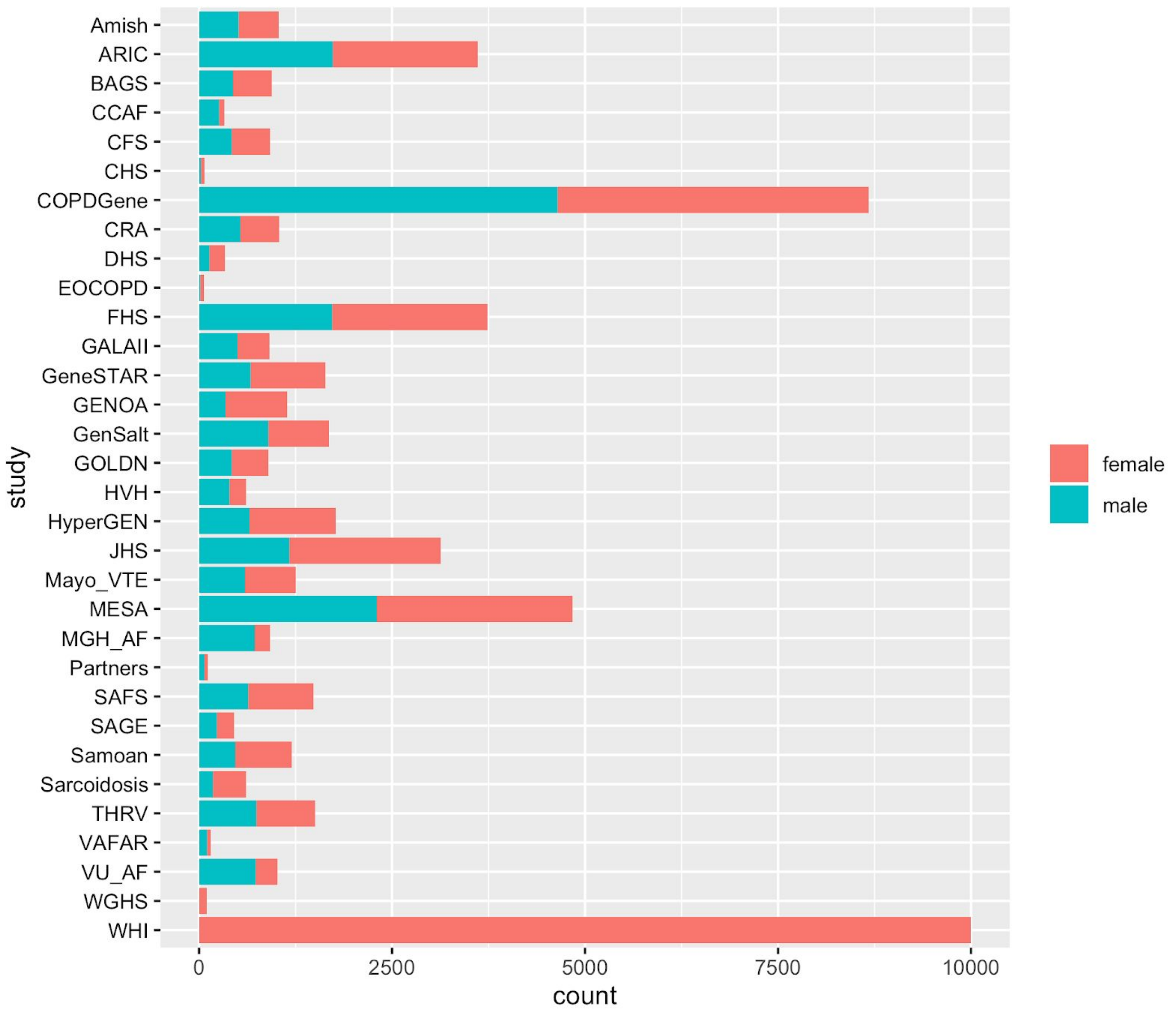

Supplementary Figure 40. Sex composition of studies included in the TOPMed Freeze 5 genotype call set. See Extended Data Table 2 for study abbreviations.

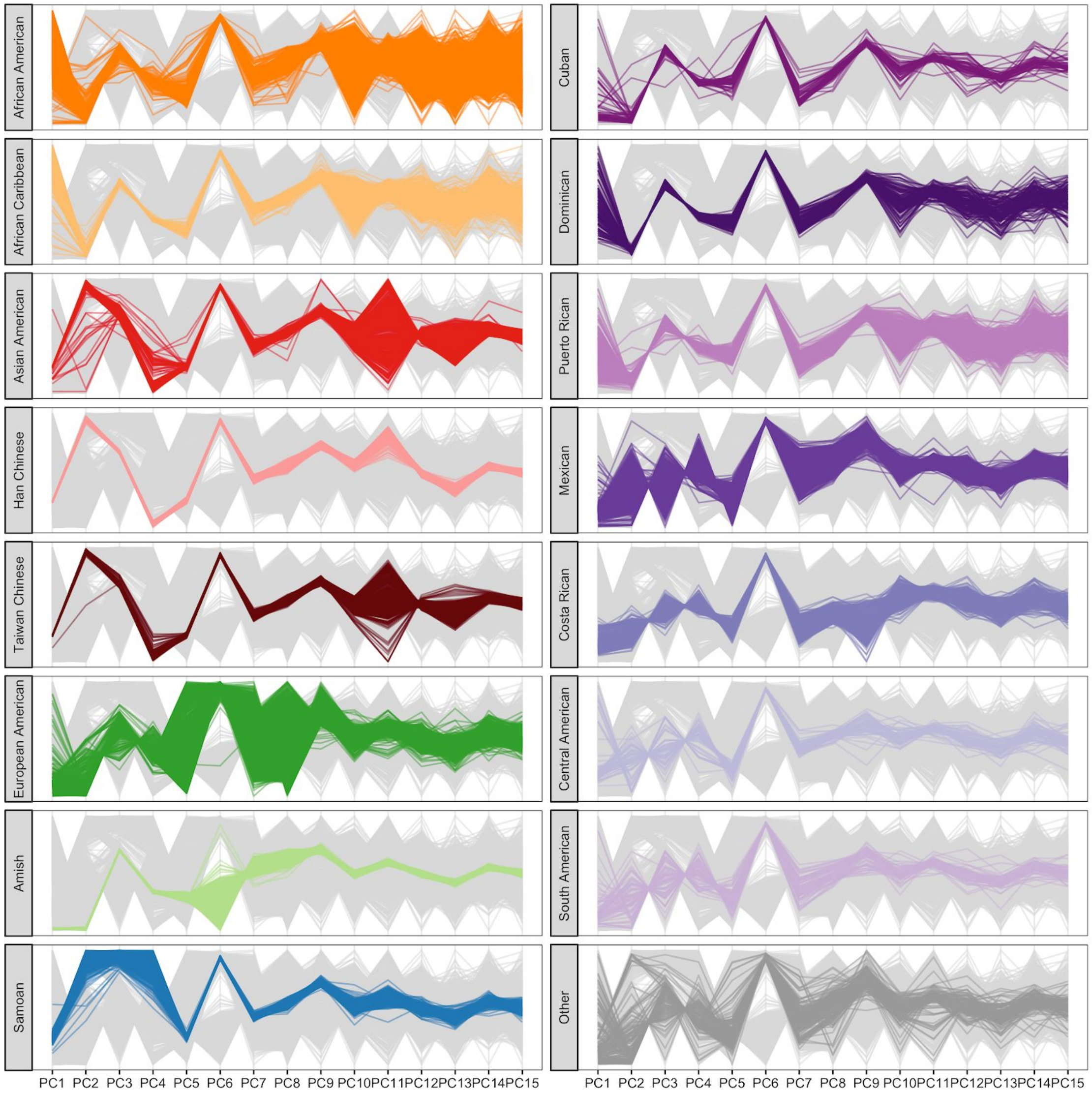

Supplementary Figure 41. Parallel coordinates plots for the first 15 principal components of Freeze 5 genotype data pooled across studies. All panels contain the same set of lines, but each panel highlights a single category reflecting race, ancestry, and/or ethnic information provided by the participants or specified by study inclusion criteria.

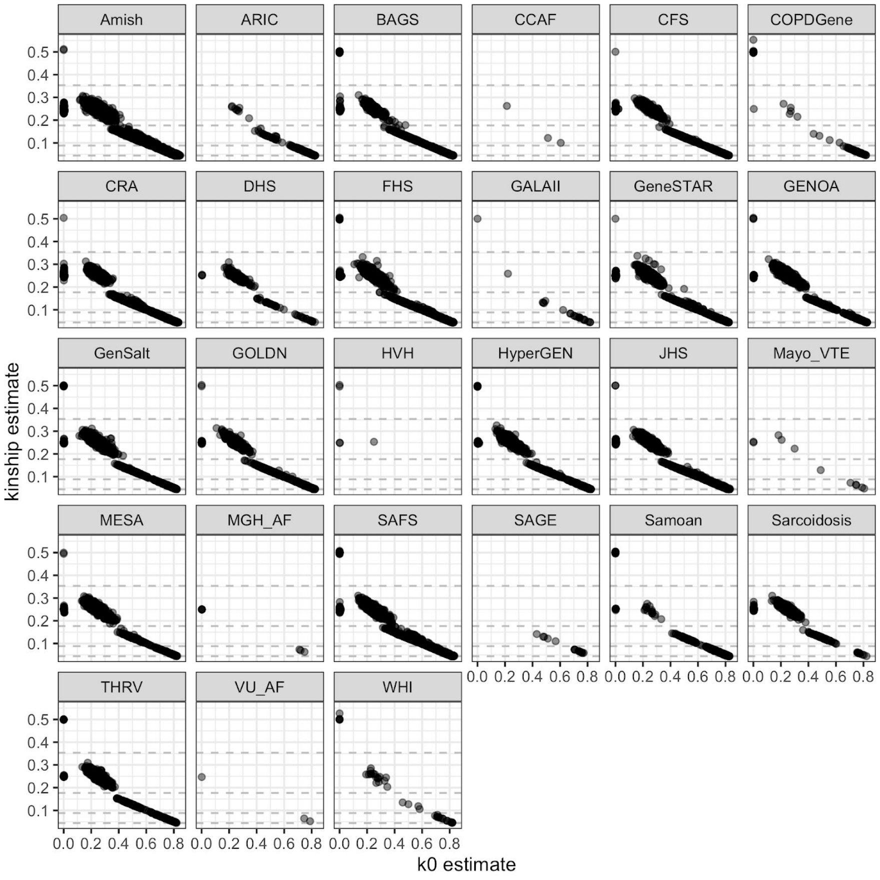

Supplementary Figure 42. Relatedness of subjects within each study. The y-axis shows the kinship coefficient (KC) estimated by PC-Relate. The kinship coefficient for a pair of participants is KC = k2/2 + k1/4, where k2 is the probability that two pairs of alleles are identical by descent (IBD) and k1 is the probability that one pair of alleles is IBD. The x-axis shows k0, the probability that zero alleles are identical by descent. Each point represents a pair of samples. Gray dashed horizontal lines show boundaries for KC values for inferring varying degrees of relatedness. Moving from the top down, monozygotic twins are in the top left corner, the first and second dashed lines form a region for expected first-degree relatives (parent-offspring and full siblings), the second and third form a region for expected second-degree relatives, the

third and fourth for expected third-degree relatives, and below the fourth we expect unrelated or related at fourth or higher degree. See Extended Data Table 2 for study abbreviations.

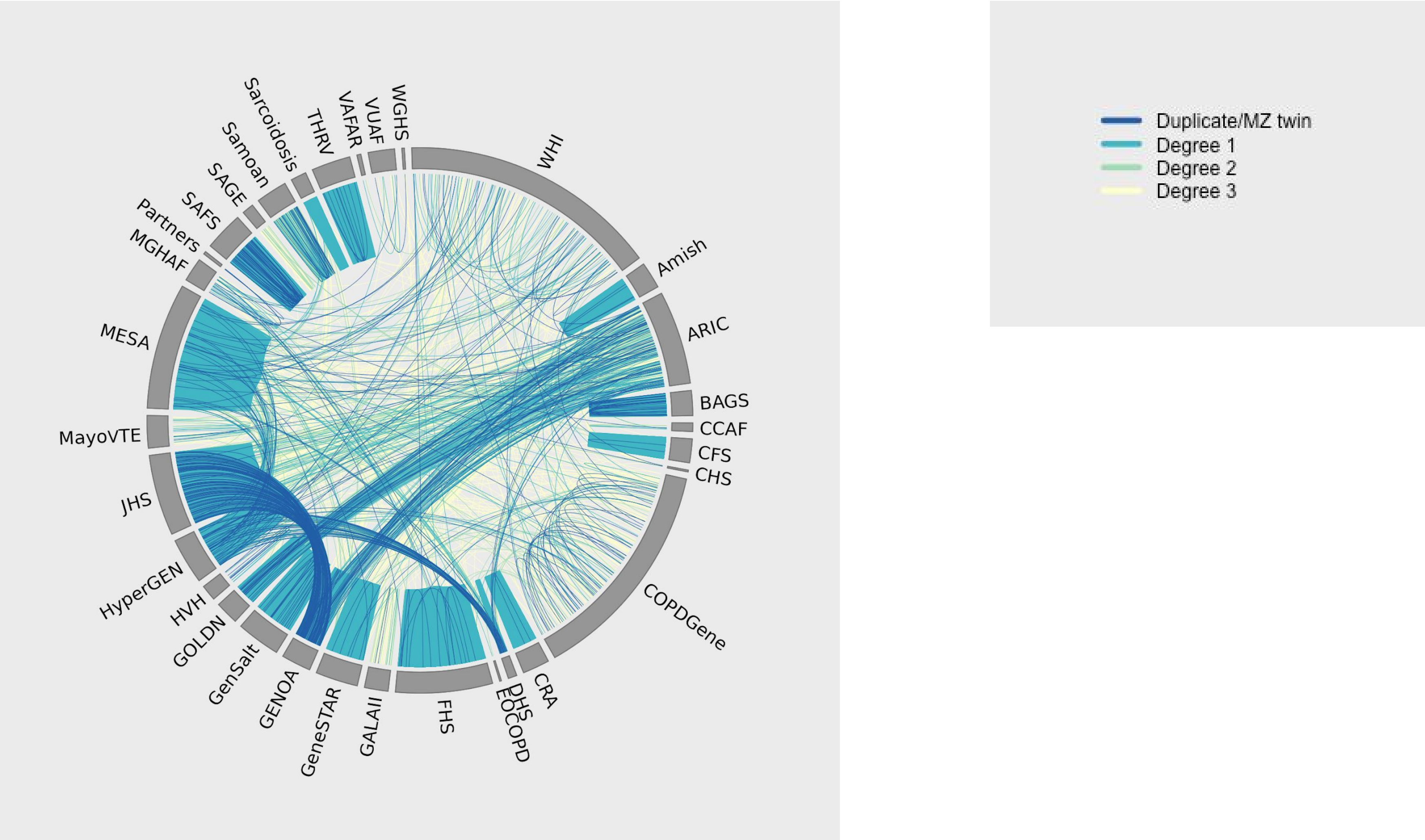

Supplementary Figure 43. Relatedness of subjects within and across studies. Line color indicates degree of relationship: blue=duplicates or monozygotic twins, blue-green=first-degree relatives (parent-offspring and full siblings), green=second-degree relatives, yellow=third-degree relatives. See Extended Data Table 2 for study abbreviations.

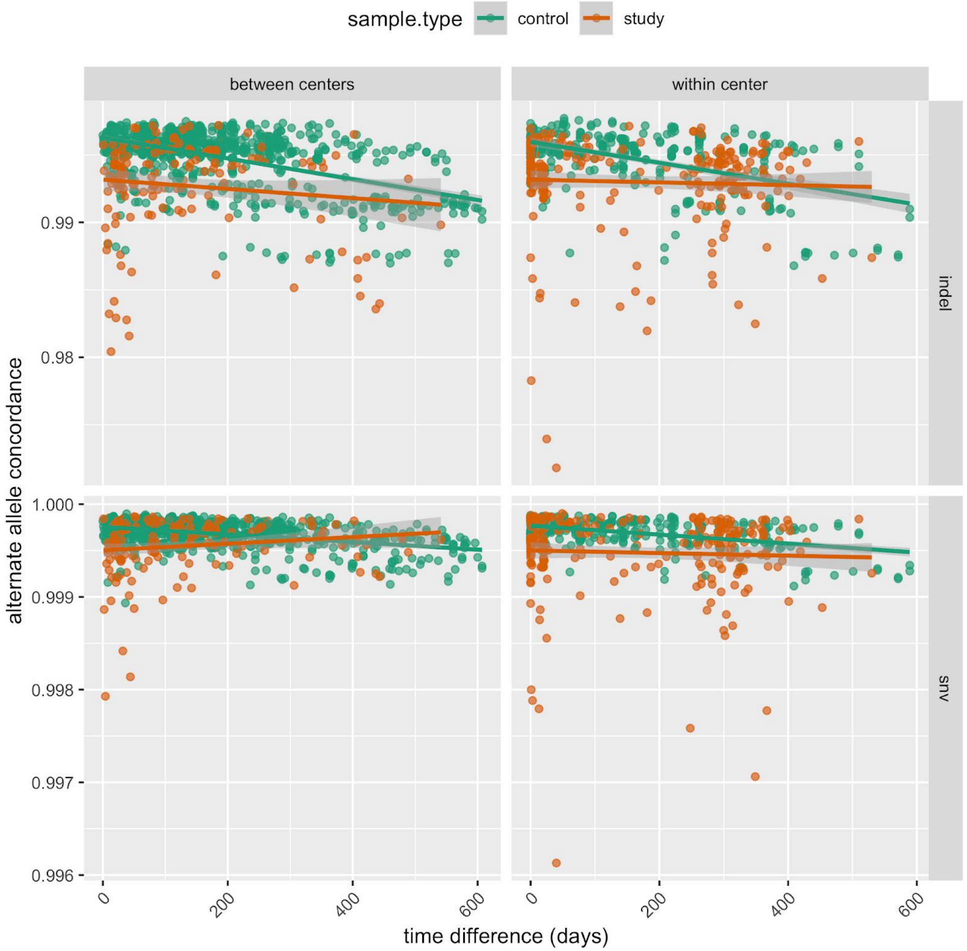

Supplementary Figure 44. Alternate allele concordance for duplicate samples sequenced at different times. Each point in these plots represents the concordance for a unique pair of samples, using passing variants only. The time difference axis represents the number of days between transmission of sequence data for a given sample between the center and the IRC (a proxy for the difference in actual sequencing dates). Control sample times tend to cluster because they are run at each center at regular intervals. The lines were derived from linear regression performed separately for control and study samples.

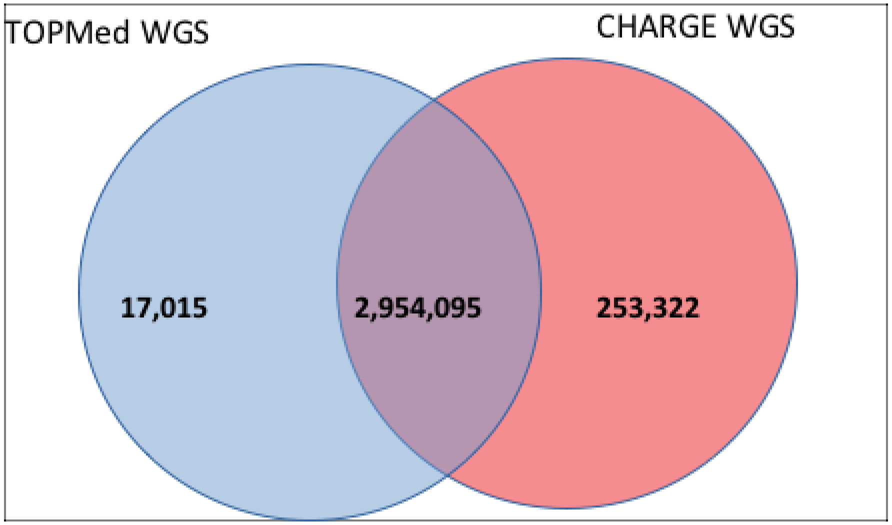

Supplementary Figure 45. Common variants overlap between TOPMed WGS and CHARGE WGS.

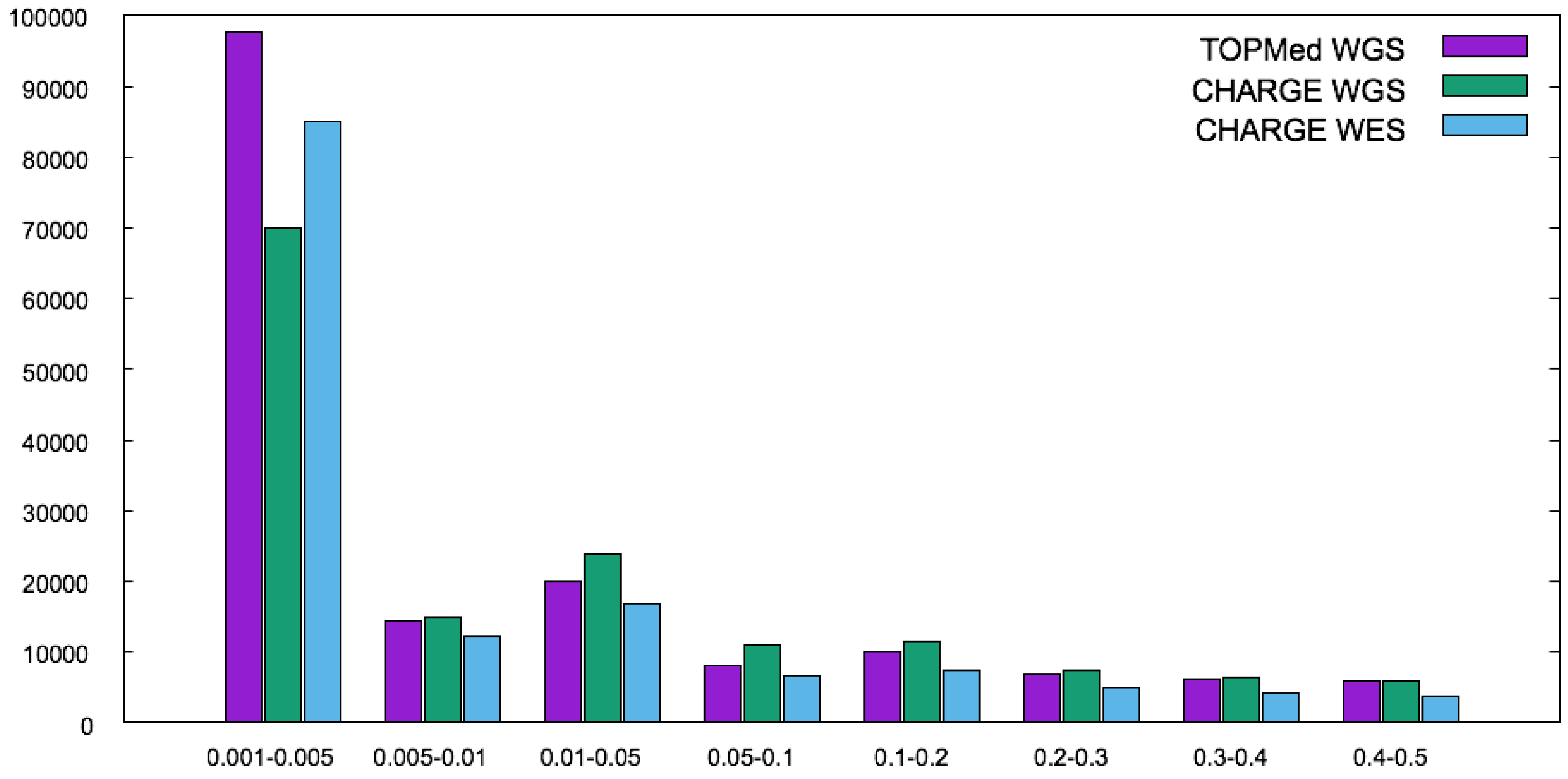

Supplementary Figure 46. Exonic bi-allelic SNV counts by minor allele frequency. The X axis is minor allele frequency bins and the Y axis is SNV count in each bin.

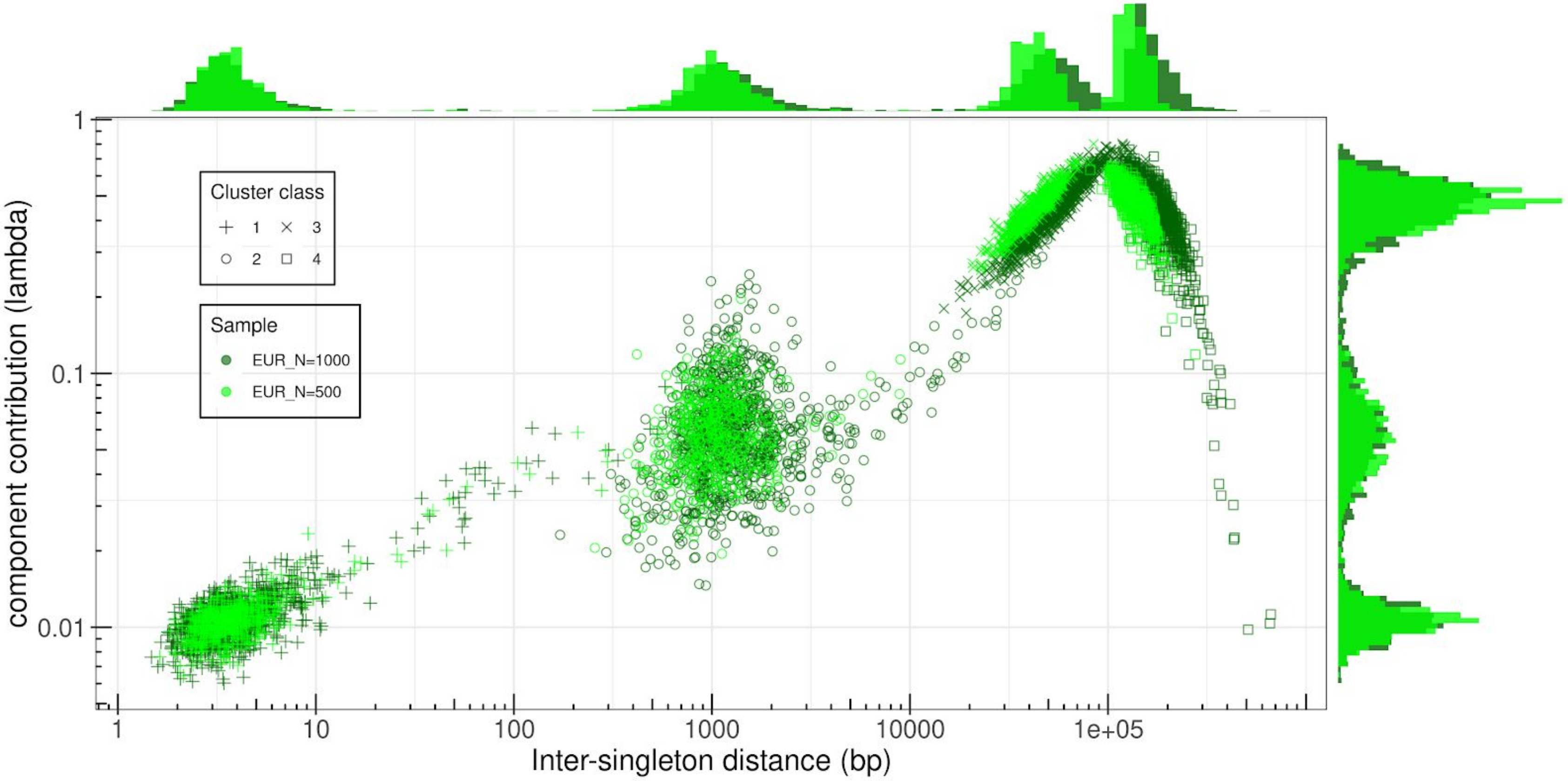

Supplementary Figure 47. Parameter estimates for exponential mixture models of singleton density, applied to different sample sizes of individuals with European ancestry. Each point represents one of the four components in one of the 1,500 individuals in the sample, colored by the majority ancestry of that individual. The rate parameters of each component are shown across the x-axis, and the lambda parameters (i.e., the proportion that component contributes to the mixture) on the y-axis (on a log-log scale). Marginal histograms show the distribution of the lambda and rate parameters for each component.

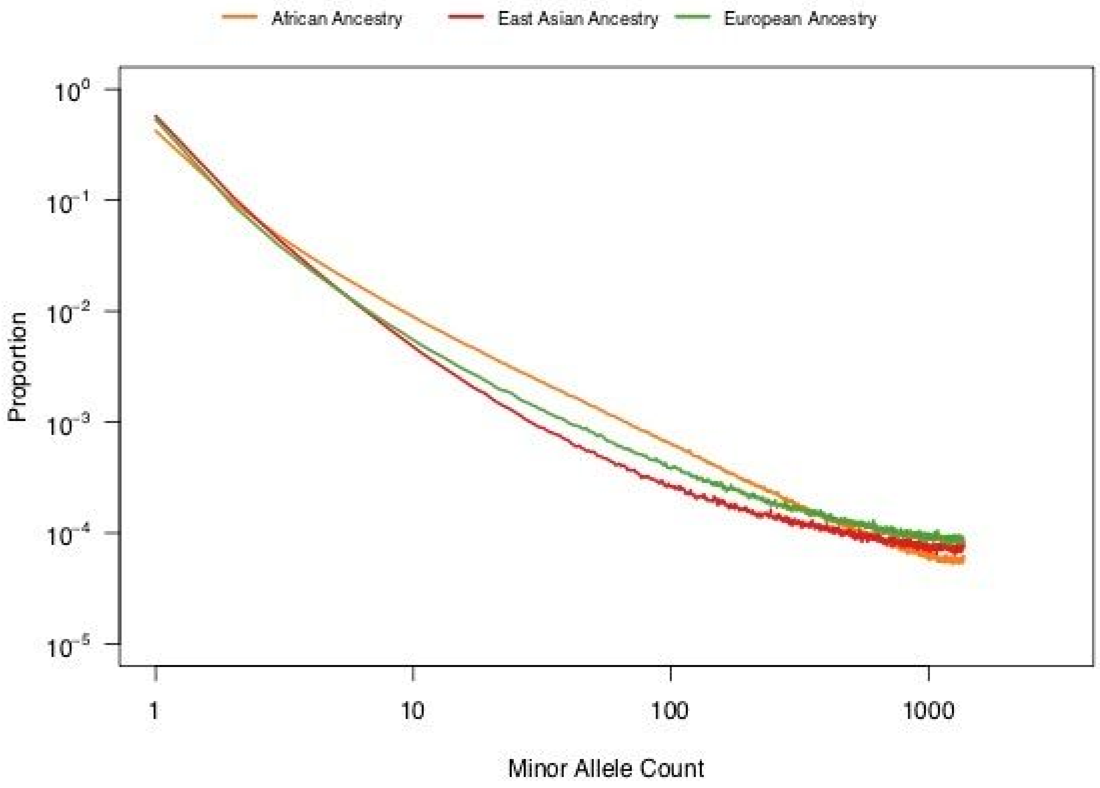

Supplementary Figure 48. Site Frequency Spectrum (SFS) across three major populations. This log-log histogram of the SFS is based on 1,370 unrelated individuals per population. In all three populations we see a shift towards extremely rare variation consistent with exponential growth in the last 5,000-10,000 years. We see a reduction in common variants for East Asian individuals consistent with a protracted bottleneck.

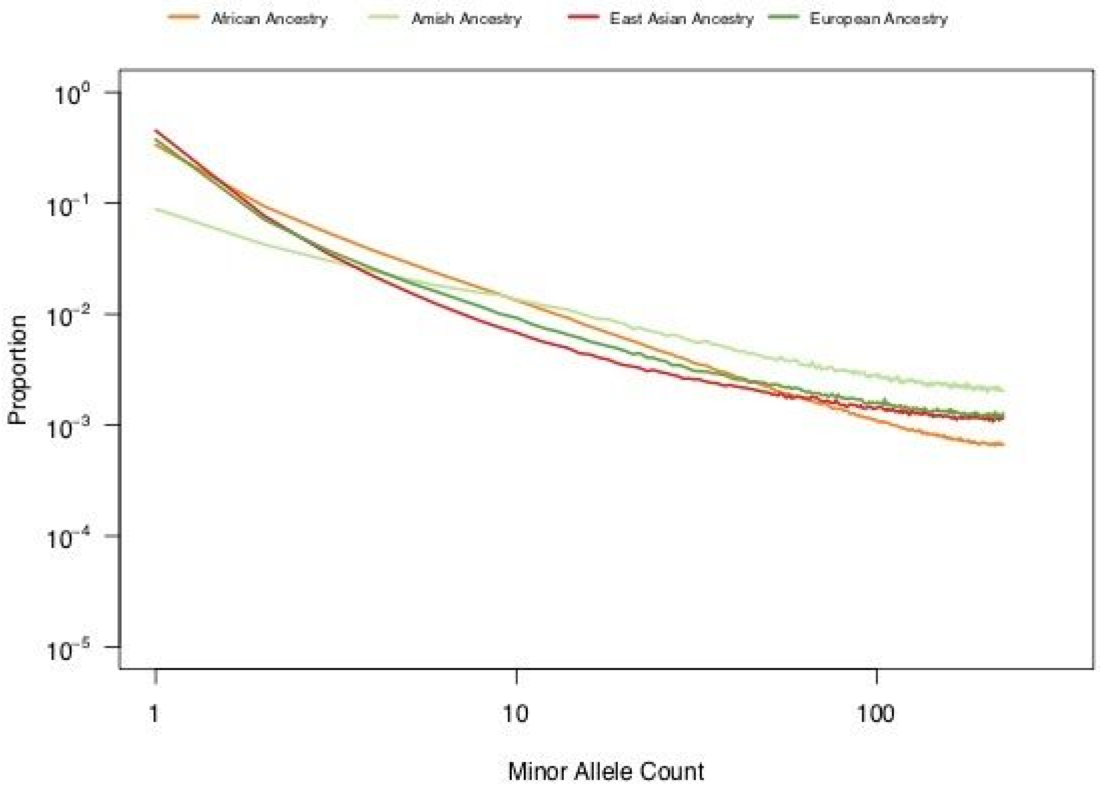

Supplementary Figure 49. This log-log histogram of the SFS is based on 225 unrelated individuals per population. While all four populations show a shift towards extremely rare variation consistent with exponential growth in the last 5,000-10,000 years, this pattern is notably less pronounced in the Amish, which is consistent with the Amish experiencing a very recent bottleneck.

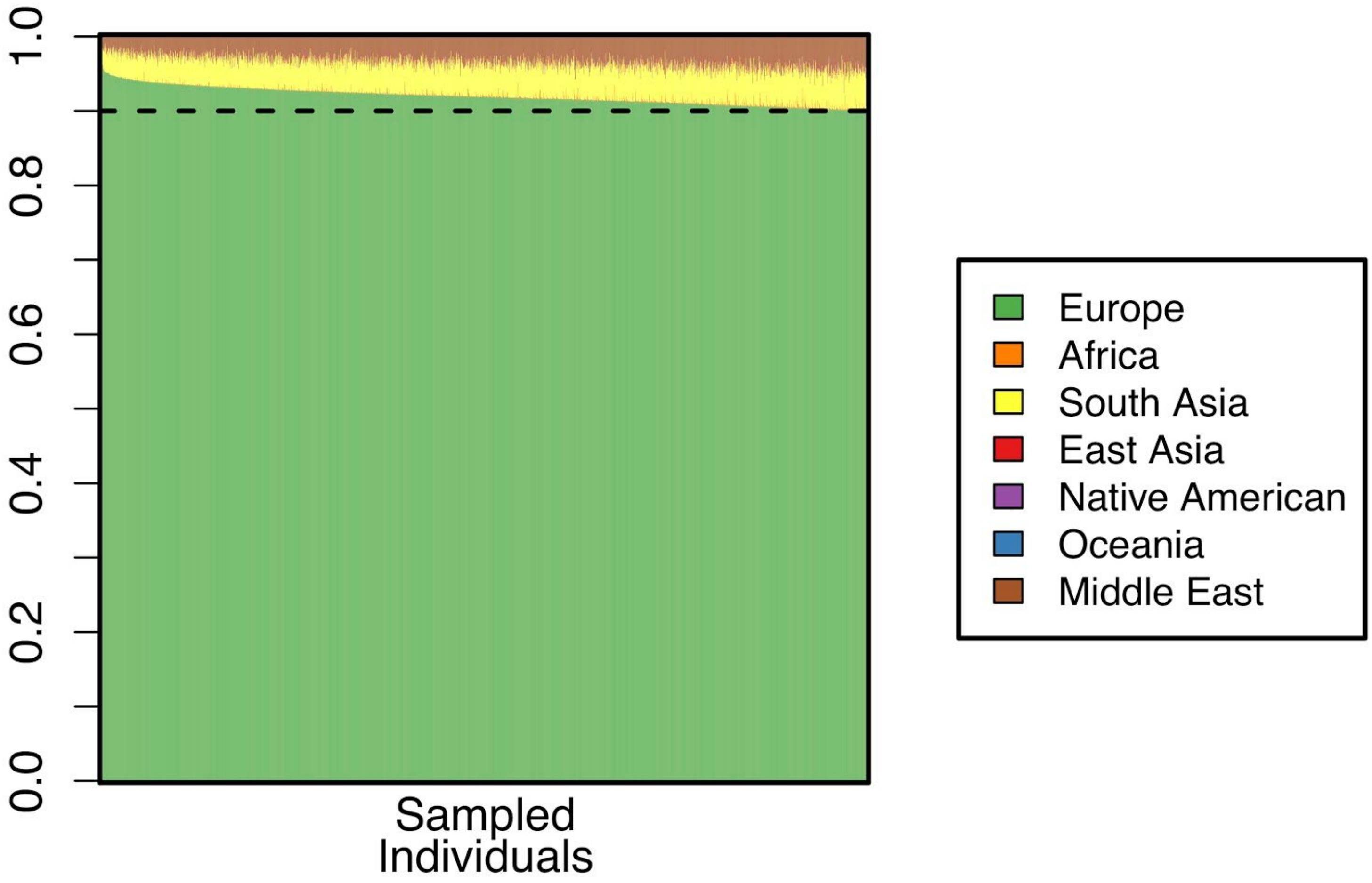

Supplementary Figure 50. Global ancestry of 2,416 European individuals (inferred by RFMix) used for demographic inference. All 2,416 individuals have 90% or greater European ancestry (represented by a dashed line).

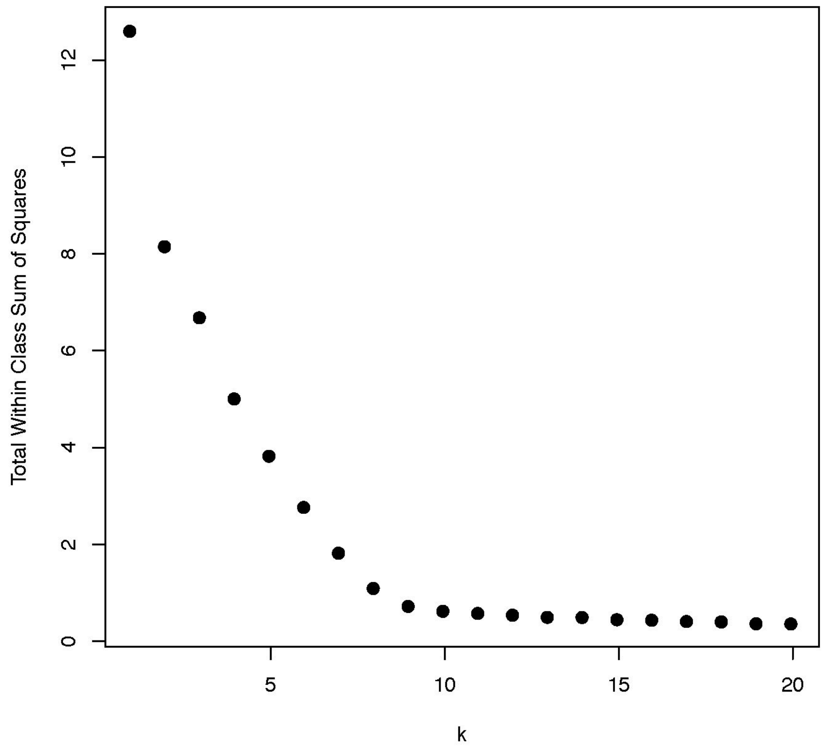

Supplementary Figure 51. Within-class sum of squares versus number of clusters (k) for k-means clustering of PCA coordinates for individuals from TOPMed freeze 3.

References

1. Sherman, R. M. *et al.* Assembly of a pan-genome from deep sequencing of 910 humans of African descent. *Nat. Genet.* (2018) doi:10.1038/s41588-018-0273-y.
2. Kehr, B. *et al.* Diversity in non-repetitive human sequences not found in the reference genome. *Nat. Genet.* **49**, 588–593 (2017).
3. Audano, P. A. *et al.* Characterizing the Major Structural Variant Alleles of the Human Genome. *Cell* **176**, 663–675.e19 (2019).
4. 1000 Genomes Project Consortium *et al.* A global reference for human genetic variation. *Nature* **526**, 68–74 (2015).
5. McCarthy, S. *et al.* A reference panel of 64,976 haplotypes for genotype imputation. *Nat. Genet.* **48**, 1279–1283 (2016).
