# Supplementary material for: Sequencing of 53,831 diverse genomes from the NHLBI TOPMed Program
Source: Nature. 2021 Feb 10;590(7845):290–9. doi: 10.1038/s41586-021-03205-y (PMC7875770; doi:10.1038/s41586-021-03205-y)
Supplement: Supplementary file 2 [file 41586_2021_3205_MOESM2_ESM.pdf]

nature research

Corresponding author(s): Gonçalo Abecasis

Last updated by author(s): November 19, 2020

Reporting Summary

Nature Research wishes to improve the reproducibility of the work that we publish. This form provides structure for consistency and transparency in reporting. For further information on Nature Research policies, see our Editorial Policies and the Editorial Policy Checklist.

Statistics

For all statistical analyses, confirm that the following items are present in the figure legend, table legend, main text, or Methods section.

n/a	Confirmed	
☐	☒	The exact sample size (*n*) for each experimental group/condition, given as a discrete number and unit of measurement
☐	☒	A statement on whether measurements were taken from distinct samples or whether the same sample was measured repeatedly
☐	☒	The statistical test(s) used AND whether they are one- or two-sided<br>*Only common tests should be described solely by name; describe more complex techniques in the Methods section.*
☐	☒	A description of all covariates tested
☐	☒	A description of any assumptions or corrections, such as tests of normality and adjustment for multiple comparisons
☐	☒	A full description of the statistical parameters including central tendency (e.g. means) or other basic estimates (e.g. regression coefficient) AND variation (e.g. standard deviation) or associated estimates of uncertainty (e.g. confidence intervals)
☐	☒	For null hypothesis testing, the test statistic (e.g. *F*, *t*, *r*) with confidence intervals, effect sizes, degrees of freedom and *P* value noted<br>*Give P values as exact values whenever suitable.*
☒	☐	For Bayesian analysis, information on the choice of priors and Markov chain Monte Carlo settings
☒	☐	For hierarchical and complex designs, identification of the appropriate level for tests and full reporting of outcomes
☐	☒	Estimates of effect sizes (e.g. Cohen's *d*, Pearson's *r*), indicating how they were calculated

Our web collection on statistics for biologists contains articles on many of the points above.

Software and code

Policy information about availability of computer code

Data collection	Complete description of data collection and used tools/software is available at https://www.nhlbiwgs.org/data-sets. We also provide a detailed description of TOPMed program's organization and data collection in Supplementary Information 1.1.
Data analysis	All code for TOPMed data quality checks and variant calling is available at https://github.com/statgen/topmed_variant_calling. Code for the WGS and WES data comparisons is available at https://github.com/statgen/sequencing_comparison. Code for modeling singleton distance distribution is available at https://github.com/carjed/topmed_singleton_clusters. Code for identifying novel genetic variants in unmapped reads is available at https://github.com/nygenome/topmed_unmapped. Code for gene burden association tests using rare pLoF variants is available at https://github.com/sgagliano/GeneBurden. Code for the imputed and genotype UK Biobank WES data comparisons is available at https://github.com/sgagliano/UKB_WES_vs_TOPMed_IMP.<br>All programs used are open-source software developed by the academic community and published in scientific literature. For each used program we provide a version number and/or corresponding reference in main and supplementary texts, and methods. These programs include: ADMIXTURE v1.3.0, ANNOVAR, bcftools, Eagle 2.4, EPACTS, Fusera, GATK v4, GENESIS (R package), GotCloud, IBDNe, LiftOver, LOFTEE v0.3-beta, Minimac4, RefineIBD, RFMix, samtools, SeqVarTools (R package), Stargazer, VEP v94, verifyBamID2, WGSA.

For manuscripts utilizing custom algorithms or software that are central to the research but not yet described in published literature, software must be made available to editors and reviewers. We strongly encourage code deposition in a community repository (e.g. GitHub). See the Nature Research guidelines for submitting code & software for further information.

Data

Policy information about availability of data

All manuscripts must include a data availability statement. This statement should provide the following information, where applicable:

- Accession codes, unique identifiers, or web links for publicly available datasets
- A list of figures that have associated raw data
- A description of any restrictions on data availability

TOPMed data used in this manuscript are available through dbGaP. The detailed description of the TOPMed participant consents and data access is provided in Box 1. The dbGaP accession numbers for all TOPMed studies referenced in this paper are listed in Extended Data Tables 2 and 3. Complete list of TOPMed genetic variants with summary level information used in this manuscript is available through the BRAVO variant browser (bravo.sph.umich.edu). TOPMed imputation reference panel described in this manuscript can be used freely for imputation through the NHLBI BioData Catalyst at TOPMed Imputation Server (imputation.biodatacatalyst.nhlbi.nih.gov). The insertion callset (no genotypes) is available on dbGaP under the TOPMed GSR accession phs001974.

The following publicly available databases/datasets were used in the analysis: 1000 Genomes Project (https://www.internationalgenome.org), CADD (https://cadd.gs.washington.edu), ClinVar (https://www.ncbi.nlm.nih.gov/clinvar/), COSMIC (https://cancer.sanger.ac.uk/cosmic), dbSNP (https://www.ncbi.nlm.nih.gov/snp/), Ensembl (https://uswest.ensembl.org/index.html), ExAC (https://gnomad.broadinstitute.org), GENCODE (https://www.gencodegenes.org), GWAS Catalog (https://www.ebi.ac.uk/gwas/), HGDP (https://www.hagsc.org/hgdp/), OMIM (https://omim.org), UK Biobank (https://www.ukbiobank.ac.uk).

Field-specific reporting

Please select the one below that is the best fit for your research. If you are not sure, read the appropriate sections before making your selection.

☒ Life sciences ☐ Behavioural & social sciences ☐ Ecological, evolutionary & environmental sciences

For a reference copy of the document with all sections, see nature.com/documents/nr-reporting-summary-flat.pdf

Life sciences study design

All studies must disclose on these points even when the disclosure is negative.

Sample size	No sample-size calculation was performed. We used all whole genome sequencing data available at the moment at the TOPMed program.
Data exclusions	Sample level QC and variant level QC for TOPMed are extensively described in the methods and supplementary text. There were no exclusions based on participants disease status.
Replication	We did not attempt to reproduce any findings in a separate dataset, because no high depth whole genome sequencing datasets of comparable size were available at the moment of writing.
Randomization	Randomization was not performed, because this is a population-based study aggregating whole-genome sequencing data from >80 different established studies with varying designs.
Blinding	Blinding was not performed, because this is a population-based study aggregating whole-genome sequencing data from >80 different established studies with varying designs.

Reporting for specific materials, systems and methods

We require information from authors about some types of materials, experimental systems and methods used in many studies. Here, indicate whether each material, system or method listed is relevant to your study. If you are not sure if a list item applies to your research, read the appropriate section before selecting a response.

Materials & experimental systems

n/a	Involved in the study
☒	☐ Antibodies
☐	☒ Eukaryotic cell lines
☒	☐ Palaeontology and archaeology
☒	☐ Animals and other organisms
☐	☒ Human research participants
☒	☐ Clinical data
☒	☐ Dual use research of concern

Methods

n/a	Involved in the study
☒	☐ ChIP-seq
☒	☐ Flow cytometry
☒	☐ MRI-based neuroimaging

Eukaryotic cell lines

Policy information about cell lines

Cell line source(s)	Lymphoblastoid cell lines were used for a small number of samples.
Authentication	Compared to previous genetic analysis.
Mycoplasma contamination	Mycoplasma sequence data was aligned to human genome which should avoid any mycoplasma originating reads.
Commonly misidentified lines (See ICLAC register)	*Name any commonly misidentified cell lines used in the study and provide a rationale for their use.*

Human research participants

Policy information about studies involving human research participants

Population characteristics	A primary goal of the TOPMed program is to improve scientific understanding of the fundamental biological processes that underlie heart, lung, blood, and sleep (HLBS) disorders. 37% of all participants come from studies with focus on heart disorders, 33% - on lung disorders, 11% - on blood disorders, 1% - on sleep disorders, and 18% - on multiple phenotypes (Supplementary Figure 1). 41% of all participants have European ancestry, 31% - African ancestry, 15% - Hispanic/Latino, 9% - Asian ancestry, 4% - others/unknown (Supplementary Figures 2 and 39). 60% of individuals are females (Supplementary Figure 40).
Recruitment	TOPMed consists of ~155k participants from >80 different studies with varying designs: prospective cohorts, case-control studies, extended family structures and population isolates. Studies were biased towards individuals with heart, lung, blood, and sleep disorders, and who are willing to participate in research. More details are available at https://www.nhlbiwgs.org.
Ethics oversight	Informed consent was obtained from all participants, and the recruiting institutions for the >80 different TOPMed studies provided ethical oversight (see Box 1 and Supplementary Information 1.1.1-1.1.2). See Supplementary Information 4 for per study ethics statements.

Note that full information on the approval of the study protocol must also be provided in the manuscript.
